# Crownphyrins: Metal‐Mediated Transformations of the Porphyrin‐Crown Ether Hybrids

**DOI:** 10.1002/anie.202211671

**Published:** 2022-11-09

**Authors:** Maksym Matviyishyn, Agata Białońska, Bartosz Szyszko

**Affiliations:** ^1^ Faculty of Chemistry University of Wrocław 14 F. Joliot-Curie St. 50-383 Wrocław Poland

**Keywords:** Crown Ethers, Porphyrinoids, Receptors, Self-Assembly, Supramolecular Chemistry

## Abstract

Crownphyrins are hybrid macrocycles combining structural features of porphyrin and crown ethers. The molecular architecture renders them an intriguing class of hosts capable of binding neutral, and ionic guests. The presence of dynamic covalent imine linkages connecting the dipyrrin segment with the ether chain enables unusual coordination behavior of crownphyrins, as demonstrated by the formation of two classes of strikingly different complexes. The remarkable metal‐mediated expansion to the helical [2+2] macrocyclic complex is reversible. The reaction of the figure‐eight mercury(II) assembly with [2.2.2]cryptand results in ring contraction providing the metal‐free crownphyrin macrocycle.

## Graphical abstract

Crownphyrins, hybrid systems forged from porphyrin and crown ether counterparts, were synthesized. The novel family of macrocycles serves as dynamic, conformationally flexible systems, able to undergo guest‐induced, reversible expansion. The tunable character of their cavities allows for binding neutral and ionic guests.

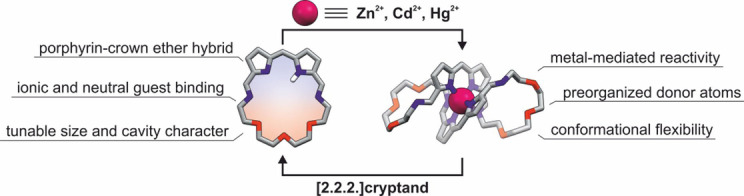

## Introduction

The design of new macrocyclic compounds can be considered a core research area of modern supramolecular chemistry.[^1^, ^2^, ^3^] Porphyrins and crown ethers are two archetypical classes of macrocycles fundamental for the development of functional supramolecular materials.[^4^, ^5^] Both groups of compounds demonstrated numerous intriguing features critical for their applications in coordination chemistry,[^6^, ^7^, ^8^] biomolecular mimetism,[^9^, ^10^, ^11^] self‐assembly,[^12^, ^13^, ^14^, ^15^] medicine,[^16^, ^17^, ^18^, ^19^] and materials sciences.[^20^, ^21^, ^22^] They acted as versatile macrocyclic platforms for artificial receptors,[^23^, ^24^, ^25^] optical sensors,[^26^, ^27^, ^28^, ^29^, ^30^] catalysts,[^31^, ^32^, ^33^, ^34^] mechanically interlocked molecules,[^35^, ^36^, ^37^, ^38^] molecular machines,[^39^, ^40^, ^41^] cages,[^42^, ^43^, ^44^, ^45^] and stimuli‐responsive materials.[^46^, ^47^]

In terms of molecular design, crown ethers and porphyrins belong to strikingly different classes of macrocyclic ligands. Porphyrins are planar, rigid, and aromatic macrocycles that incorporate nitrogen donors in the cavity predesigned to coordinate transition metals.[^48^, ^49^] They typically act as four‐coordinate ligands enforcing the square planar,^[50]^ tetragonal pyramidal,^[51]^ or octahedral geometry^[52]^ of metal centers, although other binding modes have also been described.[^53^, ^54^] Crown ethers whose cavities incorporate oxygen donors, show flexibility, allowing them to adjust conformation to the requirements of the guest.^[3]^ These structural features enable crown ethers to bind not only transition[^32^, ^55^] but, most importantly, alkali and alkaline earth metals.[^56^, ^57^, ^58^] The cavity composed of hydrogen bond‐accepting oxygens rendered them ideal hosts for various organic compounds incorporating functional groups serving as hydrogen bond (HB) donors.[^58^, ^59^]

**Figure 1:**
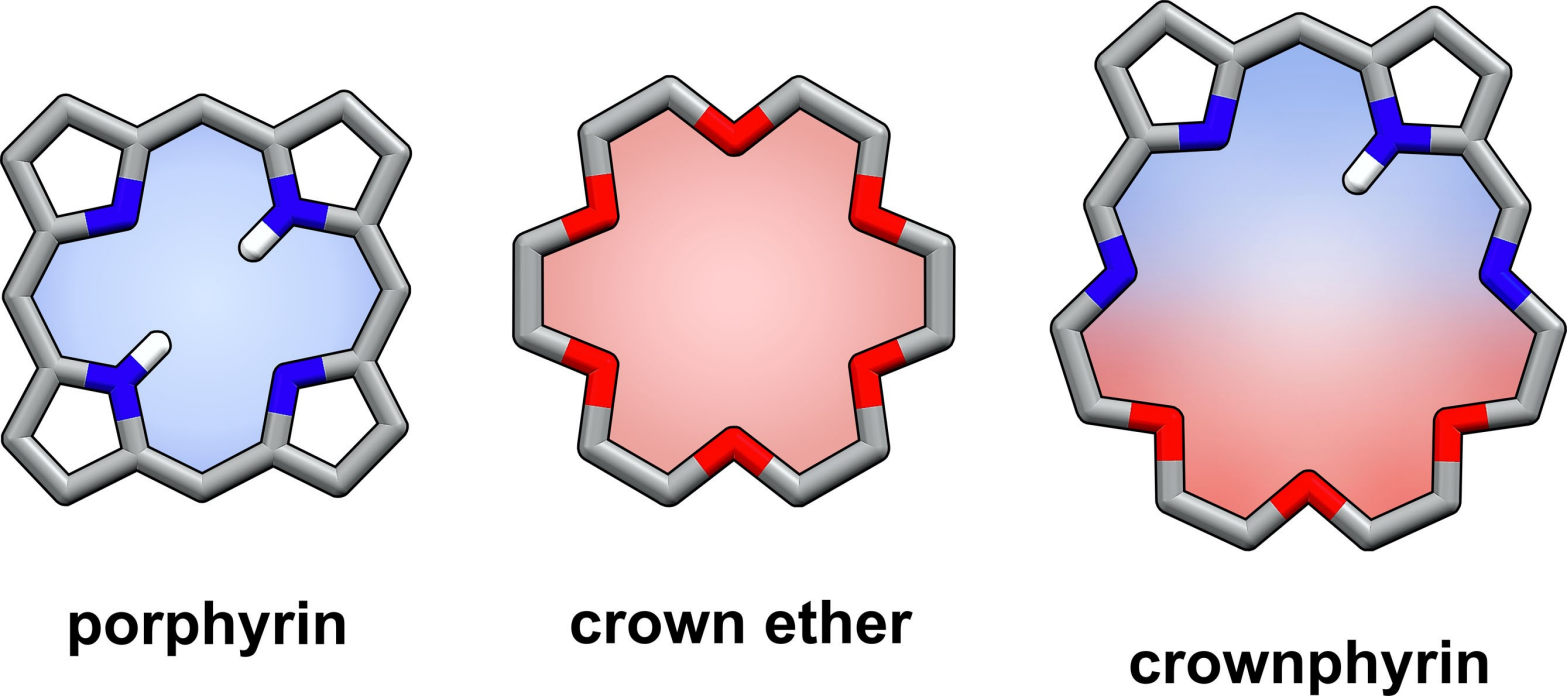
Crownphyrin—a hybrid of porphyrin and crown ether.

Herein we report the hybrid macrocycles bearing structural features of porphyrins and crown ethers, namely crownphyrins (Figure 1). The compounds can be considered imine analogs of expanded porphyrinoids incorporating flexible oligo(ethylene glycol) moieties. Previously the Bowman‐James, Sessler, and Love groups have reported a variety of imine analogs of expanded porphyrins, including accordion porphyrins,[^60^, ^61^, ^62^, ^63^] texaphyrins,[^64^, ^65^, ^66^, ^67^, ^68^, ^69^, ^70^] and Pacman calixpyrroles.[^71^, ^72^, ^73^, ^74^, ^75^]

**Scheme 1:**
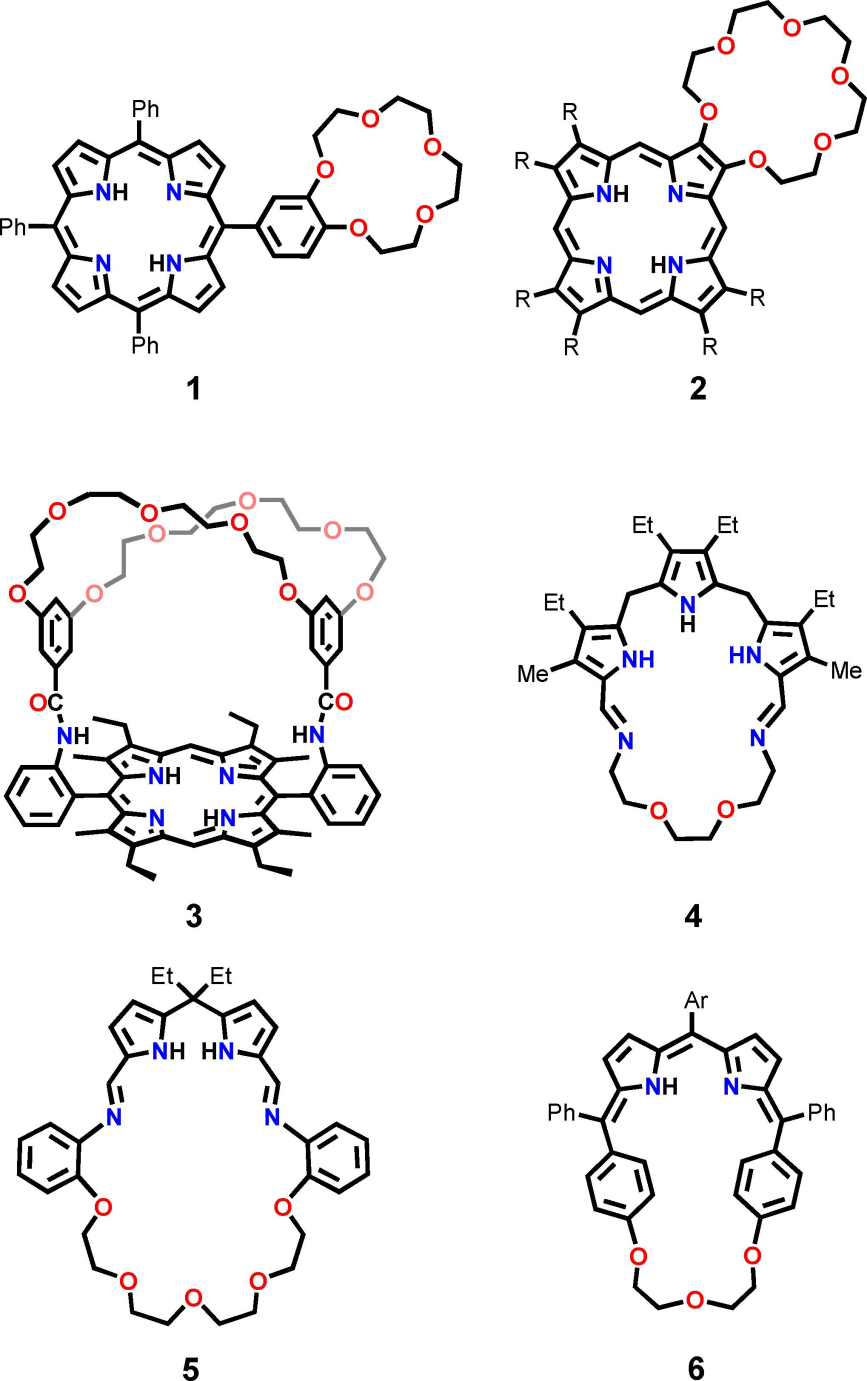
Crown ether‐embedded porphyrins and porphyrinoids.

Several crowned porphyrins were reported in which the crown ether ring was embedded as a *meso*‐substituent (**1**),^[76]^ or attached to the *β*‐positions of a pyrrole ring (**2**) (Scheme 1).^[77]^ Boitrel and others reported systems where the ether formed a strap placed on a single (**3**) or both sides of the macrocycle plane.[^78^, ^79^, ^80^] In fact, the first synthetic attempts toward the macrocyclic system merging the architectures of porphyrin and crown ether were undertaken by Sessler and co‐workers and published in the late‐1980s.[^81^, ^82^] The group succeeded in synthesizing a tripyrrane‐incorporated macrocycle **4** and its derivative with a longer bridge, yet their properties and coordination chemistry remained untackled. In 2012 Love and co‐workers obtained iminocalixpyrroles, e.g., **5**, forming Pacman‐type architectures upon metalation.^[75]^ Ravikanth developed a copper(II)‐sensitive sensor **6** incorporating the structural elements of porphyrin and crown ether.^[83]^

In this contribution, we present a straightforward synthesis of crownphyrins and demonstrate that they act as remarkable macrocyclic ligands, forming two fundamentally different classes of complexes, depending on the requirements of the metal cation. The incorporation of lead(II) into the crownphyrin yielded a monomeric complex with the coordination environment of the metal composed of both—nitrogen and oxygen donors. Remarkably, the coordination of zinc(II), cadmium(II), and mercury(II), although initially leading to lead(II)‐analogous species, enforced the unexpected ring expansion, resulting in the figure‐eight architectures. Importantly, we have found that the same products can be directly targeted in three‐component reactions between diformyldipyrrins, amine, and a metal source, showcasing, to the best of our knowledge, the first use of diformyldipyrrins in the subcomponents self‐assembly.^[84]^

## Results and Discussion

**Scheme 2:**
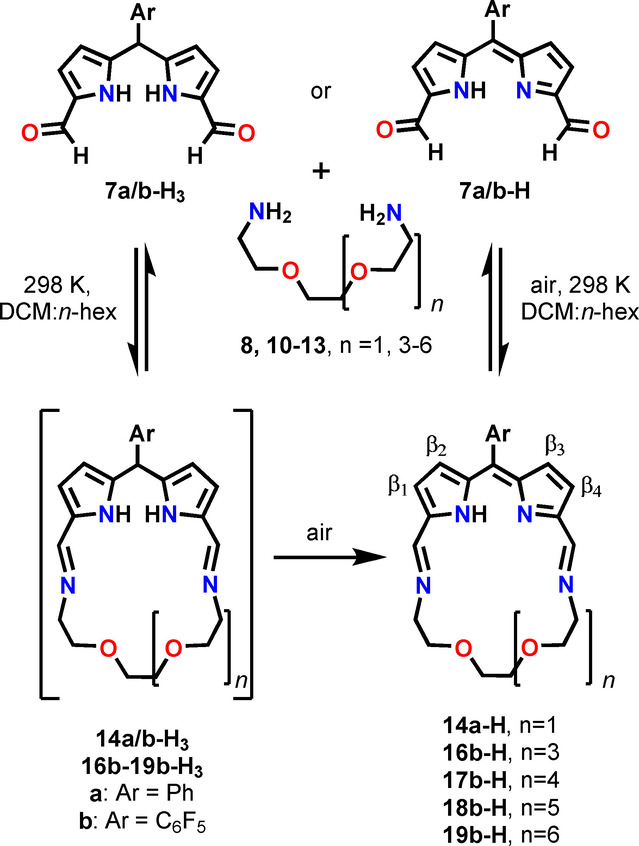
The synthesis of crownphyrinogens and crownphyrins. **15 b‐H** was obtained from 4,7,10‐trioxa‐1,13‐tridecanediamine **9**.

The synthesis of crownphyrins is straightforward and involves the condensation of a dicarboxyaldehyde introducing the dipyrrin motif with an ether chain‐embedded diamine (Scheme 2). Eventually, the diformyldipyrromethanes incorporating the *meso*‐phenyl‐**7 a‐H_3_** and *meso*‐pentafluorophenyl‐ **7 b‐H_3_** substituents were found as suitable building blocks.[^62^, ^85^]

The reactions of **7 a‐H_3_** and **7 b‐H_3_** with diamine **8** were carried out in DCM‐*n*‐hexane for 24 hours at 298 K. Depending on the *meso*‐substituent in the **7‐H_3_** substrate, crownphyrinogen **14 a‐H_3_** or crownphyrin **14 b‐H** were nearly quantitatively obtained. However, even upon prolonged stirring of **14 a‐H_3_** under aerobic conditions, only up to 5–10 % of **14 a‐H** was obtained. Reversely, the presence of pentaflurophenyl in **7 b‐H_3_** did not allow isolating pure **14 b‐H_3_**, as upon exposition to air, spontaneous oxidation to **14 b‐H** was observed. The alternative pathway of **14 b‐H** synthesis involved a direct condensation of **7 b‐H** with **8** (Scheme 2). Following the synthetic protocol a series of crownphyrins **14 b‐H**–**19 b‐H**, differing in the oligo(ethylene glycol) chain length, was synthesized. All diamine reagents were either commercially available (**8**, **9**) or could be easily obtained in reasonable quantities (**10**–**13**).^[86]^

Interestingly, our results are different from Bowman‐James′ reports on the accordion‐porphyrins obtained in the template‐directed reactions between diformyldipyrromethane and propane‐1,3‐diamine.^[60]^ In our case carrying out reactions in the presence of alkali and alkaline‐earth metal templates did not result in the preferable formation of [2+2] condensation products. Thus, the geometric dimensions of the diamine reagent seem to be the factor enforcing crownphyrinogens formation over dimerization.

**Figure 2:**
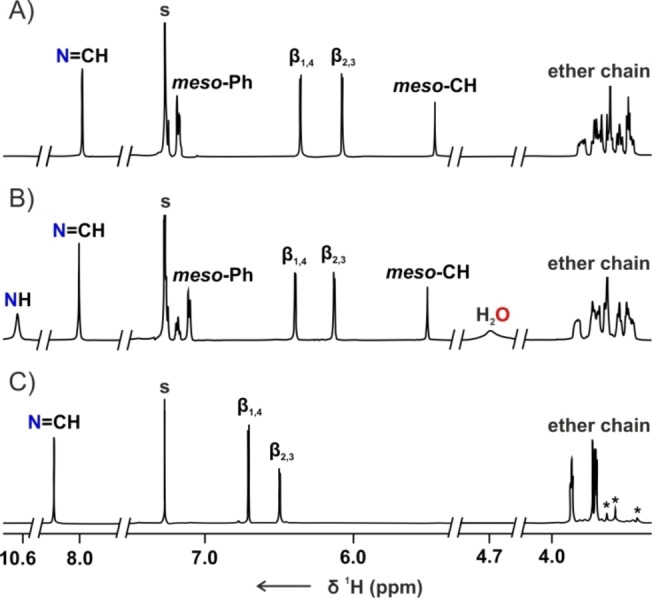
The ^1^H NMR spectra of A) **14 a‐H_3_** (300 K, CDCl_3_, 500 MHz), B) **14 a‐H_3_** (205 K, CDCl_3_, 600 MHz), C) **14 b‐H** (300 K, CDCl_3_, 600 MHz). The impurities were marked with asterisks.

The ^1^H NMR spectrum of **14 a‐H_3_** at 300 K demonstrated the features expected of the macrocycle composed of the *meso*‐aryl dipyrromethane connected to the ether chain through imine linkages (Figure 2A). The azomethine proton resonated at 7.98 ppm, whereas the *β*‐H gave rise to an AB spin system at 6.08 and 6.35 ppm. The *sp* ^3^ hybridization of the *meso*‐carbon was corroborated by the ^13^C NMR chemical shift of 44.10 ppm (Figure S30, Supporting Information) and the position of the CH singlet at 5.46 ppm. The signals corresponding to the crown ether segment demonstrated diastereotopic differentiation. Upon lowering the temperature to 205 K, the H_2_O line at 4.80 ppm appeared, altogether with the NH resonance at 10.63 ppm (Figure 2B, S25, Supporting Information). The chemical shift of the broad signal, down‐field relocated in comparison to the free water resonance in CDCl_3_ (ca. 1.56 ppm), suggested the placement of the water molecule in close proximity to the cavity.

The absence of the *meso*‐CH signal in the ^1^H NMR spectrum ([D]chloroform, 300 K) of **14 b‐H** confirmed the introduction of the dipyrrin motif into the macrocyclic framework (Figure 2C). The resonances attributed to imine and *β*‐pyrrolic protons were found at 8.44, 6.69, and 6.48 ppm. Interestingly, the ^1^H NMR spectrum of **15 b‐H** recorded in [D_2_]DCM at 160 K showed that the tautomerization within the dipyrrin subunit was slowed down sufficiently to observe differentiation of the imine and *β*‐pyrrolic signals (Figure S63, Supporting Information). The broad resonance at 4.15 ppm, detected for **15 b‐H** at 180 K, confirmed the crownphyrin is also amenable to binding water.

**Figure 3:**
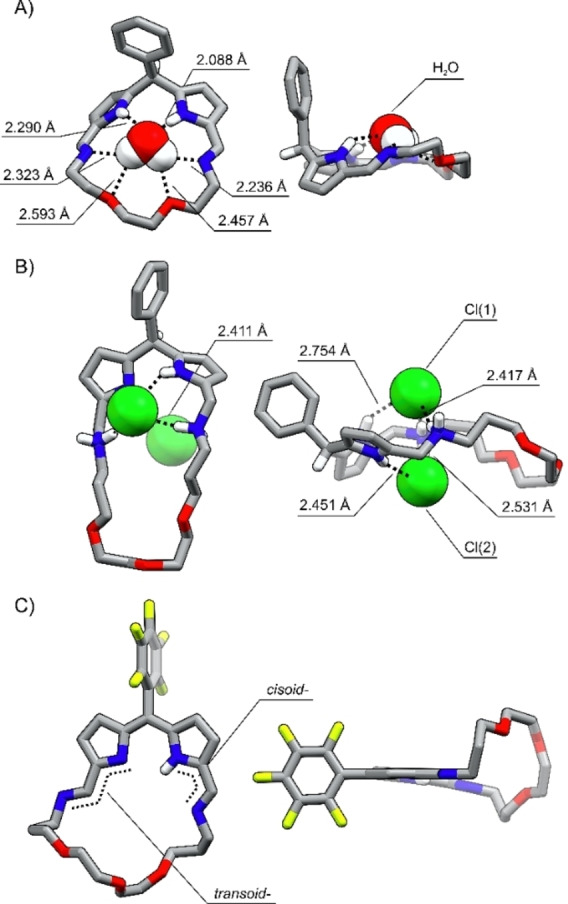
The X‐ray molecular structures of A) **14 a‐H_3_** ⋅ **H_2_O**, B) **15 a‐H_7_** ⋅ **(HCl)_2_**, C) **15 b‐H**. Left: front view, right: side projection with selected protons omitted for clarity.

The XRD studies^[87]^ unambiguously confirmed the identity of **14 a‐H_3_** and **15 b‐H**. The molecular structure of **14 a‐H_3_** demonstrated the macrocycle built of the dipyrromethane segment connected with the ether chain through the imine linkages (Figure 3A). The C_α_−C_meso_ bond lengths equal to 1.501(3) Å, confirmed that both pyrroles are linked through C(*sp* ^2^)−C(*sp* ^3^) single bonds with the tetrahedral *meso*‐carbon. The C=N distances of 1.257(4) Å and 1.267(3) Å are in a range typical for imine bonds.^[88]^ The single water molecule was found nesting within the cavity. **14 a‐H_3_** ⋅ **H_2_O** complex is stabilized through a network of hydrogen bonds with the water protons and pyrrole NH acting as HB donors, oxygen atoms of ether chain, and imine nitrogens being acceptors.

Crownphyrinogens can be reduced in a reaction with excess sodium borohydride (Scheme S2, Supporting Information). The molecular structure of **14 a‐H_7_** revealed the macrocycle retaining the conformation of **14 a‐H_3_** and also binding the water molecule in the cavity (Figure S200, Supporting Information). Interestingly, the larger analog **15 a‐H_7_** crystallized as a dihydrochloride salt, as it interacts with chlorides so effectively that it bound them during the post‐reaction work‐up involving washing with the ammonium chloride solution (Figure 3B). The C−N bond lengths in the protonated secondary amine groups are equal to 1.500(10), 1.479(8), and 1.476(10), 1.489(11) Å. The pyrrole rings tilt in opposite directions at a 67° angle creating two pockets located above and below the mean macrocyclic plane, which supports the binding of chlorides on both sides.

The bond lengths pattern in the dipyrrin segment of **15 b‐H** confirmed the valence structure presented in Scheme 2. The C_α_−N−C_α_ bond angles equal 105.1(4)°and 110.5(4)° correspond to the imine, and amine pyrrolic nitrogens, respectively (Figure 3C). The length of the ether chain enforced its bending out of the diiminodipyrrin plane. The C=N distances are equal to 1.279(6) and 1.276(6) Å, expectedly of the imine C=N bond. One of the imine groups adopted a transoid geometry expelling the nitrogen atom outside the cavity.

**Figure 4:**
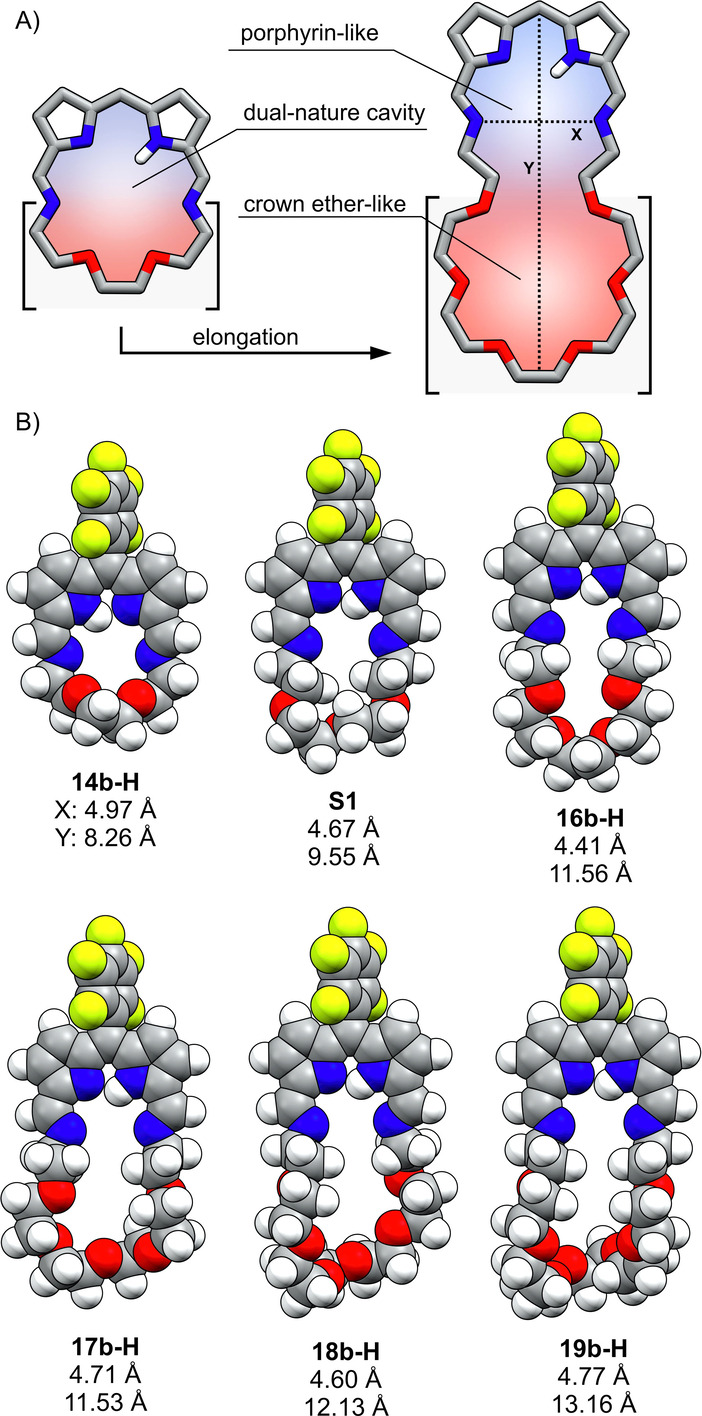
A) The chain elongation strategy; B) the DFT optimized models of **14 b‐H**–**19 b‐H** and **S1**.

Even the smallest representative of the crownphyrin series, i.e. **14 b‐H** has a cavity large enough to classify it as an expanded porphyrinoid.^[89]^ The modification of the ether chain length fundamentally alters the macrocycle‘s structural facets affecting the ligand‘s coordination properties (Figure 4A). Thus, **14 b‐H** is a dual nature, single cavity ligand, in which the interaction with oxygen atoms supplements the coordination through the nitrogen donors. Further elongation of the ether chain enlarged the cavity dimensions, resulting in the macrocycles in which two separate binding pockets could arise. The first, porphyrin‐like is constructed of the dipyrrin moiety and two imine nitrogens preorganized for the coordination. The second one, composed solely of the oligo(ethylene glycol) units, mimics the crown ether pocket, as illustrated for **18 b‐H**/**19 b‐H**.

The presence of an crown ether segment in crownphyrins results in their conformational elasticity, more pronounced than for expanded porphyrinoids constructed of rigid heterocyclic or carbocyclic subunits.[^90^, ^91^] In particular, the cavity of crownphyrin is expected to shrink, expand or bend upon metal binding or guest complexation. To estimate the cavity dimensions of **14 b‐H**–**19 b‐H** in their extended conformations, the DFT calculations (B3LYP/6‐31G(d,p)) were performed (Figure 4B). However, it needs to be pointed out that the pronounced flexibility of crownphyrins limits the precision of geometric dimensions estimation. As shown in Figure 4B the imine N⋅⋅⋅N distance (**X**) varies in a 4.4–5.0 Å range, whereas the vertical dimension (**Y**) ranges from 8.3 to >13.2 Å.

**Scheme 3:**
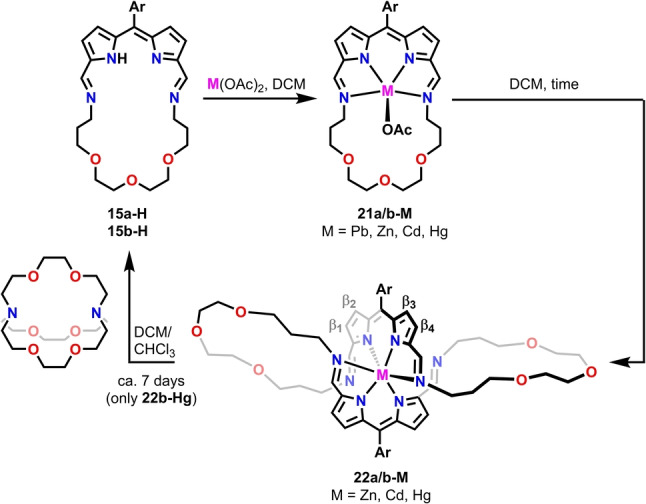
Metal‐mediated reactivity of **15 b‐H**.

To explore the ligand properties of crownphyrins the reactions with metal cations differing in geometric preferences and coordination numbers were carried out for **14‐H** and **15‐H** as the representative examples (Scheme 3).

The lead(II) having the covalent radius of 1.46 Å^[92]^ was selected as the size‐wise adjusted to the cavity of **14‐H** and **15‐H**. In addition, the coordination sphere of Pb^II^ is adaptable, offering a wide range of coordination modes accessible for the crown‐ and heterocrown‐ethers, with the metal reaching high coordination numbers from eight to eleven.[^93^, ^94^, ^95^, ^96^]

Stirring the dichloromethane solution of **15 b‐H** with lead(II) acetate for 24 hours was associated with a distinct color change from orange to purple‐blue, indicative of the formation of **21 b‐Pb**. Metalation of the macrocycle was reflected in the highly altered electronic spectrum of the product containing three absorption bands at 296, 551, and 585 nm (Figure S194, Supporting Information). Interestingly, the same complex type could be obtained directly from **14 a‐H_3_**. In fact, the spontaneous oxidation of dipyrromethane to dipyrrin upon metalation was reported.^[97]^

**Figure 5:**
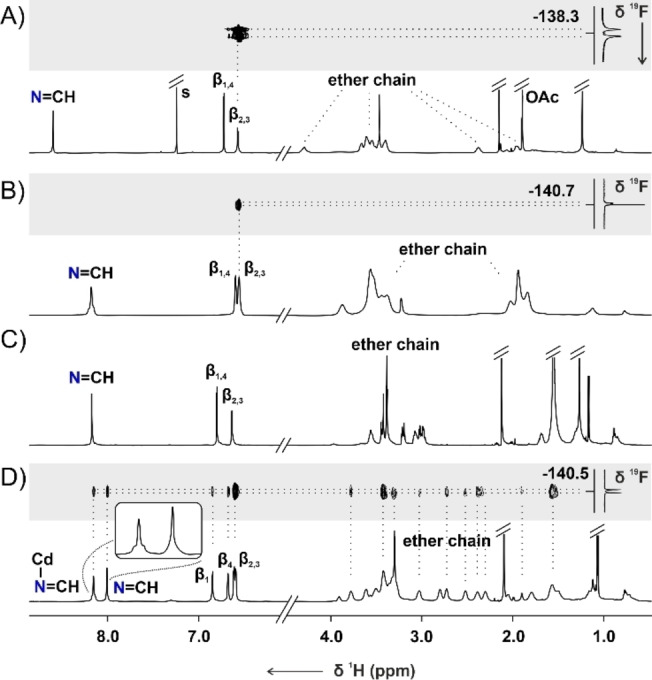
The ^1^H NMR spectra of A) **21 b‐Pb** (300 K, CDCl_3_, 600 MHz), B) **21 b‐Cd** (178 K, CD_2_Cl_2_, 500 MHz), C) **22 b‐Cd** (300 K, CD_2_Cl_2_, 600 MHz), D) **22 b‐Cd** (180 K, CD_2_Cl_2_, 600 MHz). The insets in traces A, B, and D contain part of the ^1^H‐^19^F HOESY spectra.

The ^1^H NMR spectrum ([D]chloroform, 300 K) of **21 b‐Pb** contained two *β*‐pyrrolic signals at 6.57 and 6.72 ppm and the azomethine resonance at 8.60 ppm (Figure 5A). The broadening of the ether chain signals likely arises due to the dynamic nature of the complex, in which oxygen atoms of the ligand undergo reversible binding to the metal cation,^[95]^ but the axial acetate↔water ligand exchange detected in the solution might also contribute. The ^19^F NMR spectrum of **21 b‐Pb** recorded at 293 K revealed five resonances correspondingly to the differentiation of fluorine atoms in the C_6_F_5_‐group, being a consequence of the axial coordination of the acetate (Figure S115, Supporting Information). Both *ortho*‐F correlated to the same *β*_2,3_ protons in the ^1^H‐^19^F HOESY spectrum (inset in Figure 5A, Figure S113, Supporting Information).

**Figure 6:**
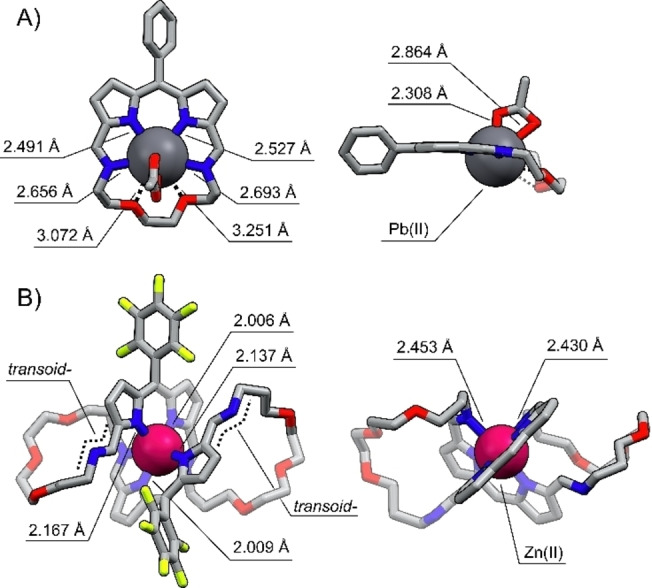
The X‐ray molecular structure of A) **20 a‐Pb** and B) **22 b‐Zn**. Left: front view, right: side projection. Protons were omitted for clarity.

Eventually, lead(II) coordination was confirmed by single crystal XRD carried out for **20 a‐Pb** (Figure 6A).^[85]^ Two nearly identical molecules were found in the asymmetric unit, differing slightly in the arrangement of the ether chain. In both, the metal center lies in the diiminodipyrrin plane coordinating to pyrrolic and imine nitrogens. The Pb‐N_pyrr_ distances remain in the 2.456(6)‐2.527(7) Å range, being notably shorter than Pb‐N_imine_ bonds which vary from 2.636(7) to 2.777(7) Å. The axially coordinated acetate ligands interact with the lead(II) through both oxygen atoms but one Pb−O bond is considerably longer, approaching 2.864(6)/2.899(6) Å, than the other equal to 2.308(6)/2.320(6) Å. The proximity of the metal and the ether chain oxygen atoms vary in a 3.072–3.328 Å range. Although smaller than the sum of van der Waals radii of lead and oxygen equal to 3.54 Å,^[98]^ these values suggested only weak secondary interactions.

To study the reactivity of crownphyrins towards metal cations with the preference for lower coordination numbers and geometrically more demanding than lead(II), the reactions of **15 b‐H** were carried out with Zn^II^, Cd^II^, and Hg^II^ salts (Scheme 3).

The zinc(II), cadmium(II), or mercury(II) acetate addition to a dichloromethane solution of **15 b‐H** was typically carried out for 48 hours and was accompanied by a distinct change of color from orange to purple.

Unexpectedly, in the case of reaction with a Zn^II^ source the mass spectrum recorded for the product mixture demonstrated, aside from the expected **21 b‐Zn** product, the intense signal at the *m/z*=1163.3232 corresponding to a zinc(II) complex of the macrocycle with a doubled molecular mass compared to **21 b‐Zn** (Figure S17,18, Supporting Information). The analogous spectra were recorded for the mixtures obtained from cadmium(II) and mercury(II) acetates (Figures S19–22, Supporting Information).

The monocrystals grown for **22‐M** (M=Zn, Cd, Hg) provided the structural proof for the formation of novel metal‐organic assemblies (Figure 6B, S201–203, Supporting Information).^[85]^ In all complexes, the penta‐ (**22 a‐Zn**, Figure S201, Supporting Information) or hexacoordinate (**22 b‐M**, M=Zn, Cd, Hg) metal cation was found to be bound to the macrocycle built of two diiminodipyrrin segments connected through ether chains. Each side of the macrocycle played a role of a tridentate ligand, coordinating the metal through two pyrrolic and single imine nitrogen, with the other, non‐involved in the coordination, acquiring a transoid geometry. The metal cation adopted a pseudo‐octahedral (pseudo‐square pyramidal for **22 a‐Zn**, Figure S201, Supporting Information) geometry, arranging two iminodipyrrin ligands in a meridional fashion, and imposing the figure‐eight architecture. The M−N_pyrr_ distances varied in ranges: 2.009(3)–2.180(3) Å for **22 b‐Zn**, 2.222(7)–2.372(7) for **22 b‐Cd**, and 2.160(7)–2.469(7) Å in **22 b‐Hg**, whereas the M‐N_imine_ bonds were substantially longer, approaching 2.437(3)/2.469(3) Å, 2.491(7)/2.671(8) Å, and 2.671(8)/2.780(2) Å, respectively.

As could be inferred from the molecular structures of **22‐M**, their formation required a metal‐enforced reassembly of **15 b‐H**. To understand the mechanism of **22‐M** formation the reactions of **15 b‐H** with the Zn^II^, Cd^II^, and Hg^II^ acetates were carried out under mass spectrometry and UV/Vis spectroscopy control (Figures S15–22, S197–199, Supporting Information).

**Figure 7:**
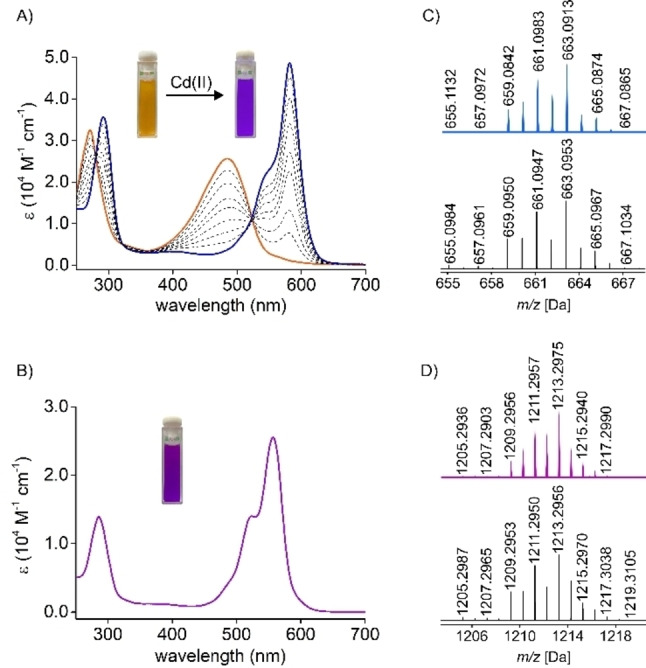
Left: the UV/Vis spectra (DCM, 278 K) A) recorded in the course of the titration of **15 b‐H** with Cd(OAc)_2_ in MeOH; B) of **22 b‐Cd** (298 K). Right: the high‐resolution mass spectra (experimental: top, simulated: bottom) of C) **21 b‐Cd**; D) **22 b‐Cd**.

The addition of cadmium(II) acetate aliquots to the solution of **15 b‐H** was accompanied by a continuous color change from orange to purple‐blue, reflecting the alteration of the UV/Vis spectrum (Figure 7A). Upon the addition of one equivalent of cadmium(II) acetate, the bathochromic shift of the **15 b‐H** band from 273 nm to 295 nm was observed, accompanied by the disappearance of the broad absorption at 485 nm and the rise of a more narrow one at the 585 nm (sh. 557 nm). The high‐resolution mass spectrum demonstrated a major signal at *m/z*=663.0913, corresponding to **21 b‐Cd** (Figure 7C). Once formed, it started transforming yielding eventually the purple **22 b‐Cd**. The transformation was associated with the 28 nm blue shift of the intense absorption to 557 nm (Figure 7B). The presence of signals at *m/z*=607.1525 (+2), and 1213.2975 (+1) in the mass spectrum confirmed the expansion to **22 b‐Cd** (Figure 7D).

**21 b‐M** (M=Zn, Cd, Hg) were obtained under the ^1^H NMR control at low temperature, which slowed down the following transformations (Figures S119, 130, Supporting Information). The ^1^H NMR spectrum of **21 b‐Cd** in [D_2_]DCM at 178 K shared a considerable resemblance to **21 b‐Pb** (Figure 5B). The cadmium(II) coordination was explicit, as confirmed by the ^111/113^Cd satellites for *β*‐ and azomethine protons signals at 6.55, 6.59, and 8.18 ppm, respectively. **21 b‐Zn** and **21 b‐Hg** complexes demonstrated resonances in similar regions (Figure S129, Supporting Information).

**Scheme 4:**
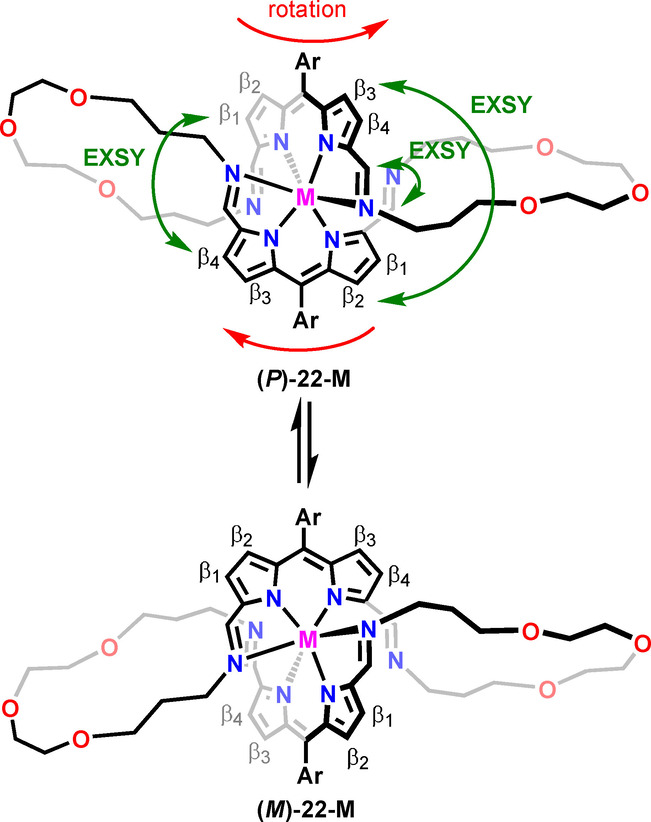
The helicity inversion in **22‐M**.

The ^1^H NMR spectrum of **22 b‐Cd** at 300 K does not allow to distinguish it from **21 b‐Cd** based on the symmetry (Figure 5C). The spectra of the corresponding **22 b‐Zn** and **22 b‐Hg** complexes are also similar to these of the respective **21 b‐M** species, preventing structure determination solely on the number and chemical shifts of the resonances. In particular, the ^1^H NMR spectra of **22 b‐M** did not reflect the symmetry of the molecule detected in the solid state (Figure 6B). Namely, two types of imine groups were identified in the crystal structure—one of which was uninvolved in the metal coordination. In contrast, only a single azomethine proton signal was identified in the ^1^H NMR spectra at 300 K. Thus, **22‐M** was expected to adopt a different conformation in the solution or to be involved in a dynamic process, increasing its effective symmetry. Lowering the temperature of **22 b‐Cd** in [D_2_]DCM resulted in a gradual broadening of resonances, followed by their doubling below 200 K (Figure S149, Supporting Information). At 180 K the spectrum corresponded to the solid‐state symmetry of **22 b‐Cd** (Figure 5D). Four *β*‐pyrrolic lines were located in the 6.5–6.9 ppm range, alongside two azomethine signals at 8.15 and 8.01 ppm. The imine resonances revealed a critical difference; namely detection of the ^111/113^Cd satellites only for one of them was consistent with the presence of coordinating and non‐coordinating imine groups in the molecule. The pattern of the EXSY cross‐peaks detected in the ROESY spectrum (Figure S159–161, Supporting Information) for the protons of metal‐coordinated and non‐coordinating imine groups, as well as respective *β*‐H's, reflected the exchange indicative of the helicity inversion of **22 b‐M** enantiomers (Scheme 4).

The difference between **21 b‐M** and **22 b‐M** became apparent upon a comparison of the ^1^H‐^19^F HOESY spectra (Figures 5B, D). In particular, for **21 b‐Cd**, a single pair of the correlation peaks between two *ortho*‐F and *β*_2,3_ protons was identified. In contrast, a set of cross‐peaks, including F⋅⋅⋅HC=N−Cd, F⋅⋅⋅HC=N, F⋅⋅⋅*β*_1‐4_, and several F⋅⋅⋅CH_2_, was identified for **22 b‐Cd** due to much closer distances between the *meso*‐C_6_F_5_ group and protons of the helical macrocycle. Thus, the HOESY experiment can be, in this case, exploited not only to probe the conformation of the assembly but also its size.

To our best knowledge, the metal‐mediated crownphyrin expansion consists of the unprecedented [1+1] to [2+2] transformation of the iminopyrrole macrocycle. In fact, the Schiff porphyrinoids expansion reactions are very rare. Love described the [2+2] to [3+3] enlargement of the calixpyrrole macrocycle upon zinc(II) coordination.^[99]^

**Figure 8:**
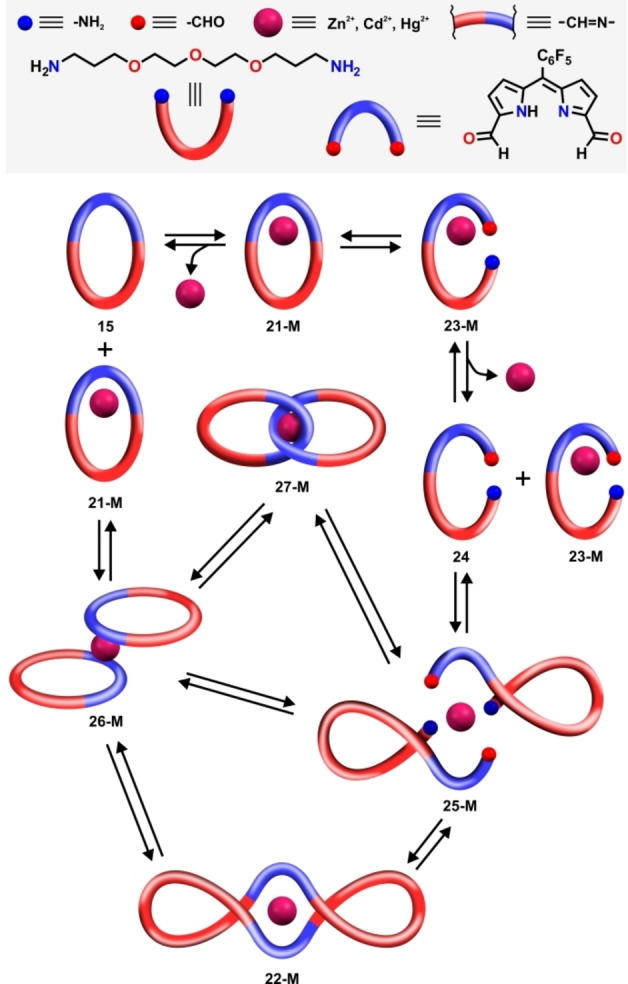
A plausible mechanism of **22‐M** formation.

Two mechanistic pathways resulting in the **21‐M** to **22‐M** expansion can be proposed (Figure 8). The first one involves the cleavage of a single imine bond of **21‐M**, producing the open‐chain complex **23‐M**. Its reaction with demetalated **24**, enforced by maximizing the occupation of the cation‘s coordination sphere, would generate an open chain dimer **25‐M**. The ring‐closing reaction of the latter could follow two routes, yielding the figure‐eight **22‐M** or a catenand **27‐M**. The shift of the equilibrium towards **22‐M** likely resulted from the structural limitations of intermediates, namely the presence of a bulky *meso*‐aryl group, preventing **27‐M** formation. The alternative pathway could involve the formation of a sandwich‐like complex **26‐M** from the reaction of **21‐M** and **15**. Once formed, **26‐M** could rearrange into **27‐M**, or undergo partial or complete imine bonds cleavage to **25‐M**, followed by ring‐closing reaction yielding **22‐M**.^[100]^

**Scheme 5:**
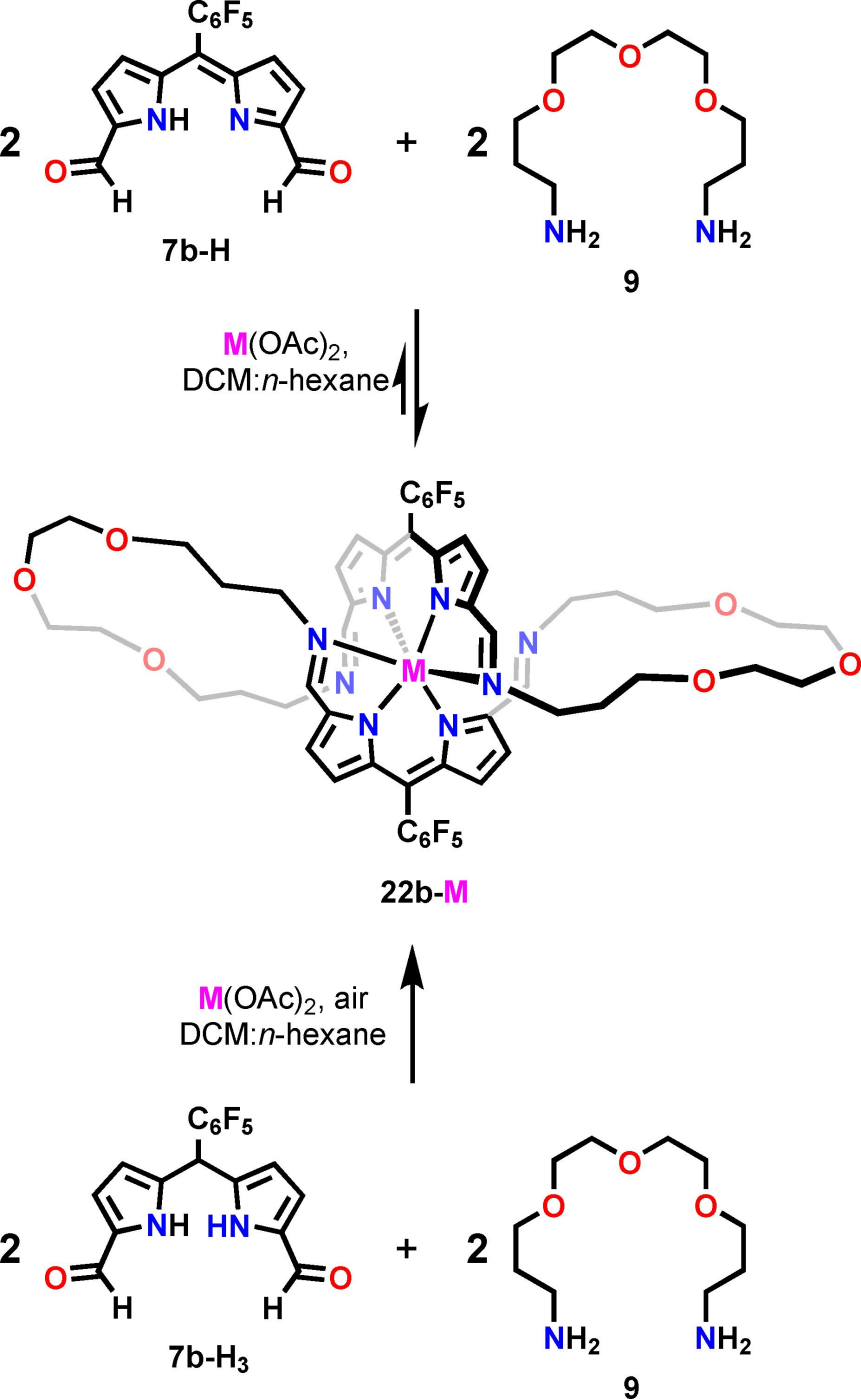
The formation of **22 b‐M** via the subcomponents self‐assembly.

In fact, similar transformations, namely the formation of the helicate and catenand, were reported by Nitschke as alternative pathways governing the outcome of subcomponents self‐assembly of 2,2′‐(ethane‐1,2‐diylbis(oxy))bis(ethan‐1‐amine) and phenanthroline‐2,9‐dicarboxaldehyde in the presence of copper(I) template.^[101]^ To verify if **22‐M** can be obtained directly from the acyclic building blocks, we carried out a three‐component reaction between **7 b‐H**, diamine **9**, and zinc(II) acetate in a 2 : 2 : 1 ratio (Scheme 5). An alternative experiment was also carried out in which **7 b‐H** was replaced by **7 b‐H_3_**. Both processes worked analogously, producing the helical **22 b‐Zn** as the major product, although accompanied by minor species which could not be identified. It is worth pointing out that only one of the two assembly processes is fully reversible, as the formation of **22 b‐Zn** required the oxidation of dipyrromethane to dipyrrin prior to the assembly, or at the macrocycle formation stage, limiting the reversibility.

Surprisingly, we have found that in the case of **22 b‐Hg**, the expansion of the macrocycle is reversible. The prolonged (ca. 7 days) stirring of the solution of **22 b‐Hg** with [2.2.2]cryptand resulted in a mercury(II) removal and the transformation of the helical complex into **15 b‐H** (Scheme 3; Figure S182, 183, Supporting Information), yet some unidentified byproducts were also formed. The same reaction carried out for **22 b‐Zn** and **22 b‐Cd** did not result in analogous contraction. The reassembly process can be understood in terms of the dynamic equilibrium involving **22 b‐Hg**, **21 b‐Hg**, **7 b‐H**, diamine **9**, and mercury(II). To our knowledge, only a single example of iminopyrrole macrocycle contraction was to date reported in the literature.^[99]^

## Conclusion

The combination of structural facets of porphyrins and crown ethers resulted in the group of novel hybrid macrocycles, i.e. crownphyrins. They were demonstrated to be conformationally flexible and structurally adjustable systems, prone to binding neutral, cationic, and anionic guests. The ligand character of the crownphyrin is highly tunable; depending on the length of the ether chain, its cavity can be considered a dual nature, providing nitrogen and oxygen donors simultaneously but at a proper size, two discrete pockets, providing chemically diverse environments could form.

The presence of dynamic covalent imine bonds linking the dipyrrin segment with the ether chain remarkably affects the crownphyrin reactivity toward metal cations. Depending on the requirements of the metal, the macrocycle could form a monomeric complex with a secondary interaction between the cation and ligand oxygens or expand providing the octahedral arrangement of donors. Exceptionally, the same architecture could be obtained directly from pyrrole building blocks and diamine in the presence of a metal template, showcasing the exploitation of pyrrolic synthons in the subcomponents self‐assembly approach.

**Figure 9:**
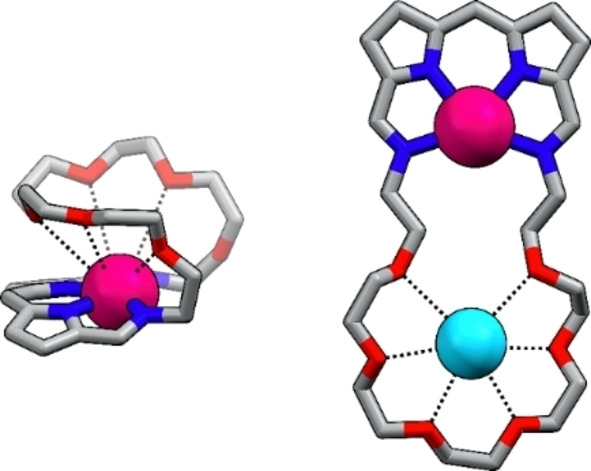
Selected structural motifs attainable for complexes of large crownphyrins.

The described metal‐mediated transformations of crownphyrins are likely just the tip of the iceberg hiding the reactivity these ligands will reveal. The crownphyrins with two well‐defined cavities are preadapted for binding more than one cation providing exciting structural motifs, and opening new routes for the coordination chemistry of flexible porphyrinoids (Figure 9). Furthermore, the introduction of catalytically‐active metals into the crownphyrin core could allow for the construction of functional supramolecular systems capable of performing the controlled transformations on the bound guests.

## Conflict of interest

The authors declare no conflict of interest.

1

## Supporting information

As a service to our authors and readers, this journal provides supporting information supplied by the authors. Such materials are peer reviewed and may be re‐organized for online delivery, but are not copy‐edited or typeset. Technical support issues arising from supporting information (other than missing files) should be addressed to the authors.

Supporting Information

Supporting Information

Supporting Information

## Data Availability

The data that support the findings of this study are available in the supplementary material of this article.
