# Supplementary material for: Crownphyrins: Metal‐Mediated Transformations of the Porphyrin‐Crown Ether Hybrids
Source: Angew Chem Int Ed Engl. 2022 Nov 9;61(49):e202211671. doi: 10.1002/anie.202211671 (PMC10098552; doi:10.1002/anie.202211671)
Supplement: Supplementary file 1 — Supporting Information [file ANIE-61-0-s002.pdf]

Angewandte Chemie
Eine Zeitschrift der Gesellschaft Deutscher Chemiker

Supporting Information

Crownphyrins: Metal-Mediated Transformations of the Porphyrin-Crown Ether Hybrids

*M. Matviyishyn, A. Białońska, B. Szyszko**

Table of Contents

Table of Contents 2
Experimental Procedures 3
- **Instrumentation** 3
- **Synthesis** 4
- **High-resolution mass spectra** 12
- **The NMR spectra of 14a-H_3** 23
- **The NMR spectra of 14a-H_7** 32
- **The NMR spectra of 14b-H** 37
- **The NMR spectra of 15a-H_7** 39
- **The NMR spectra of 15b-H** 42
- **The NMR spectra of 16b-H** 52
- **The NMR spectra of 17b-H** 54
- **The NMR spectra of 18b-H** 56
- **The NMR spectra of 19b-H** 58
- **The NMR spectra of 20a-Pb** 60
- **The NMR spectra of 20b-Pb** 61
- **The NMR spectra of 21b-Pb** 67
- **The NMR spectra of 21b-Cd** 73
- **The NMR spectra of 21b-Hg** 80
- **The NMR spectra of 22a-Zn** 85
- **The NMR spectra of 22b-Zn** 89
- **The NMR spectra of 22b-Cd** 90
- **The NMR spectra of 22b-Hg** 103
- **UV-vis spectra** 111
- **X-ray structure models** 118
- **DFT-calculated Cartesian coordinates of 14b-H.** 120
- **DFT-calculated Cartesian coordinates of S1.** 120
- **DFT-calculated Cartesian coordinates of 16b-H.** 120
- **DFT-calculated Cartesian coordinates of 17b-H.** 121
- **DFT-calculated Cartesian coordinates of 18b-H.** 121
- **DFT-calculated Cartesian coordinates of 19b-H.** 122

References 123

Experimental Procedures

Instrumentation

NMR spectroscopy: The ^{1}H NMR were recorded on high-field Bruker spectrometers (600 and 500 MHz), equipped with a broadband inverse gradient probe head and a high-field JEOL (500 MHz) spectrometer, equipped with a 5 mm wide wideband probe. The spectra were referenced to the residual solvent signal ([D]chloroform – 7.24 ppm, [D_2]dichloromethane – 5.32 ppm). Two-dimensional NMR spectra were recorded with 2048 data points in the t_2 domain and up to 1024 points in the t_1 domain, with a 1 s recovery delay.

Mass spectrometry: The mass spectra were recorded on Shimadzu q-TOF LCMS 9030 and Bruker Q-TOF-MS/MS maXis impact spectrometers.

UV/Vis spectroscopy: Electronic spectra were recorded on a Varian Carry-60 Bio spectrophotometer.

X-ray diffraction data:

Single- crystal X-ray diffraction data were collected at 100 K, on KUMA Xcalibur, (Sapphire2 CCD detector) (**14a-H$_3$**) or Rigaku XtaLAB Synergy R, DW system (HyPix-Arc 150) (**14a-H$_7$**, **15a-H$_7$•2[HCl]**, **15b-H**, **20a-Pb**, **22a-Zn**, **22b-Zn**, **22b-Cd**, **22b-Hg**) κ-geometry diffractometers using *Mo* K*α* or *Cu* K*α* radiation. Data reduction and analysis were carried out with the CrysAlis Pro programs.[1] The structures were solved by direct methods and refined with the full-matrix least-squares technique using the *SHELXS*[2] /*SIR2014*[3], and *Shelxl-2018/3*[4] programs. Hydrogen atoms were placed at calculated positions or were found on the Δρ map. Before the last cycle of refinement, all H atoms were fixed and were allowed to ride on their parent atoms. Anisotropic displacement parameters were refined for all non-hydrogen atoms. However, EADP constraints, SIMU or ISOR restraints were applied for disordered components in **14a-H$_7$**, **15a-H$_7$•2[HCl]**, **15b-H**, **22a-Zn**, **22b-Cd**, **22b-Hg**. The geometry of the disordered ether chain in **22b-Cd** and **22b-Hg** was restrained applying SADI command, and the geometry of the disordered C_6F_5 group in **22b-Cd** was restrained applying FLAT command. The occupancy factor for the disordered components was refined. For disordered solvent molecules in **22b-Zn** the SQUEEZE procedure[5] was applied.

Crystal data for compound **14a-H$_3$**: $C_{23}H_{26}N_4$ $_2$·H_2O, M = 408.49, monoclinic, $P2_1/n$, a = 12.185(3) Å, b = 9.096(2) Å, c = 20.334(3) Å, β = 102.14(3)°, V = 2203.3(8) Å^3, Z = 4, D_c = 1.231 Mg m^{-3}, T = 100(2) K, R = 0.0762, wR = 0.1694 (3272 reflections with $I > 2\sigma(I)$) for 275 variables, CCDC 2192561;

Crystal data for compound **14a-H$_7$**: $C_{23}H_{30}N_4O_2$·H_2O, M = 412.52, orthorhombic, $Pnma$, a = 8.520(2) Å, b = 17.383(3) Å, c = 15.051(3) Å, V = 2229.1(8) Å^3, Z = 4, D_c = 1.229 Mg m^{-3}, T = 100(2) K, R = 0.0746, wR = 0.1814 (2308 reflections with $I > 2\sigma(I)$) for 276 variables, CCDC 2192562;

Crystal data for compound **15a-H$_7$•2[HCl]**: $C_{27}H_{40}N_4O_3^{+2}$·$2Cl^-$, M = 539.53, monoclinic, $P2_1/c$, a = 13.411(5) Å, b = 10.170(3) Å, c = 20.383(6) Å, β = 97.98(3)°, V = 2753.1(16) Å^3, Z = 4, D_c = 1.302 Mg m^{-3}, T = 100(2) K, R = 0.0751, wR = 0.2123 (3240 reflections with $I > 2\sigma(I)$) for 489 variables, CCDC 2192563;

Crystal data for compound **15b-H**: $C_{27}H_{27}F_5N_4O_3$, M = 550.52, orthorhombic, $Pca2_1$, a = 22.711(3) Å, b = 14.166(3) Å, c = 8.134(2) Å, V = 2616.9(9) Å^3, Z = 4, D_c = 1.397 Mg m^{-3}, T = 100(2) K, R = 0.0581, wR = 0.1380 (3197 reflections with $I > 2\sigma(I)$) for 371 variables, CCDC 2192564;

Crystal data for compound **20a-Pb**: $C_{25}H_{26}N_4O_4Pb$, M = 653.69, monoclinic, $P2_1/c$, a = 19.442(2) Å, b = 13.794(2) Å, c = 19.055(2) Å, β = 113.33(3)°, V = 4692.4(14) Å^3, Z = 8, D_c = 1.851 Mg m^{-3}, T = 100(2) K, R = 0.0509, wR = 0.1403 (14374 reflections with $I > 2\sigma(I)$) for 614 variables, CCDC 2192565;

Crystal data for compound **22a-Zn**: $C_{54}H_{62}N_8O_6Zn$, M = 984.48, triclinic, P-1, a = 8.507(2) Å, b = 17.186(3) Å, c = 17.472(3) Å, α = 100.29(2)°, β = 96.09(2)°, γ = 102.48(3)°, V = 2426.0(9) Å^3, Z = 2, D_c = 1.348 Mg m^{-3}, T = 100(2) K, R = 0.0338, wR = 0.0890 (8945 reflections with $I > 2\sigma(I)$) for 650 variables, CCDC 2192566;

Crystal data for compound **22b-Zn**: $C_{54}H_{52}F_{10}N_8O_6Zn$ [+ solvent], *M* = 1164.40, monoclinic, *P*2$_1$/*c*, *a* = 13.301(3) Å, *b* = 15.701(3) Å, *c* = 28.077(4) Å, β = 94.99(2)°, *V* = 5841.3(19) Å^3, *Z* = 4, D_c = 1.324 Mg m^{-3}, *T* = 100(2) K, *R* = 0.0617, *wR* = 0.1691 (7895 reflections with *I* > 2σ(*I*)) for 713 variables, CCDC 2192567;

Crystal data for compound **22b-Cd**: $C_{54}H_{52}CdF_{10}N_8O_6$, *M* = 1211.43, monoclinic, *P*2$_1$/*c*, *a* = 23.482(3) Å, *b* = 8.277(2) Å, *c* = 28.339(3) Å, β = 106.77(2)°, *V* = 5273.7(16) Å^3, *Z* = 4, D_c = 1.526 Mg m^{-3}, *T* = 100(2) K, *R* = 0.1015, *wR* = 0.3045 (5483 reflections with *I* > 2σ(*I*)) for 1037 variables, CCDC 2192569;

Crystal data for compound **22b-Hg**: $C_{54}H_{54}F_{10}HgN_8O_7$, *M* = 1317.64, monoclinic, *P*2$_1$/*c*, *a* = 23.216(3) Å, *b* = 8.308(2) Å, *c* = 28.491(3) Å, β = 106.91(2)°, *V* = 5257.7(16) Å^3, *Z* = 4, D_c = 1.665 Mg m^{-3}, *T* = 100(2) K, *R* = 0.0696, *wR* = 0.1920 (8013 reflections with *I* > 2σ(*I*)) for 967 variables, CCDC 2192568;

Theoretical calculations: Geometry optimizations were carried out within unconstrained C_1 symmetry in vacuo using Gaussian software.[6] Starting coordinates were derived from preoptimized models taking into account all imposed geometrical restrictions or from crystal structures. Harmonic frequencies were calculated using analytical second derivatives as a verification of local minimum achievement, with no imaginary frequencies observed. Optimizations were performed at B3LYP/6-31G(d,p)[7,8] level of theory.

Synthesis

[D]Chloroform, [D$_2$]dichloromethane, and solvents for synthesis were prepared directly before use by running through a basic alumina column. Reagents not listed here were used as received. 2,2'-(Ethylenedioxy)bis(ethylamine) **8** and 1,13-diamino-4,7,10-trioxatridecane **9** are commercially available. Amines **10 – 13:** were synthesized as described in literature.[9]

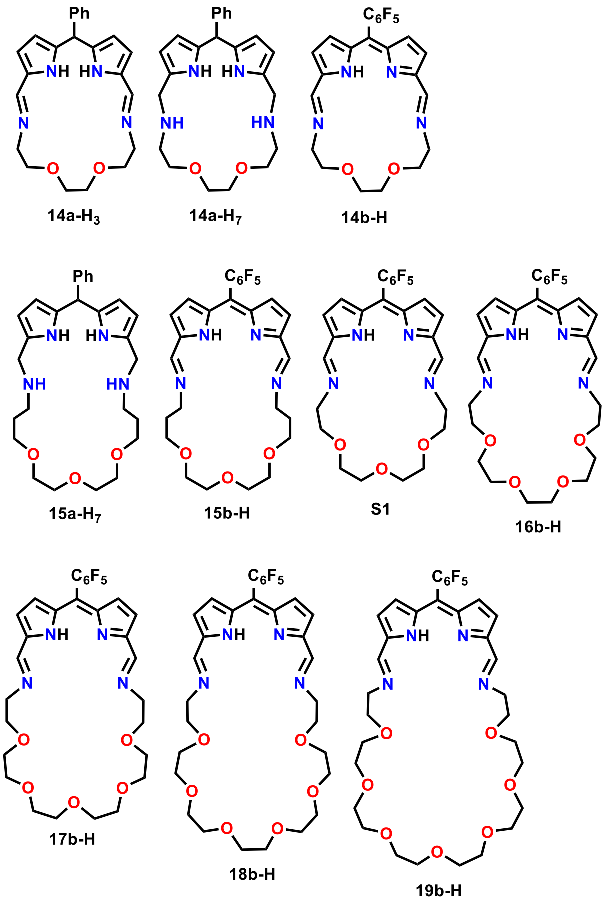

Scheme S1. The macrocycles which are discussed in the article.

General macrocyclization procedure. Synthesis of 14a-H$_3$, and **14b-H – 19b-H**:

In a 250 ml round-bottom flask equipped with a magnetic stirrer, **7a-H$_3$/7b-H$_3$** (0.55 mmol) was suspended in a solvent mixture of DCM:*n*-hexane 1:4 *v/v* (125 ml). Afterwards, **8 – 13** (0.55 mmol) was added to the reaction mixture, and the mixture was stirred at room temperature on air for 48 hours (96 hours for **14b-H**) sealed under a rubber septum. The mixture was then gravity filtered, and the obtained filtrate was evaporated to dryness under reduced pressure. The solid was washed with *n*-hexane (10 ml). After drying under reduced pressure, the products were isolated in a pure form. Obtained products were better preserved when kept in a cool dry place as a DCM solution not exceeding a 0.025 M concentration (allowed for long-term storage, and slowed down the degradation substantially).

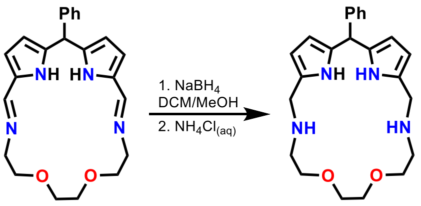

14a-H$_3$ 14a-H$_7$

Scheme S2. Reduction of **14a-H$_3$**.

Reduced crownphyrinogens 14a-H$_7$ and **15a-H$_7$**:

In a 50 ml round-bottom flask, crownphyrinogen **14a-H$_3$/15a-H$_3$** (0.06 mmol) was dissolved in a DCM:MeOH 7:1 solvent mixture (25 ml). Then, cerium(III) chloride heptahydrate ($CeCl_3 \cdot 7H_2O$;90 mg, 4 equiv.) was added under a flow of argon, followed by a subsequent addition of sodium borohydride ($NaBH_4$) in 30 mg portions every 2 minutes (227 mg, 6.0 mmol, 100 equiv.). Afterwards, the mixture was stirred for 18 hours at room temperature (298 K) under argon. The flask was opened on air, and a saturated solution of ammonium chloride (NH_4Cl) was introduced until evolution of gas ceased. The crude product was extracted with DCM three times (25 ml each), and the collected organic phase was washed with brine, water, and then dried over anhydrous Na_2SO_4. The filtrate was collected via gravity filtration, and the solvent was removed under reduced pressure. The obtained pale yellow oil was recrystallized from a DCM:*n*-hexane solvent mixture. Both products were isolated as off-white (beige) solids.

Lead(II) complexes 20a/b-Pb, 21b-Pb:

In a 25 ml round-bottom flask, to a 10 ml DCM solution of crownphyrinogen or crownphyrins **14a-H$_3$/14b-H/15b-H** (0.025 mmol) was added solid $Pb(OAc)_2$ (8.1 mg, 1.0 equiv.). The reaction mixture was then stirred at room temperature (298 K) for 24 hours sealed under a rubber septum. The solvent was removed under reduced pressure, which afforded stable dark-purple solid films. To obtain analytically pure samples of **20a/b-Pb**, the solid films were dissolved in a solvent mixture of DCM:*n*-hexane 1:4 *v/v* (50 ml), and the solution was divided evenly into 5 separate vials. Crystals of pure products were then grown by slow evaporation of the DCM:*n*-hexane mixture over the course of several days at room temperature. The dried crystals were mechanically isolated from the vials. This purification method lowered the yield substantially, although it allowed to obtain analytically pure complexes **20a/b-Pb**.

Zinc(II), cadmium(II), and mercury(II) complexes 21b-M:

In a 25 ml round-bottom flask, to a 10 ml DCM solution of crownphyrin **15b-H** (27.5 mg, 0.05 mmol) at -40 C°, was added a solution of $M(OAc)_2$ (1.0 equiv.) in methanol (0.5 ml) (M = Zn^{2+}, Cd^{2+}, Hg^{2+}), the conversion was visibly observed indicated by an instantaneous strong color change from orange to dark blue/purple. Complexes **21b-Zn**, **21b-Cd**, and **21b-Hg** underwent transformation above -40 C°.

Zinc(II), cadmium(II), and mercury(II) complexes 22a/b-M:

In a 50 ml round-bottom flask, to a 20 ml DCM solution of crownphyrinogen or crownphyrin **15a-H$_3$/15b-H** (0.05 mmol) was added solid $M(OAc)_2$ (0.5 equiv.) (M = Zn^{2+}, Cd^{2+}, Hg^{2+}). The reaction mixture was then stirred at room temperature (298 K) for 48 hours sealed under a rubber septum. The intensely purple colored solutions were then transferred to a 250 ml beaker, and diluted with *n*-hexane (40 ml). Following the dilution, 20 ml solutions were taken and then separated into separate vials. Crystals

of pure products were then grown by slow evaporation of the DCM:*n*-hexane mixture over the course of several days at room temperature. The dried crystals were mechanically isolated from the vials. Storage of the **22-M** complexes in a crystalline form, disallowed the formation of polymeric films, which tend to form upon evaporation of the solvent from the reaction mixture. This purification method lowered the yield substantially, although it allowed to obtain analytically pure complexes **22-M**. Dissolved **22-M** crystals in de-acidified polar organic solvents remained stable at r.t. under standard conditions.

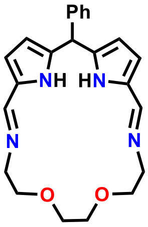

Crownphyrinogen 14a-H$_3$: off-white solid. Yield: 215 mg (97%) obtained from 153 mg (0.55 mmol) of **7a-H$_3$**, and 80 µl (0.55 mmol) of **8**.

^{1}H NMR (600 MHz, [D$_2$]dichloromethane, 300 K) δ (ppm): 8.01 (s, 2H, N=CH), 7.28 (t, 2H, ^3J = 7.6 Hz, *m*-Ph), 7.21 (t, 1H, ^3J = 7.4 Hz, *p*-Ph), 7.16 (d, 2H, ^3J = 7.6 Hz, *o*-Ph), 6.39 (d, 2H, ^3J = 3.5 Hz, $\beta_{1,4}$), 6.13 (d, 2H, ^3J = 3.5 Hz, $\beta_{2,3}$), 5.49 (s, 1H, *meso*-CH), 3.83 – 3.78 (m, 2H, oligo(ethylene glycol) chain), 3.74 – 3.67 (m, 4H, oligo(ethylene glycol) chain), 3.64 – 3.60 (m, 2H, oligo(ethylene glycol) chain), 3.57 – 3.53 (m, 2H, oligo(ethylene glycol) chain), 3.51 – 3.46 (m, 2H, oligo(ethylene glycol) chain).

^{13}C NMR (125 MHz, [D]chloroform, 300 K) δ (ppm): 152.8, 141.7, 136.1, 130.9, 128.7, 127.8, 126.7, 114.5, 109.0, 71.5, 70.8, 58.9, 44.1.

HRMS (ESI+, TOF) *m/z*: [M+H]$^+$ 391.2144, calcd. for $C_{23}H_{27}N_4O_2^+$ 391.2129.

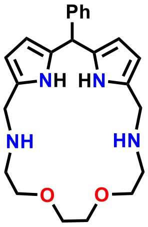

Crownphyrinogen 14a-H$_7$: beige solid. Yield: 10 mg (42%) obtained from 23 mg (0.06 mmol) of **14a-H$_3$**.

^{1}H NMR (600 MHz, [D]chloroform, 300 K) δ (ppm): 9.22 (b, 2H, NH), 7.26 – 7.22 (m, 2H, *m*-Ph), 7.19 – 7.14 (m, 3H, *o,p*-Ph), 5.90 (t, 2H, ^3J = 2.8 Hz $\beta_{1,4}$), 5.81 (t, 2H, ^3J = 7.4 Hz, $\beta_{2,3}$), 5.44 (s, 1H, *meso*-CH), 3.68 (s, 4H, C_α-CH$_2$), 3.61 – 3.51 (m, 8H, oligo(ethylene glycol) chain), 2.87 – 2.77 (m, 4H, oligo(ethylene glycol) chain).

^{13}C NMR (151 MHz, [D]chloroform, 300 K) δ (ppm): 143.2, 132.6, 129.7, 128.3, 128.1, 126.3, 106.5, 70.2, 49.0, 47.0, 44.0. *Not all of the signals in the ^{13}C NMR spectrum were assigned due to high broadening.*

HRMS (ESI+, TOF) *m/z*: [M+H]$^+$ 395.2452, calcd. for $C_{23}H_{31}N_4O_2^+$ 395.2442.

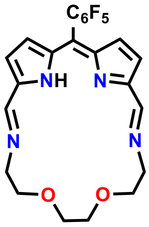

Crownphyrin 14b-H: dark orange solid. Yield: 252 mg (96%) obtained from 203 mg (0.55 mmol) of **7b-H$_3$**, and 80 µl (0.55 mmol) of **8**.

^{1}H NMR (600 MHz, [D]chloroform, 300 K) δ (ppm): 8.44 (s, 2H, N=CH), 6.69 (d, 2H, ^3J = 4.3 Hz, $\beta_{1,4}$), 6.48 (d, 2H, ^3J = 4.3 Hz, $\beta_{2,3}$), 3.93 – 3.88 (m, 4H, oligo(ethylene glycol) chain), 3.78 – 3.72 (m, 8H, oligo(ethylene glycol) chain).

^{13}C NMR (125 MHz, [D]chloroform, 300 K) δ (ppm): 155.8, 154.1, 144.7 (d, $^1J_{CF}$ = 250.1 Hz), 142.4, 141.7 (d, $^1J_{CF}$ = 255.2 Hz), 137.5 (d, $^1J_{CF}$ = 256.7 Hz), 127.3, 123.3, 119.4, 111.0, 71.2, 70.9, 59.3.

^{19}F NMR (471 MHz, [D]chloroform, 300 K) δ (ppm): -137.6 (dd, 2F, ^3J = 22.4 Hz, ^4J = 7.4 Hz, ArF *o*-F), -151.7 (t, 1F, ^3J = 20.9 Hz, ArF *p*-F), -160.5 (td, 2F, ^3J = 22.1 Hz, ^4J = 7.1 Hz, ArF *m*-F).

HRMS (ESI+, TOF) *m/z*: [M+H]$^+$ 479.1502, calcd. for $C_{23}H_{20}F_5N_4O_2^+$ 479.1501.

UV–vis (CH$_2$Cl$_2$, 298 K): λ_{max} (log (ε)) 273 (4.2), 325 (3.8), 483 (4.0).

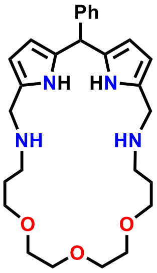

Crownphyrinogen 15a-H$_7$: beige solid. Yield: 4.5 mg (16%) obtained from 28 mg (0.06 mmol) of **15a-H$_3$**.

^{1}H NMR (600 MHz, [D]chloroform, 300 K) δ (ppm): 10.65 – 9.80 (b, 2H, NH), 7.50 – 7.10 (m, 5H, Ph), 6.10 (b, 2H, $\beta_{1,4}$), 5.85 (b, 2H, $\beta_{2,3}$), 5.40 (s, 1H, *meso*-CH), 4.18 – 4.00 (m, 4H, C_α-CH$_2$), 3.72 – 3.28 (m, 20H, oligo(ethylene glycol) chain), 3.16 – 3.00 (m, 4H, oligo(ethylene glycol) chain), 2.07 – 1.90 (m, 4H oligo(ethylene glycol) chain). **The integration for Ph signals could not be precisely determined due to broadening and overlap of residual solvents signals.*

^{13}C NMR (151 MHz, [D]chloroform, 300 K) δ (ppm): 142.2, 135.3, 128.3, 128.2, 126.6, 120.8, 111.1, 107.8, 69.7, 69.5, 69.1, 45.7, 44.3, 44.0, 25.0.

HRMS (ESI+, TOF) *m/z*: [M+H]$^+$ 467.3027, calcd. for $C_{27}H_{39}N_4O_3^+$ 467.3017.

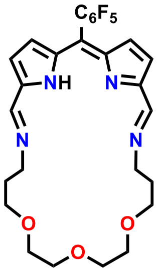

Crownphyrin 15b-H: dark orange solid. Yield: 278 mg (92%) obtained from 203 mg (0.55 mmol) of **7b-H$_3$**, and 120 µl (0.55 mmol) of **9**.

^{1}H NMR (600 MHz, [D]chloroform, 300 K) δ (ppm): 8.30 (s, 2H, N=CH), 6.75 (d, 2H, ^3J = 4.4 Hz, $\beta_{1,4}$), 6.46 (d, 2H, ^3J = 4.4 Hz, $\beta_{2,3}$), 3.78 (td, 4H, ^3J = 6.4 Hz, ^4J = 1.1 Hz oligo(ethylene glycol) chain), 3.72 – 3.69 (m, 4H, oligo(ethylene glycol) chain), 3.66 – 3.63 (m, 8H, oligo(ethylene glycol) chain), 1.98 (quint., 4H, ^3J = 6.2 Hz oligo(ethylene glycol) chain).

^{13}C NMR (151 MHz, [D]chloroform, 300 K) δ (ppm): 154.6, 154.3, 144.7 (d, $^1J_{CF}$ = 251.2 Hz), 142.4, 141.7 (d, $^1J_{CF}$ = 251.2 Hz), 137.6 (d, $^1J_{CF}$ = 251.2 Hz), 136.5, 127.5, 123.3, 120.0, 111.2, 70.7, 70.5, 68.3, 58.1, 30.8.

^{19}F NMR (471 MHz, [D]chloroform, 300 K) δ (ppm): -137.9 (dd, 2F, ^3J = 22.2 Hz, ^4J = 7.2 Hz, ArF *o*-F), -151.7 (t, 1F, ^3J = 20.6 Hz, ArF *p*-F), -160.5 (td, 2F, ^3J = 22.0 Hz, ^4J = 7.0 Hz, ArF *m*-F).

HRMS (ESI+, TOF) *m/z*: [M+H]$^+$ 551.2073, calcd. for $C_{27}H_{28}F_5N_4O_3^+$ 551.2076.

UV–vis (CH_2Cl_2, 298 K): λ_{max} (log (ε)) 273 (4.5), 483 (4.4).

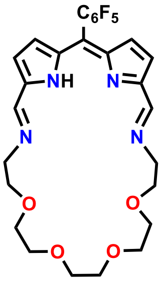

Crownphyrin 16b-H: dark orange solid. Yield: 224 mg (72%) obtained from 203 mg (0.55 mmol) of **7b-H$_3$**, and 130 mg (0.55 mmol) of **10**.

^{1}H NMR (600 MHz, [D]chloroform, 300 K) δ (ppm): 8.27 (s, 2H, N=CH), 6.77 (d, 2H, ^3J = 4.4 Hz, $\beta_{1,4}$), 6.46 (d, 2H, ^3J = 4.4 Hz, $\beta_{2,3}$), 3.88 – 3.82 (m, 8H, oligo(ethylene glycol) chain), 3.70 – 3.67 (m, 4H, oligo(ethylene glycol) chain), 3.66 – 3.64 (m, 8H, oligo(ethylene glycol) chain).

^{13}C NMR (125 MHz, [D]chloroform, 300 K) δ (ppm): 155.9, 154.3, 144.8 (d, $^1J_{CF}$ = 248.2 Hz), 142.5, 141.7 (d, $^1J_{CF}$ = 251.8 Hz), 137.5 (d, $^1J_{CF}$ = 253.5 Hz), 127.5, 123.3, 120.3, 111.2, 71.1, 70.9, 70.8, 70.5, 61.8.

^{19}F NMR (471 MHz, [D]chloroform, 300 K) δ (ppm): -137.9 (dd, 2F, ^3J = 22.2 Hz, ^4J = 6.8 Hz, ArF *o*-F), -151.8 (t, 1F, ^3J = 21.0 Hz, ArF *p*-F), -160.5 (td, 2F, ^3J = 21.4 Hz, ^4J = 6.5 Hz, ArF *m*-F).

HRMS (ESI+, TOF) *m/z*: [M+H]$^+$ 567.2015, calcd. for $C_{27}H_{28}F_5N_4O_4^+$ 567.2025.

UV–vis (CH_2Cl_2, 298 K): λ_{max} (log (ε)) 272 (4.2), 478 (4.2).

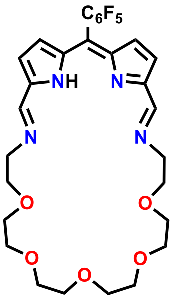

Crownphyrin 17b-H: dark orange solid. Yield: 205 mg (61%) obtained from 203 mg (0.55 mmol) of **7b-H$_3$**, and 154 mg (0.55 mmol) of **11**.

^{1}H NMR (500 MHz, [D]chloroform, 300 K) δ (ppm): 8.30 (s, 2H, N=CH), 6.78 (d, 2H, ^3J = 4.4 Hz, $\beta_{1,4}$), 6.45 (d, 2H, ^3J = 4.4 Hz, $\beta_{2,3}$), 3.90 – 3.78 (m, oligo(ethylene glycol) chain), 3.70 – 3.56 (m, oligo(ethylene glycol) chain), 3.55 – 3.50 (m, oligo(ethylene glycol) chain). **The integration for CH_2 resonances could not be precisely determined due to broadening and overlap with water and residual solvents signals.*

^{13}C NMR (125 MHz, [D]chloroform, 300 K) δ (ppm): 156.0, 154.3, 144.8 (d, $^1J_{CF}$ = 250.5 Hz), 142.4, 141.7 (d, $^1J_{CF}$ = 247.2 Hz), 138.5 (d, $^1J_{CF}$ = 257.4 Hz), 127.5, 123.3, 120.3, 111.2, 70.9, 70.8, 70.6, 70.5, 70.3, 61.7.

^{19}F NMR (471 MHz, [D]chloroform, 300 K) δ (ppm): -137.9 (dd, 2F, ^3J = 22.3 Hz, ^4J = 6.7 Hz, ArF *o*-F), -151.6 (t, 1F, ^3J = 20.7 Hz, ArF *p*-F), -160.4 (td, 2F, ^3J = 21.2 Hz, ^4J = 6.3 Hz, ArF *m*-F).

HRMS (ESI+, TOF) *m/z*: [M+H]$^+$ 611.2308, calcd. for $C_{29}H_{32}F_5N_4O_5^+$ 611.2287.

UV–vis (CH_2Cl_2, 298 K): λ_{max} (log (ε)) 273 (4.3), 479 (4.2).

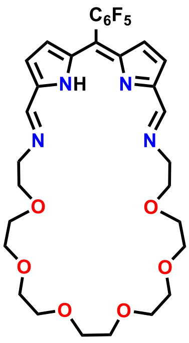

Crownphyrin 18b-H: dark orange solid. Yield: 237 mg (66%) obtained from 203 mg (0.55 mmol) of **7b-H$_3$**, and 178 mg (0.55 mmol) of **12**.

^{1}H NMR (600 MHz, [D]chloroform, 300 K) δ (ppm): 8.31 (s, 2H, N=CH), 6.78 (d, 2H, ^3J = 4.4 Hz, $\beta_{1,4}$), 6.45 (d, 2H, ^3J = 4.4 Hz, $\beta_{2,3}$), 3.89 – 3.85 (m, 4H, oligo(ethylene glycol) chain), 3.84 – 3.80 (m, 4H, oligo(ethylene glycol) chain), 3.67 – 3.59 (m, 12H, oligo(ethylene glycol) chain), 3.59 – 3.53 (m, 8H, oligo(ethylene glycol) chain).

^{13}C NMR (125 MHz, [D]chloroform, 300 K) δ (ppm): 155.9, 154.3, 144.8 (d, $^1J_{CF}$ = 252.4 Hz), 142.4, 141.8 (d, $^1J_{CF}$ = 252.7 Hz), 137.5 (d, $^1J_{CF}$ = 250.3 Hz), 127.5, 123.3, 120.3, 111.2, 70.8, 70.7, 70.6, 70.5, 70.5, 70.4, 61.6.

^{19}F NMR (471 MHz, [D]chloroform, 300 K) δ (ppm): -137.9 (d, 2F, ^{3}J = 20.2 Hz, ArF *o*-F), -151.6 (t, 1F, ^{3}J = 21.0 Hz, ArF *p*-F), -160.4 (t, 2F, ^{3}J = 20.2 Hz, ArF *m*-F).

HRMS (ESI+, TOF) *m/z*: $[M+H]^+$ 655.2565, calcd. for $C_{31}H_{36}F_5N_4O_6^+$ 655.2550.

UV–vis (CH_2Cl_2, 298 K): λ_{max} (log (ε)) 272 (4.4), 480 (4.3).

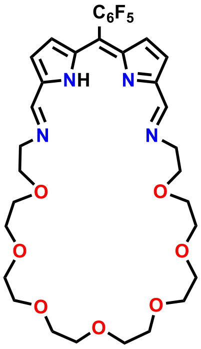

Crownphyrin 19b-H: dark orange solid. Yield: 288 mg (75%) obtained from 203 mg (0.55 mmol) of **7b-H$_3$**, and 203 mg (0.55 mmol) of **13**.

^{1}H NMR (600 MHz, [D]chloroform, 300 K) δ (ppm): 8.31 (s, 2H, N=CH), 6.78 (d, 2H, ^{3}J = 4.4 Hz, $\beta_{1,4}$), 6.45 (d, 2H, ^{3}J = 4.4 Hz, $\beta_{2,3}$), 3.90 – 3.86 (m, 4H, oligo(ethylene glycol) chain), 3.84 – 3.80 (m, 4H, oligo(ethylene glycol) chain), 3.68 – 3.56 (m, oligo(ethylene glycol) chain). **The integration for CH$_2$ resonances could not be precisely determined due to broadening and overlap with water and residual solvents signals.*

^{13}C NMR (125 MHz, [D]chloroform, 300 K) δ (ppm): 155.9, 154.3, 144.7 (d, $^{1}J_{CF}$ = 250.1 Hz), 142.4, 141.7 (d, $^{1}J_{CF}$ = 252.0 Hz), 137.5 (d, $^{1}J_{CF}$ = 255.0 Hz), 127.5, 123.3, 120.4, 111.2, 70.7, 70.7, 70.6, 70.6, 70.6, 70.6, 70.5, 61.5.

^{19}F NMR (471 MHz, [D]chloroform, 300 K) δ (ppm): -137.9 (dd, 2F, ^{3}J = 22.2 Hz, ^{4}J = 6.7 Hz, ArF *o*-F), -151.6 (t, 1F, ^{3}J = 20.8 Hz, ArF *p*-F), -160.4 (dd, 2F, ^{3}J = 21.5 Hz, ^{4}J = 6.2 Hz, ArF *m*-F).

HRMS (ESI+, TOF) *m/z*: $[M+H]^+$ 699.2822, calcd. for $C_{33}H_{40}F_5N_4O_7^+$ 699.2812.

UV–vis (CH_2Cl_2, 298 K): λ_{max} (log (ε)) 272 (4.3), 484 (4.3).

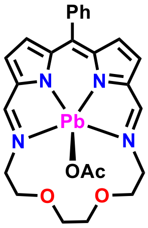

Lead(II) complex 20a-Pb: dark purple crystals. Yield: 8.5 mg (52%) obtained from 9.8 mg (0.025 mmol) of **14a-H$_3$**, and 8.1 mg (0.025 mmol, 1.0 equiv.) of $Pb(OAc)_2$.

^{1}H NMR (600 MHz, [D]chloroform, 300 K) δ (ppm): 8.60 (s, 2H, N=CH), 7.48 – 7.41 (m, 3H, Ph), 7.40 – 7.36 (m, 2H, Ph), 6.67 (d, 2H, ^{3}J = 4.2 Hz, $\beta_{1,4}$), 6.59 (d, 2H, ^{3}J = 4.2 Hz, $\beta_{2,3}$), 4.07 – 3.87 (m, 4H, oligo(ethylene glycol) chain), 3.86 – 3.57 (m, 8H, oligo(ethylene glycol) chain), 1.82 (s, 3H, OAc).

^{13}C NMR (125 MHz, [D]chloroform, 300 K) δ (ppm): 178.0, 157.6, 156.3, 152.2, 144.9, 139.8, 133.9, 131.0, 128.4, 127.0, 120.9, 70.8, 70.2, 58.8, 25.6.

HRMS (ESI+, TOF) *m/z*: $[M]^+$ 595.1593, calcd. for $C_{23}H_{23}N_4O_2Pb^+$ 595.1585.

UV–vis (CH_2Cl_2, 298 K): λ_{max} (log (ε)) 293 (4.4), 318 (4.3), 544 (4.2), 578 (4.7).

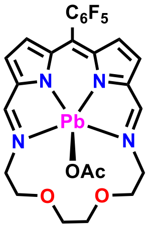

Lead(II) complex 20b-Pb: dark purple crystals. Yield: 10.6 mg (57%) obtained from 11.7 mg (0.025 mmol) of **14b-H**, and 8.1 mg (0.025 mmol, 1.0 equiv.) of $Pb(OAc)_2$.

^{1}H NMR (600 MHz, [D]chloroform, 210 K) δ (ppm): 8.69 (s, 2H, N=CH), 6.78 (d, 2H, ^{3}J = 4.2 Hz, $\beta_{1,4}$), 6.59 (d, 2H, ^{3}J = 4.2 Hz, $\beta_{2,3}$), 4.38 (t, 2H, ^{3}J = 10.9 Hz, oligo(ethylene glycol) chain), 4.03 – 3.97 (m, 2H, oligo(ethylene glycol) chain), 3.96 – 3.91 (m, 2H, oligo(ethylene glycol) chain), 3.69 – 3.59 (m, 4H, oligo(ethylene glycol) chain), 3.44 (t, 2H, ^{3}J = 9.5 Hz, oligo(ethylene glycol) chain), 1.87 (s, 3H, OAc).

^{13}C NMR (125 MHz, [D]chloroform, 300 K) δ (ppm): 177.3, 157.8, 157.3, 144.8 (d, $^{1}J_{CF}$ = 250.1 Hz), 143.7, 141.3 (d, $^{1}J_{CF}$ = 251.5 Hz), 137.1 (d, $^{1}J_{CF}$ = 251.5 Hz), 133.2, 131.7, 122.1, 114.1, 70.8, 70.3, 58.9, 24.9.

^{19}F NMR (471 MHz, [D]chloroform, 300 K) δ (ppm): -137.3 (b, 1F, ArF *o*-F), -138.9 (b, 1F, ArF *o*-F), -153.1 (t, 1F, ^{3}J = 20.7 Hz, ArF *p*-F), -161.2 (b, 1F, ArF *m*-F), -161.6 (b, 1F, ArF *m*-F).

HRMS (ESI+, TOF) *m/z*: $[M]^+$ 685.1087, calcd. for $C_{23}H_{18}F_5N_4O_2Pb^+$ 685.1110.

UV–vis (CH_2Cl_2, 298 K): λ_{max} (log (ε)) 299 (4.4), 552 (4.3), 589 (4.7).

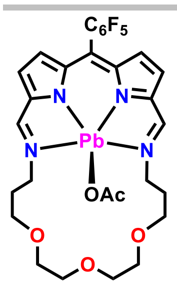

Lead(II) complex 21b-Pb: dark purple solid. Yield: 19 mg (93%) obtained from 13.8 mg (0.025 mmol) of **15b-H**, and 8.1 mg (0.025 mmol, 1.0 equiv.) of $Pb(OAc)_2$.

^{1}H NMR (600 MHz, [D]chloroform, 300 K) δ (ppm): 8.60 (s, 2H, N=CH), 6.72 (d, 2H, ^3J = 4.2 Hz, $\beta_{1,4}$), 6.57 (d, 2H, ^3J = 4.2 Hz, $\beta_{2,3}$), 4.36 – 4.23 (m, 2H, oligo(ethylene glycol) chain), 3.71 – 3.32 (m, oligo(ethylene glycol) chain), 2.44 – 2.32 (m, 2H, oligo(ethylene glycol) chain), 2.00 – 1.92 (m, 2H, oligo(ethylene glycol) chain), 1.89 (s, 3H, OAc). **The integration for CH_2 resonances could not be precisely determined due to broadening and overlap with water and residual solvents signals.*

^{13}C NMR (125 MHz, [D]chloroform, 300 K) δ (ppm): 178.1, 157.8, 156.0, 144.8 (d, $^1J_{CF}$ = 248.1 Hz), 143.3, 141.4 (d, $^1J_{CF}$ = 256.4 Hz), 137.2 (d, $^1J_{CF}$ = 250.0 Hz), 132.9, 131.8, 122.0, 113.7, 70.2, 69.8, 69.6, 59.1, 31.2, 29.7.

^{19}F NMR (471 MHz, [D]chloroform, 300 K) δ (ppm): -137.3 (b, 1F, ArF *o*-F), -139.1 (b, 1F, ArF *o*-F), -152.7 (t, 1F, ^3J = 21.5 Hz, ArF *p*-F), -160.9 (b, 1F, ArF *m*-F), -161.4 (b, 1F, ArF *m*-F).

HRMS (ESI+, TOF) *m/z*: $[M]^+$ 757.1690, calcd. for $C_{27}H_{26}F_5N_4O_3Pb^+$ 757.1686.

UV–vis (CH_2Cl_2, 298 K): λ_{max} (log (ε)) 295 (4.4), 551 (4.2), 585 (4.6).

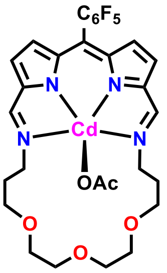

Cadmium(II) complex 21b-Cd: dark blue/purple solution. Yield: quantitative conversion from 27.5 mg (0.05 mmol) of **15b-H**, and 11.5 mg (0.05 mmol, 1.0 equiv.) of $Cd(OAc)_2$ in methanol (0.5 ml).

^{1}H NMR (600 MHz, [D]chloroform, 300 K) δ (ppm): 8.14 (t, 2H, ^3J = 13.4 Hz, N=CH), 6.52 (b, 4H, β_{1-4}), 3.80 – 3.65 (m, 4H, oligo(ethylene glycol) chain), 3.60 – 3.45 (m, oligo(ethylene glycol) chain), 3.40 – 3.29 (m, 2H, oligo(ethylene glycol) chain), 2.20 – 2.13 (m, 4H, oligo(ethylene glycol) chain), 1.95 (s, 3H, OAc). **The integration for CH_2 resonances could not be precisely determined due to broadening and overlap with water and residual solvents signals.*

^{13}C NMR (125 MHz, [D]chloroform, 300 K) δ (ppm): 178.1, 157.1, 155.8, 142.1, 131.0, 119.6, 70.1, 68.3, 57.1, 29.4, 22.0. *Not all of the signals in the ^{13}C NMR spectrum were assigned due to broadening.*

^{13}C NMR (151 MHz, [D_2]dichloromethane, 300 K) δ (ppm): 178.7, 155.7, 155.1, 144.9 (d, $^1J_{CF}$ = 248.1 Hz), 144.3, 141.6 (d, $^1J_{CF}$ = 254.0 Hz), 137.6 (d, $^1J_{CF}$ = 250.6 Hz), 131.7, 119.8, 113.2, 70.1, 69.6, 69.5, 58.0, 30.3, 21.3.

^{19}F NMR (471 MHz, [D_2]dichloromethane, 178 K) δ (ppm): -140.5 (b, 1F, ArF *o*-F), -140.9 (b, 1F, ArF *o*-F), -153.8 (b, 1F, ArF *p*-F), -162.2 (b, 2F, ArF *m*-F).

HRMS (ESI+, TOF) *m/z*: $[M]^+$ 663.0913, calcd. for $C_{27}H_{26}F_5N_4O_3Cd^+$ 663.0953.

UV–vis (CH_2Cl_2, 298 K): λ_{max} (log (ε)) 295 (4.5), 557 (4.4), 585 (4.6).

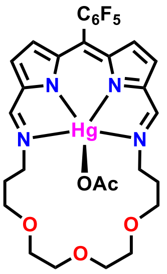

Mercury(II) complex 21b-Hg: dark blue/purple solution. Yield: quantitative conversion from 27.5 mg (0.05 mmol) of **15b-H**, and 16.0 mg (0.05 mmol, 1.0 equiv.) of $Hg(OAc)_2$ in methanol (0.5 ml).

^{1}H NMR (600 MHz, [D_2]dichloromethane, 220 K) δ (ppm): 8.43 (s, 2H, N=CH), 6.72 (d, 2H, ^3J = 4.2 Hz, $\beta_{1,4}$), 6.62 (d, 2H, ^3J = 4.2 Hz, $\beta_{2,3}$), 3.89 – 3.79 (m, 4H, oligo(ethylene glycol) chain), 3.60 – 3.55 (m, 8H, oligo(ethylene glycol) chain), 3.55 – 3.51 (m, 4H, oligo(ethylene glycol) chain), 2.06 (quint., 4H, ^3J = 6.9 Hz oligo(ethylene glycol) chain)**,** 1.94 (s, OAc; overlapping with the signal of $Hg(OAc)_2$).

^{13}C NMR (125 MHz, [D_2]dichloromethane, 300 K) δ (ppm): 175.5, 155.7, 153.2, 144.7, 144.1 (d, $^1J_{CF}$ = 251.3 Hz), 141.2 (d, $^1J_{CF}$ = 253.4 Hz), 136.9 (d, $^1J_{CF}$ = 249.8 Hz), 132.3, 131.6, 119.7, 111.6, 69.8, 69.4, 67.7, 56.3, 30.1, 21.3.

^{19}F NMR (471 MHz, [D_2]dichloromethane, 178 K) δ (ppm): -138.6 (b, 2F, ArF *o*-F), -151.7 (t, 1F, ^3J = 21.0 Hz, ArF *p*-F), -160.6 (b, 2F, ArF *m*-F).

HRMS (ESI+, TOF) *m/z*: $[M]^+$ 751.1630, calcd. for $C_{27}H_{26}F_5N_4O_3Hg^+$ 751.1631.

UV–vis (CH_2Cl_2, 298 K): λ_{max} (log (ε)) 296 (4.4), 550 (4.3), 600 (4.2).

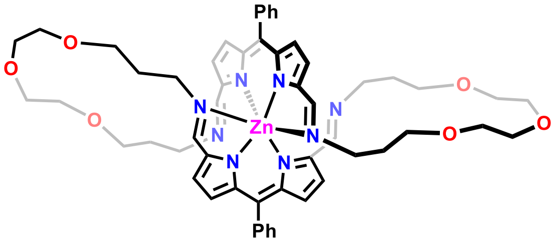

Zinc(II) complex 22a-Zn: dark purple crystals. Yield: 2.5 mg (11%) obtained from 23.1 mg (0.05 mmol) of **15a-H$_3$** and 4.6 mg (0.025 mmol, 0.5 equiv.) of Zn(OAc)$_2$.

^{1}H NMR (600 MHz, [D$_2$]dichloromethane, 300 K) δ (ppm): 7.99 (s, 4H, N=CH), 7.56 – 7.47 (m, 10H, Ph), 6.72 (d, 4H, ^3J = 4.2 Hz, $\beta_{1,4}$), 6.67 (d, 4H, ^3J = 4.2 Hz, $\beta_{2,3}$), 3.48 – 3.42 (m, 4H, oligo(ethylene glycol) chain), 3.40 – 3.35 (m, 4H, oligo(ethylene glycol) chain), 3.35 – 3.30 (m, 4H, oligo(ethylene glycol) chain), 3.28 – 3.24 (m, 4H, oligo(ethylene glycol) chain), 3.21 – 3.16 (m, 4H, oligo(ethylene glycol) chain), 3.08 – 3.03 (m, 12H, oligo(ethylene glycol) chain), 1.72 – 1.64 (m, 4H, oligo(ethylene glycol) chain), 1.52 – 1.47 (m, 4H, oligo(ethylene glycol) chain).

^{13}C NMR (125 MHz, [D$_2$]dichloromethane, 300 K) δ (ppm): 156.2, 156.0, 147.9, 143.0, 139.3, 132.9, 130.7, 128.7, 127.5, 118.0, 71.2, 70.2, 68.3 57.5, 29.7.

HRMS (ESI+, TOF) *m/z*: [M+H]$^+$ 983.4131, calcd. for $C_{54}H_{63}N_8O_6Zn^+$ 983.4157, [M+2H]$^{2+}$ 492.2126, calcd. for $C_{54}H_{64}N_8O_6Zn^{2+}$ 492.2115.

UV–vis (CH$_2$Cl$_2$, 298 K): λ_{max} (log (ε)) 298 (4.4), 309 (3.8), 356 (3.8), 513 (4.5), 545 (4.8).

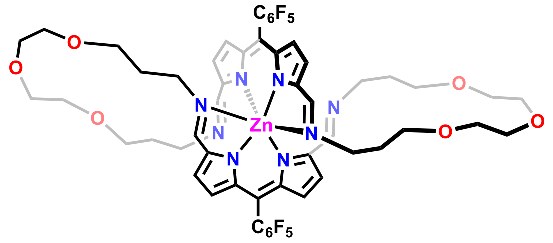

Zinc(II) complex 22b-Zn:

^{1}H NMR (600 MHz, [D]chloroform, 300 K) δ (ppm): 7.91 (s, 4H, N=CH), 6.77 (d, 4H, ^3J = 4.3 Hz, $\beta_{1,4}$), 6.61 (d, 4H, ^3J = 4.3 Hz, $\beta_{2,3}$), 3.49 – 3.43 (m, oligo(ethylene glycol) chain), 3.43 – 3.39 (m, oligo(ethylene glycol) chain), 3.39 – 3.35 (m, oligo(ethylene glycol) chain), 3.35 – 3.31 (m, 4H, oligo(ethylene glycol) chain), 3.26 – 3.21 (m, 4H, oligo(ethylene glycol) chain), 3.14 – 3.03 (m, 12H, oligo(ethylene glycol) chain), 1.75 – 1.67 (m, 4H, oligo(ethylene glycol) chain), 1.47 – 1.40 (m, 4H, oligo(ethylene glycol) chain). **The integration for CH$_2$ resonances could not be precisely determined due to broadening and overlap with water and residual solvents signals.*

HRMS (ESI+, TOF) *m/z*: [M+H]$^+$ 1163.3232, calcd. for $C_{54}H_{53}F_{10}N_8O_6Zn^+$ 1163.3214, [M+2H]$^{2+}$ 582.1650, calcd. for $C_{54}H_{54}F_{10}N_8O_6Zn^{2+}$ 582.1644.

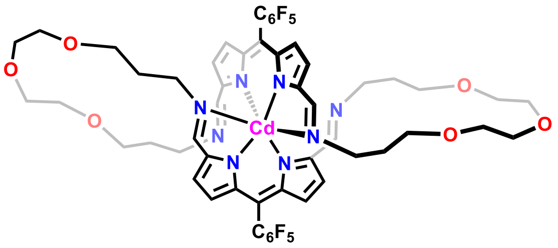

Cadmium(II) complex 22b-Cd: dark purple crystals. Yield: 11.5 mg (19%) obtained from 27.5 mg (0.05 mmol) of **15b-H** and 5.8 mg (0.025 mmol, 0.5 equiv.) of Cd(OAc)$_2$.

^{1}H NMR (600 MHz, [D]chloroform, 300 K) δ (ppm): 8.17 (s, 4H, N=CH), 6.78 (d, 4H, ^3J = 4.2 Hz, $\beta_{1,4}$), 6.59 (d, 4H, ^3J = 4.2 Hz, $\beta_{2,3}$), 3.60 – 3.54 (m, 4H, oligo(ethylene glycol) chain), 3.49 – 3.44 (m, 4H, oligo(ethylene glycol) chain), 3.44 – 3.38 (m, 8H, oligo(ethylene glycol) chain), 3.25 – 3.20 (m, 4H, oligo(ethylene glycol) chain), 3.10 – 3.02 (m, 8H, oligo(ethylene glycol) chain), 3.02 – 2.97 (m, 4H, oligo(ethylene glycol) chain), 1.77 – 1.69 (m, 4H, oligo(ethylene glycol) chain), 1.62 – 1.55 (m, 4H, oligo(ethylene glycol) chain).

^{13}C NMR (125 MHz, [D$_2$]dichloromethane, 300 K) δ (ppm): 157.6, 156.8, 144.8 (d, $^1J_{CF}$ = 249.2 Hz), 143.5, 141.4 (d, $^1J_{CF}$ = 249.5 Hz), 137.3 (d, $^1J_{CF}$ = 252.0 Hz), 131.6, 129.9, 119.7, 113.8, 71.0, 70.3, 68.0, 57.3, 29.3.

^{19}F NMR (471 MHz, [D$_2$]dichloromethane, 300 K) δ (ppm): -139.7 (d, 2F, ^3J = 22.3 Hz, ArF *o*-F), -153.9 (t, 1F, ^3J = 19.7 Hz, ArF *p*-F), -153.9 (t, 2F, ^3J = 22.1 Hz, ArF *m*-F).

^{19}F NMR (471 MHz, [D$_2$]dichloromethane, 180 K) δ (ppm): -140.4 (b, 1F, ArF *o*-F), -141.0 (b, 1F, ArF *o*-F), -153.9 (b, 1F, ArF *p*-F), -162.0 (b, 1F, ArF *m*-F), -162.3 (b, 1F, ArF *m*-F).

HRMS (ESI+, TOF) *m/z*: [M+H]$^+$ 1213.2975, calcd. for $C_{54}H_{53}F_{10}N_8O_6Cd^+$ 1213.2956, [M+2H]$^{2+}$ 607.1525, calcd. for $C_{54}H_{54}F_{10}N_8O_6Cd^{2+}$ 607.1515.

UV–vis (CH$_2$Cl$_2$, 298 K): λ_{max} (log (ε)) 285 (4.6), 524 (2.7), 557 (4.9).

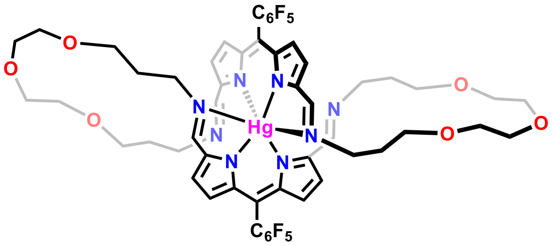

Mercury(II) complex 22b-Hg: dark purple crystals. Yield: 5.2 mg (8%) obtained from 27.5 mg (0.05 mmol) of **15b-H** and 8.0 mg (0.025 mmol, 0.5 equiv.) of $Hg(OAc)_2$.

^{1}H NMR (600 MHz, [D_2]dichloromethane, 300 K) δ (ppm): 8.15 (s, 4H, N=CH), 6.84 (d, 4H, ^3J = 4.3 Hz, $β_{1,4}$), 6.61 (b, 4H, $β_{2,3}$), 3.70 – 3.66 (m, 4H, oligo(ethylene glycol) chain), 3.64 – 3.58 (m, 4H, oligo(ethylene glycol) chain), 3.44 – 3.39 (m, oligo(ethylene glycol) chain), 3.38 – 3.35 (m, oligo(ethylene glycol) chain), 3.20 – 3.12 (m, 8H, oligo(ethylene glycol) chain), 3.11 – 3.01 (m, 8H, oligo(ethylene glycol) chain), 1.78 – 1.70 (m, 4H, oligo(ethylene glycol) chain), 1.63 – 1.57 (m, 4H, oligo(ethylene glycol) chain). **The integration for CH_2 resonances could not be precisely determined due to broadening and overlap with water and residual solvents signals.*

^{13}C NMR (125 MHz, [D_2]dichloromethane, 300 K) δ (ppm): 157.3, 155.4, 144.8 (d, $^1J_{CF}$ = 250.3 Hz), 144.0, 141.6 (d, $^1J_{CF}$ = 252.2 Hz), 137.5 (d, $^1J_{CF}$ = 251.5 Hz), 131.5, 128.8, 120.0, 114.1, 70.9, 70.3, 67.9, 57.5, 29.6.

^{19}F NMR (471 MHz, [D_2]dichloromethane, 300 K) δ (ppm): -139.8 (d, 2F, ^3J = 22.0 Hz, ArF *o*-F), -153.7 (t, 1F, ^3J = 20.5 Hz, ArF *p*-F), -161.8 (td, 2F, ^3J = 21.6 Hz, ^4J = 7.4 Hz, ArF *m*-F).

HRMS (ESI+, TOF) *m/z*: $[M+H]^+$ 1301.3643, calcd. for $C_{54}H_{53}F_{10}N_8O_6Hg^+$ 1301.3629, $[M+2H]^{2+}$ 651.1892, calcd. for $C_{54}H_{54}F_{10}N_8O_6Hg^{2+}$ 651.1851.

UV–vis (CH_2Cl_2, 298 K): $λ_{max}$ (log (ε)) 282 (4.7), 517 (4.7), 548 (4.8).

High-resolution mass spectra

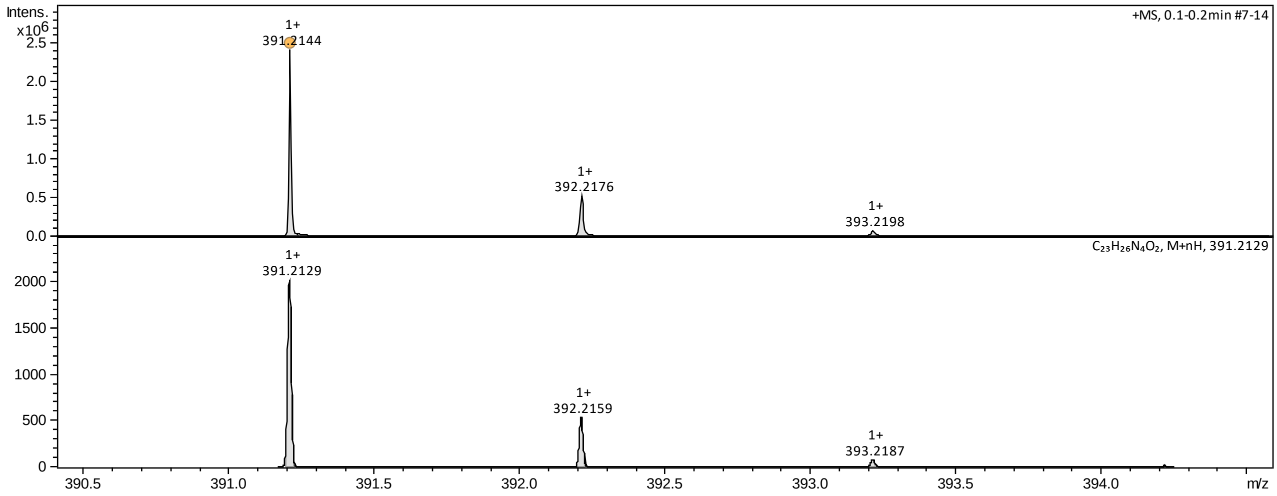

Figure S 1. The high-resolution mass spectrum of **14a-H$_3$** (ESI, TOF, $[M+H]^+$). Top: experimental spectrum, bottom: simulated pattern.

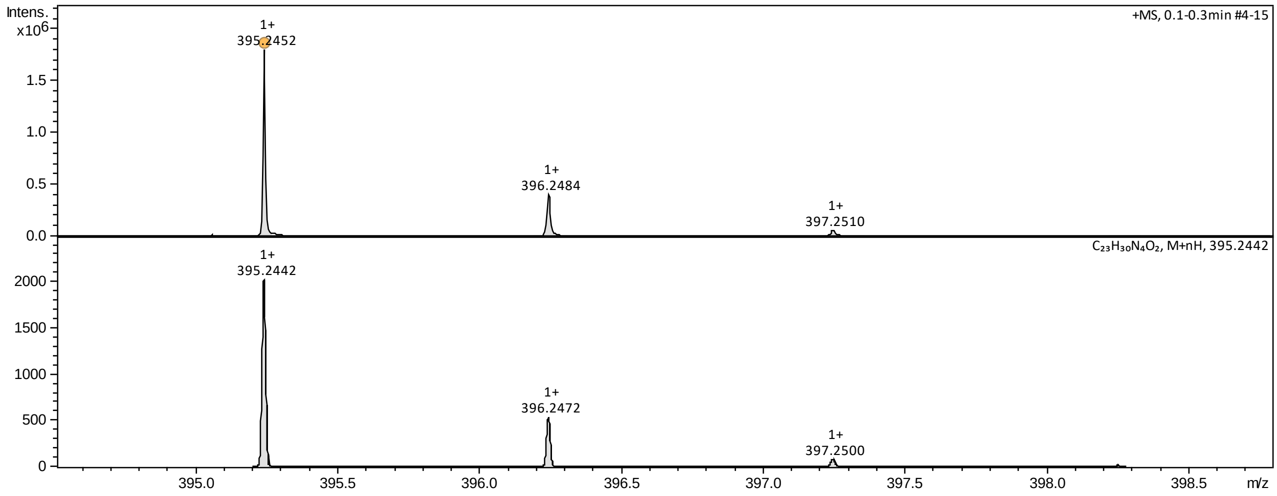

Figure S 2. The high-resolution mass spectrum of **14a-H$_7$** (ESI, TOF, $[M+H]^+$). Top: experimental spectrum, bottom: simulated pattern.

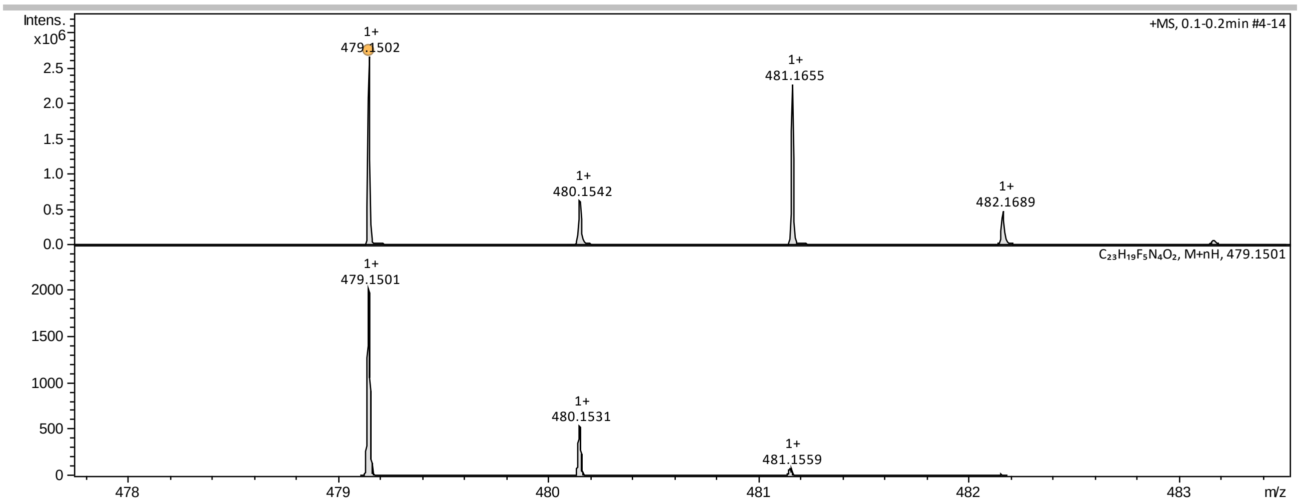

Figure S 3. The high-resolution mass spectrum of **14b-H** (ESI, TOF, $[M+H]^+$). Top: experimental spectrum, bottom: simulated pattern.

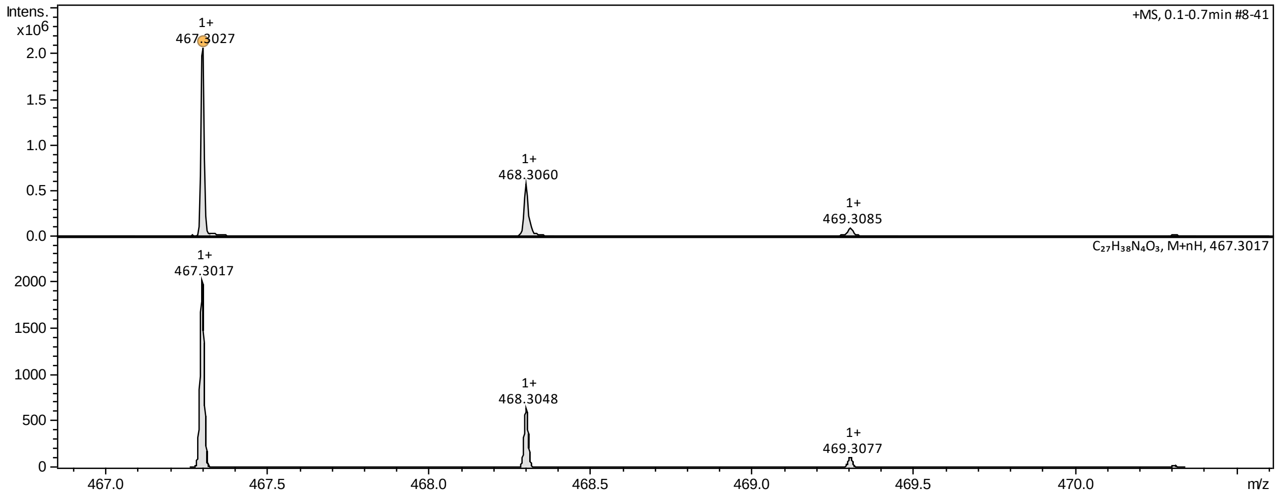

Figure S 4. The high-resolution mass spectrum of **15a-H$_7$** (ESI, TOF, $[M+H]^+$). Top: experimental spectrum, bottom: simulated pattern.

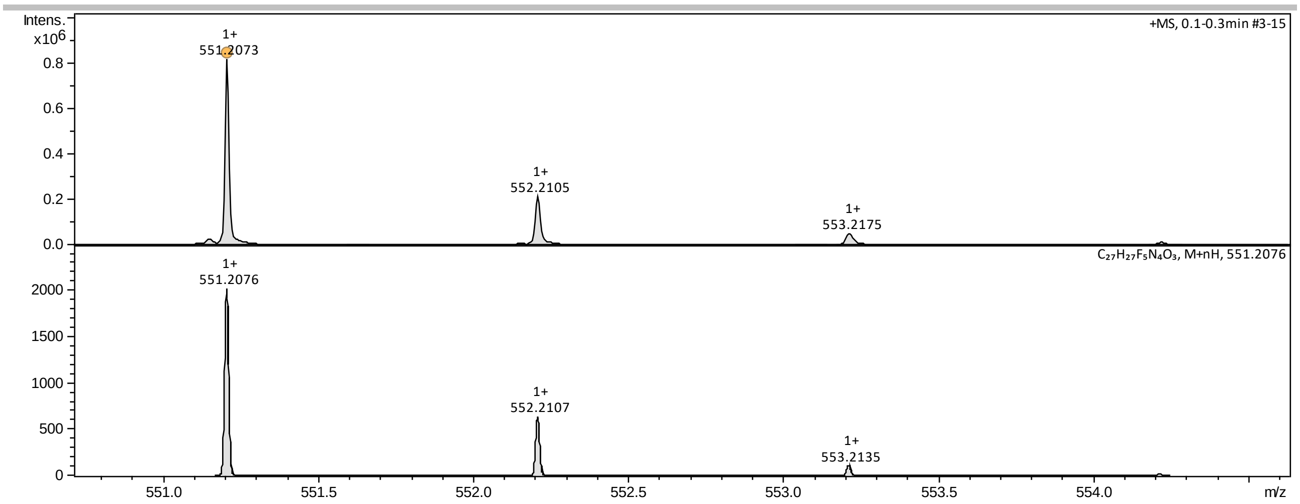

Figure S 5. The high-resolution mass spectrum of **15b-H** (ESI, TOF, $[M+H]^+$). Top: experimental spectrum, bottom: simulated pattern.

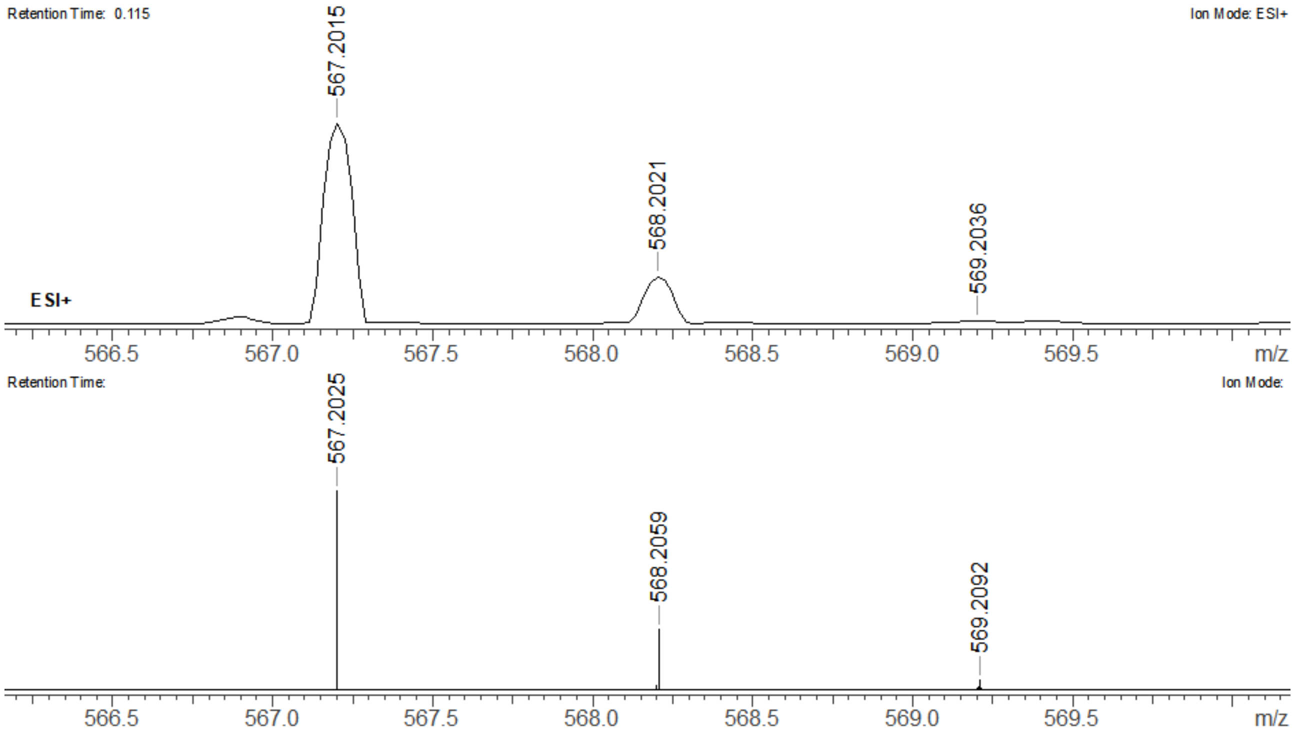

Figure S 6. The high-resolution mass spectrum of **16b-H** (ESI, TOF, $[M+H]^+$). Top: experimental spectrum, bottom: simulated pattern.

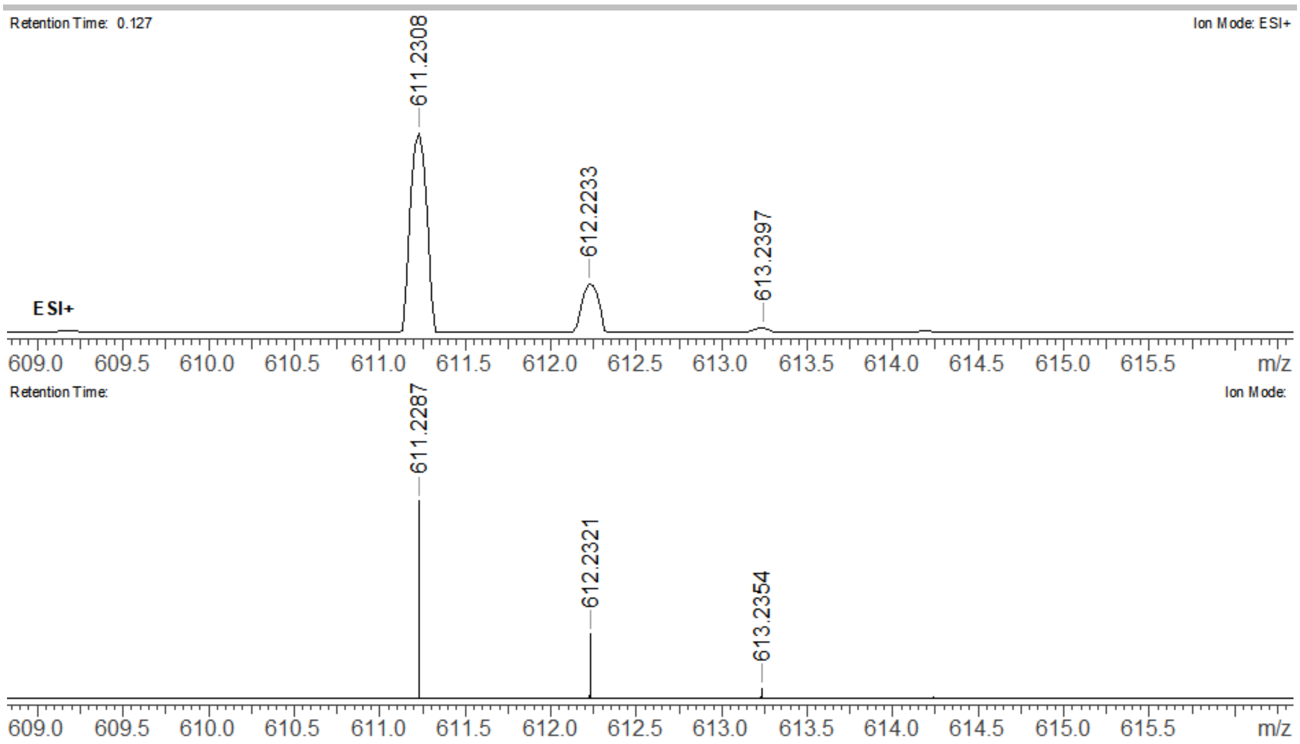

Figure S 7. The high-resolution mass spectrum of **17b-H** (ESI, TOF, $[M+H]^+$). Top: experimental spectrum, bottom: simulated pattern.

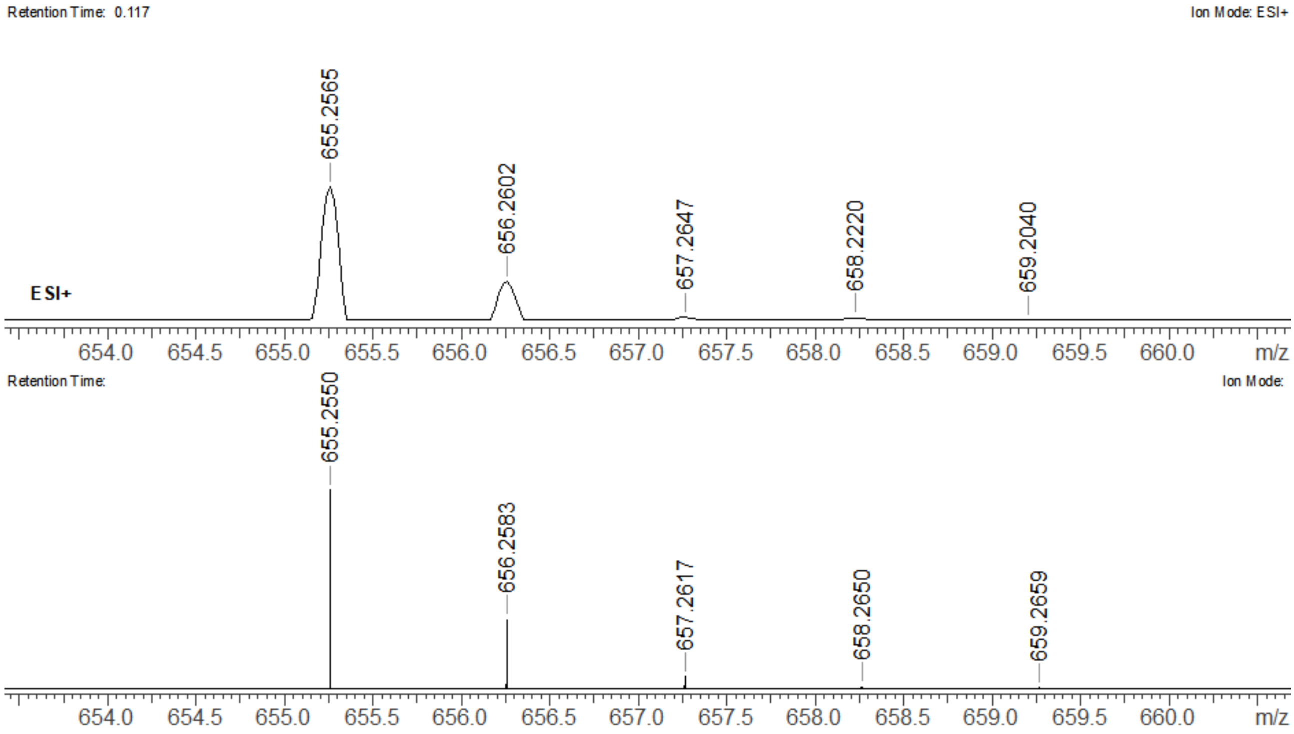

Figure S 8. The high-resolution mass spectrum of **18b-H** (ESI, TOF, $[M+H]^+$). Top: experimental spectrum, bottom: simulated pattern.

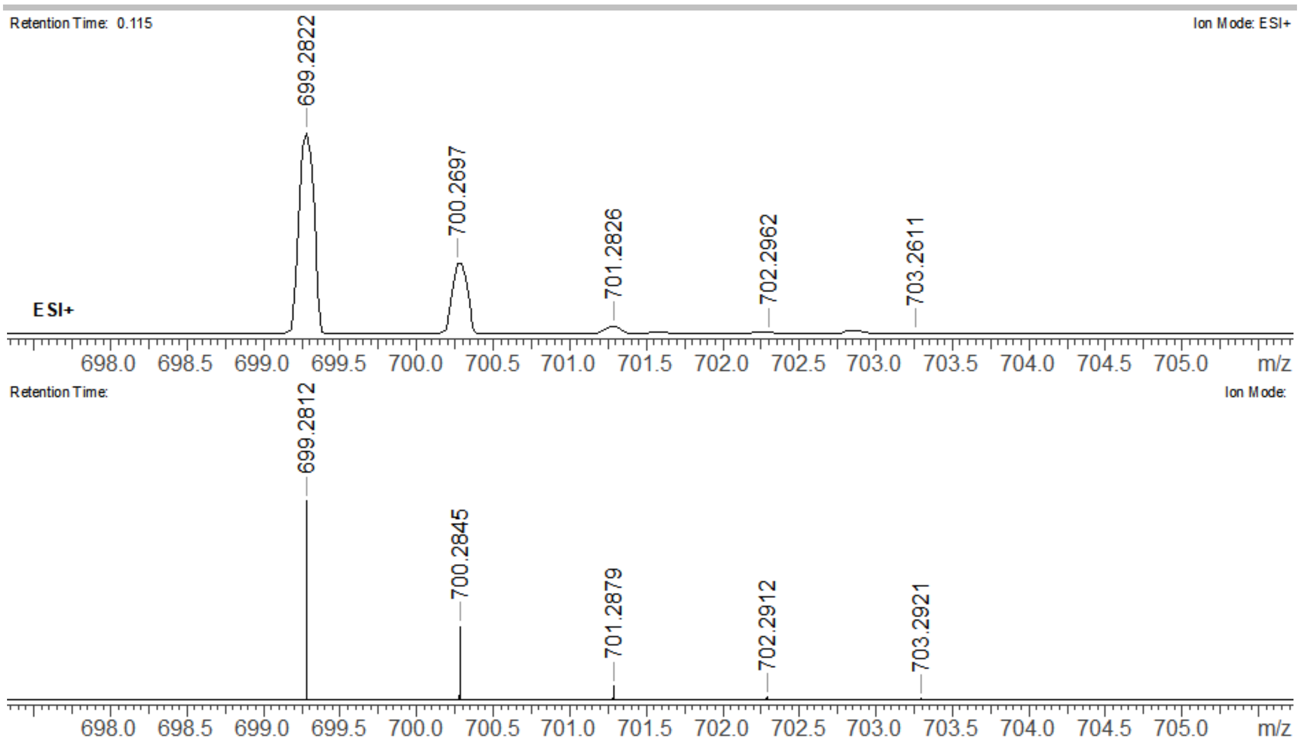

Figure S 9. The high-resolution mass spectrum of **19b-H** (ESI, TOF, $[M+H]^+$). Top: experimental spectrum, bottom: simulated pattern.

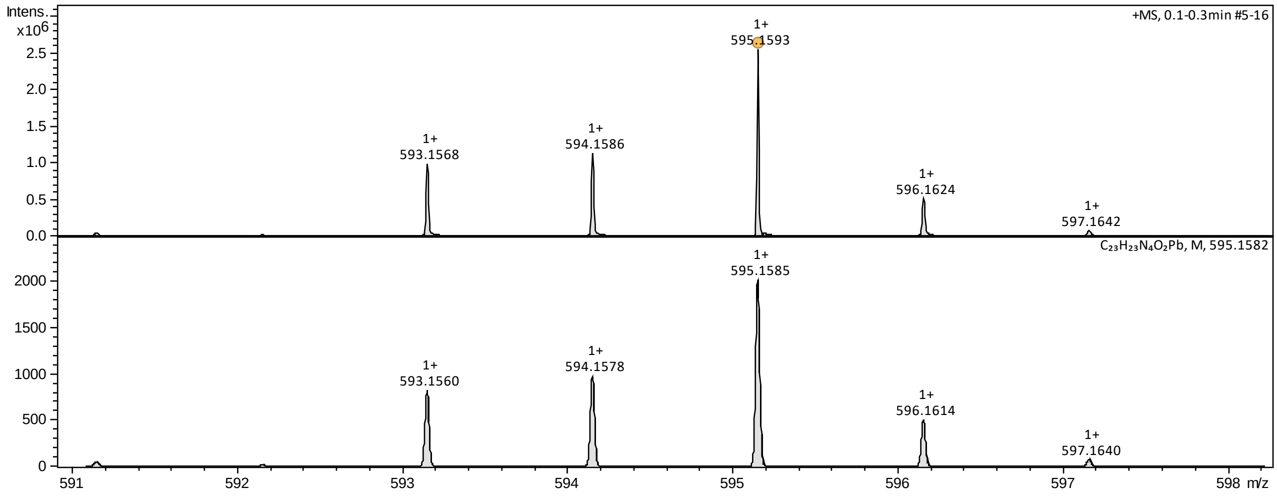

Figure S 10. The high-resolution mass spectrum of **20a-Pb** (ESI, TOF, $[M+H]^+$). Top: experimental spectrum, bottom: simulated pattern.

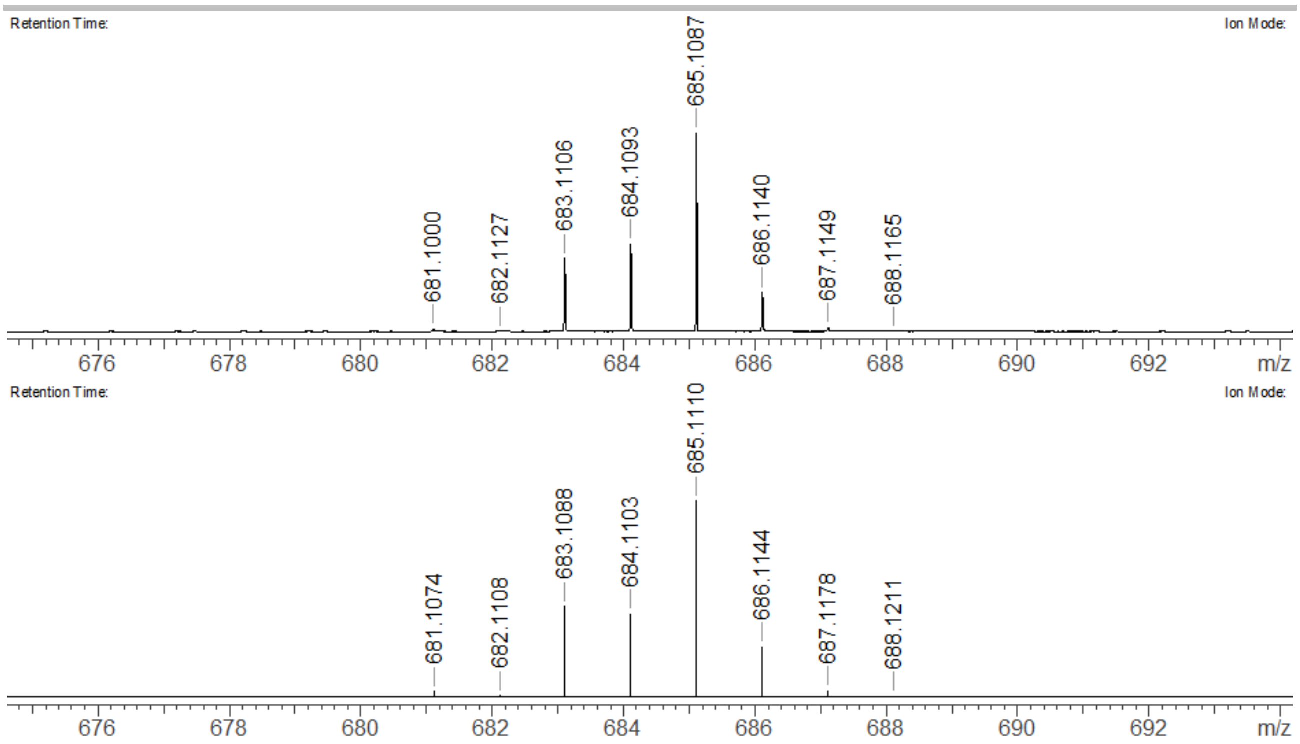

Figure S 11. The high-resolution mass spectrum of **20b-Pb** (ESI, TOF, $[M+H]^+$). Top: experimental spectrum, bottom: simulated pattern.

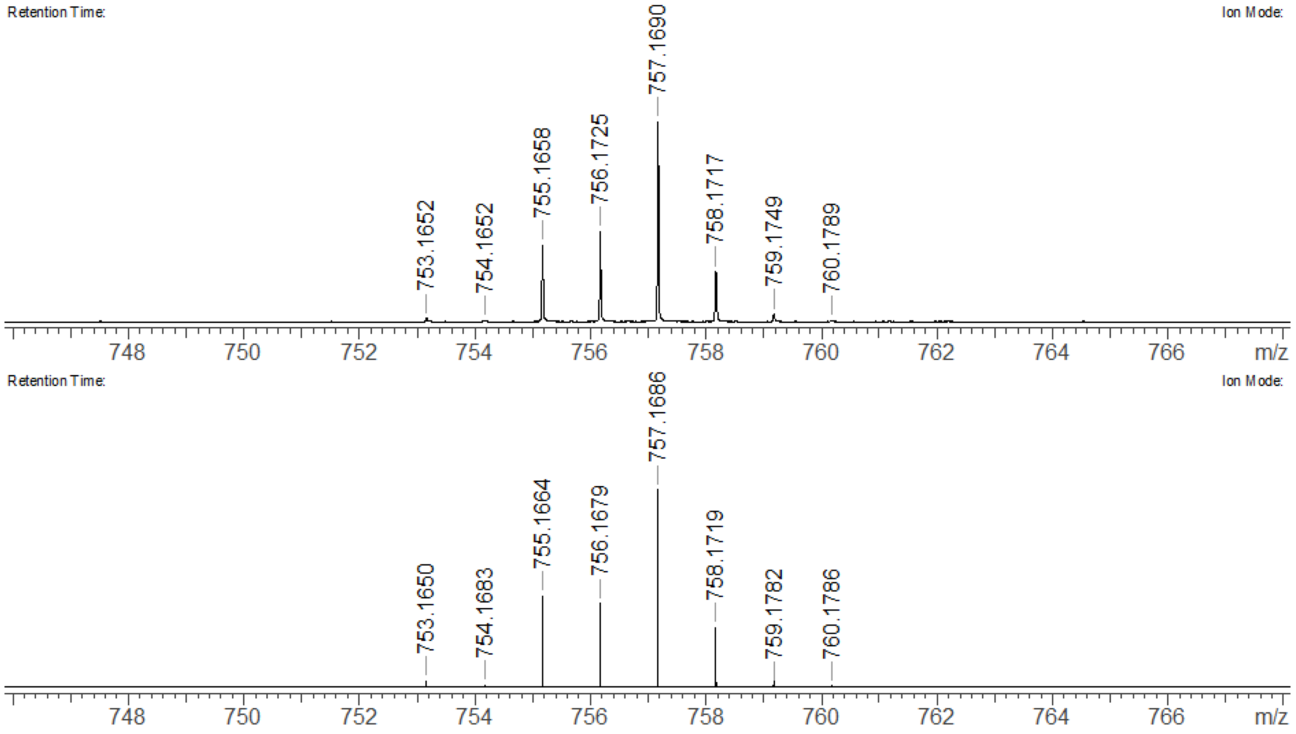

Figure S 12. The high-resolution mass spectrum of **21b-Pb** (ESI, TOF, $[M+H]^+$). Top: experimental spectrum, bottom: simulated pattern.

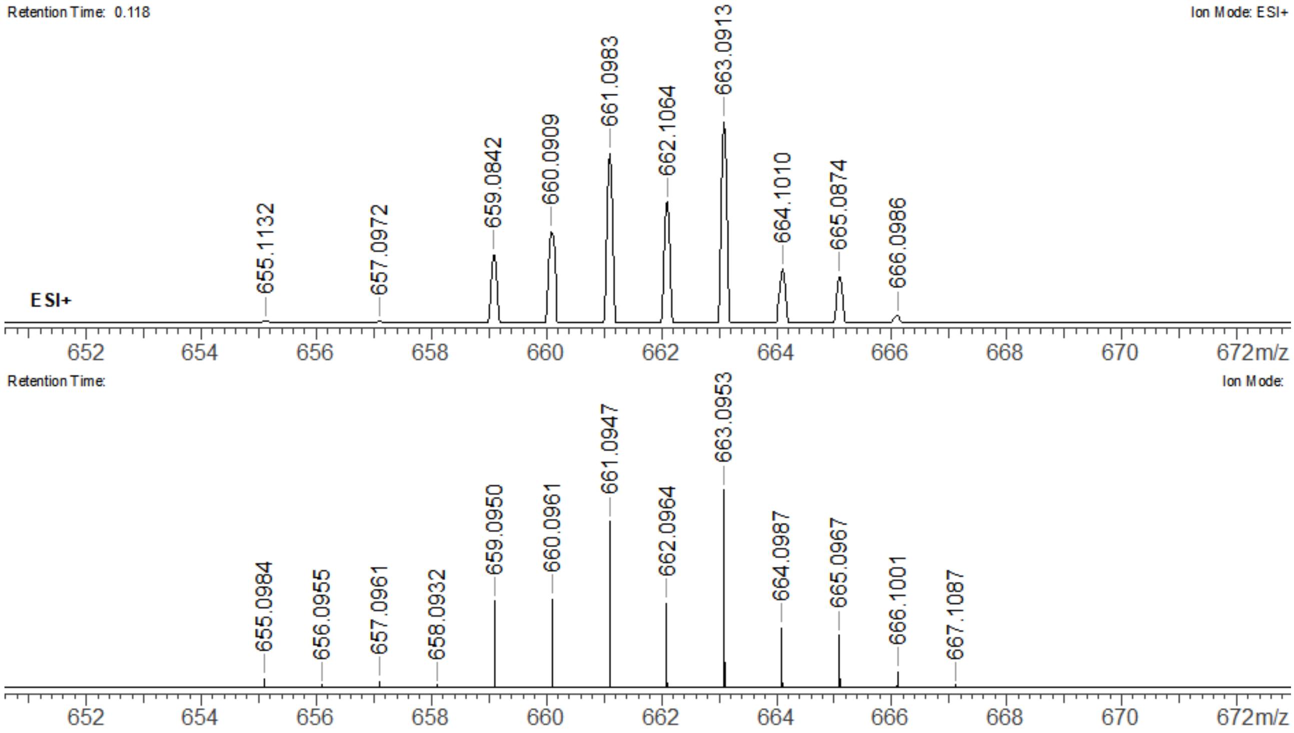

Figure S 13. The high-resolution mass spectrum of **21b-Cd** (ESI, TOF, $[M+H]^+$). Top: experimental spectrum, bottom: simulated pattern.

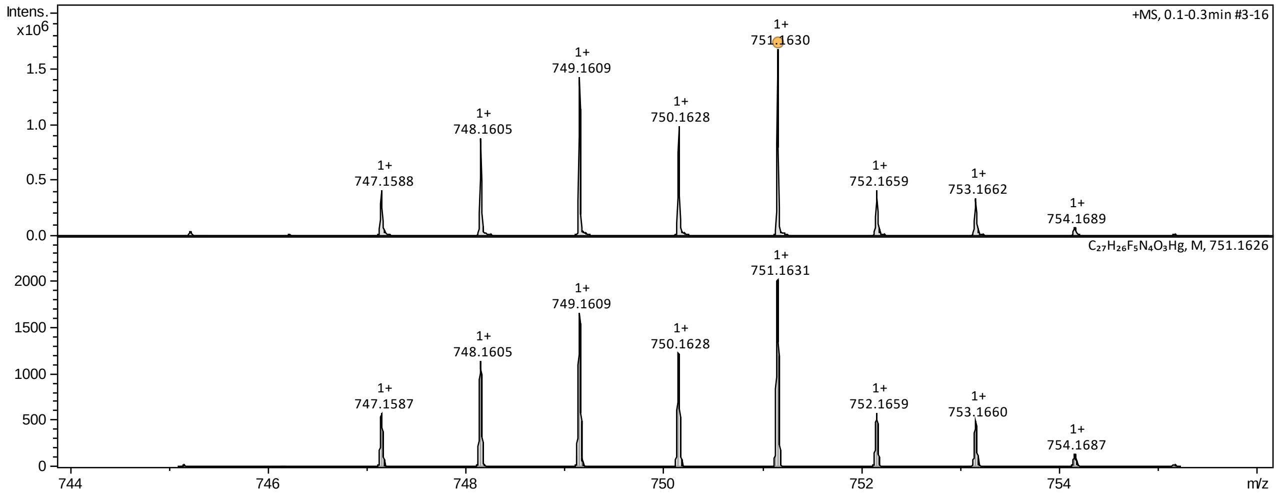

Figure S 14. The high-resolution mass spectrum of **21b-Hg** (ESI, TOF, $[M+H]^+$). Top: experimental spectrum, bottom: simulated pattern.

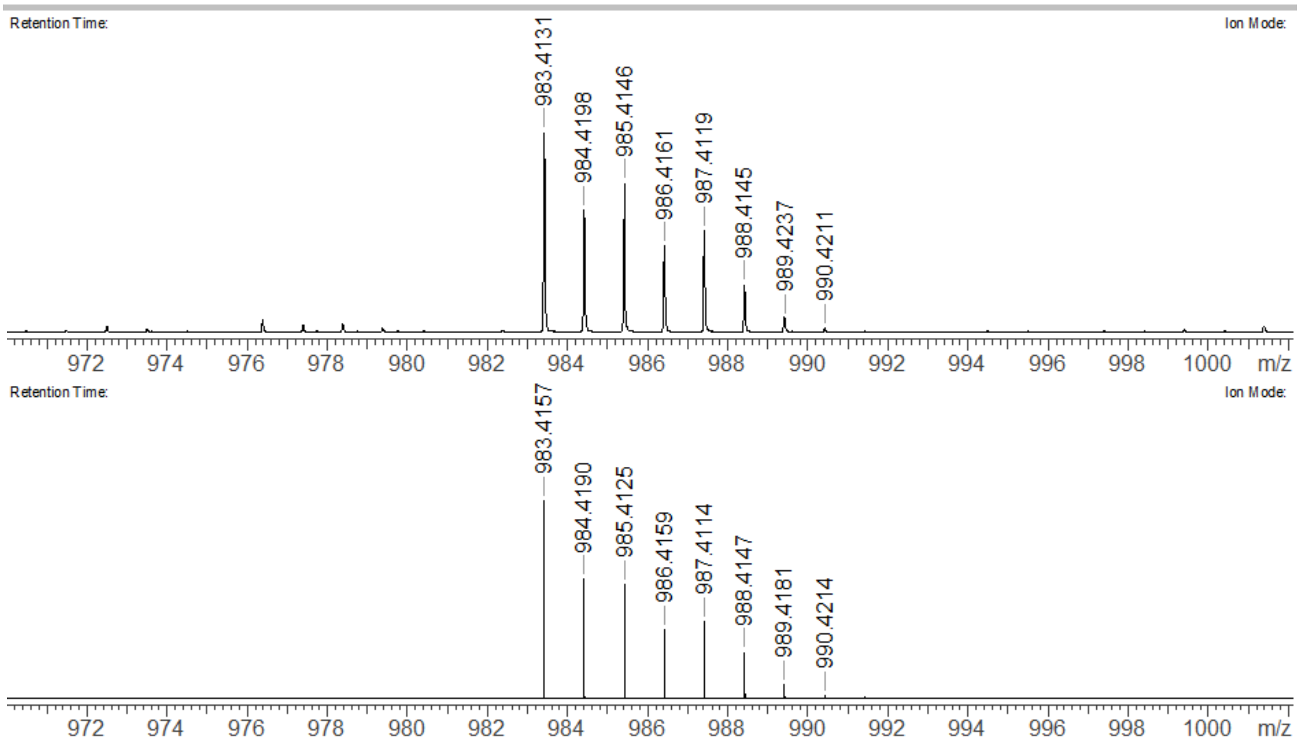

Figure S 15. The high-resolution mass spectrum of **22a-Zn** (ESI, TOF, $[M+H]^+$). Top: experimental spectrum, bottom: simulated pattern.

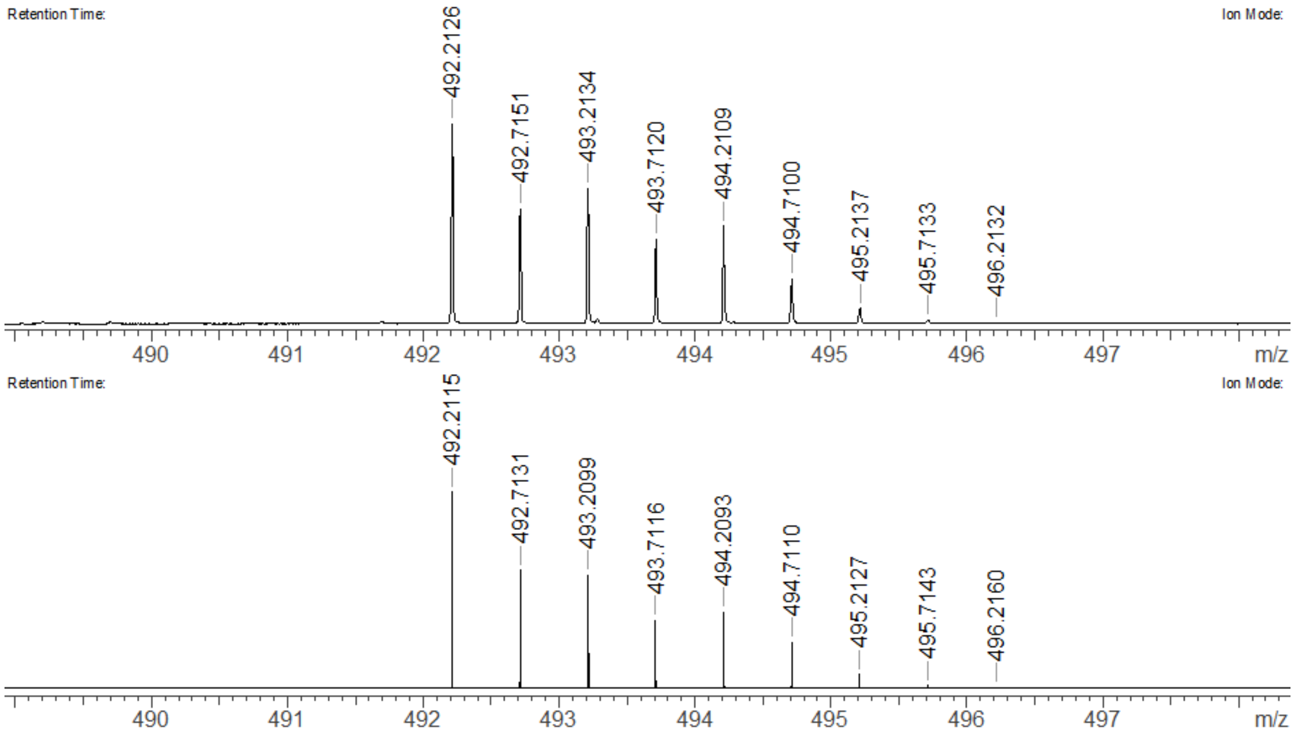

Figure S 16. The high-resolution mass spectrum of **22a-Zn** (ESI, TOF, $[M+2H]^{2+}$). Top: experimental spectrum, bottom: simulated pattern.

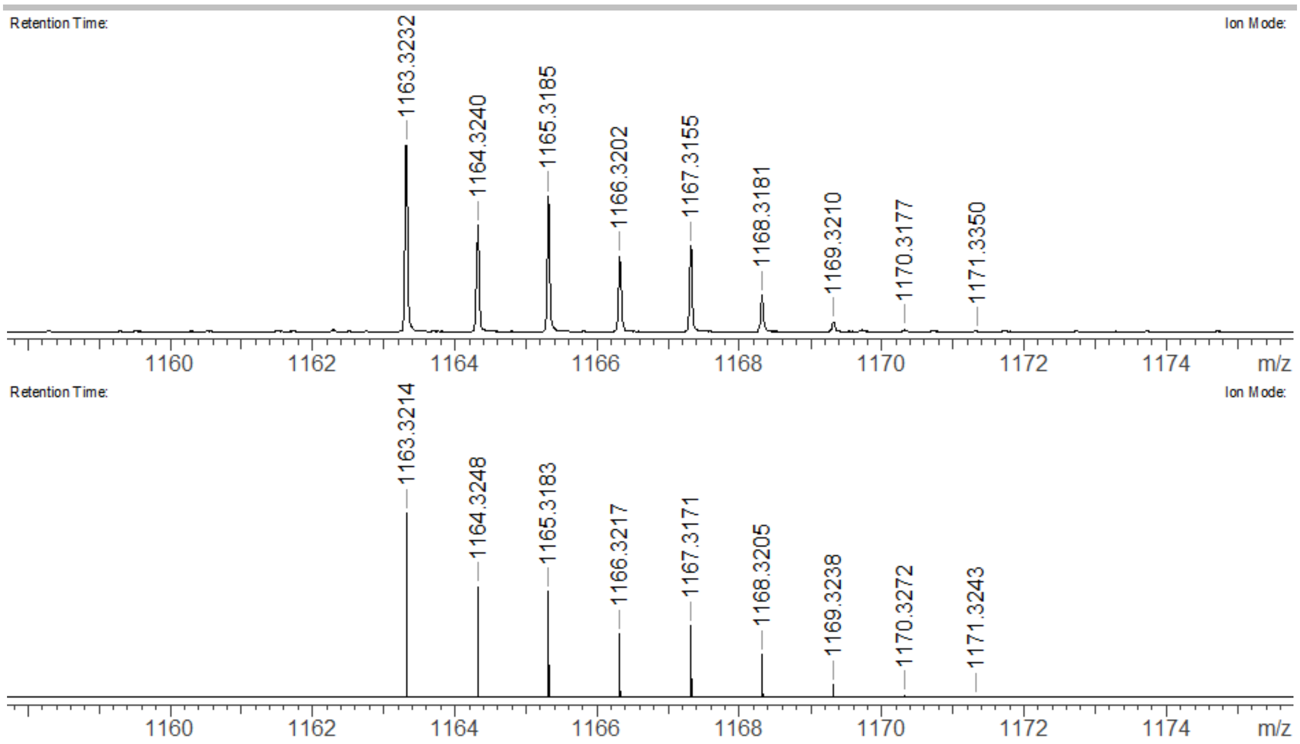

Figure S 17. Th e high-resolution mass spectrum of **22b-Zn** (ESI, TOF, $[M+H]^+$). Top: experimental spectrum, bottom: simulated pattern.

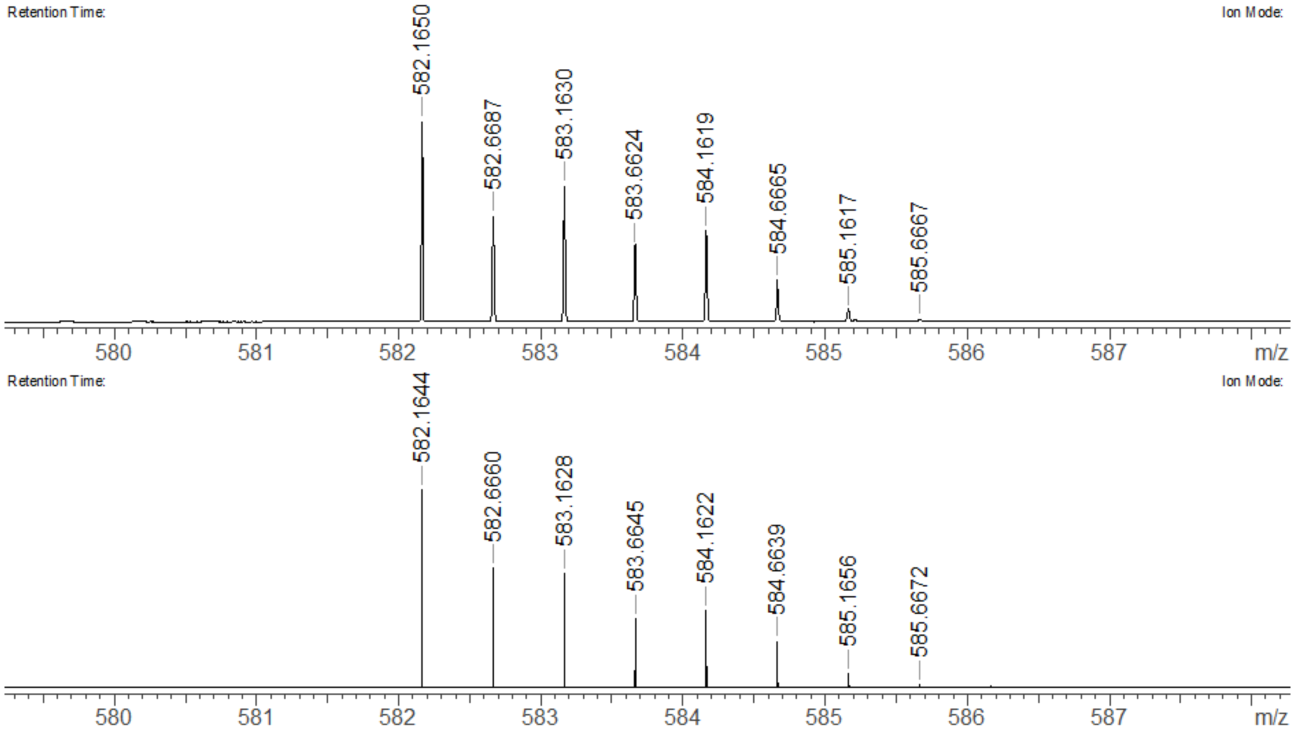

Figure S 18. Th e high-resolution mass spectrum of **22b-Zn** (ESI, TOF, $[M+2H]^{2+}$). Top: experimental spectrum, bottom: simulated pattern.

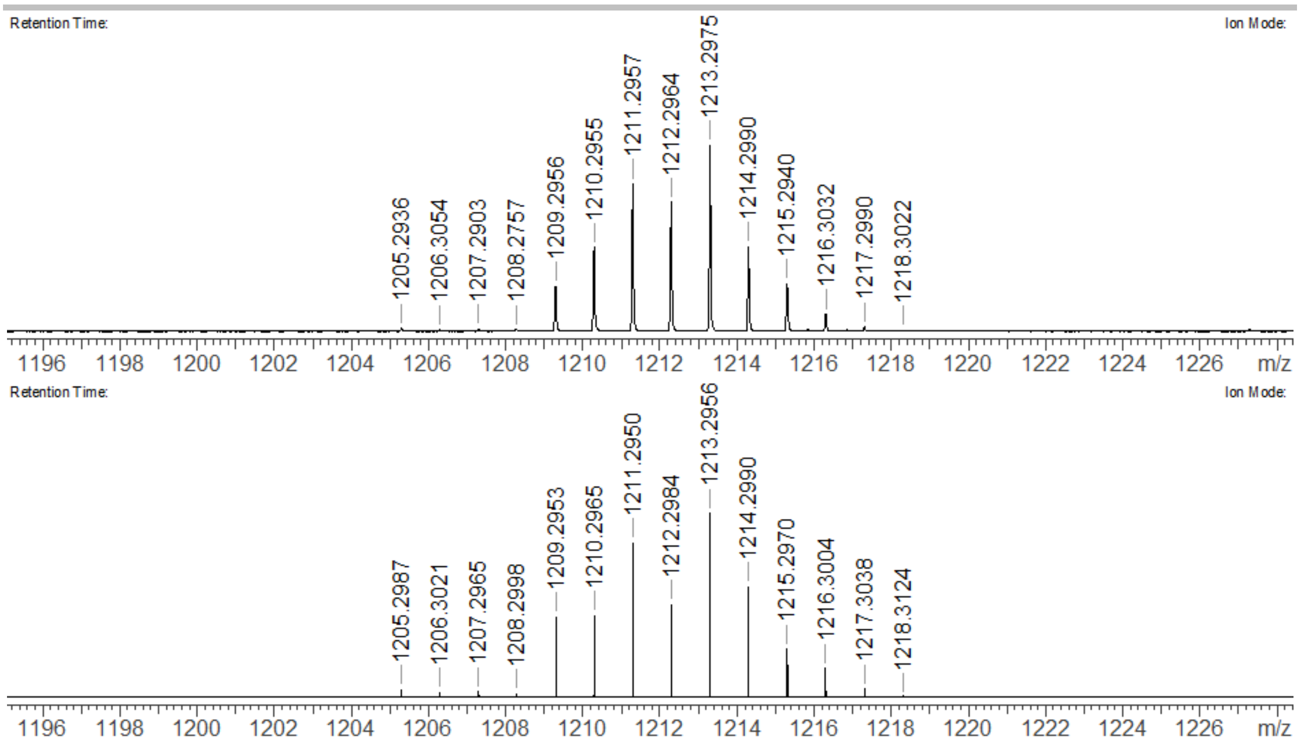

Figure S 19. The high-resolution mass spectrum of **22b-Cd** (ESI, TOF, $[M+H]^+$). Top: experimental spectrum, bottom: simulated pattern.

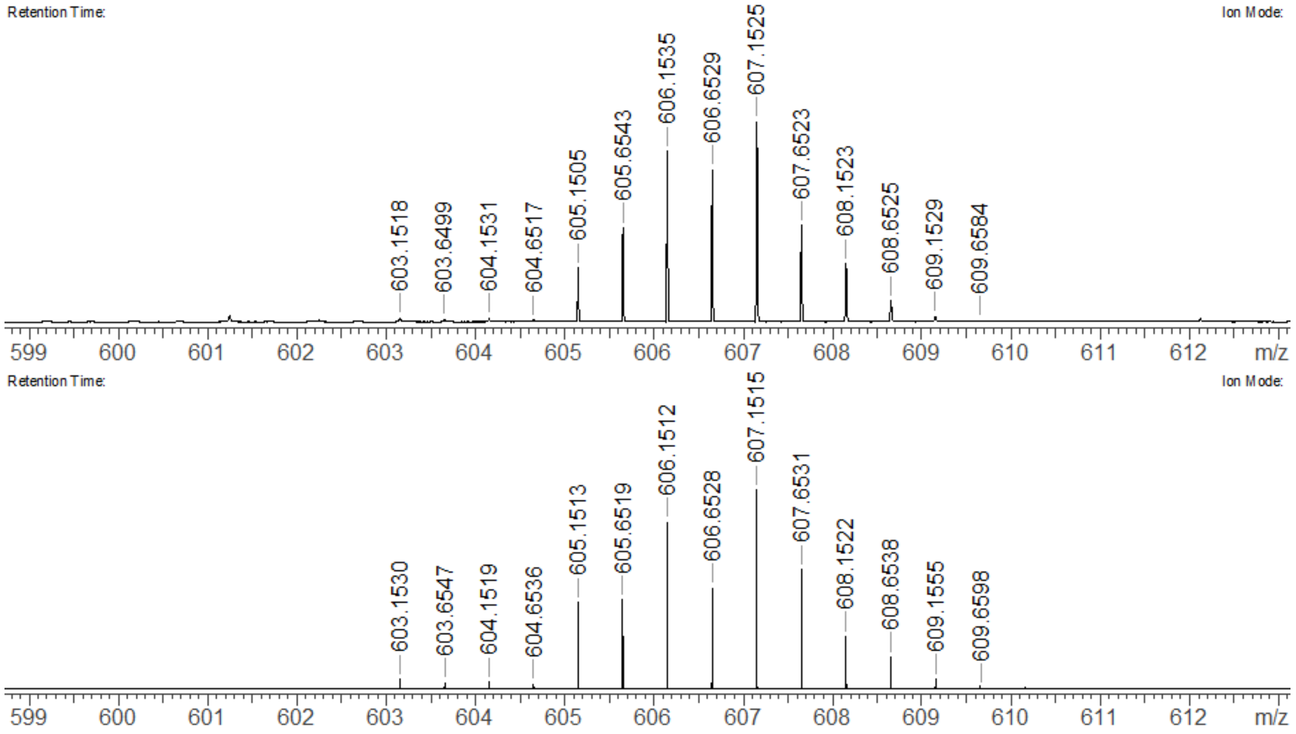

Figure S 20. The high-resolution mass spectrum of **22b-Cd** (ESI, TOF, $[M+2H]^{2+}$). Top: experimental spectrum, bottom: simulated pattern.

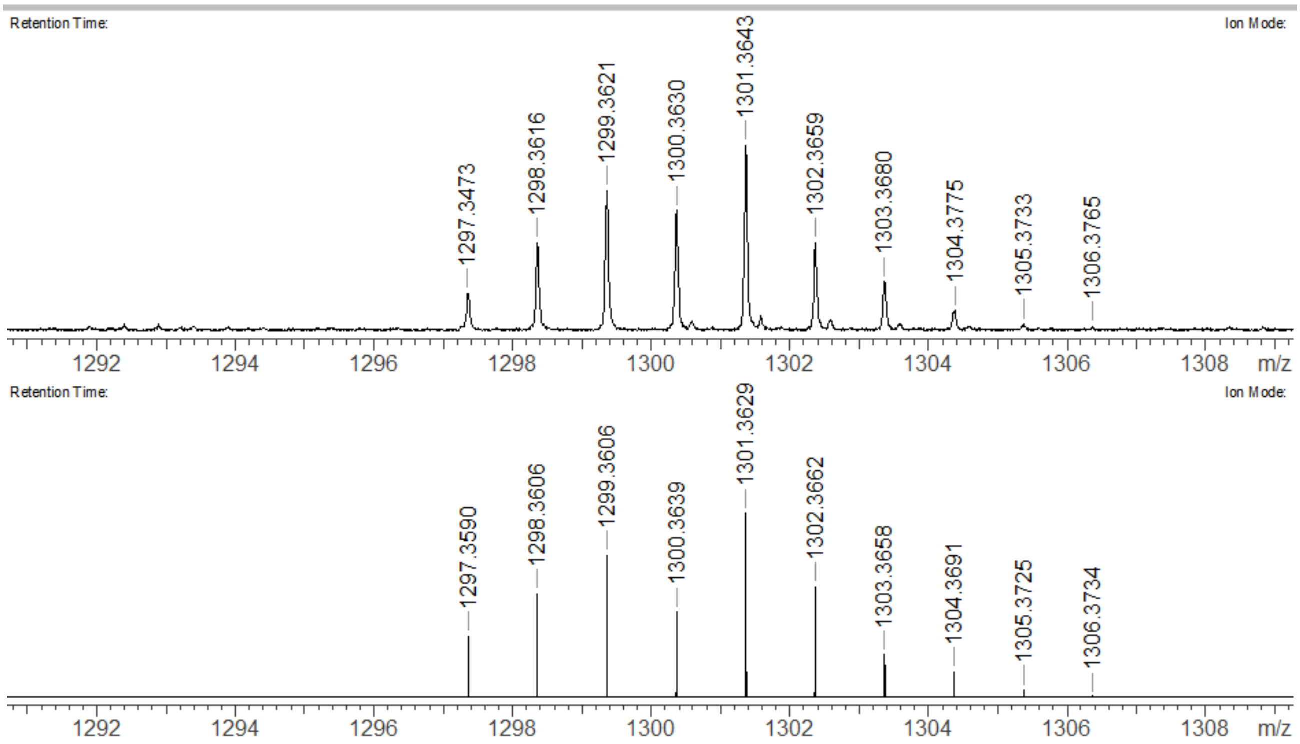

Figure S 21. The high-resolution mass spectrum of **22b-Hg** (ESI, TOF, $[M+H]^+$). Top: experimental spectrum, bottom: simulated pattern.

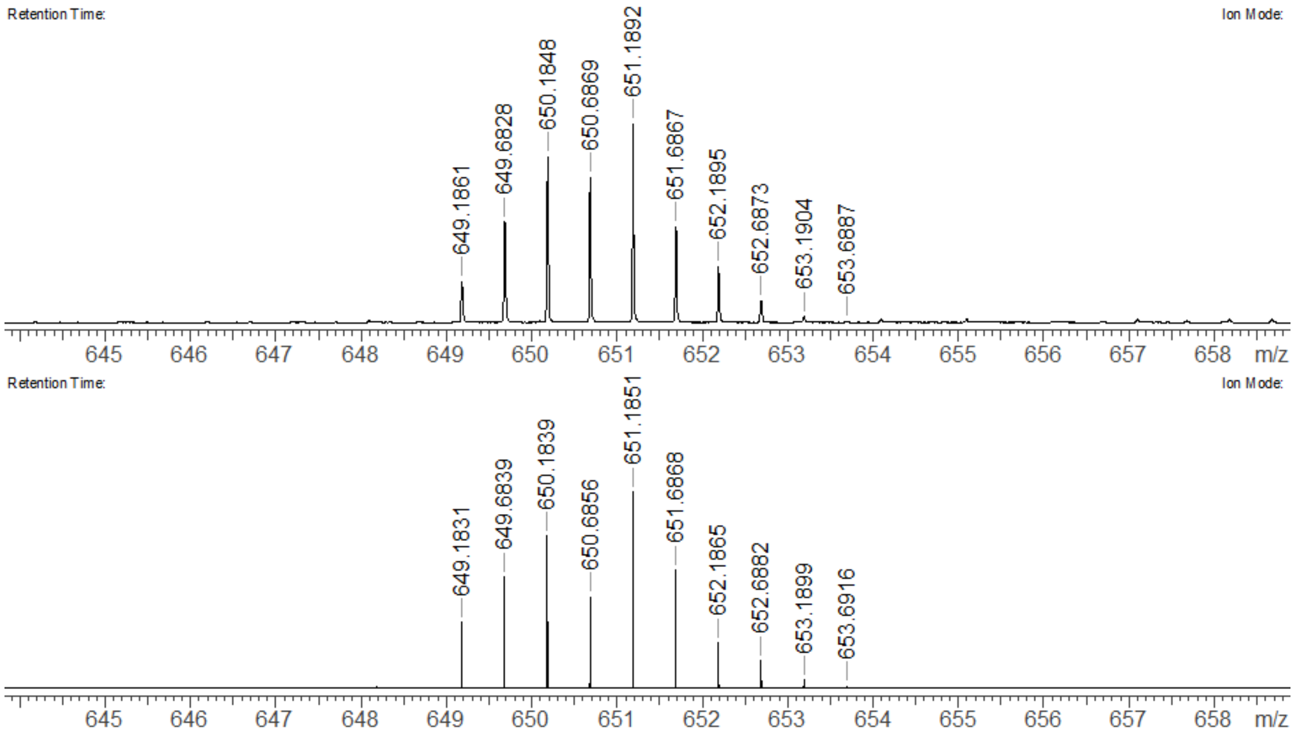

Figure S 22. The high-resolution mass spectrum of **22b-Hg** (ESI, TOF, $[M+2H]^{2+}$). Top: experimental spectrum, bottom: simulated pattern.

The NMR spectra of 14a-H$_3$

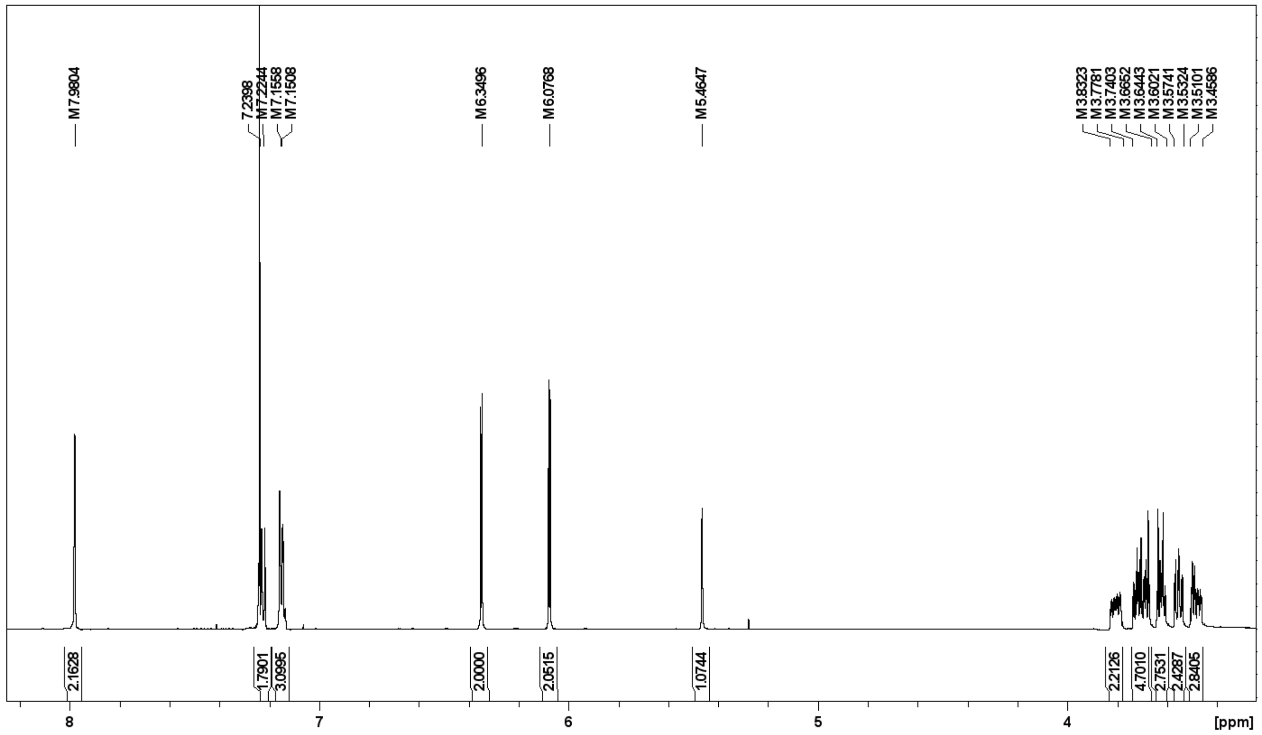

Figure S 23. The ^{1}H NMR spectrum of **14a-H$_3$** ([D]chloroform, 300 K, 600 MHz).

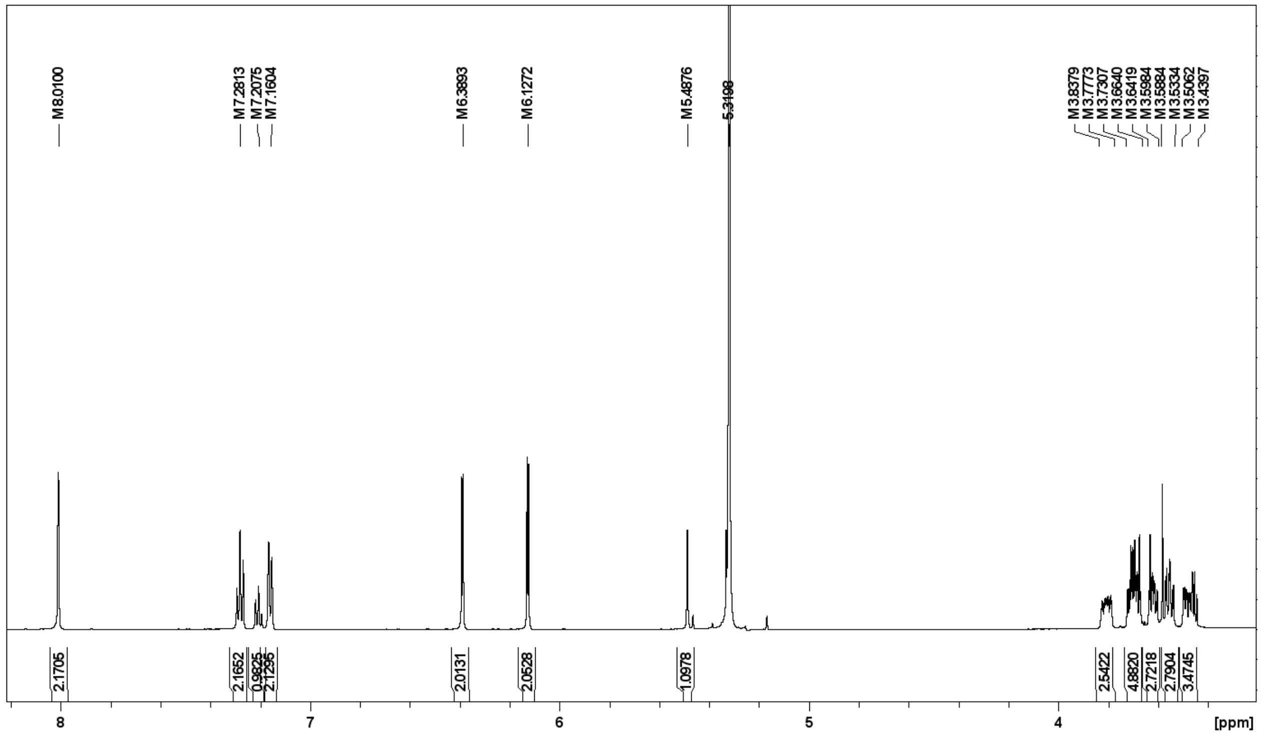

Figure S 24. The ^{1}H NMR spectrum of **14a-H$_3$** ([D$_2$]dichloromethane, 300 K, 600 MHz).

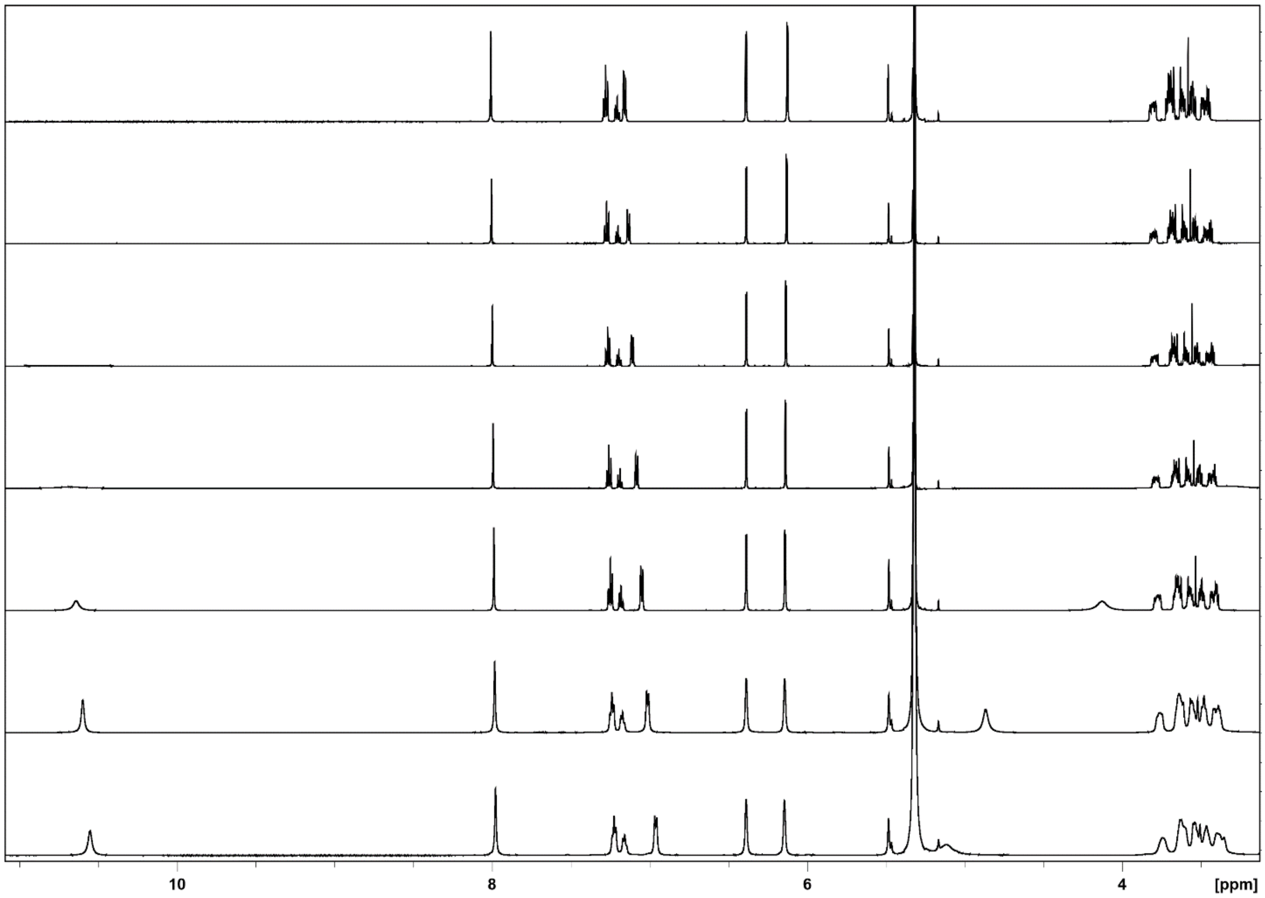

Figure S 25. ^{1}H NMR spectra of **14a-H$_3$** recorded in 300 K (top) – 180 K (bottom) temperature range every 20 K ([D$_2$]dichloromethane, 600 MHz).

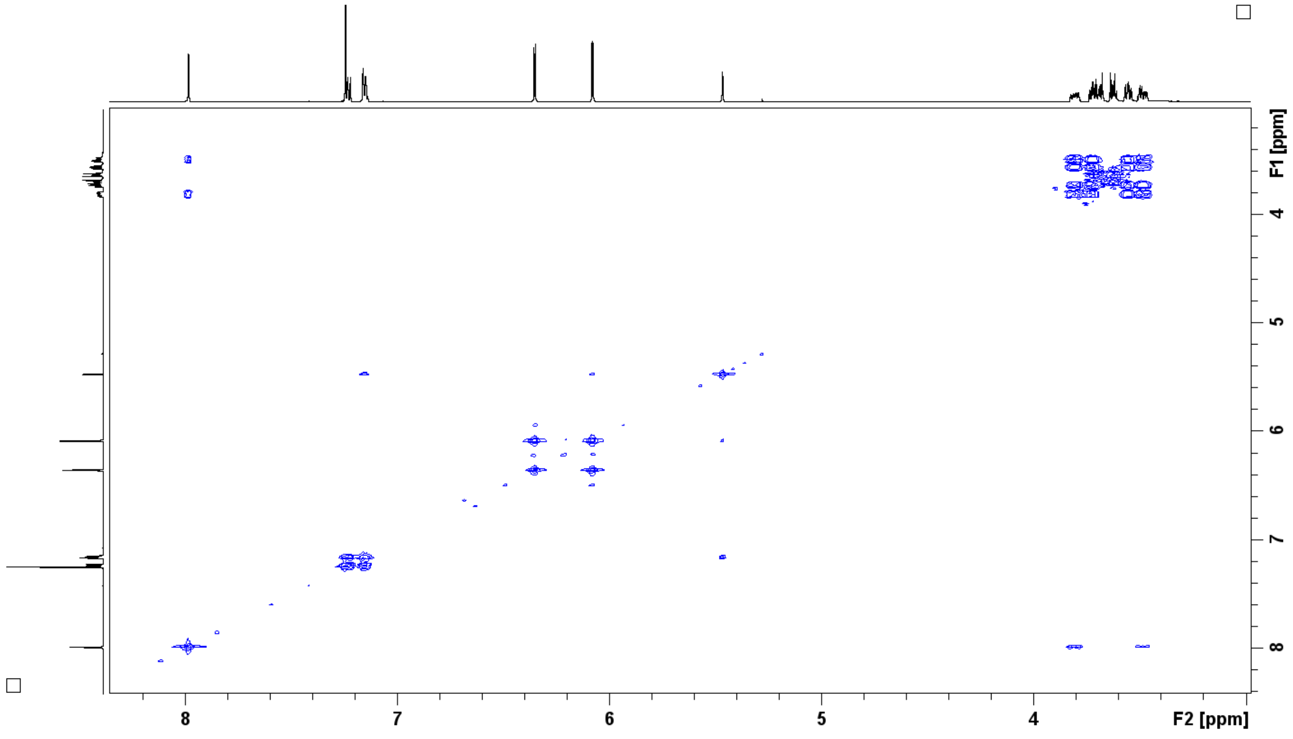

Figure S 26. The ^{1}H-^{1}H COSY spectrum of **14a-H$_3$** ([D]chloroform, 300 K, 600 MHz).

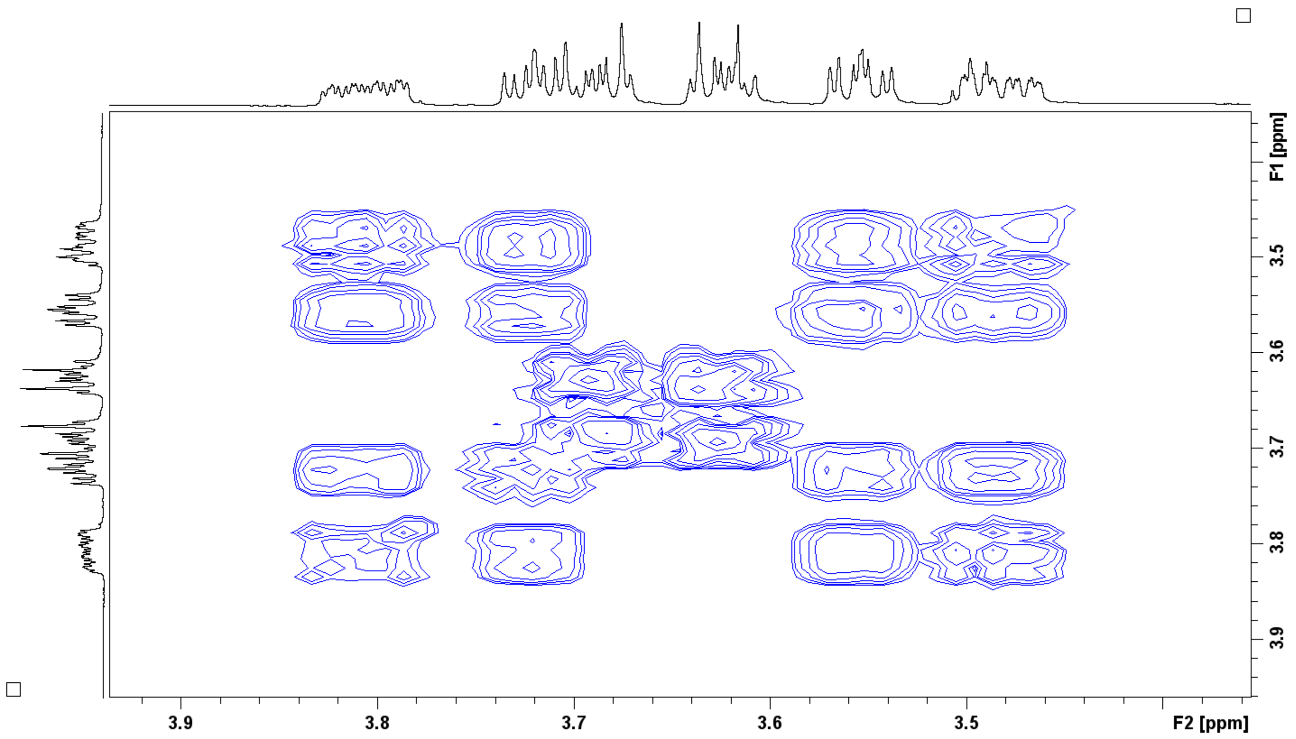

Figure S 27. The oligo(ethylene glycol) chain region of the ^{1}H-^{1}H COSY spectrum of **14a-H$_3$** ([D]chloroform, 300 K, 600 MHz).

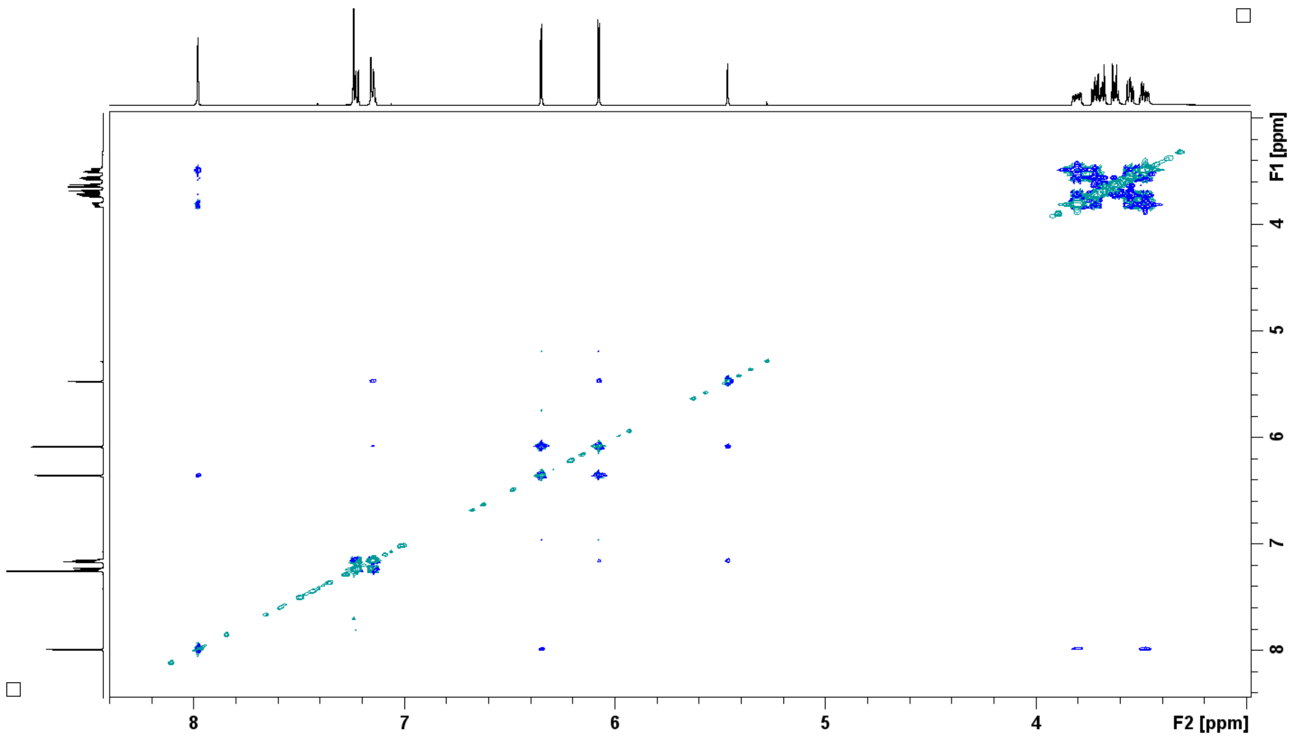

Figure S 28. The ^{1}H-^{1}H NOESY spectrum of **14a-H$_3$** ([D]chloroform, 300 K, 600 MHz).

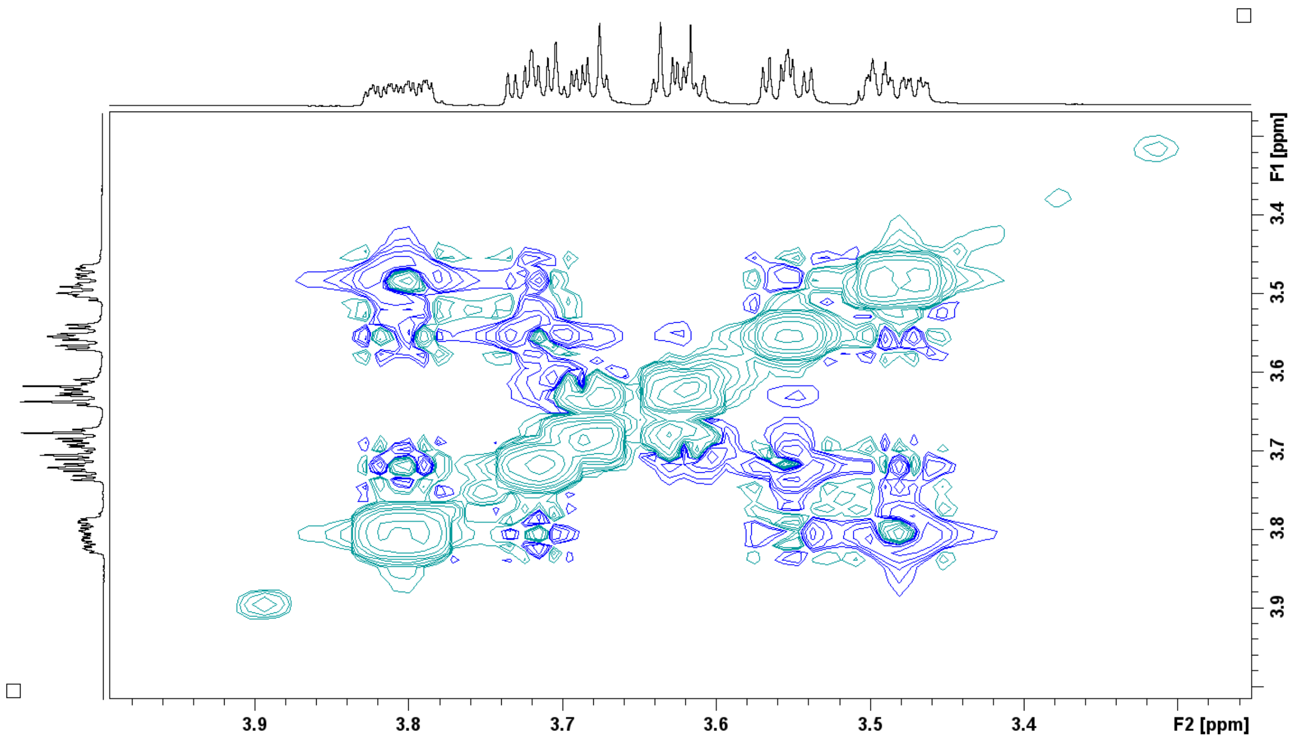

Figure S 29. The oligo(ethylene glycol) chain region of the NOESY spectrum of **14a-H_3** ([D]chloroform, 300 K, 600 MHz).

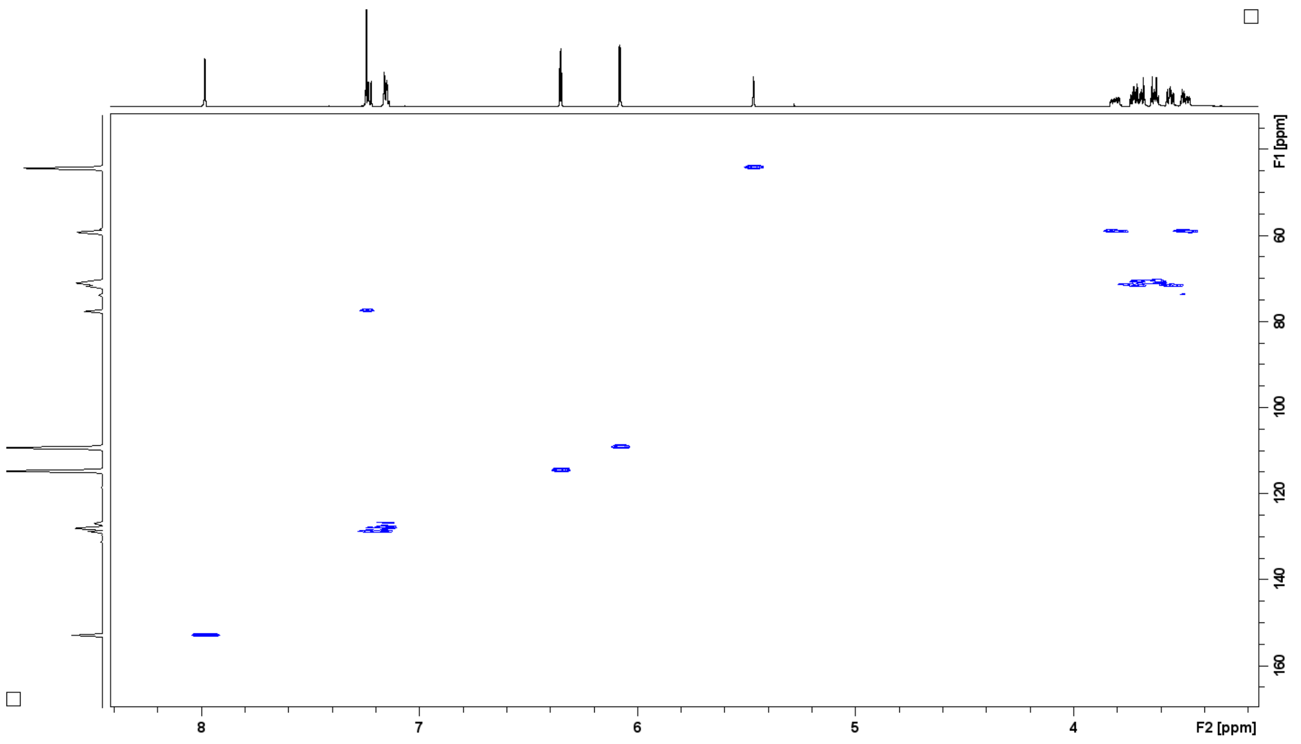

Figure S 30. The ^{1}H-^{13}C HMQC spectrum of **14a-H_3** ([D]chloroform, 300 K, 600 MHz).

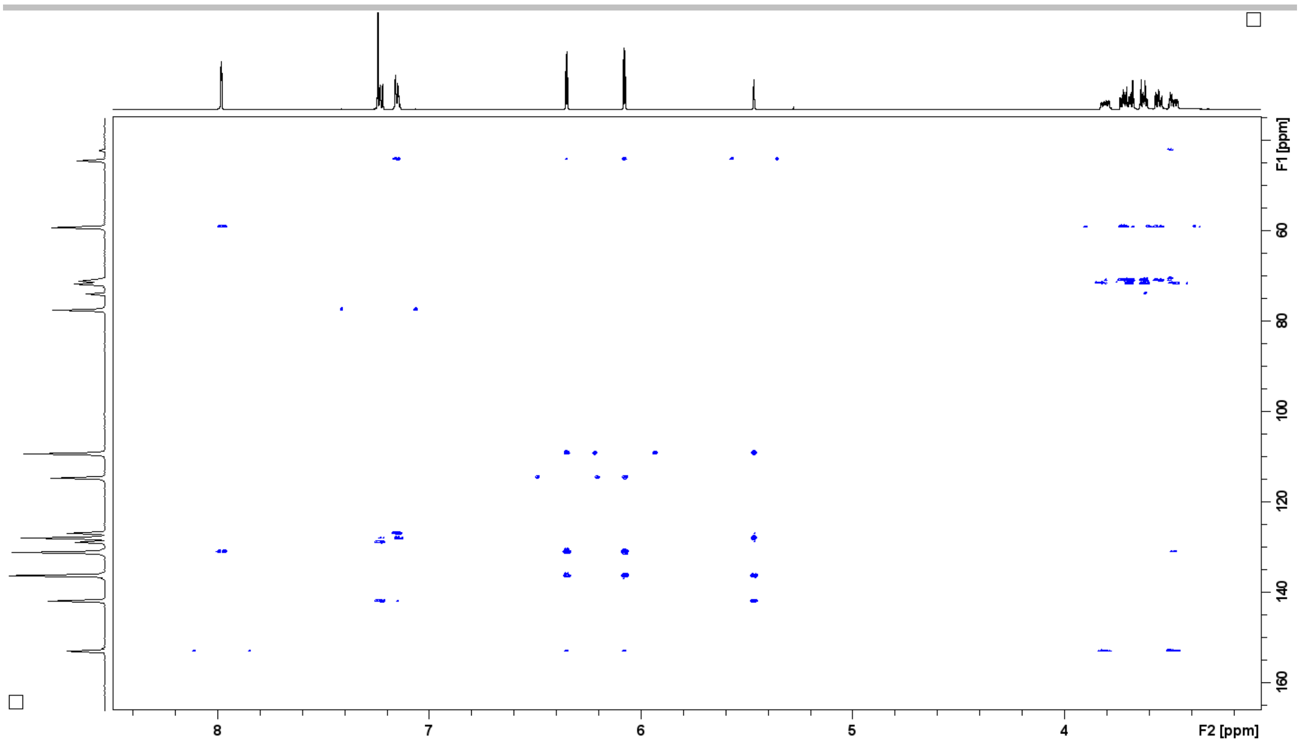

Figure S 31. The ^{1}H-^{13}C HMBC spectrum of **14a-H3** ([D]chloroform, 300 K, 600 MHz).

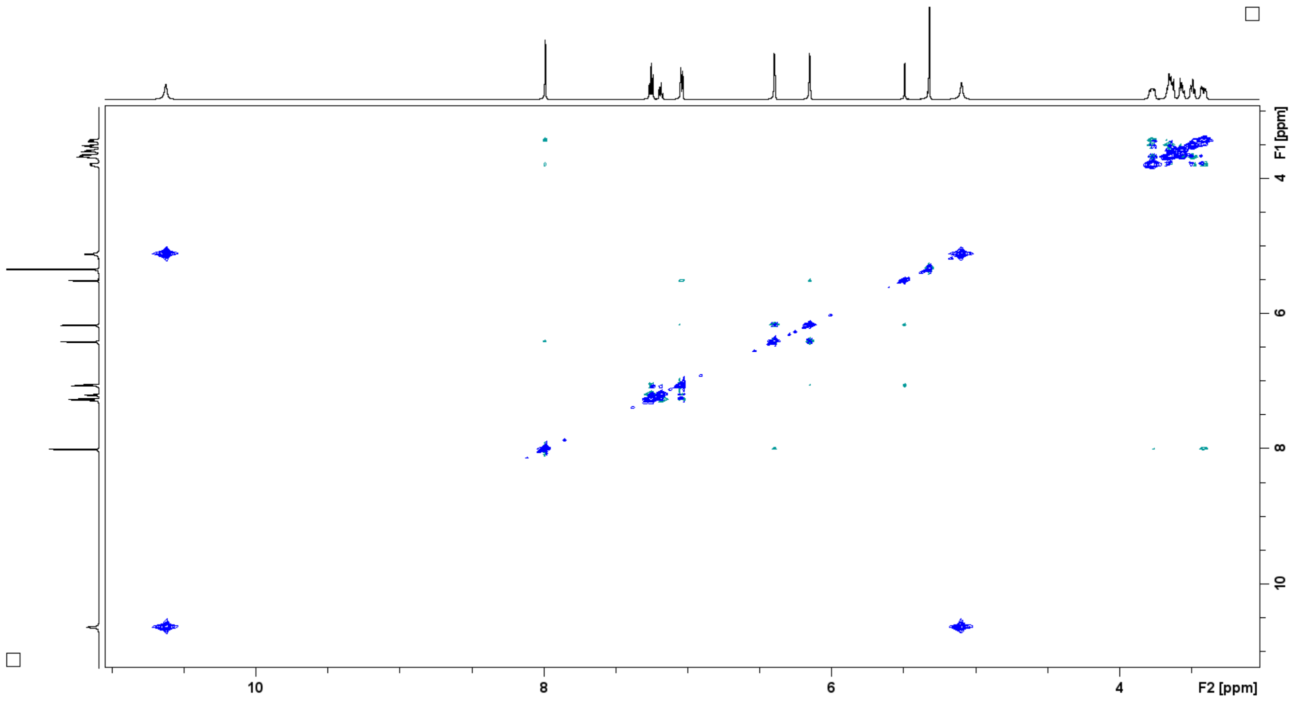

Figure S 32. The ^{1}H-^{1}H NOESY spectrum of **14a-H3** ([D2]dichloromethane, 210 K, 600 MHz).

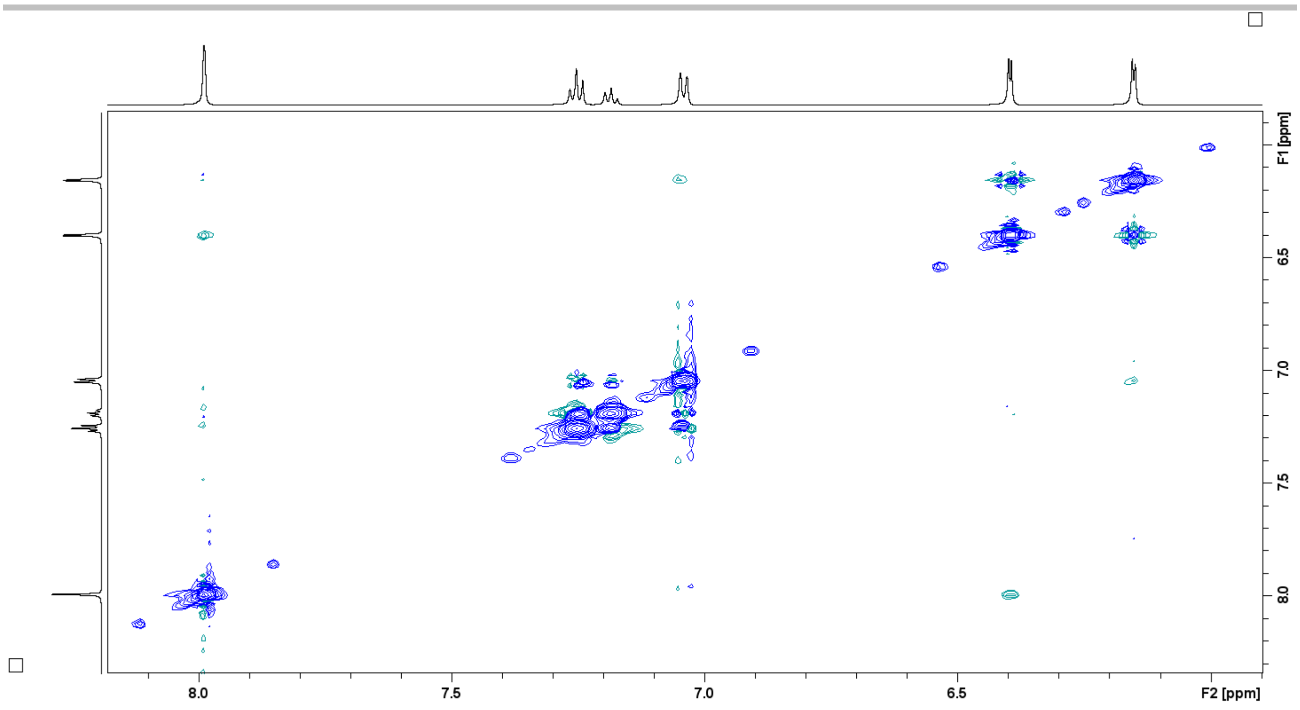

Figure S 33. The aromatic region of the ^{1}H-^{1}H NOESY spectrum of **14a-H$_3$** ([D$_2$]dichloromethane, 210 K, 600 MHz).

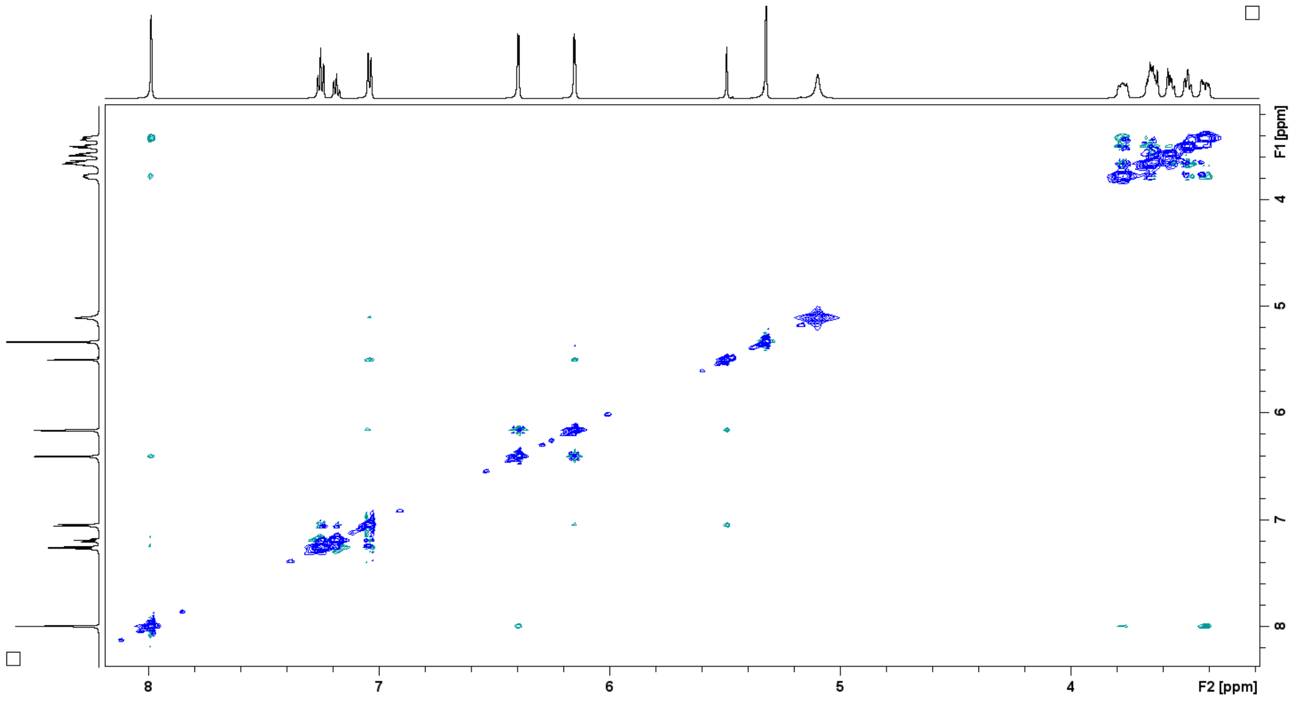

Figure S 34. The imine, aromatic and oligo(ethylene glycol) chain regions of the ^{1}H-^{1}H NOESY spectrum of **14a-H$_3$** ([D$_2$]dichloromethane, 210 K, 600 MHz).

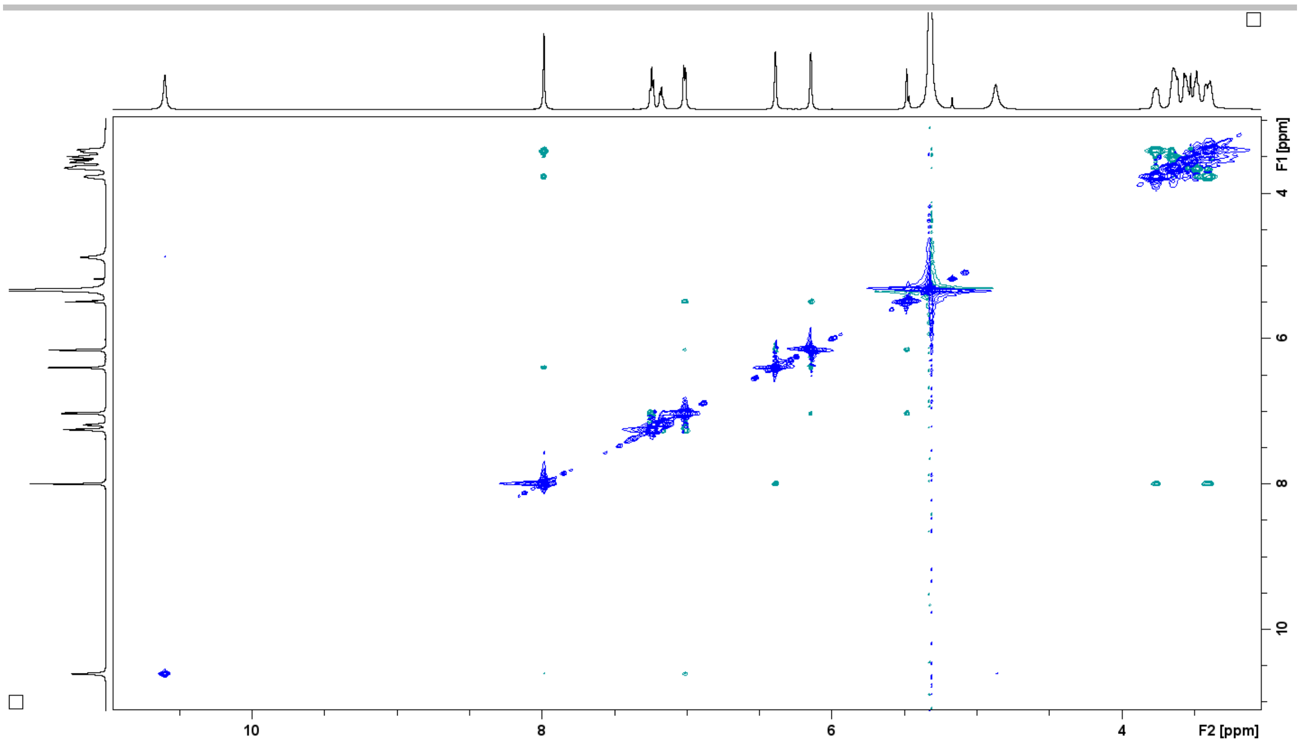

Figure S 35. The ^{1}H-^{1}H ROESY spectrum of **14a-H3** ([D2]dichloromethane, 200 K, 600 MHz).

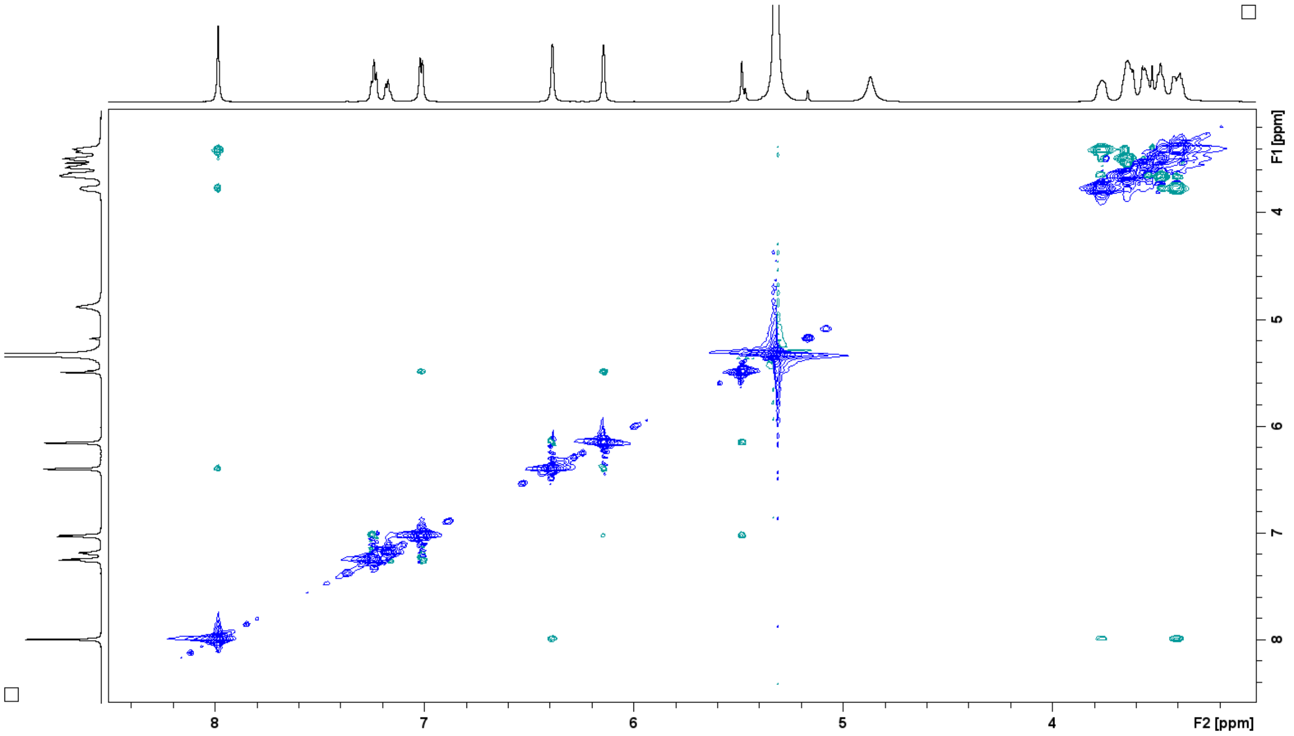

Figure S 36. The aromatic and oligo(ethylene glycol) chain regions of the ROESY spectrum of **14a-H3** ([D2]dichloromethane, 200 K, 600 MHz).

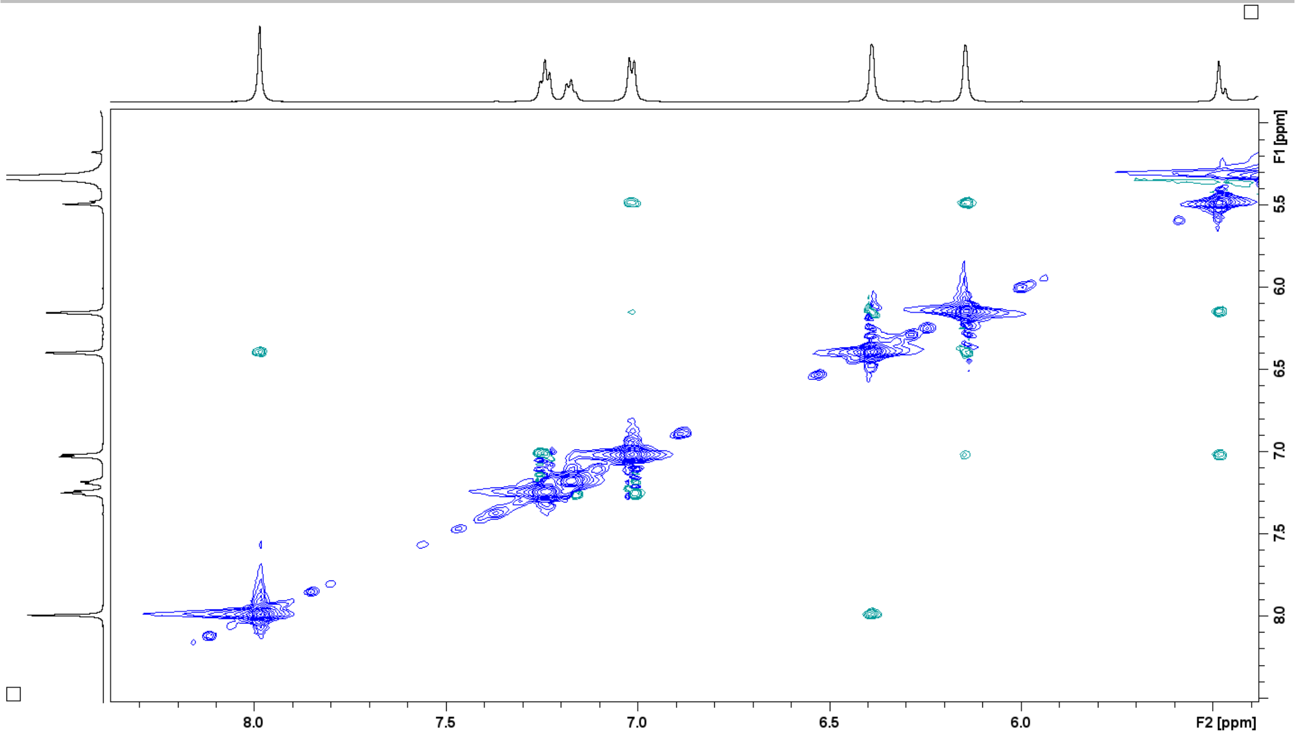

Figure S 37. The imine and aromatic region of the ROESY spectrum of **14a-H₃** ([D₂]dichloromethane, 200 K, 600 MHz).

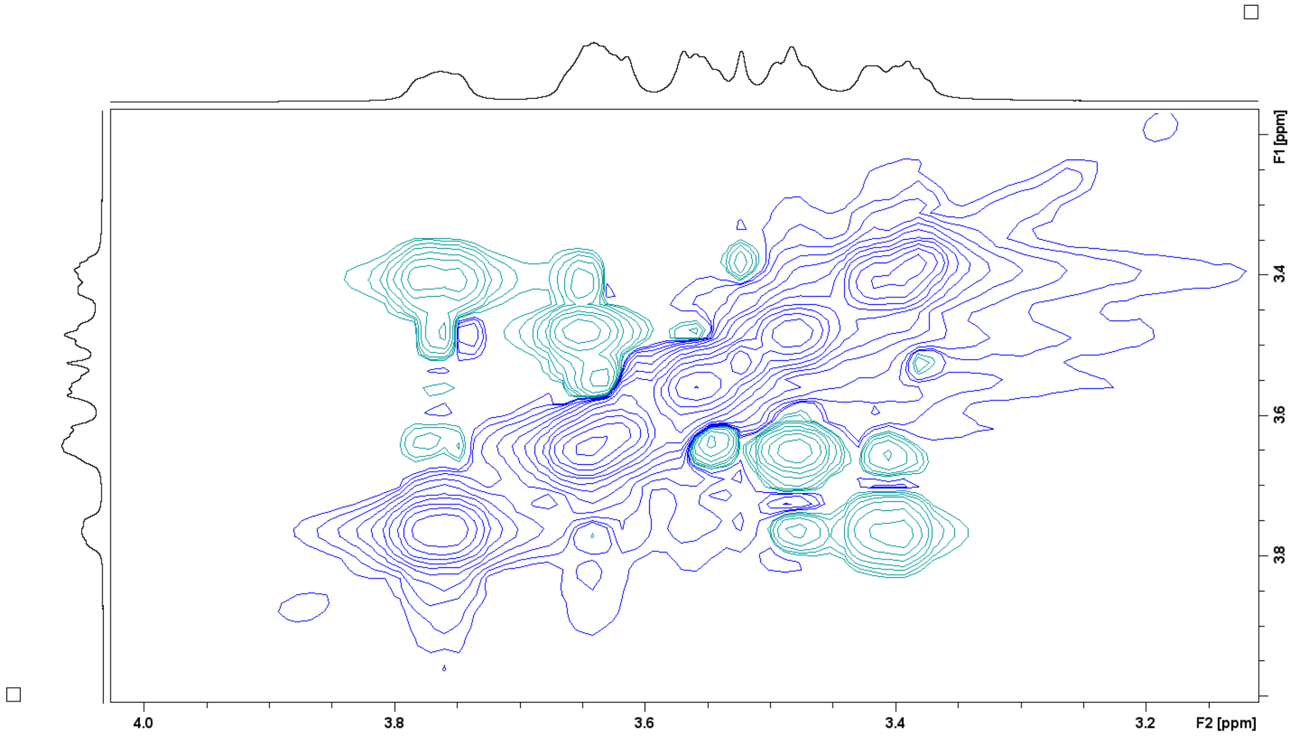

Figure S 38. The oligo(ethylene glycol) chain region of the ROESY spectrum of **14a-H₃** ([D₂]dichloromethane, 200 K, 600 MHz).

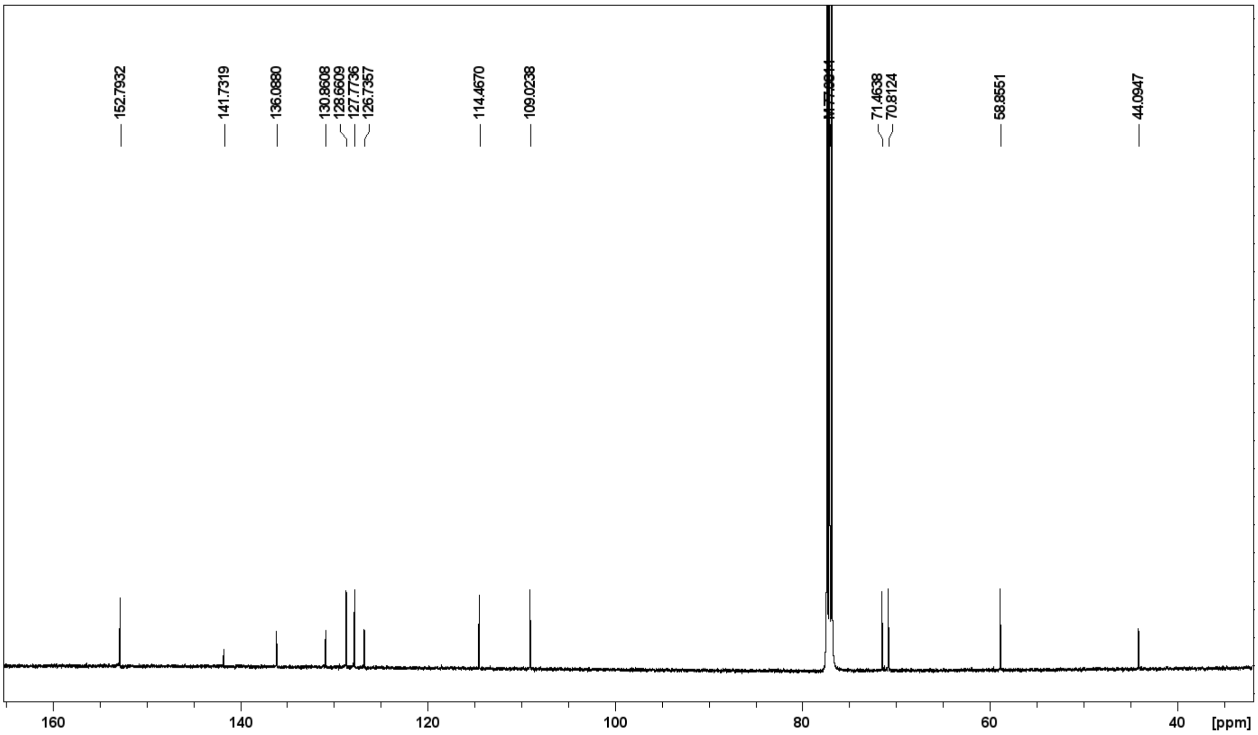

Figure S 39. The ^{13}C NMR spectrum of **14a-H$_3$** ([D]chloroform, 300 K, 125 MHz).

The NMR spectra of 14a-H7

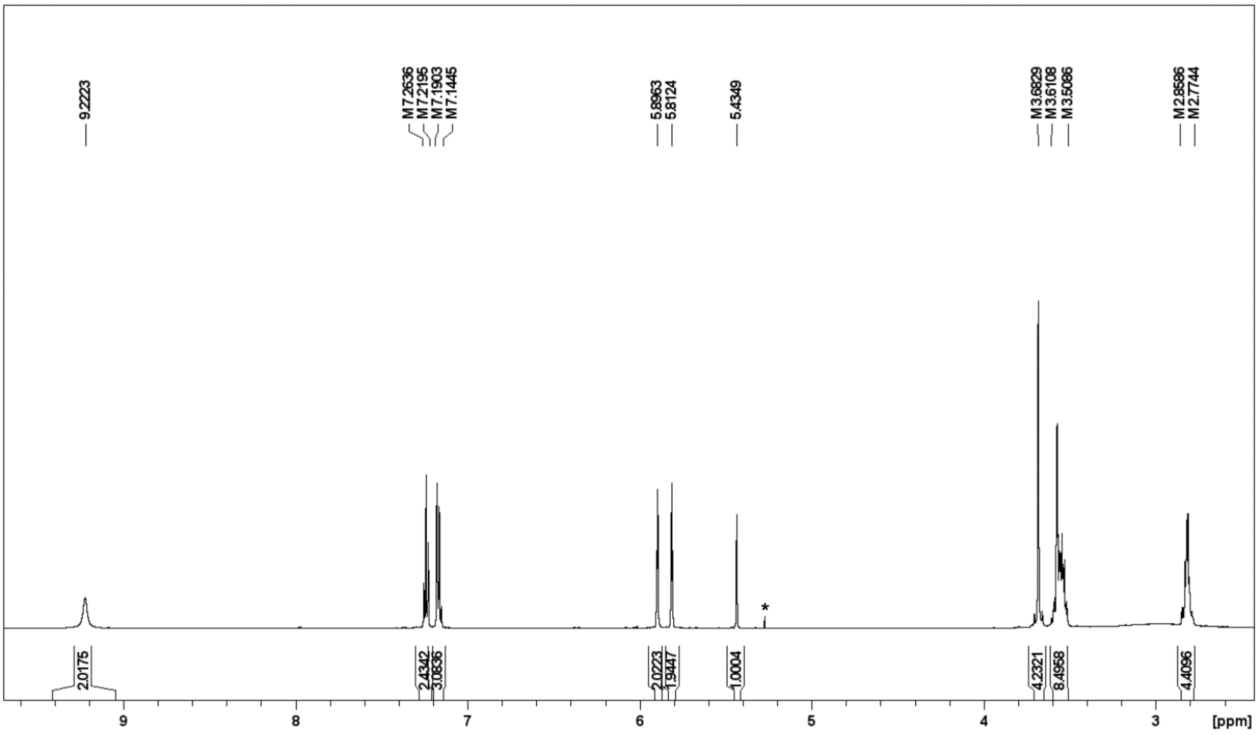

Figure S 40. The ^{1}H NMR spectrum of **14a-H7** ([D]chloroform, 300 K, 600 MHz).

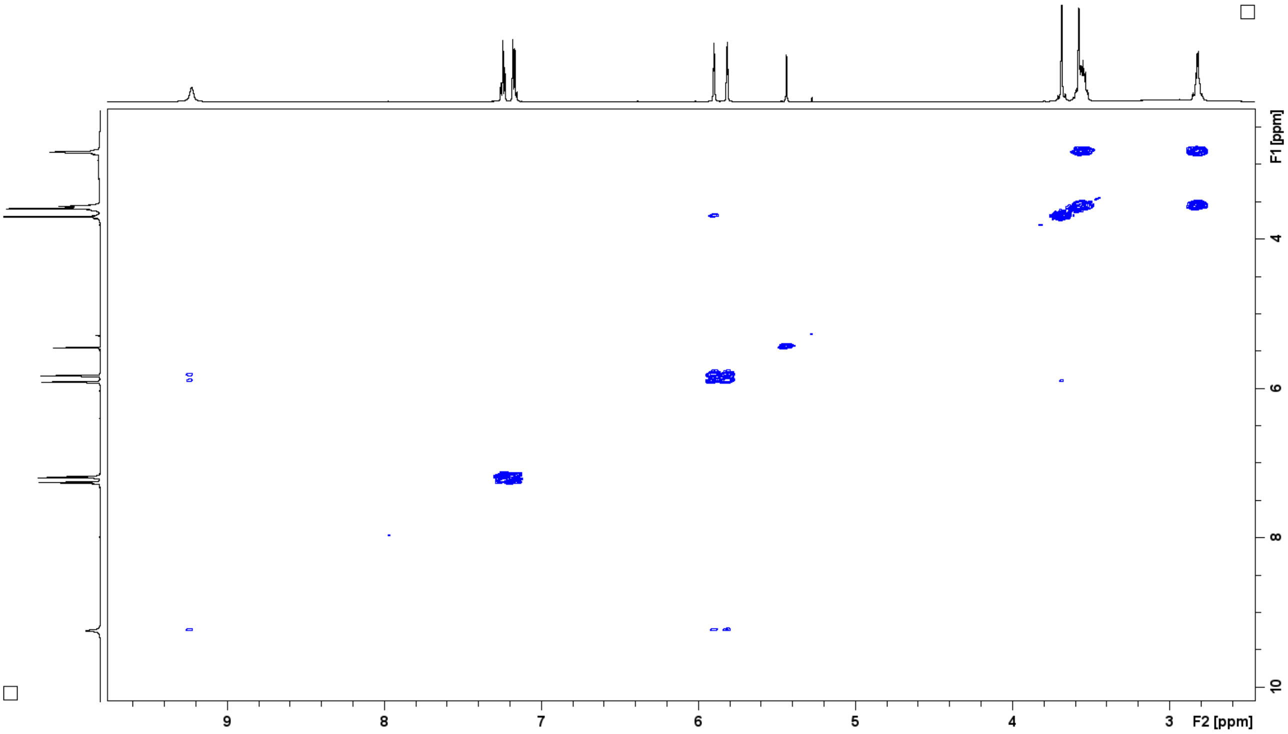

Figure S 41. The ^{1}H-^{1}H COSY spectrum of **14a-H7** ([D]chloroform, 300 K, 600 MHz).

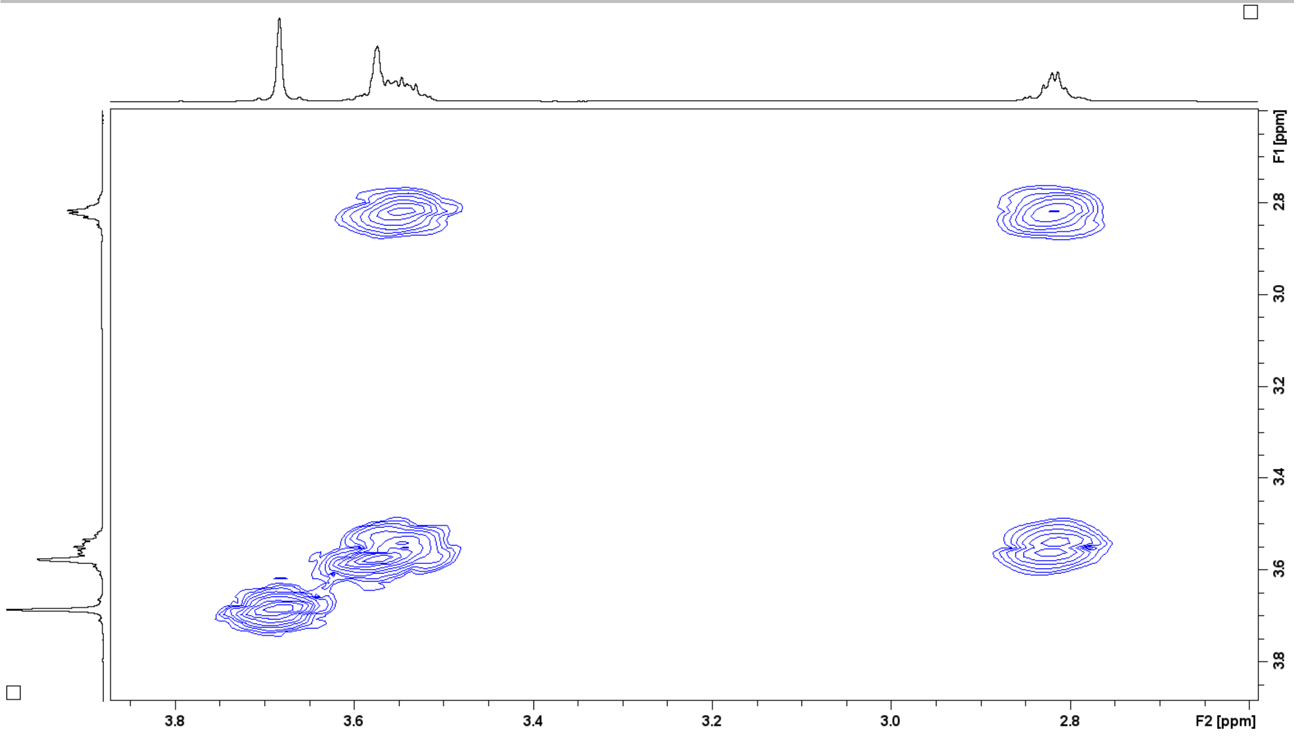

Figure S 42. The oligo(ethylene glycol) chain region of the ^{1}H-^{1}H COSY spectrum of **14a-H$_7$** ([D]chloroform, 300 K, 600 MHz).

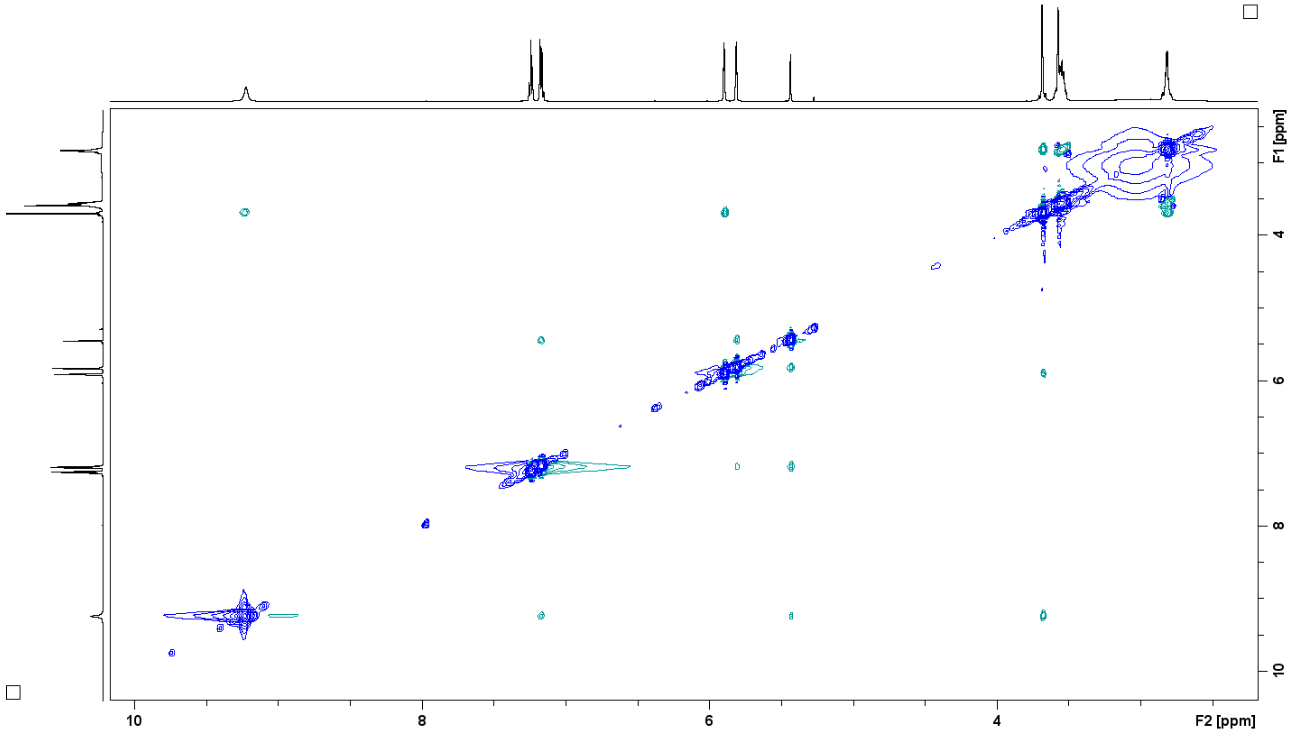

Figure S 43. The ^{1}H-^{1}H NOESY spectrum of **14a-H$_7$** ([D]chloroform, 300 K, 600 MHz).

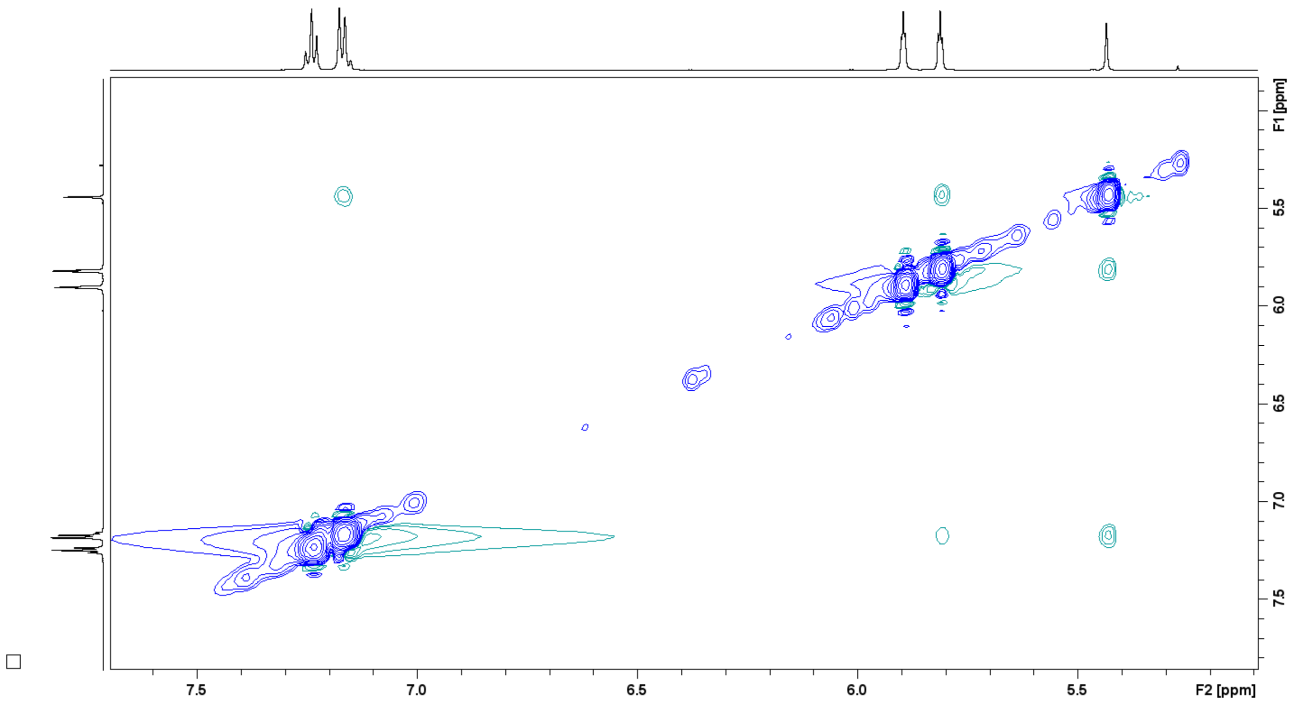

Figure S 44. The aromatic region of the NOESY spectrum of **14a-H$_7$** ([D]chloroform, 300 K, 600 MHz).

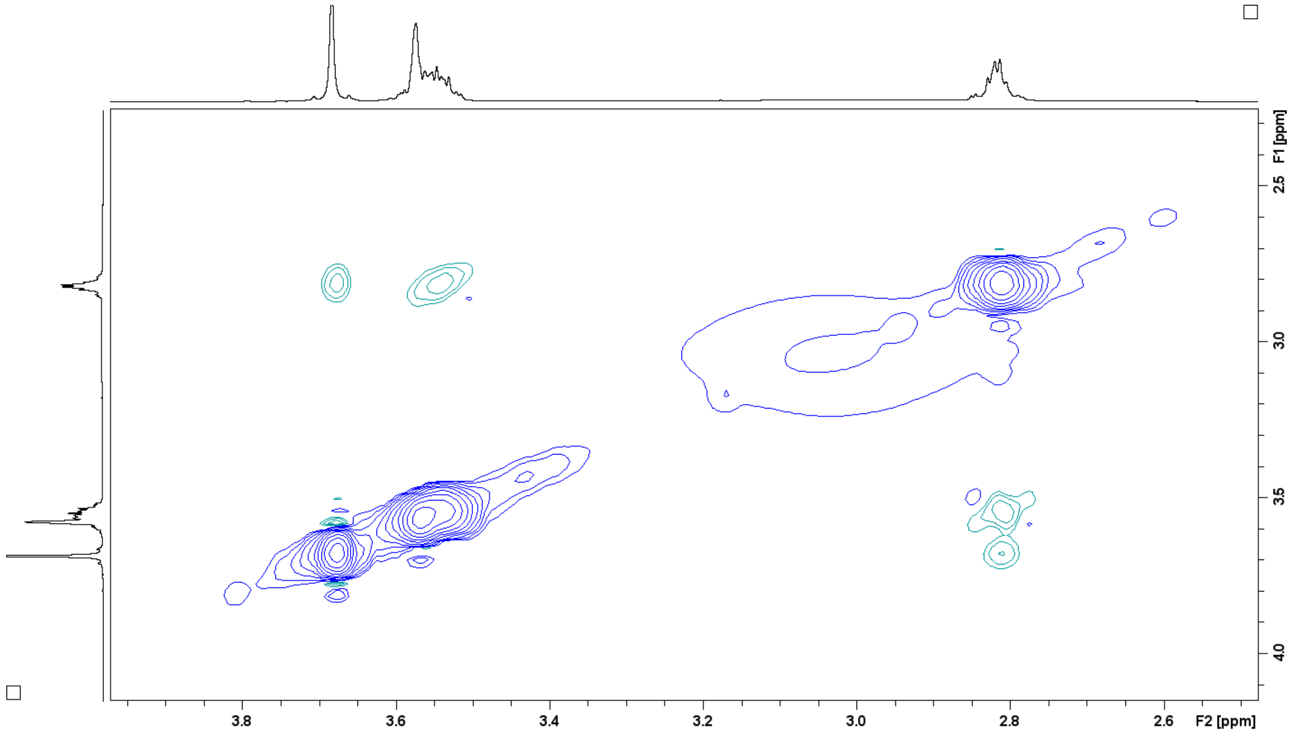

Figure S 45. The oligo(ethylene glycol) chain region of the NOESY spectrum of **14a-H$_7$** ([D]chloroform, 300 K, 600 MHz).

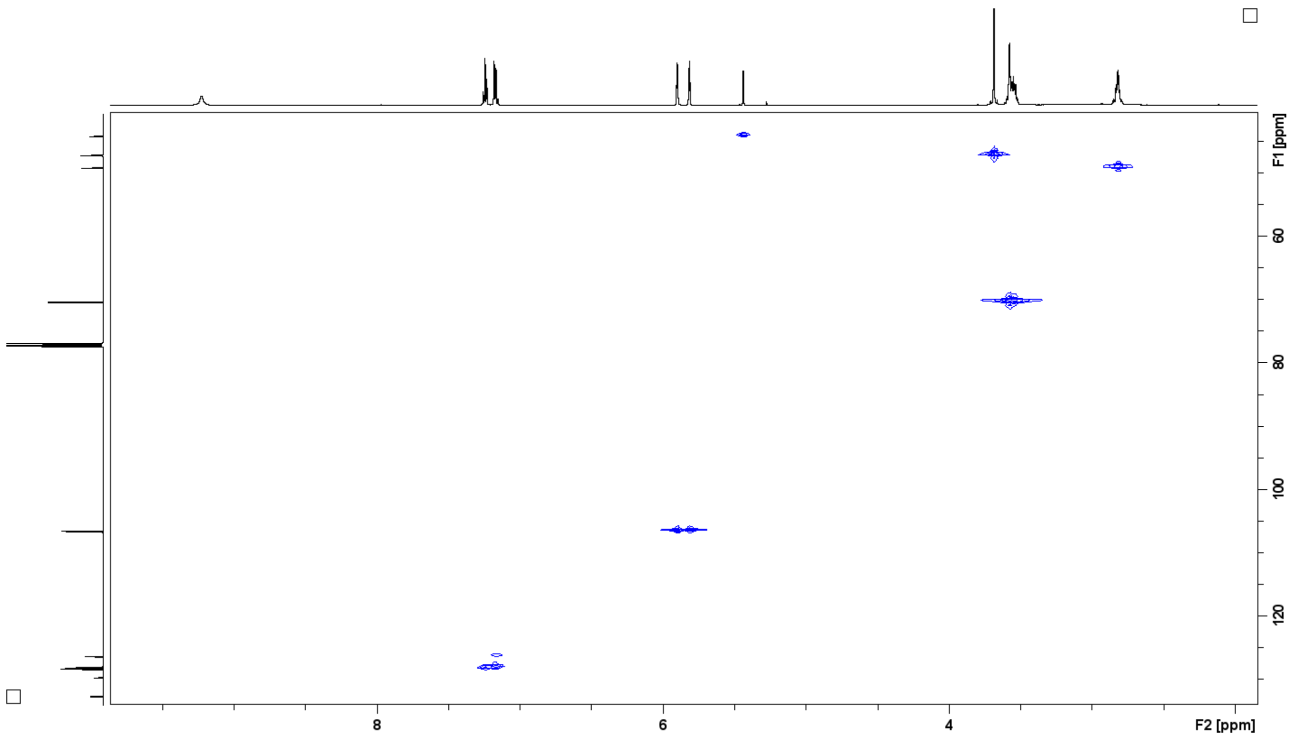

Figure S 46. The ^{1}H-^{13}C HMQC spectrum of **14a-H₇** ([D]chloroform, 300 K, 600 MHz).

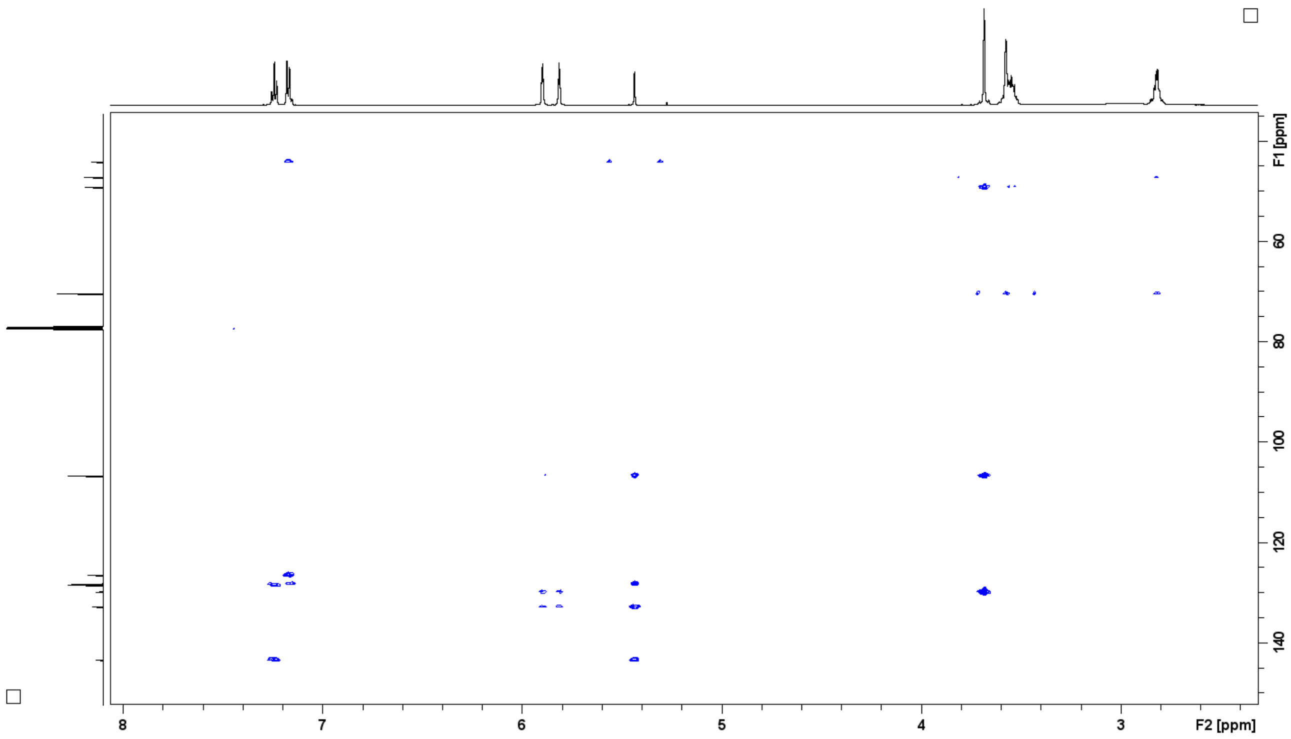

Figure S 47. The ^{1}H-^{13}C HMBC spectrum of **14a-H₇** ([D]chloroform, 300 K, 600 MHz).

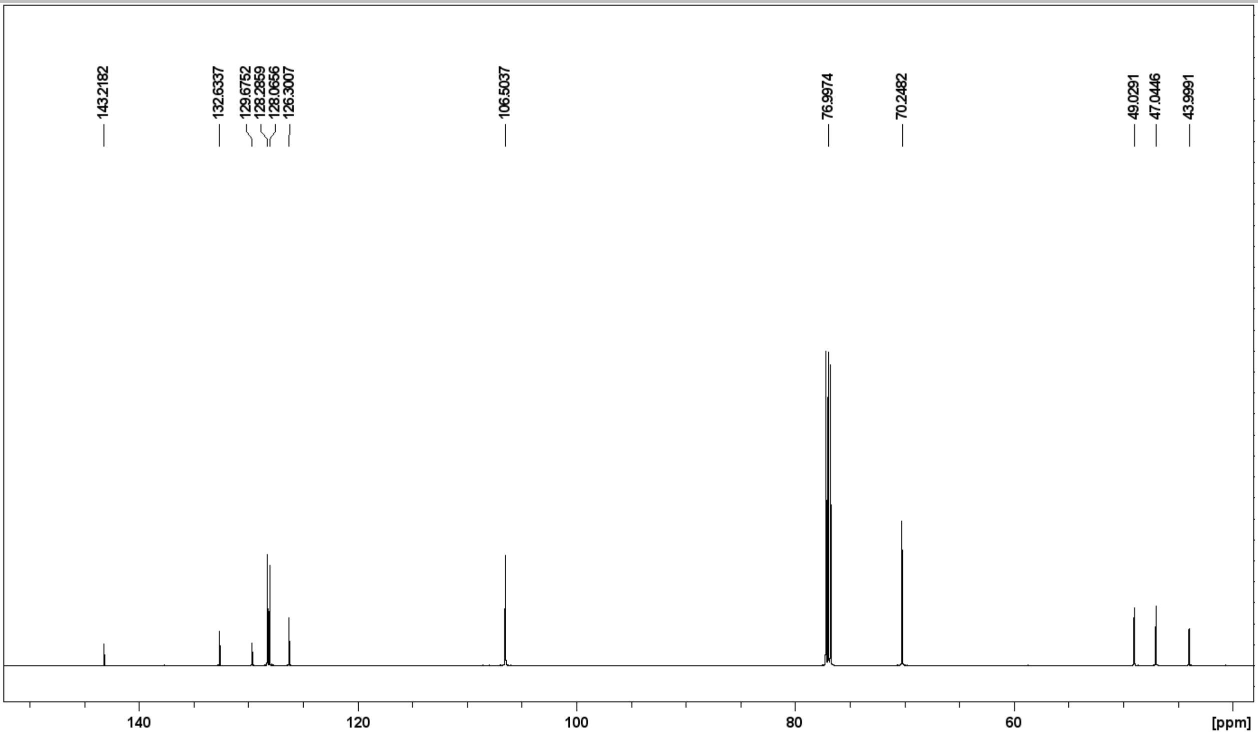

Figure S 48. The ^{13}C NMR spectrum of **14a-H$_7$** ([D]chloroform, 300 K, 151 MHz).

The NMR spectra of 14b-H

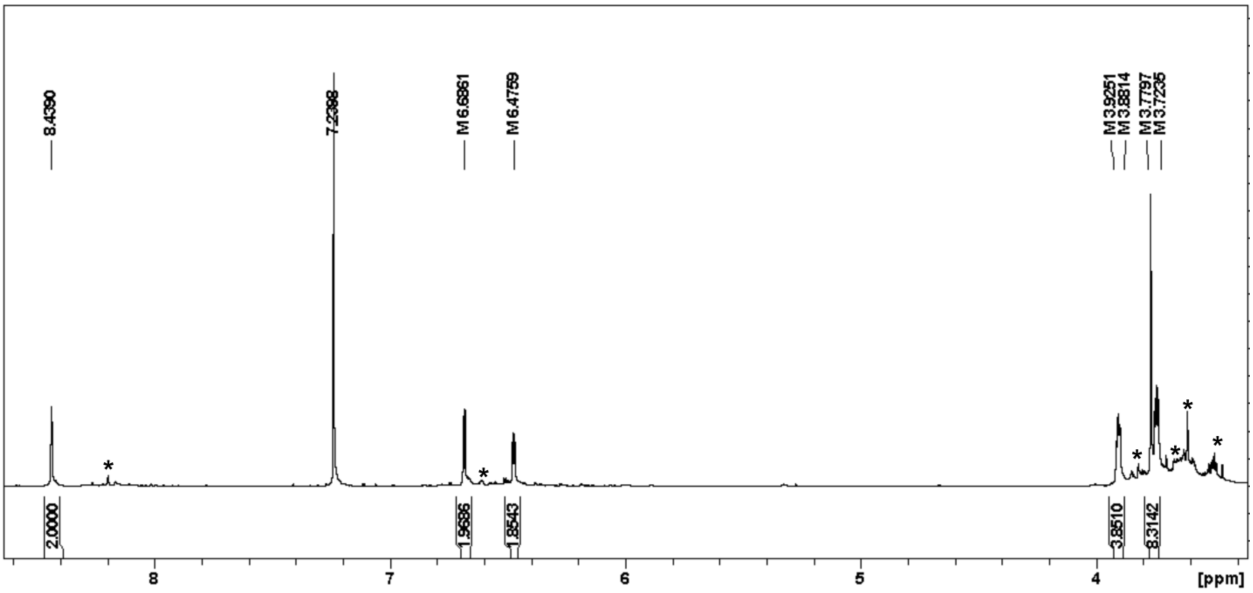

Figure S 49. The ^{1}H NMR spectrum of **14b-H** ([D]chloroform, 300 K, 600 MHz). Signals corresponding to impurities were marked with asterisks.

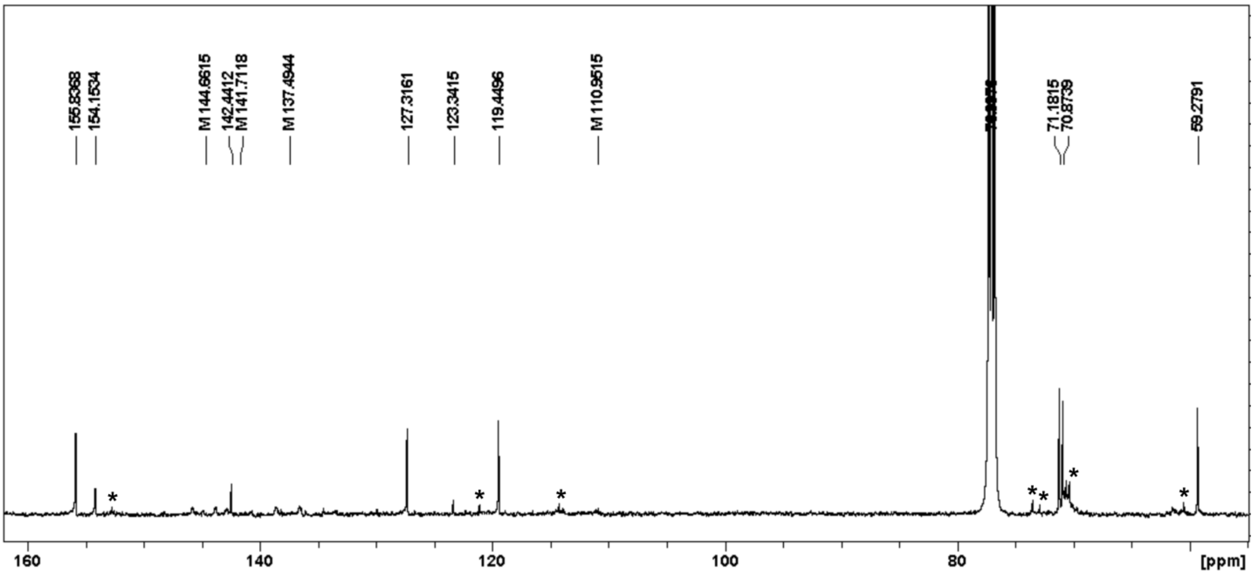

Figure S 50. The ^{13}C NMR spectrum of **14b-H** ([D]chloroform, 300 K, 125 MHz). Signals corresponding to impurities were marked with asterisks.

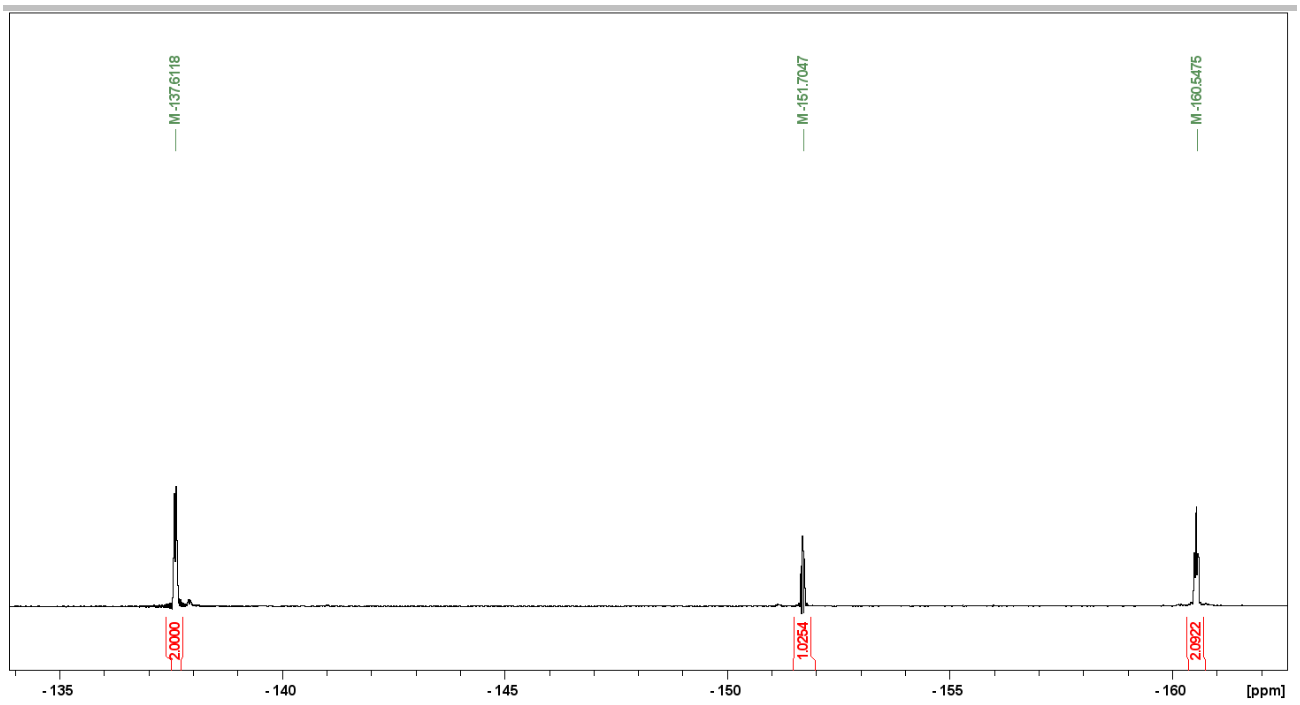

Figure S 51. The ^{19}F NMR spectrum of **14b-H** ([D]chloroform, 300 K, 471 MHz).

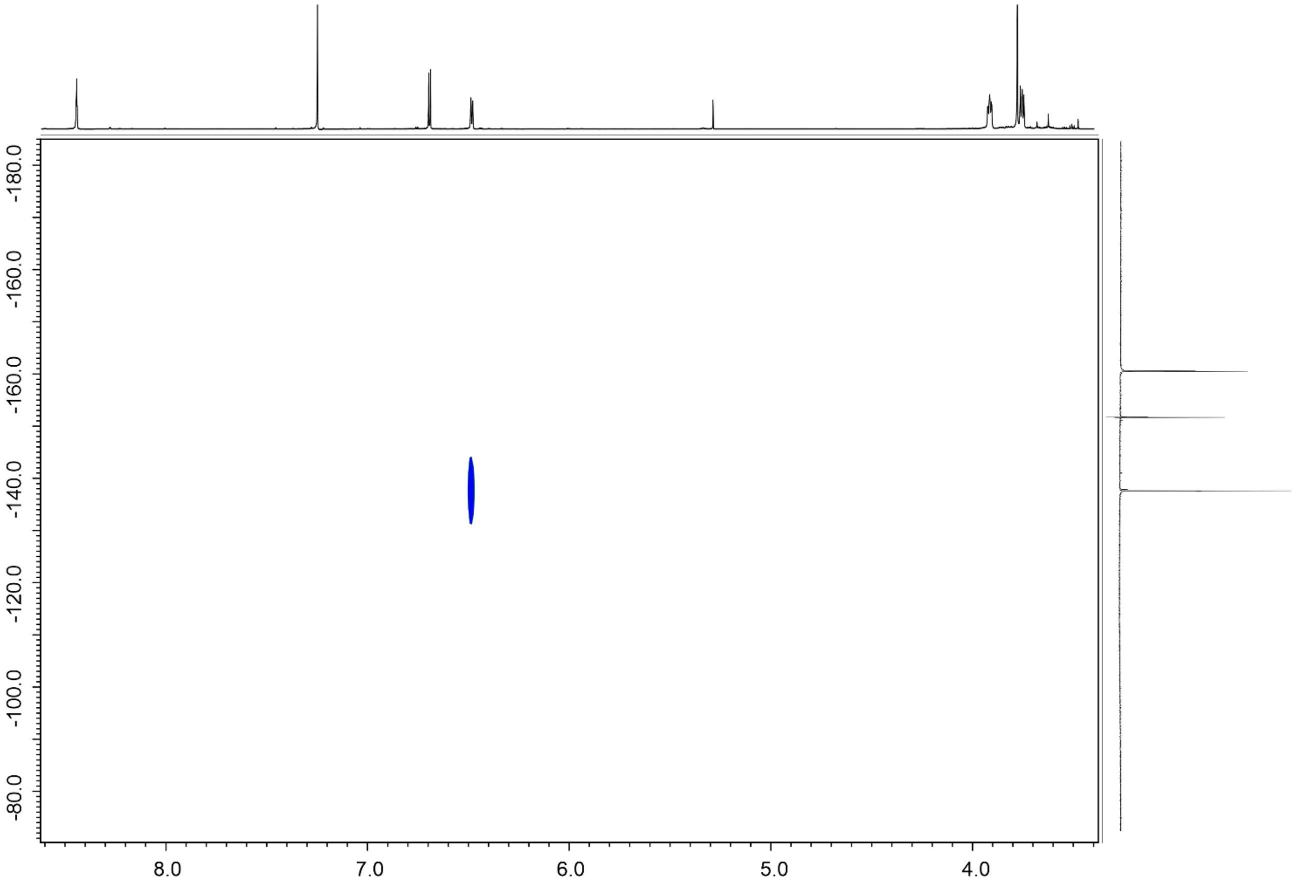

Figure S 52. The ^{1}H-^{19}F HOESY spectrum of **14b-H** ([D]chloroform, 300 K, 500 MHz).

The NMR spectra of 15a-H$_7$

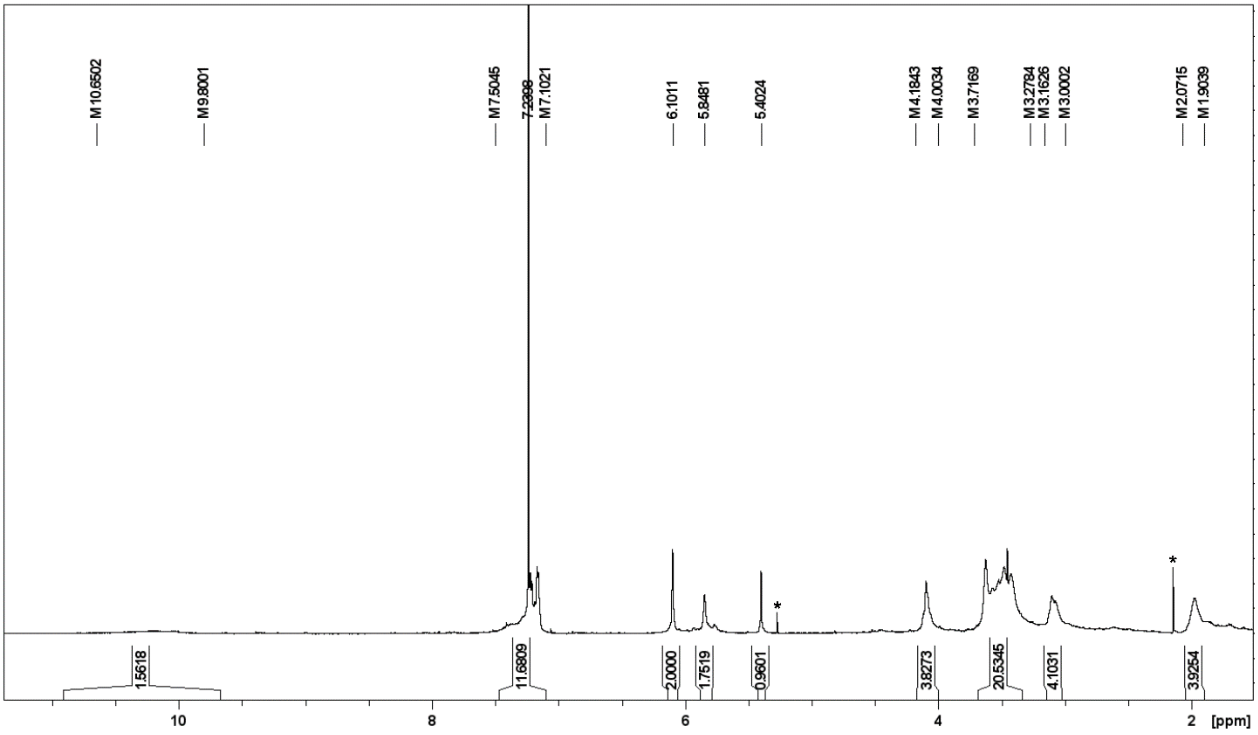

Figure S 53. The ^{1}H NMR spectrum of **15a-H$_7$** ([D]chloroform, 300 K, 600 MHz). Signals corresponding to impurities were marked with asterisks.

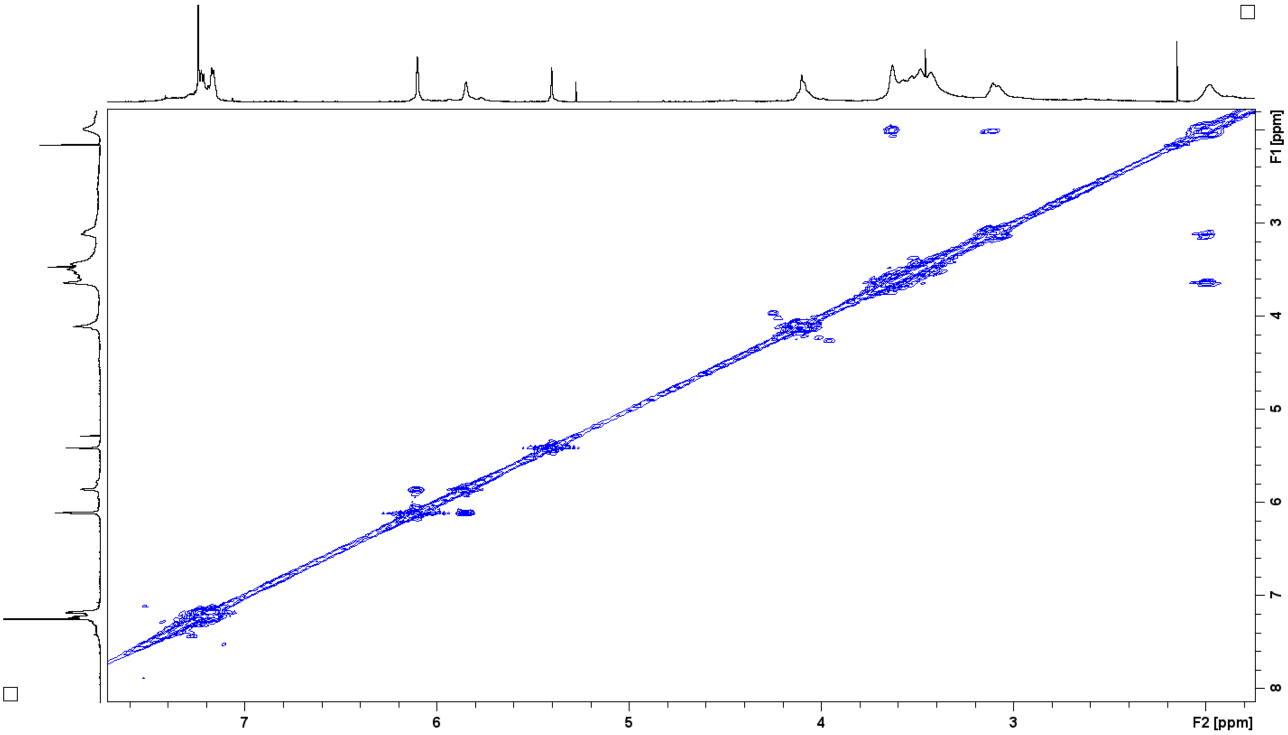

Figure S 54. The ^{1}H-^{1}H COSY spectrum of **15a-H$_7$** ([D]chloroform, 300 K, 600 MHz).

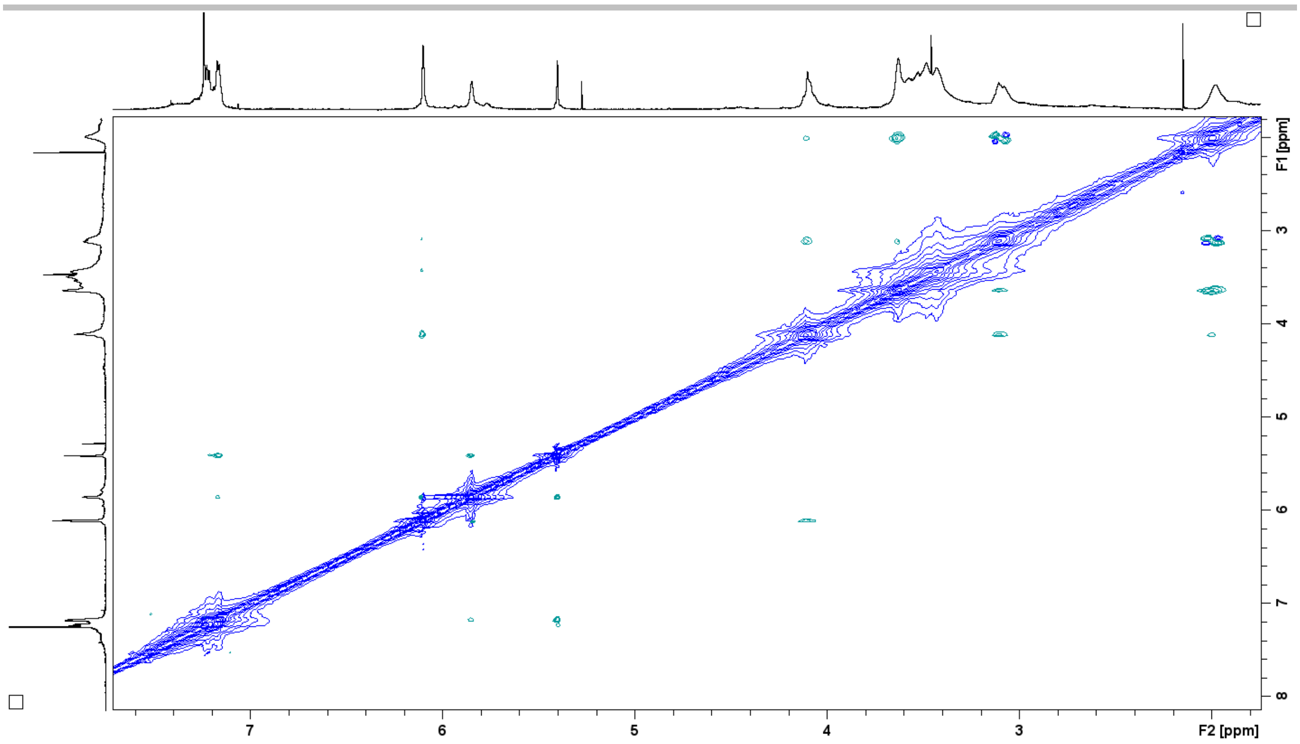

Figure S 55. The ^{1}H-^{1}H NOESY spectrum of **15a-H$_7$** ([D]chloroform, 300 K, 600 MHz).

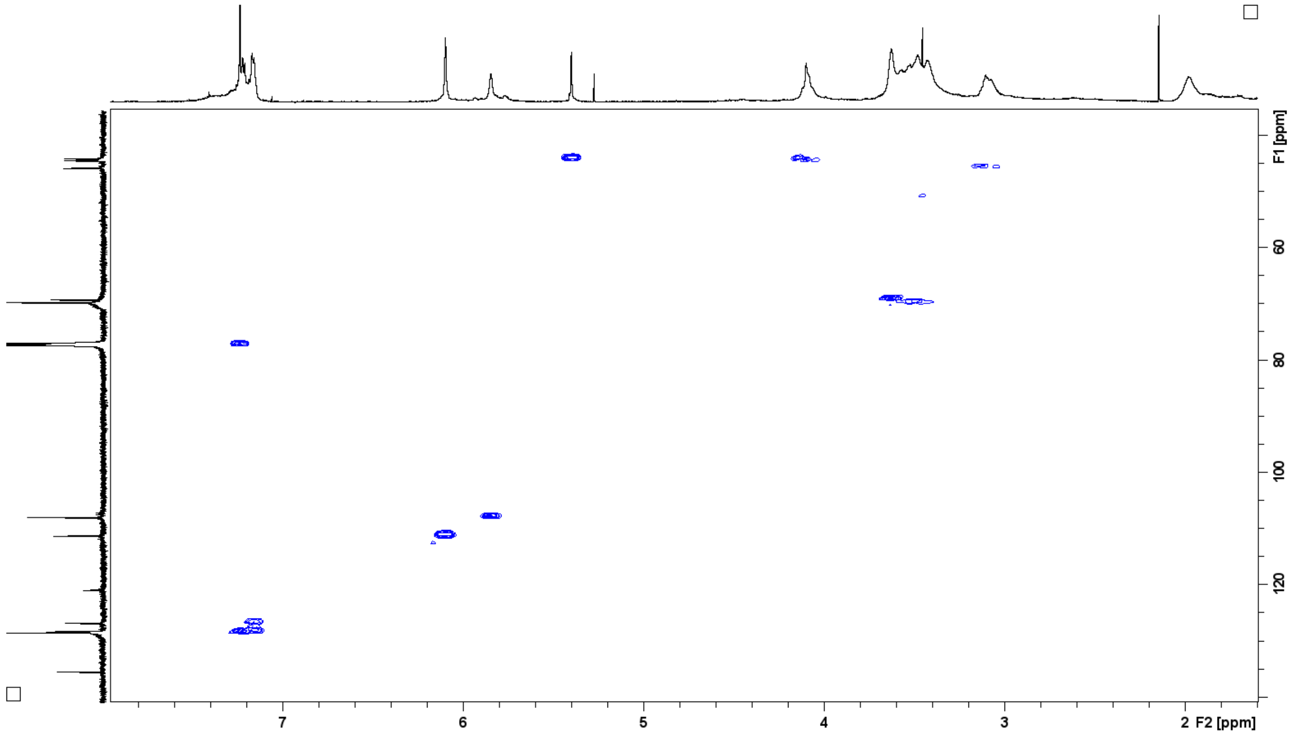

Figure S 56. The ^{1}H-^{13}C HMQC spectrum of **15a-H$_7$** ([D]chloroform, 300 K, 600 MHz).

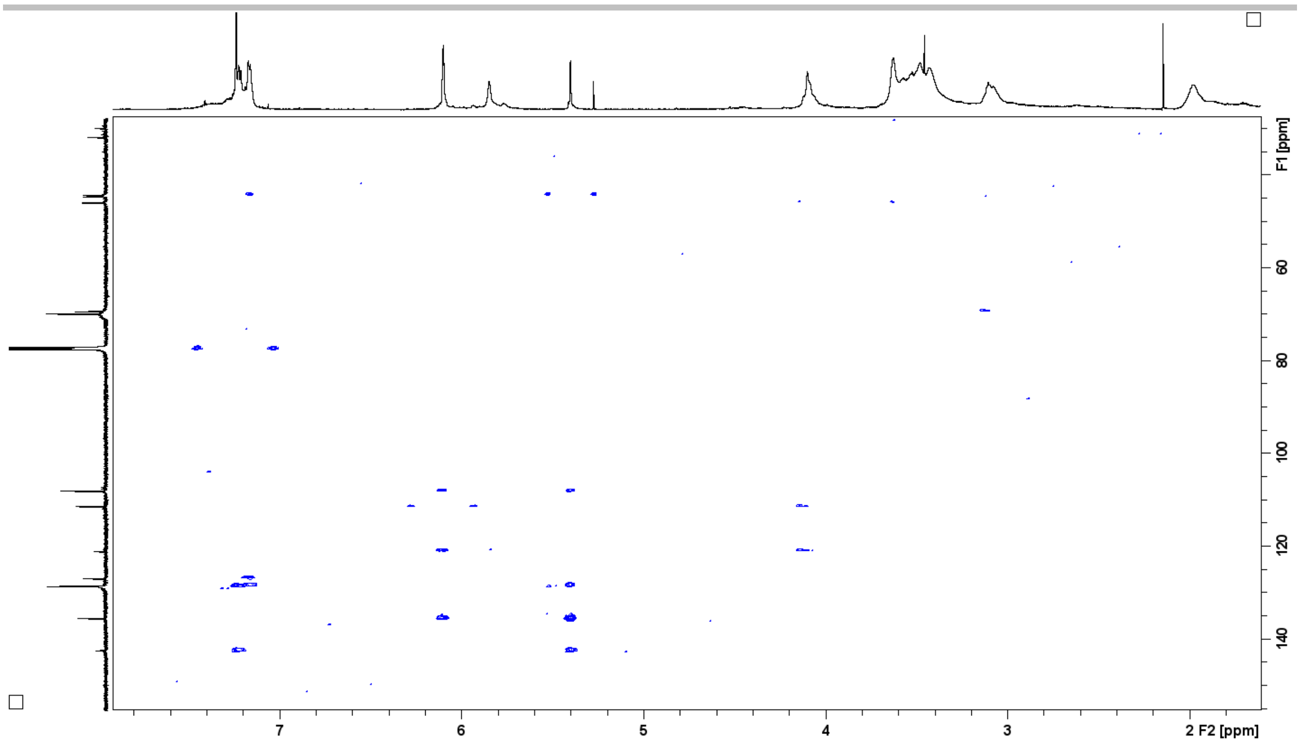

Figure S 57. The ^{1}H-^{13}C HMBC spectrum of **15a-H$_7$** ([D]chloroform, 300 K, 600 MHz).

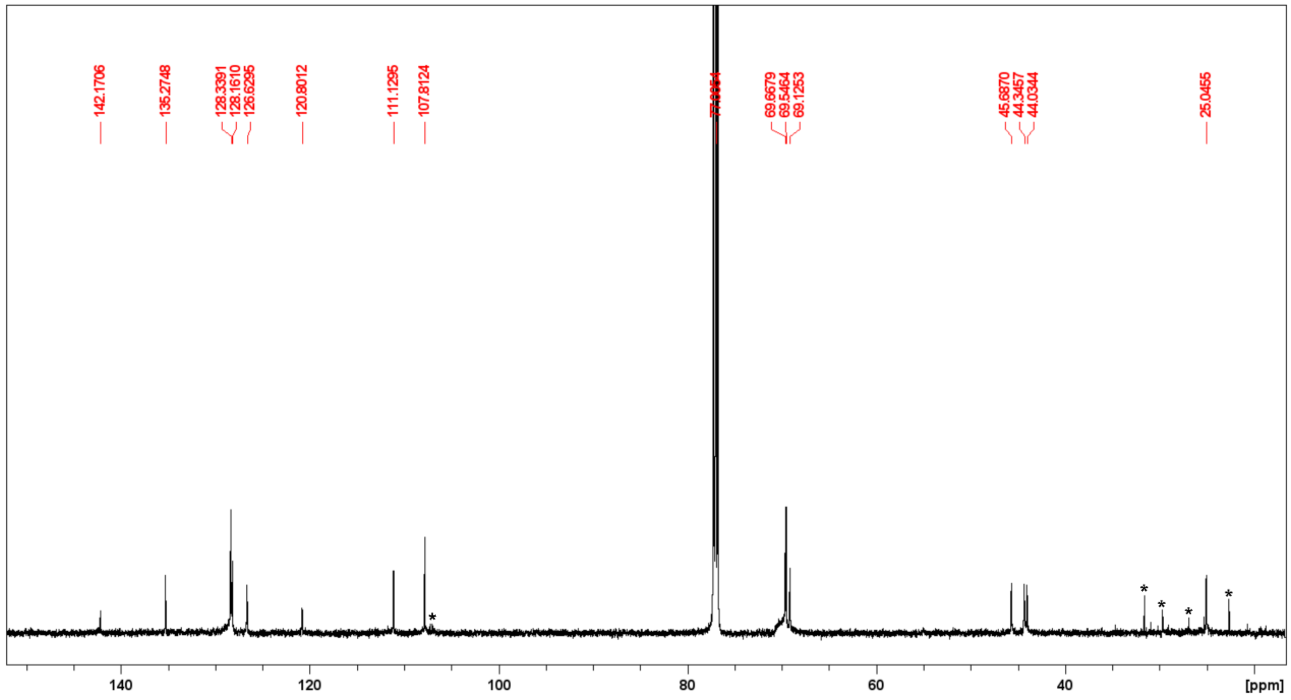

Figure S 58. The ^{13}C NMR spectrum of **15a-H$_7$** ([D]chloroform, 300 K, 151 MHz). Signals corresponding to impurities were marked with asterisks.

The NMR spectra of 15b-H

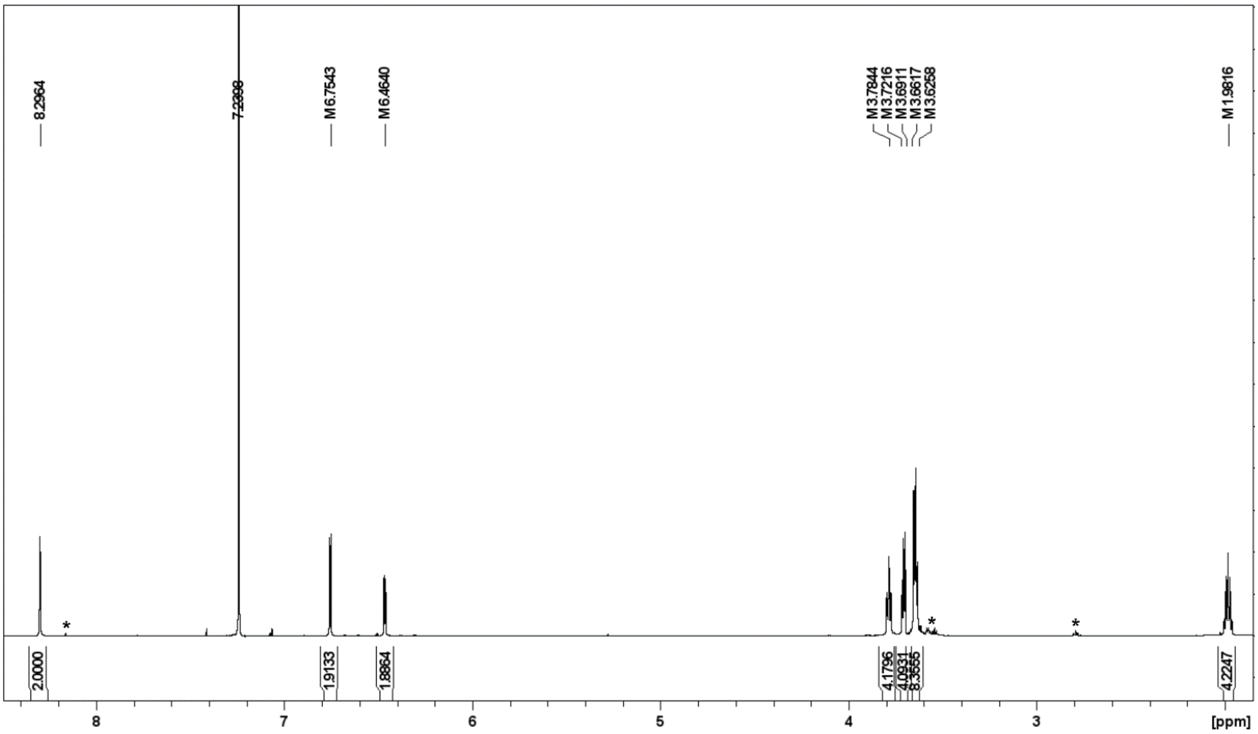

Figure S 59. The ^{1}H NMR spectrum of **15b-H** ([D]chloroform, 300 K, 600 MHz). Signals corresponding to impurities were marked with asterisks.

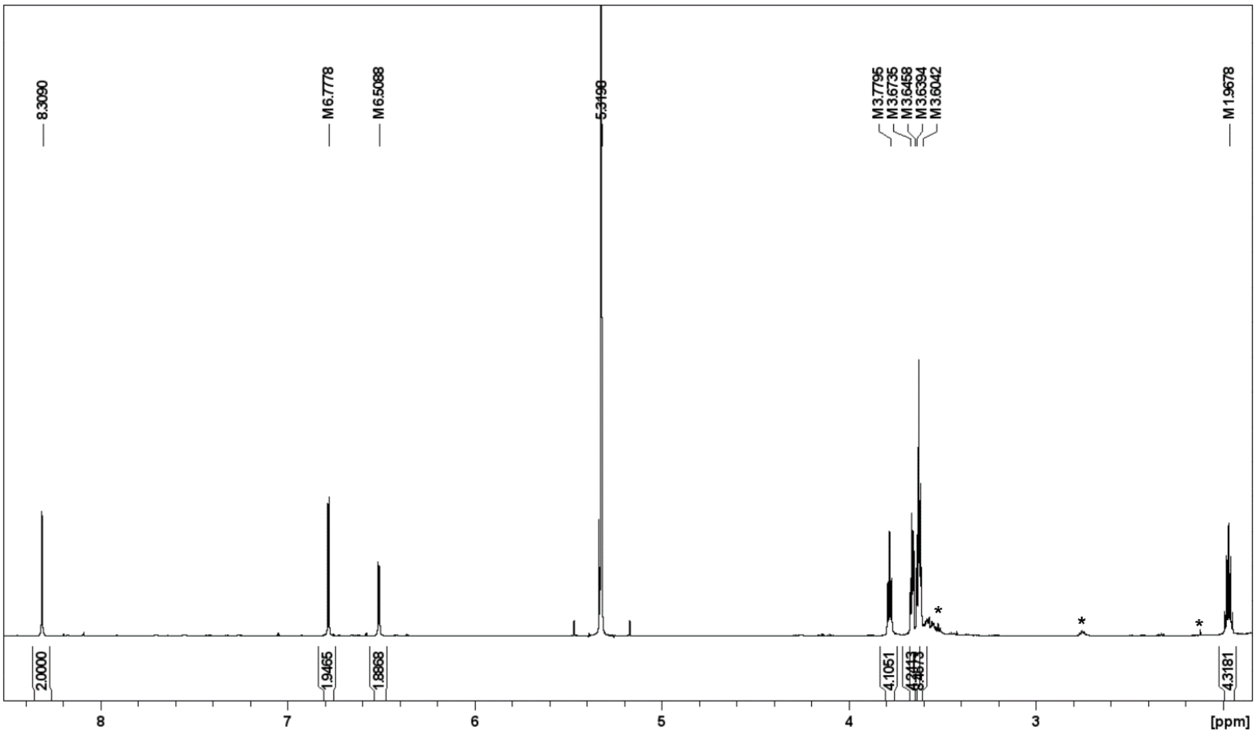

Figure S 60. The ^{1}H NMR spectrum of **15b-H** ([D_2]dichloromethane, 300 K, 600 MHz). Signals corresponding to impurities were marked with asterisks.

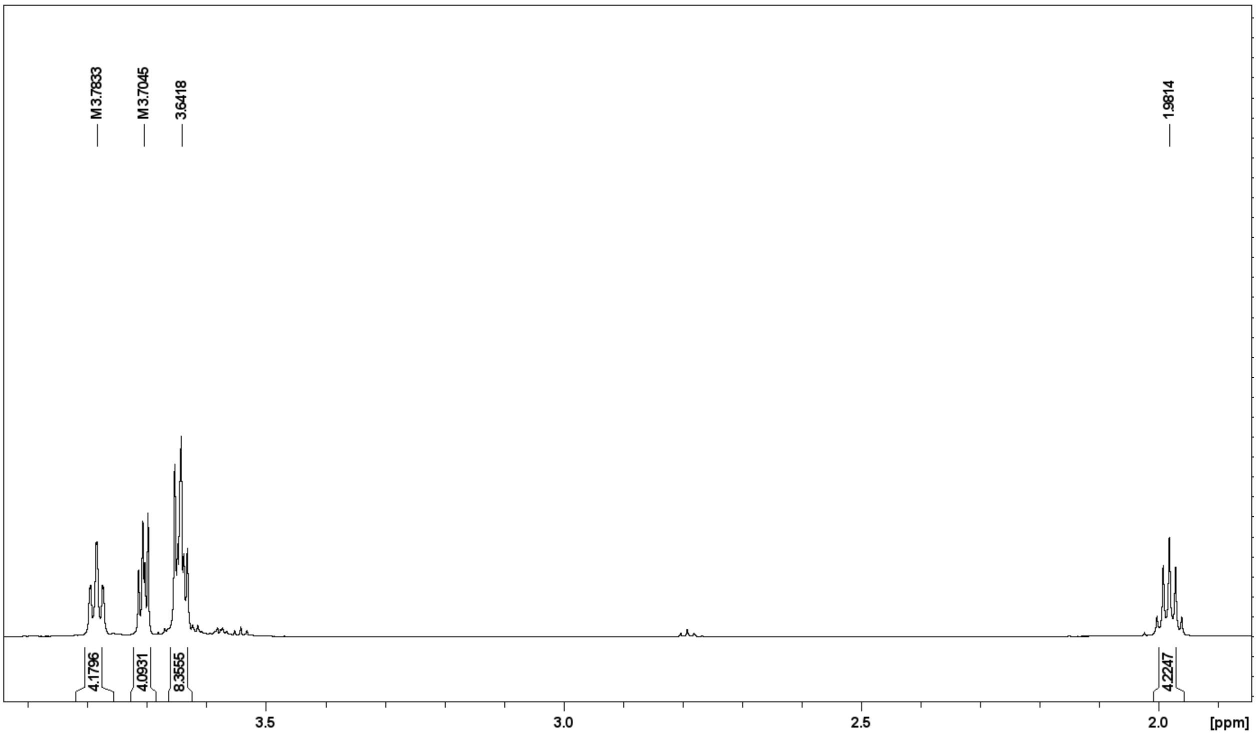

Figure S 61. The oligo(ethylene glycol) chain region of the ^{1}H NMR spectrum of **15b-H** ([D]chloroform, 300 K, 600 MHz).

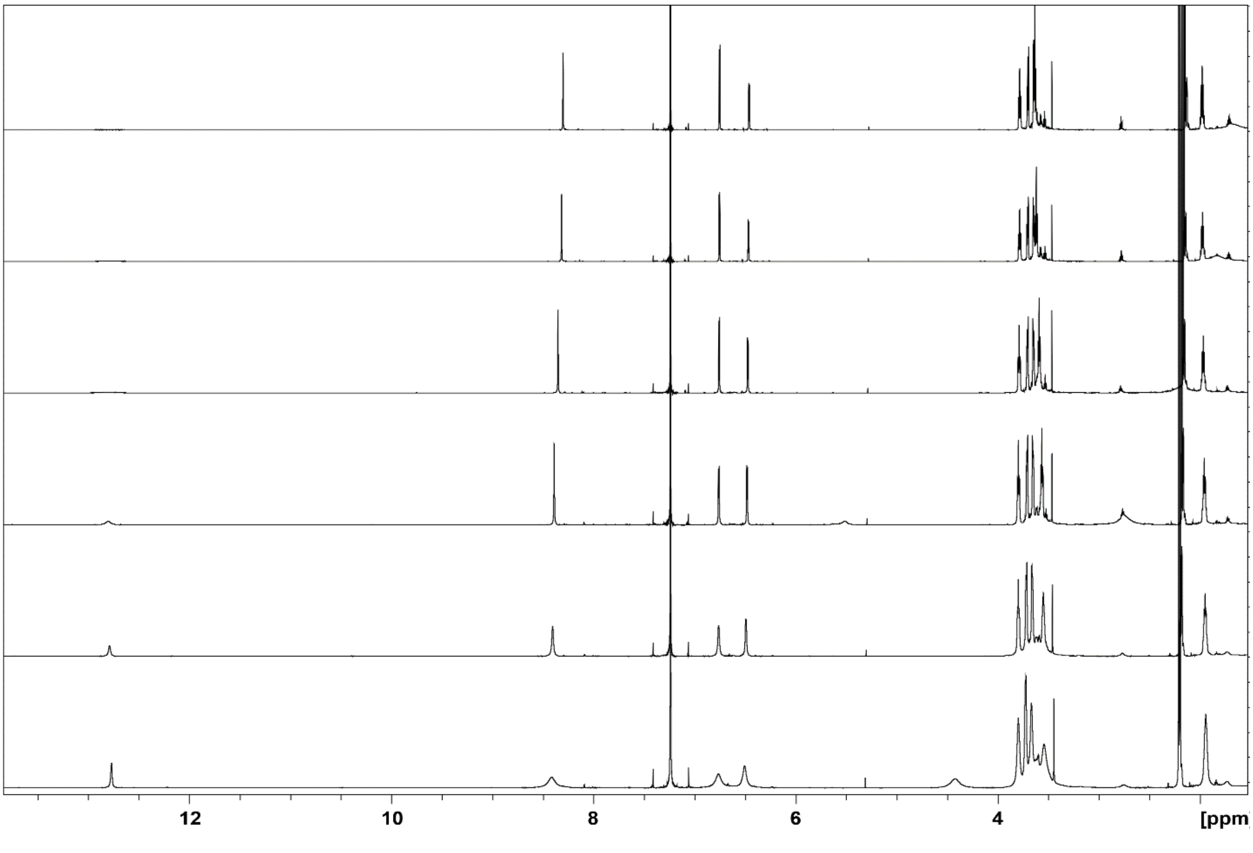

Figure S 62. ^{1}H NMR spectra of **15b-H** recorded in 300 K (top) – 200 K (bottom) temperature range every 20 K ([D]chloroform, 600 MHz).

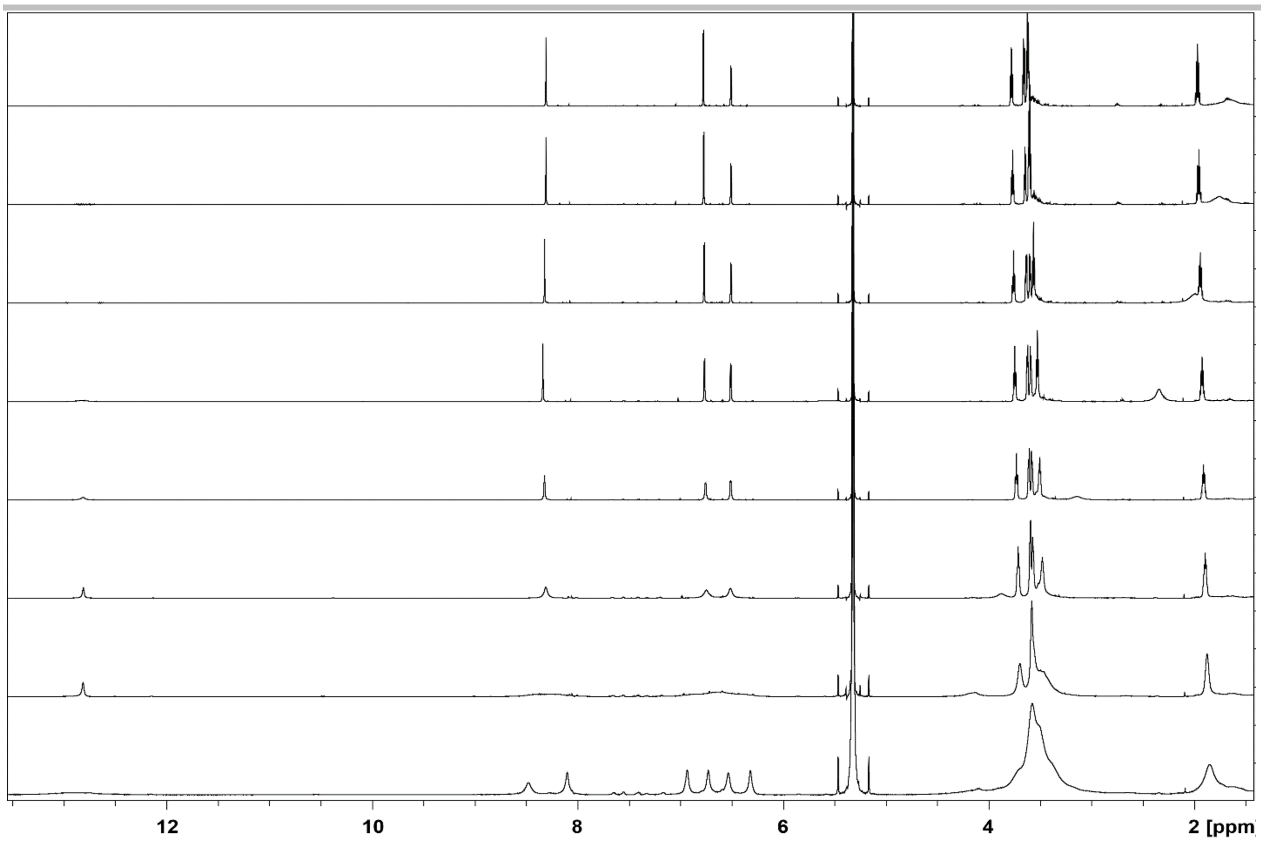

Figure S 63. ^1H NMR spectra of **15b-H** recorded in 300 K (top) – 160 K (bottom) temperature range every 20 K ([D$_2$]dichloromethane, 600 MHz).

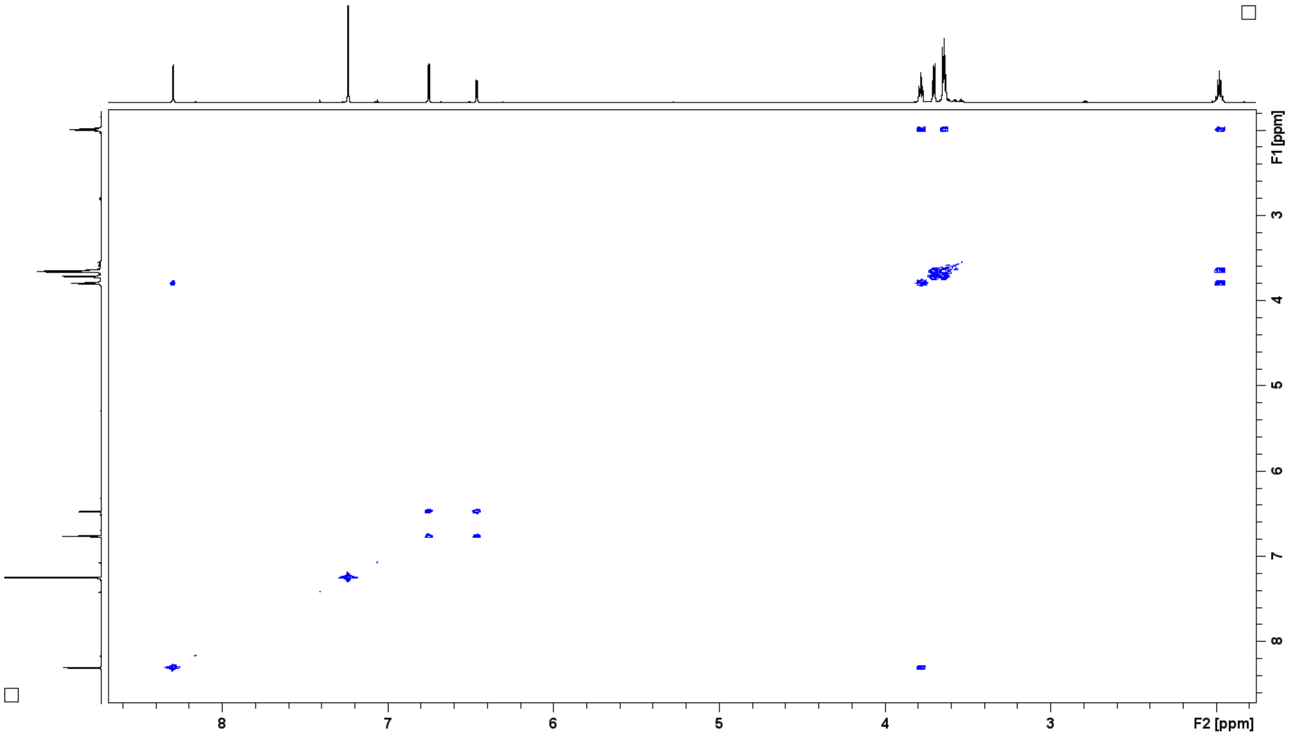

Figure S 64. The ^1H-^1H COSY spectrum of **15b-H** ([D]chloroform, 300 K, 600 MHz).

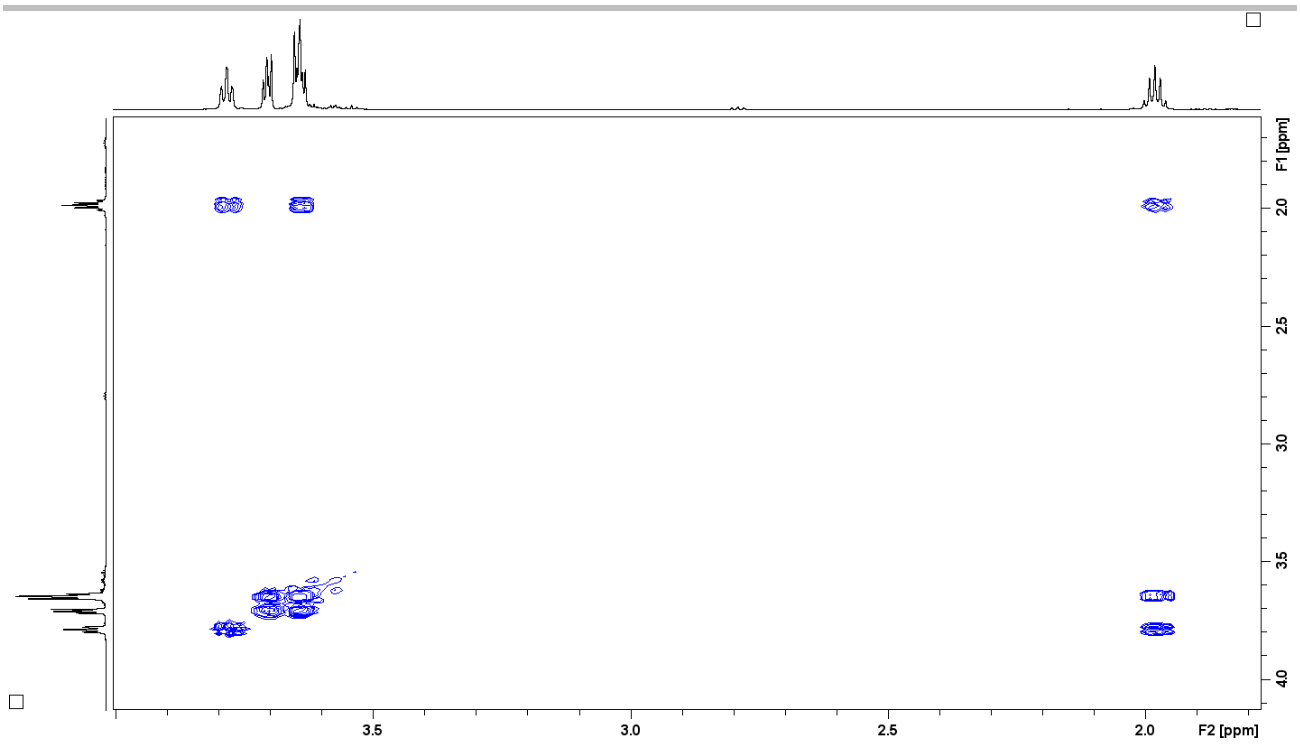

Figure S 65. The oligo(ethylene glycol) chain region of the ^{1}H-^{1}H COSY spectrum of **15b-H** ([D]chloroform, 300 K, 600 MHz).

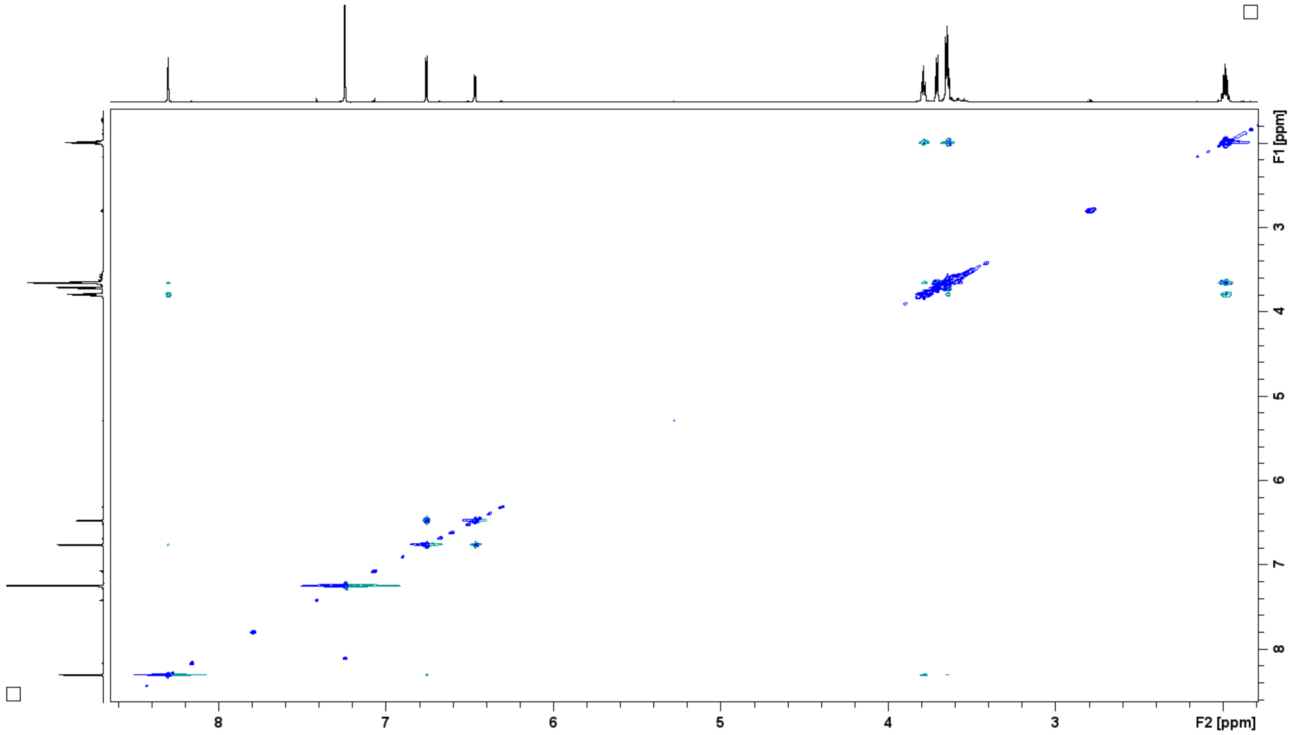

Figure S 66. The ^{1}H-^{1}H NOESY spectrum of **15b-H** ([D]chloroform, 300 K, 600 MHz).

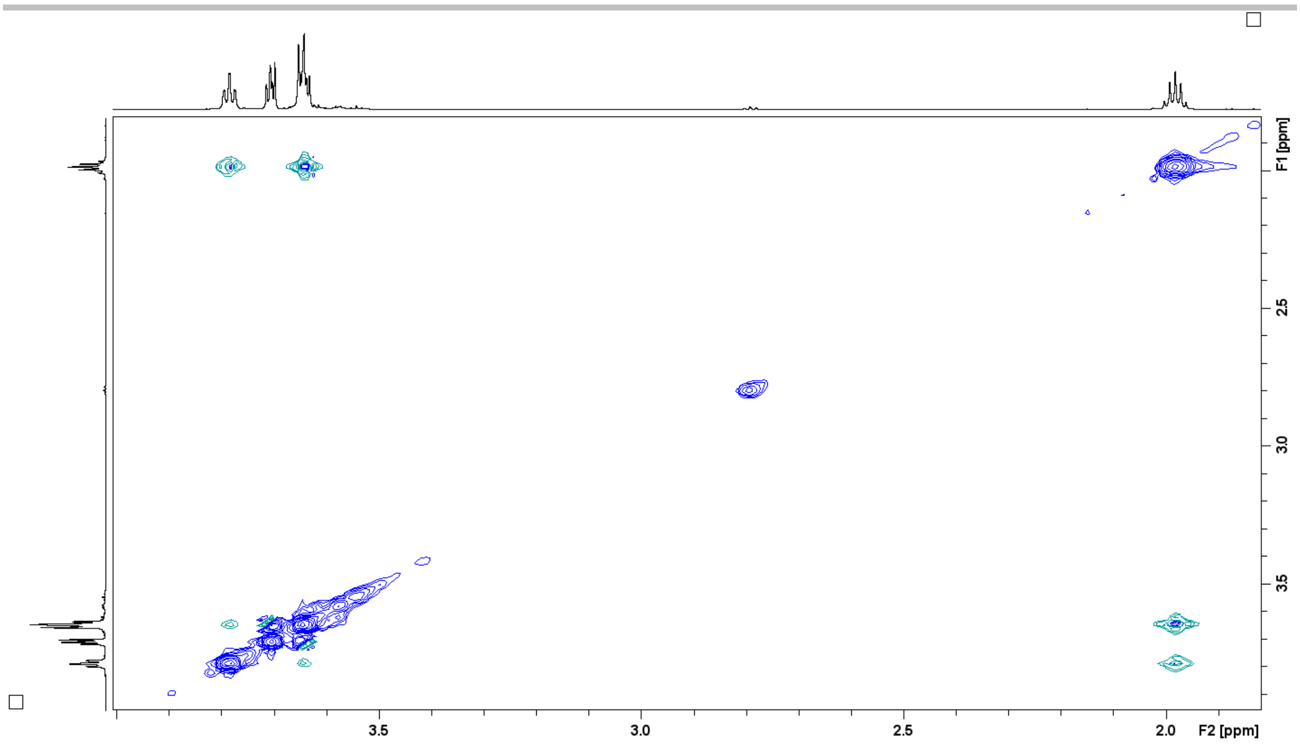

Figure S 67. The oligo(ethylene glycol) chain region of the NOESY spectrum of **15b-H** ([D]chloroform, 300 K, 600 MHz).

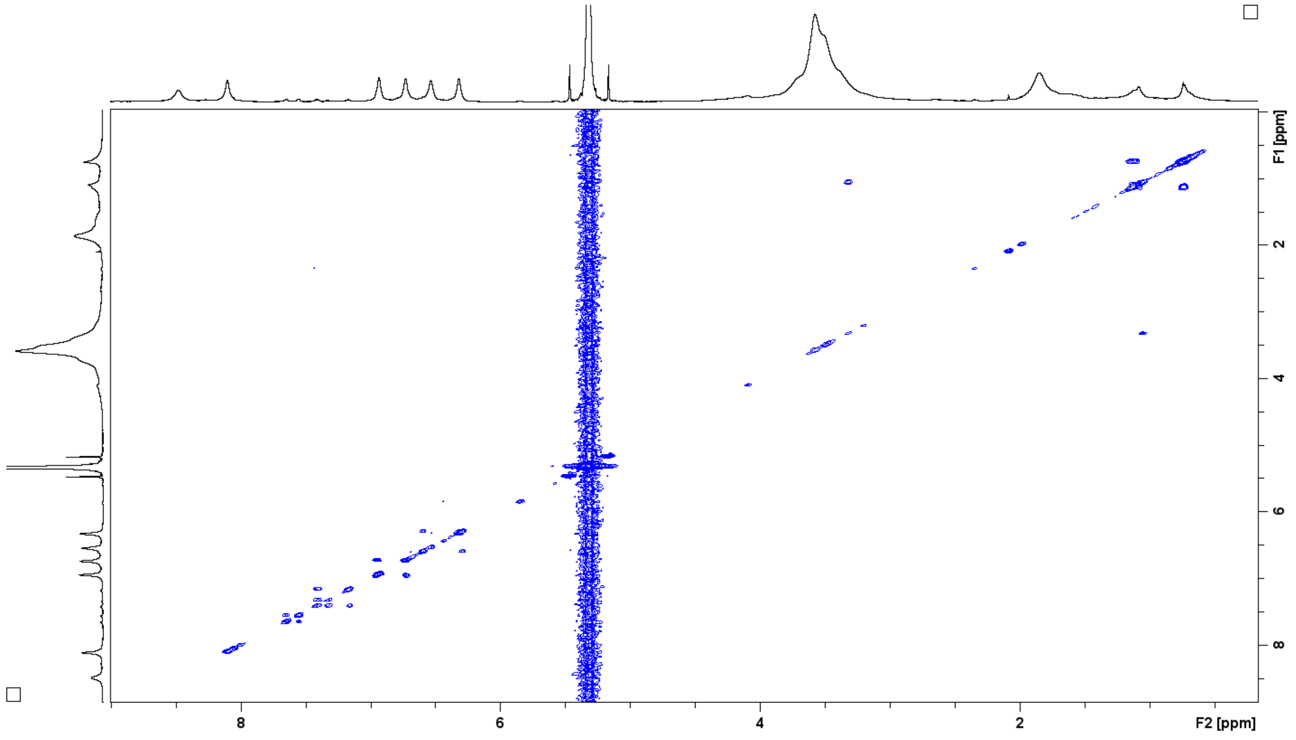

Figure S 68. The ^{1}H-^{1}H COSY spectrum of **15b-H** ([D_2]dichloromethane, 160 K, 600 MHz).

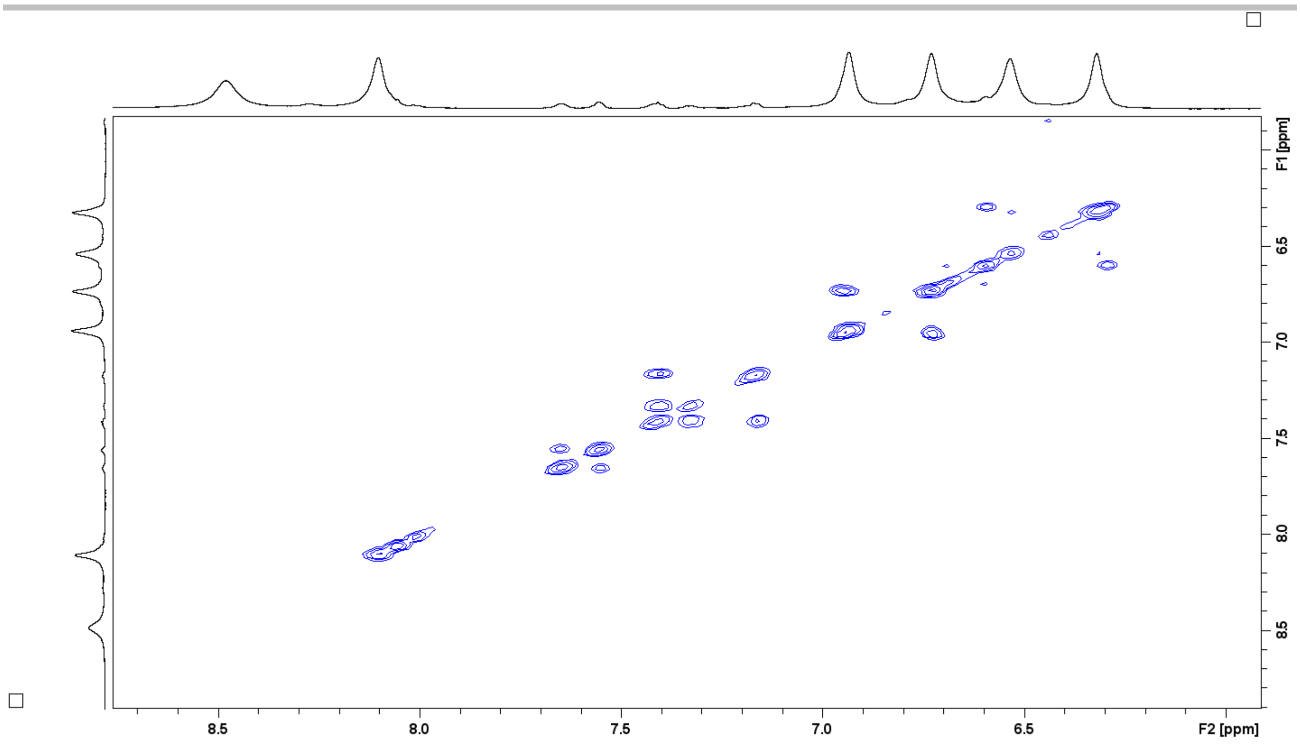

Figure S 69. The aromatic region of the ^{1}H-^{1}H COSY spectrum of **15b-H** ([D$_2$]dichloromethane, 160 K, 600 MHz).

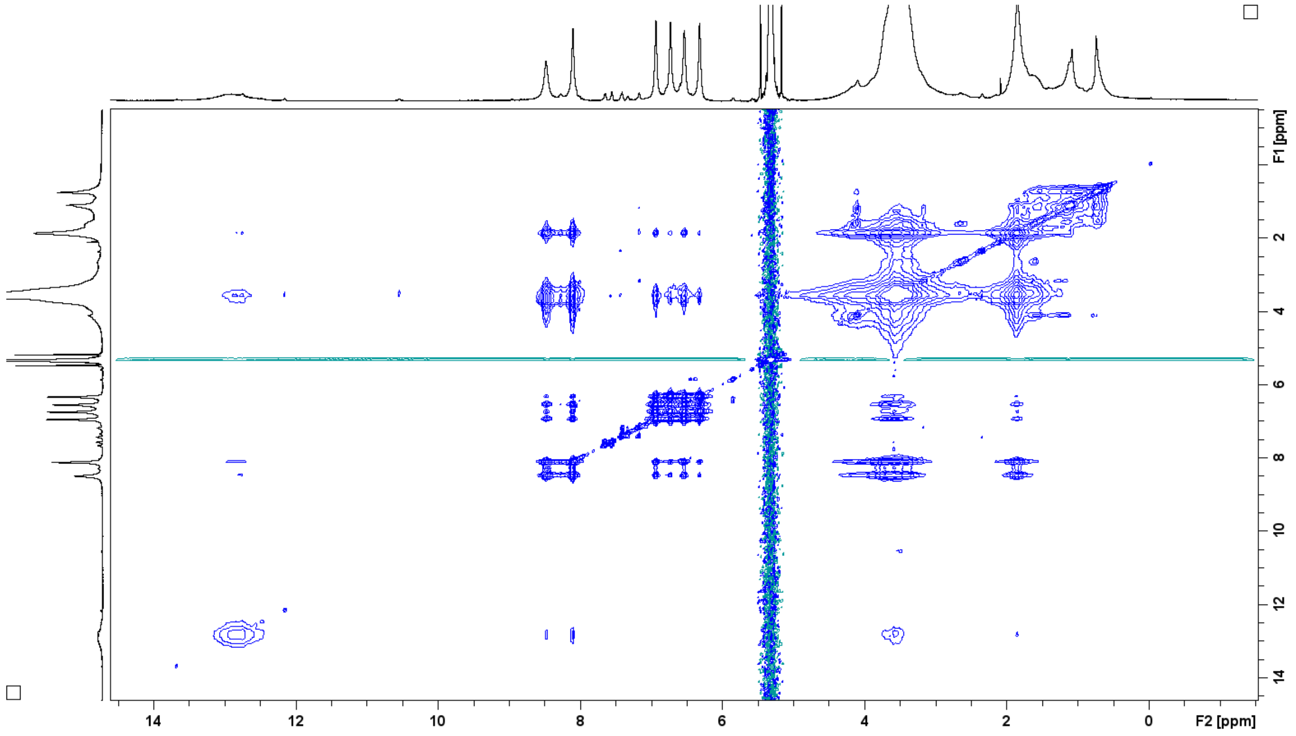

Figure S 70. The ^{1}H-^{1}H NOESY spectrum of **15b-H** ([D$_2$]dichloromethane, 160 K, 600 MHz).

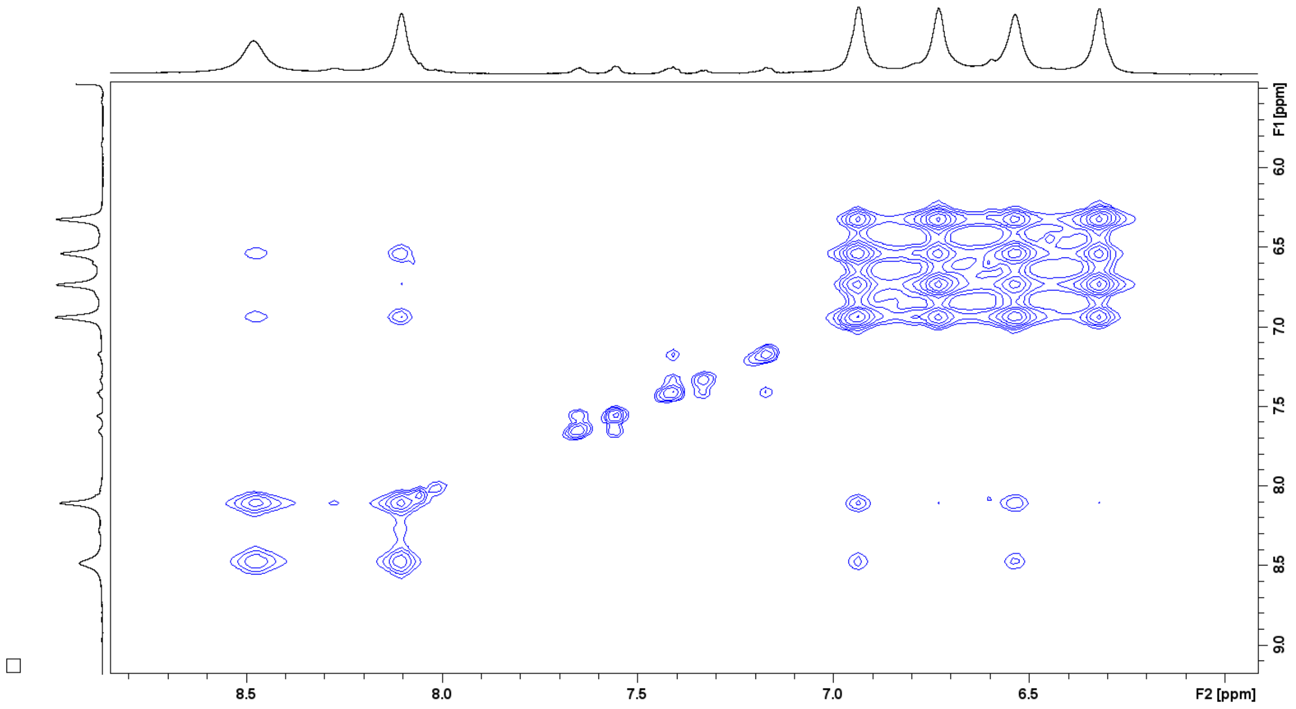

Figure S 71. The aromatic region of the ^{1}H-^{1}H COSY spectrum of **15b-H** ([D$_2$]dichloromethane, 160 K, 600 MHz).

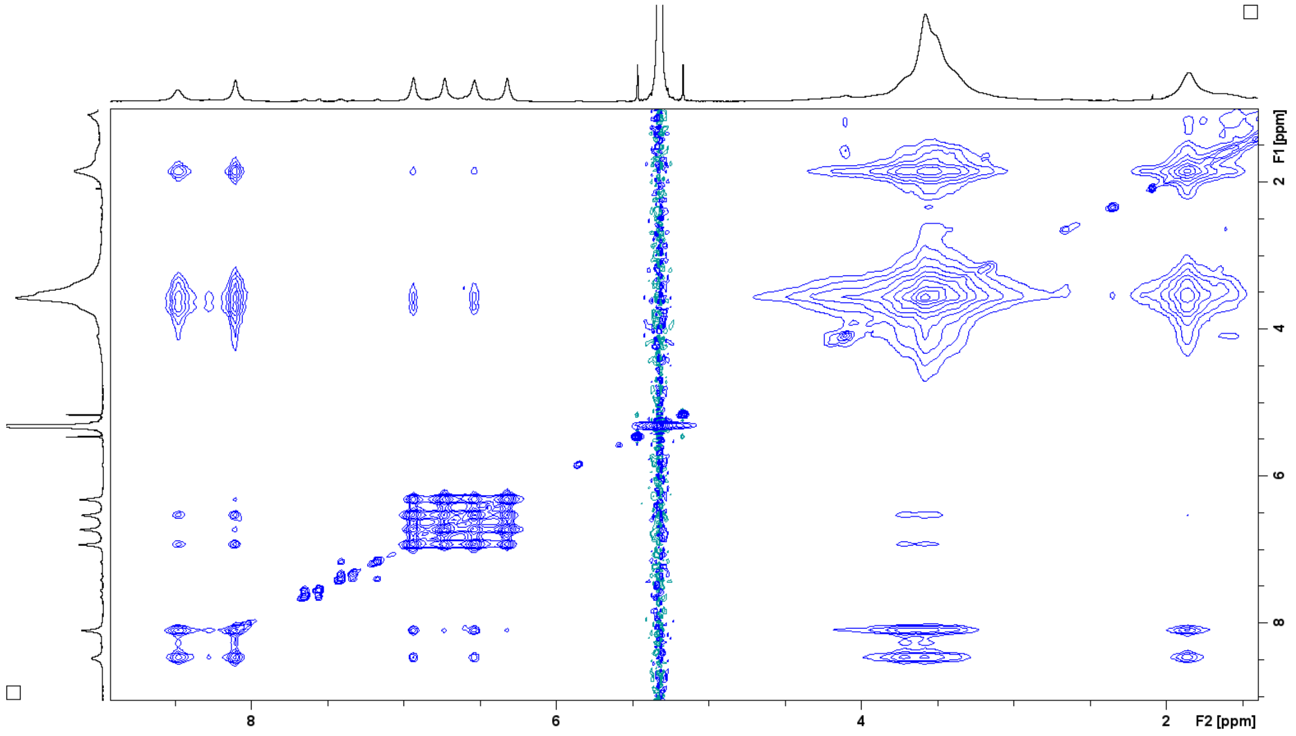

Figure S 72. The aromatic and oligo(ethylene glycol) chain regions of the ^{1}H-^{1}H COSY spectrum of **15b-H** ([D$_2$]dichloromethane, 160 K, 600 MHz).

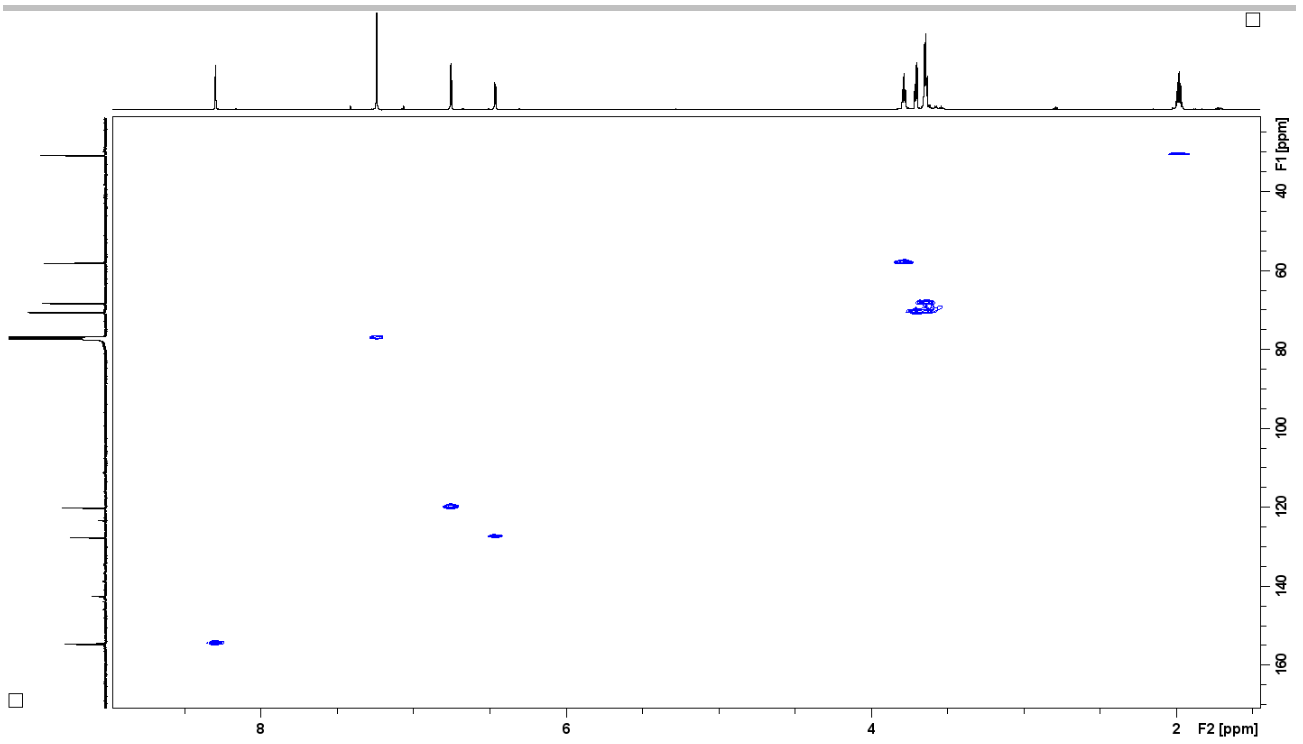

Figure S 73. The ^{1}H-^{13}C HMQC spectrum of **15b-H** ([D]chloroform, 300 K, 600 MHz).

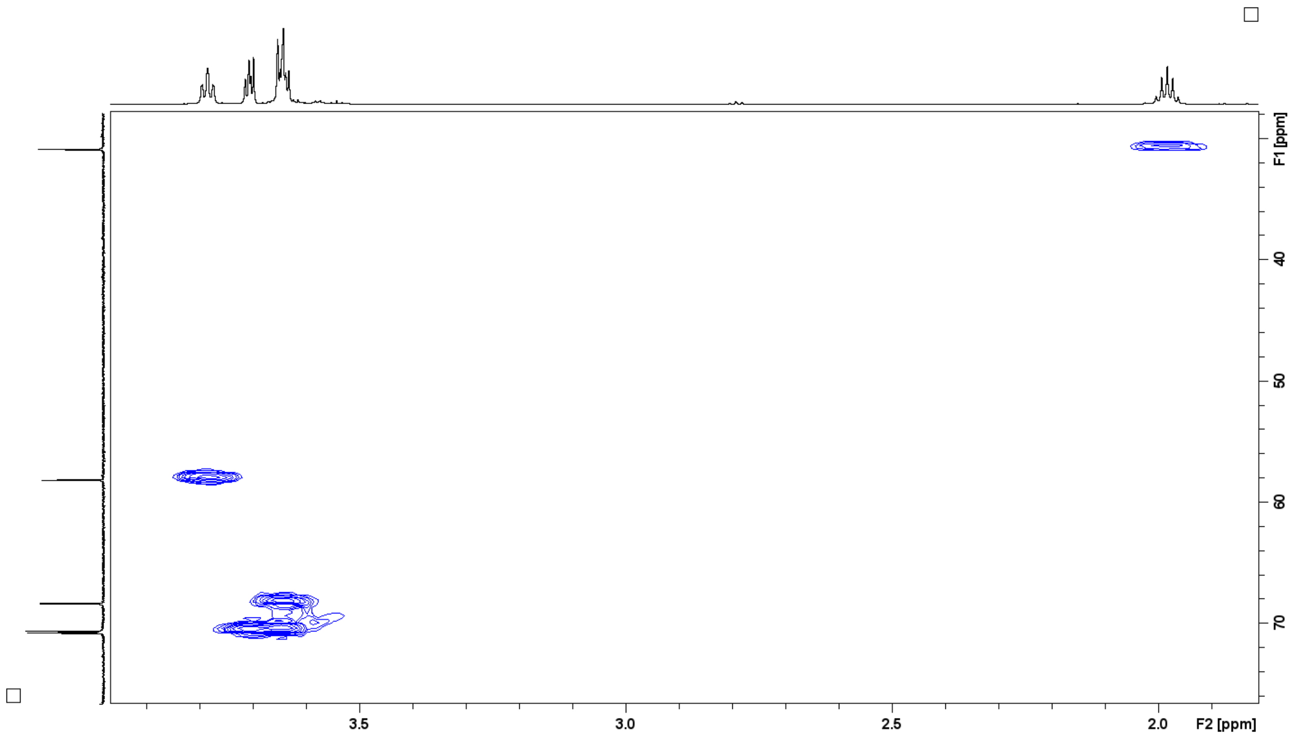

Figure S 74. The oligo(ethylene glycol) chain region of the ^{1}H-^{13}C HMQC spectrum of **15b-H** ([D]chloroform, 300 K, 600 MHz).

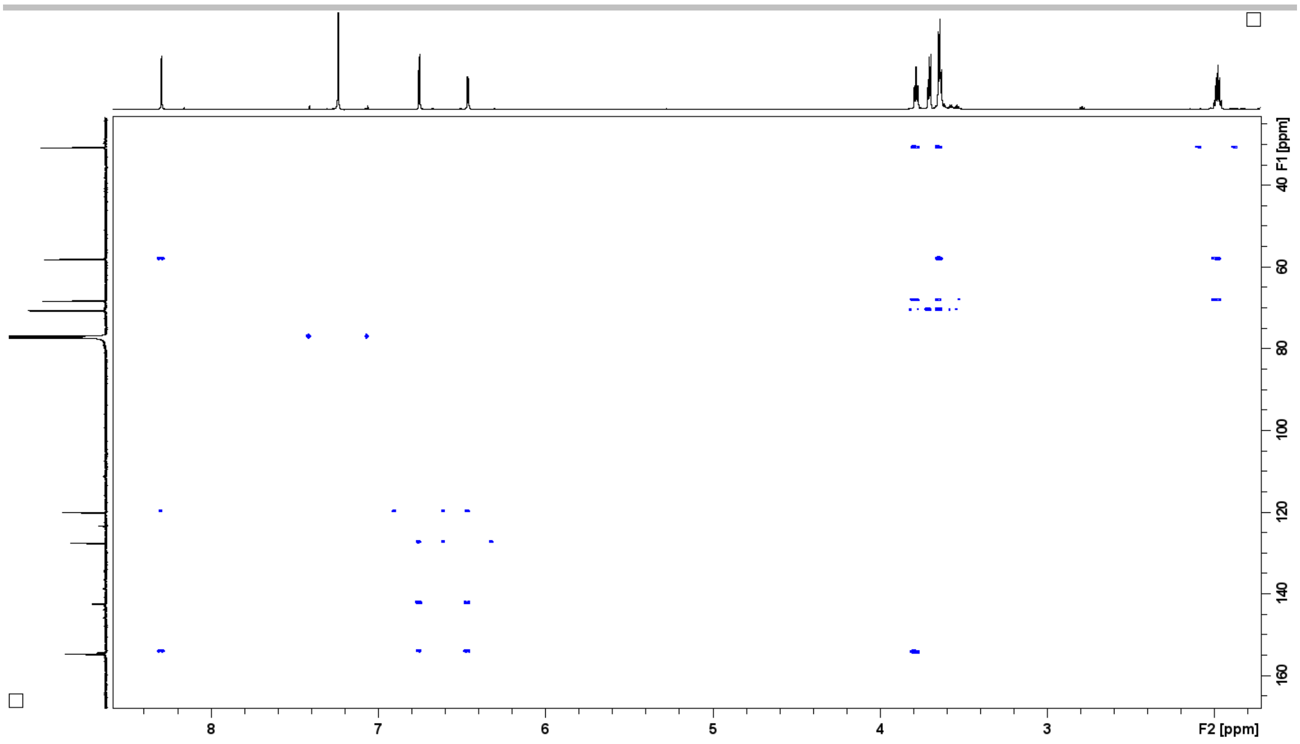

Figure S 75. The ^{1}H-^{13}C HMBC spectrum of **15b-H** ([D]chloroform, 300 K, 600 MHz).

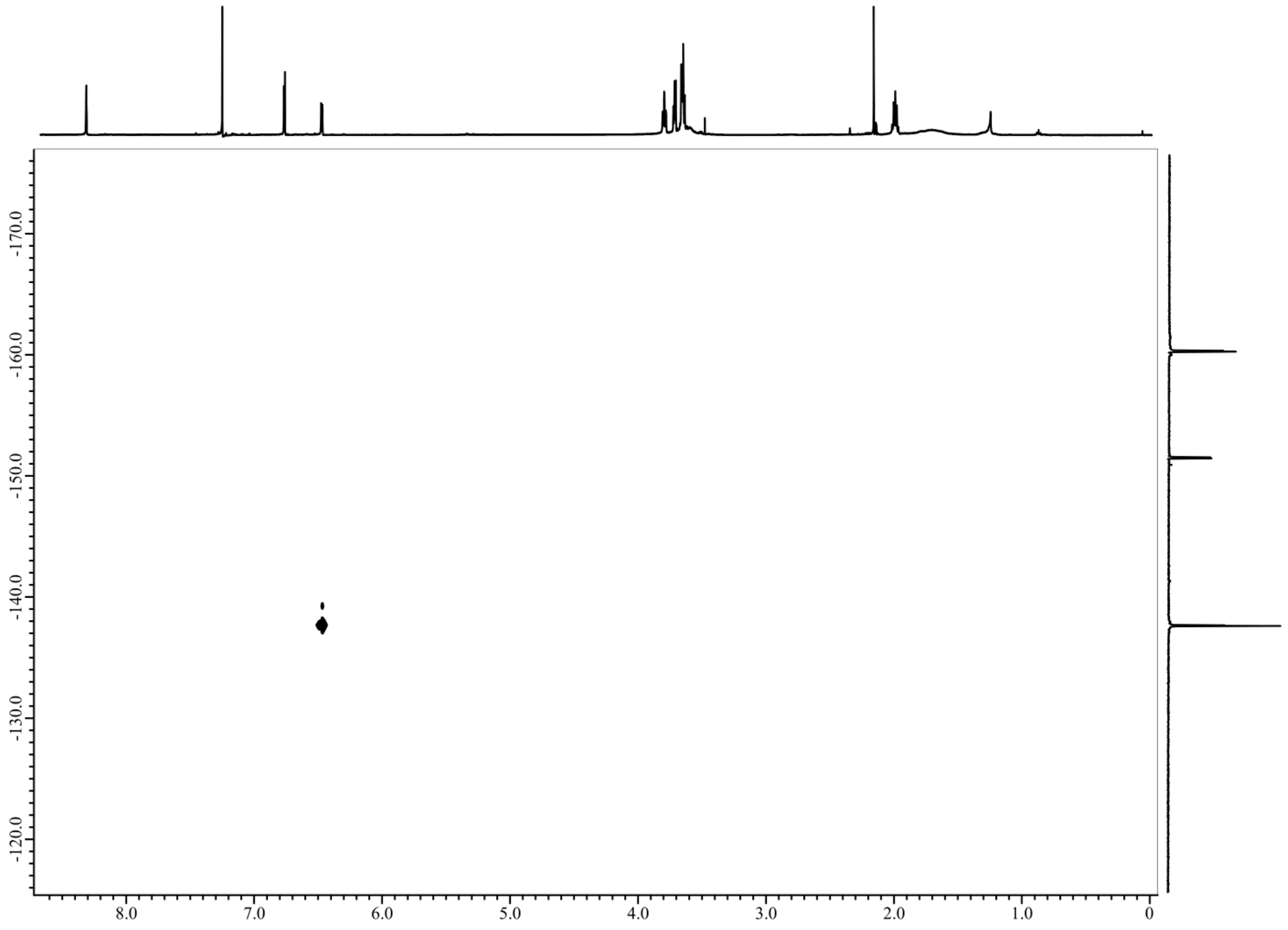

Figure S 76. The ^{1}H-^{19}F HOESY spectrum of **15b-H** ([D]chloroform, 300 K, 500 MHz).

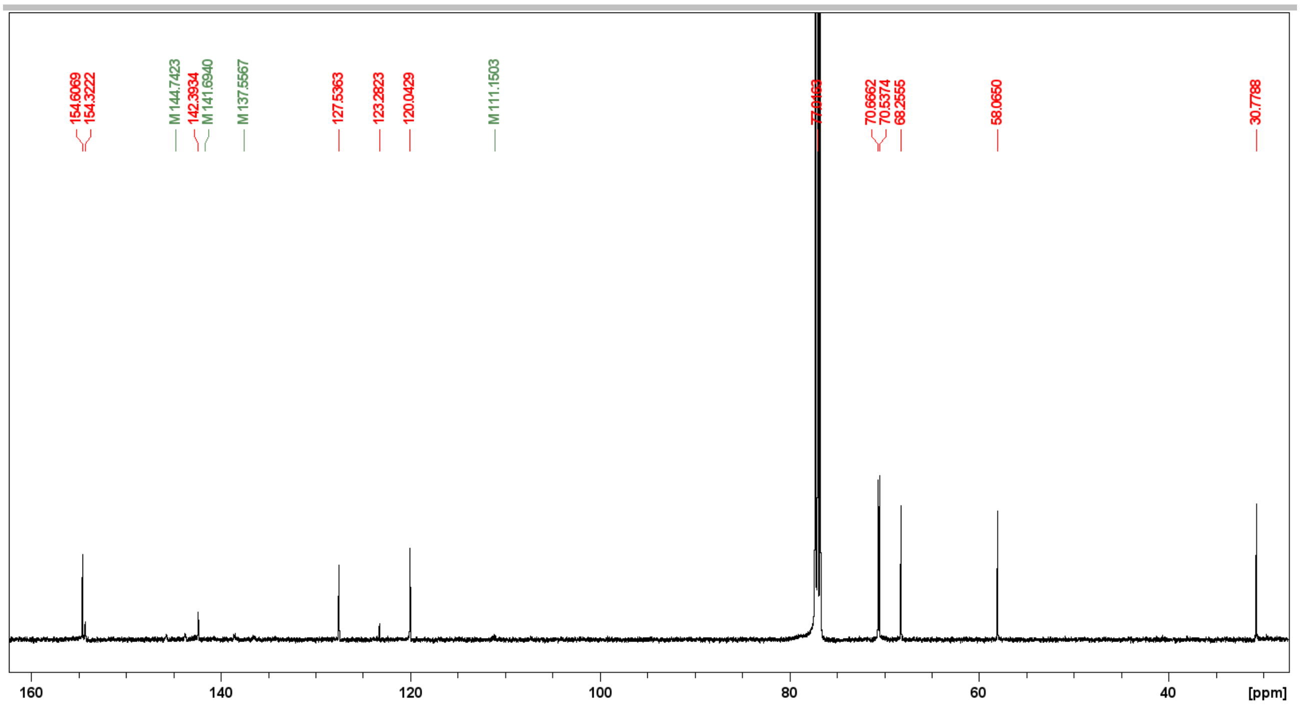

Figure S 77. The ^{13}C NMR spectrum of **15b-H** ([D]chloroform, 300 K, 151 MHz). Signals corresponding to impurities were marked with asterisks.

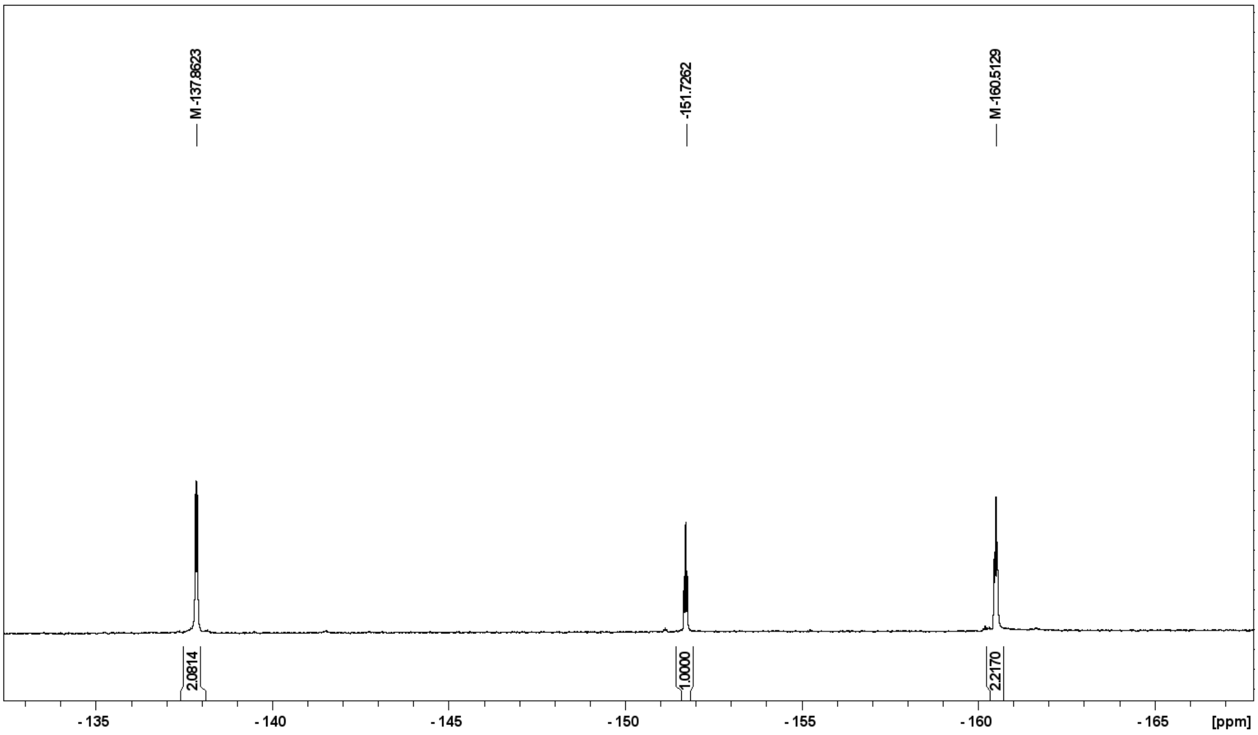

Figure S 78. The ^{19}F NMR spectrum of **15b-H** ([D]chloroform, 300 K, 471 MHz).

The NMR spectra of 16b-H

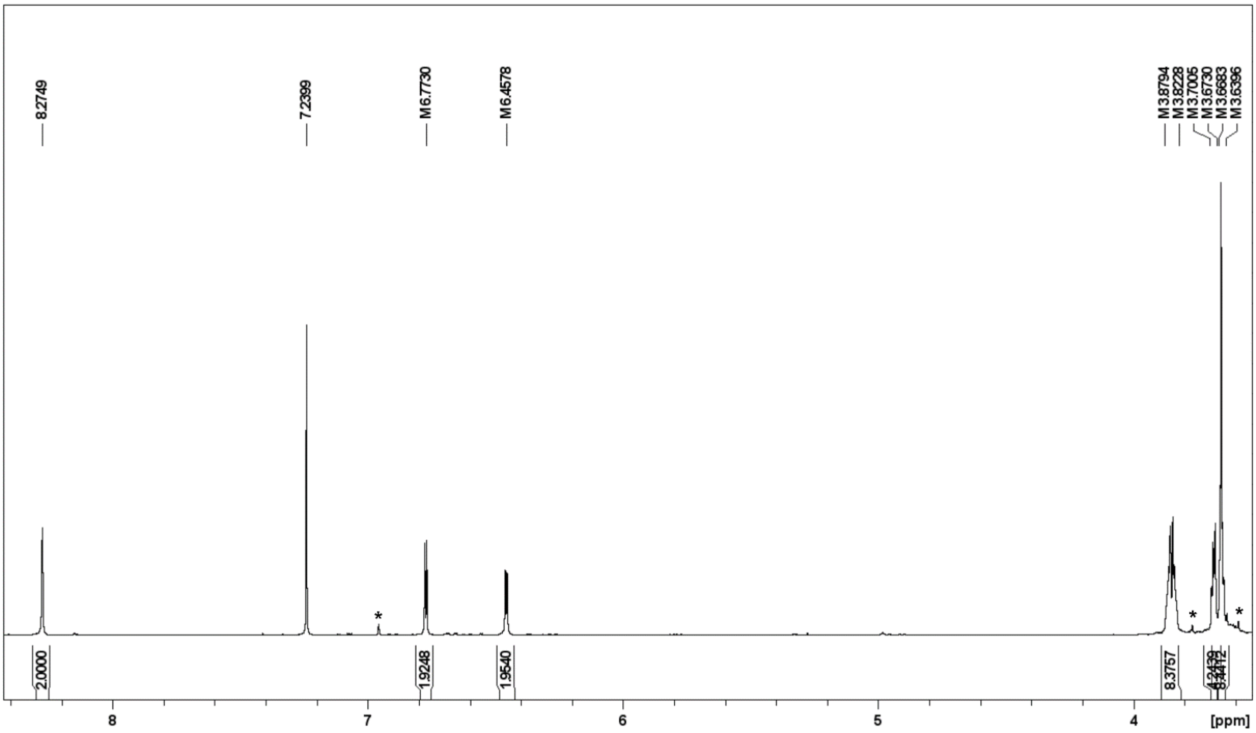

Figure S 79. The ^{1}H NMR spectrum of **16b-H** ([D]chloroform, 300 K, 600 MHz). Signals corresponding to impurities were marked with asterisks.

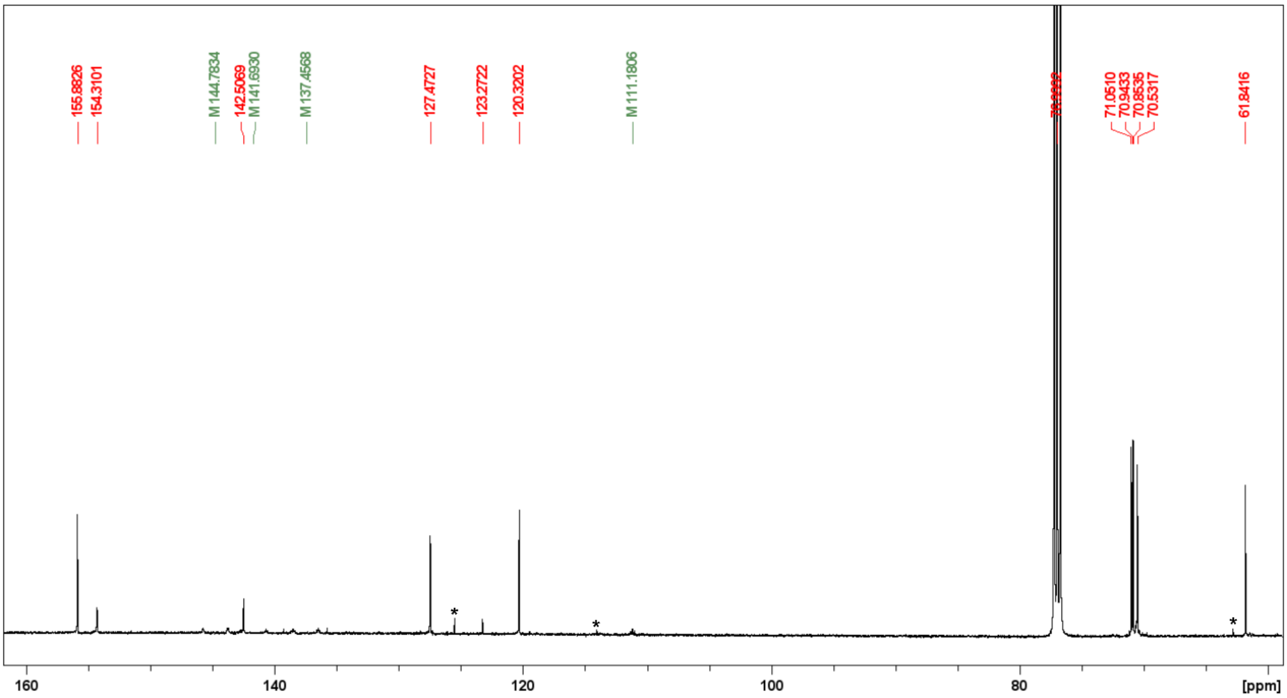

Figure S 80. The ^{13}C NMR spectrum of **16b-H** ([D]chloroform, 300 K, 125 MHz). Signals corresponding to impurities were marked with asterisks.

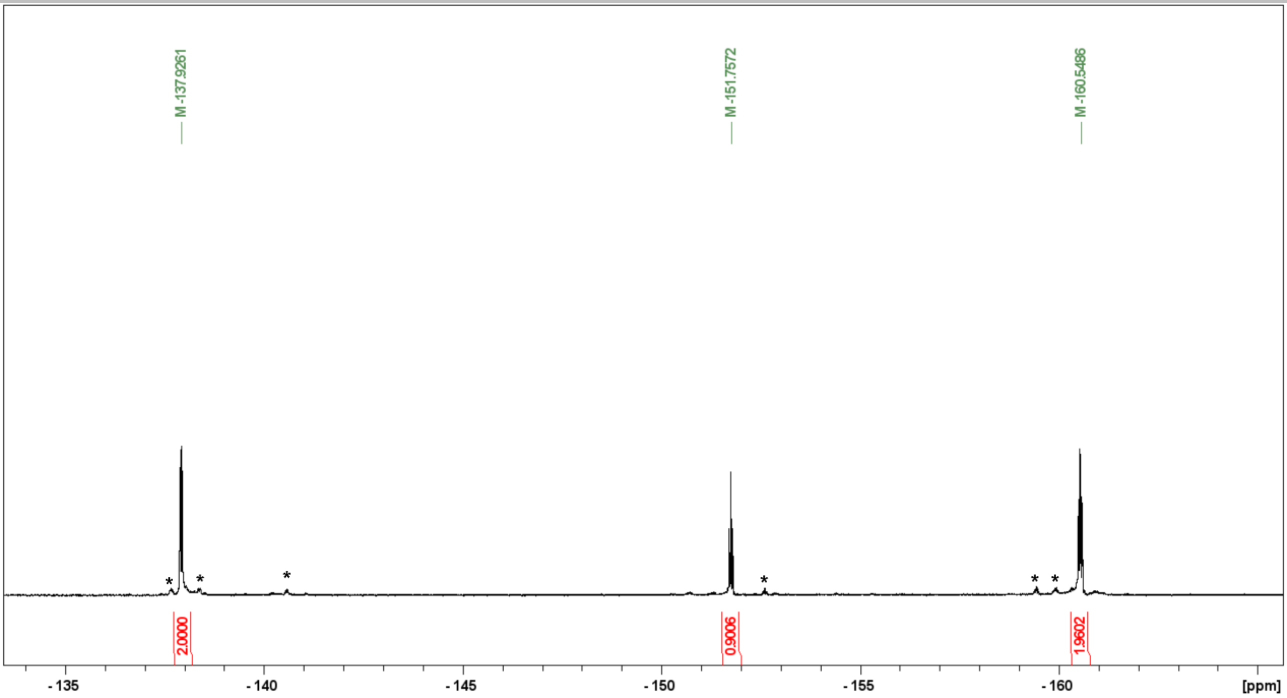

Figure S 81. The ^{19}F NMR spectrum of **16b-H** ([D]chloroform, 300 K, 471 MHz).

The NMR spectra of 17b-H

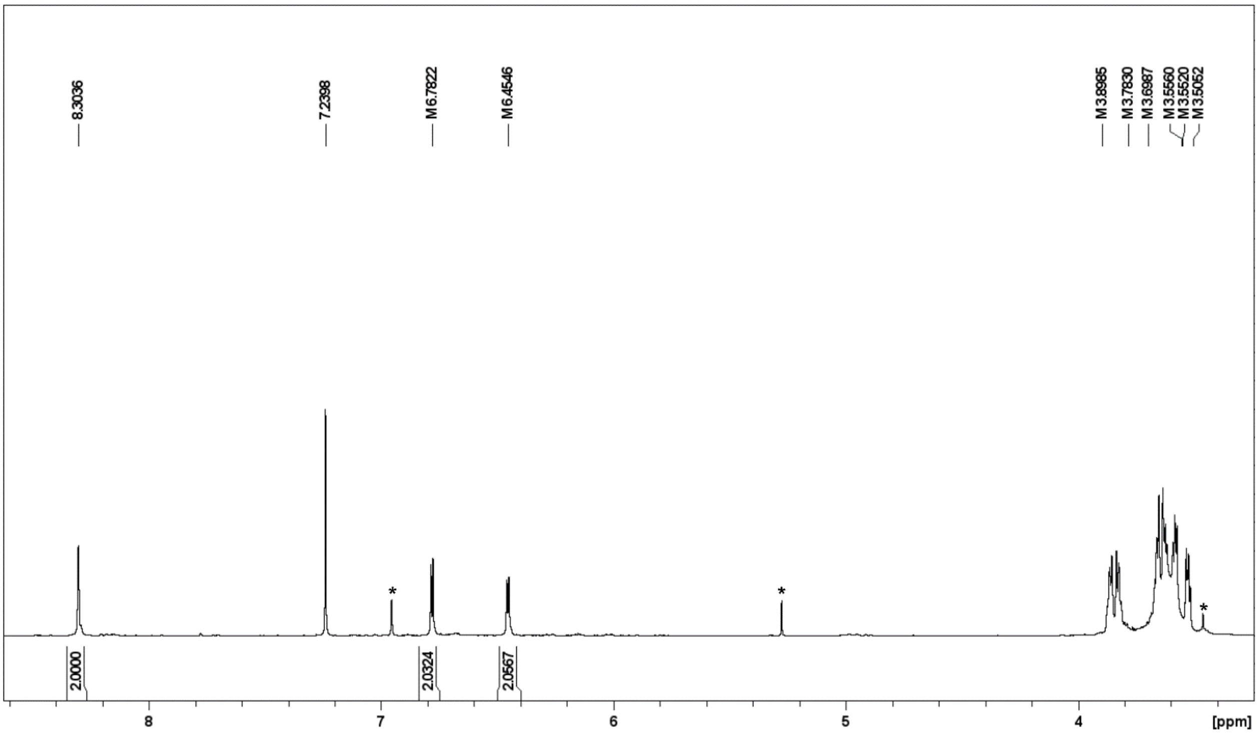

Figure S 82. The ^{1}H NMR spectrum of **17b-H** ([D]chloroform, 300 K, 500 MHz). Signals corresponding to impurities were marked with asterisks.

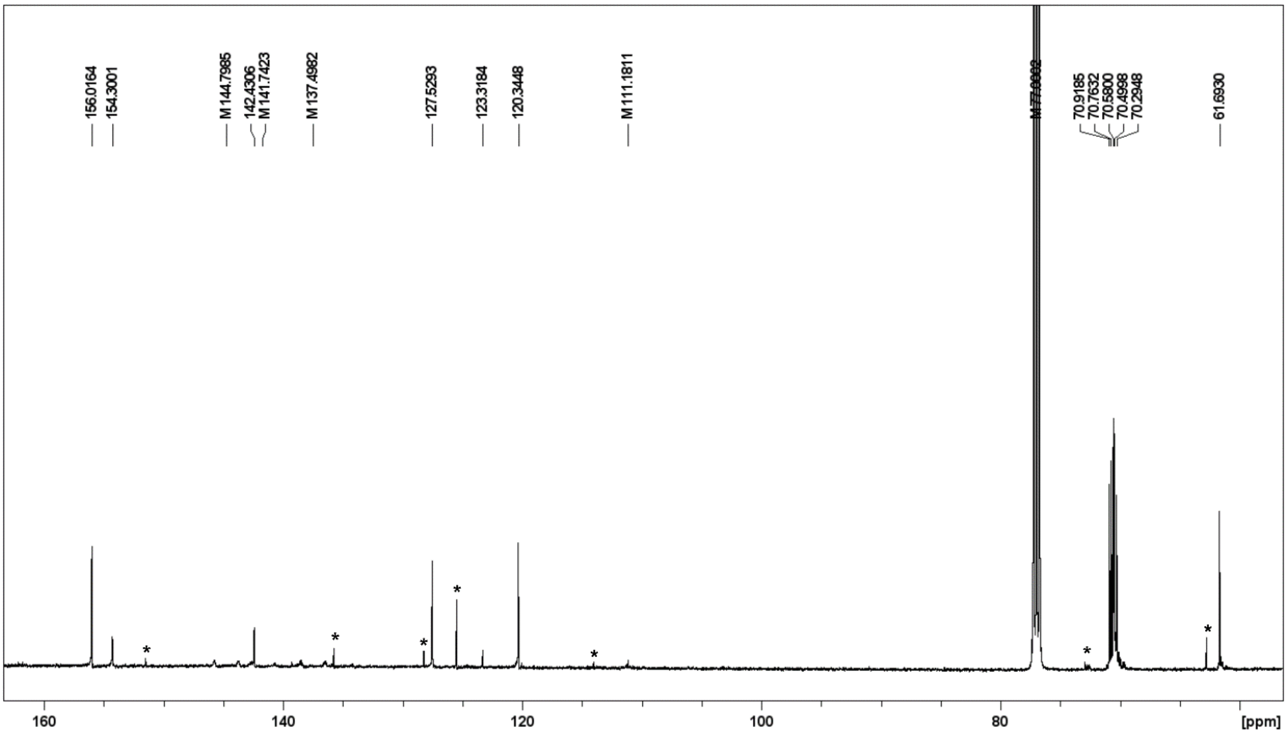

Figure S 83. The ^{13}C NMR spectrum of **17b-H** ([D]chloroform, 300 K, 125 MHz). Signals corresponding to impurities were marked with asterisks.

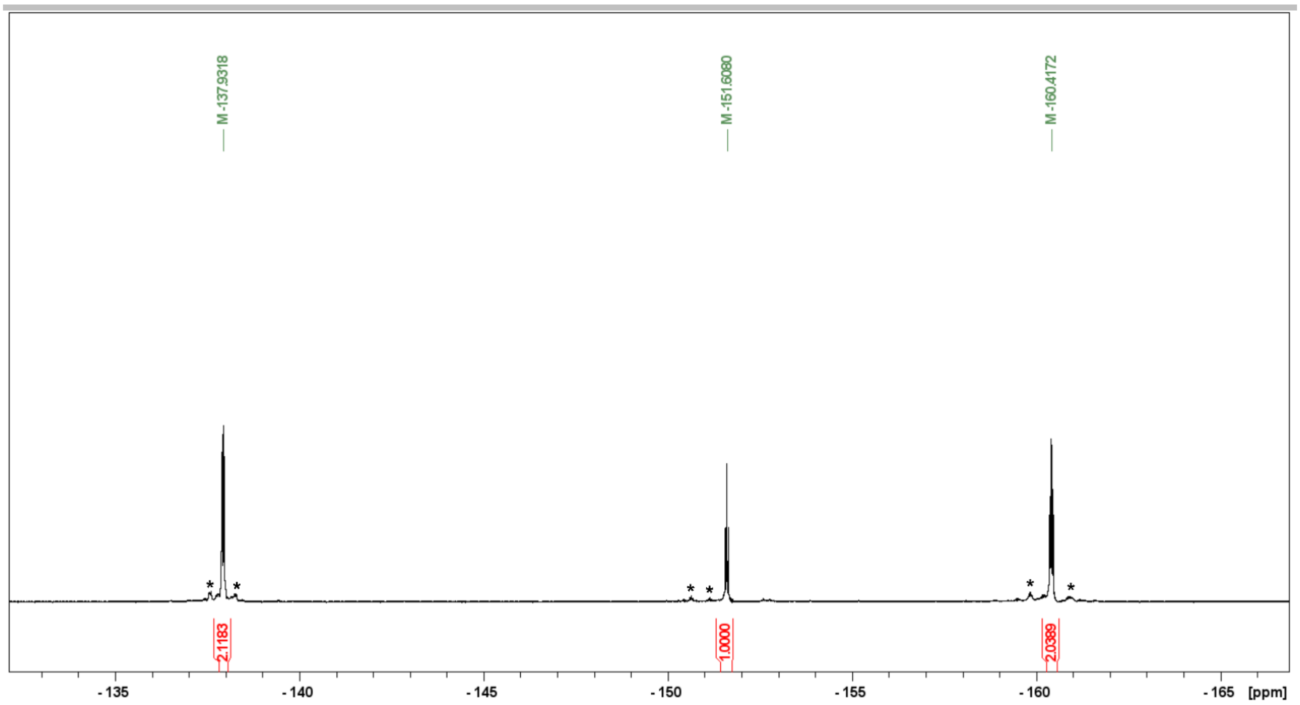

Figure S 84. The ^{19}F NMR spectrum of **17b-H** ([D]chloroform, 300 K, 471 MHz).

The NMR spectra of 18b-H

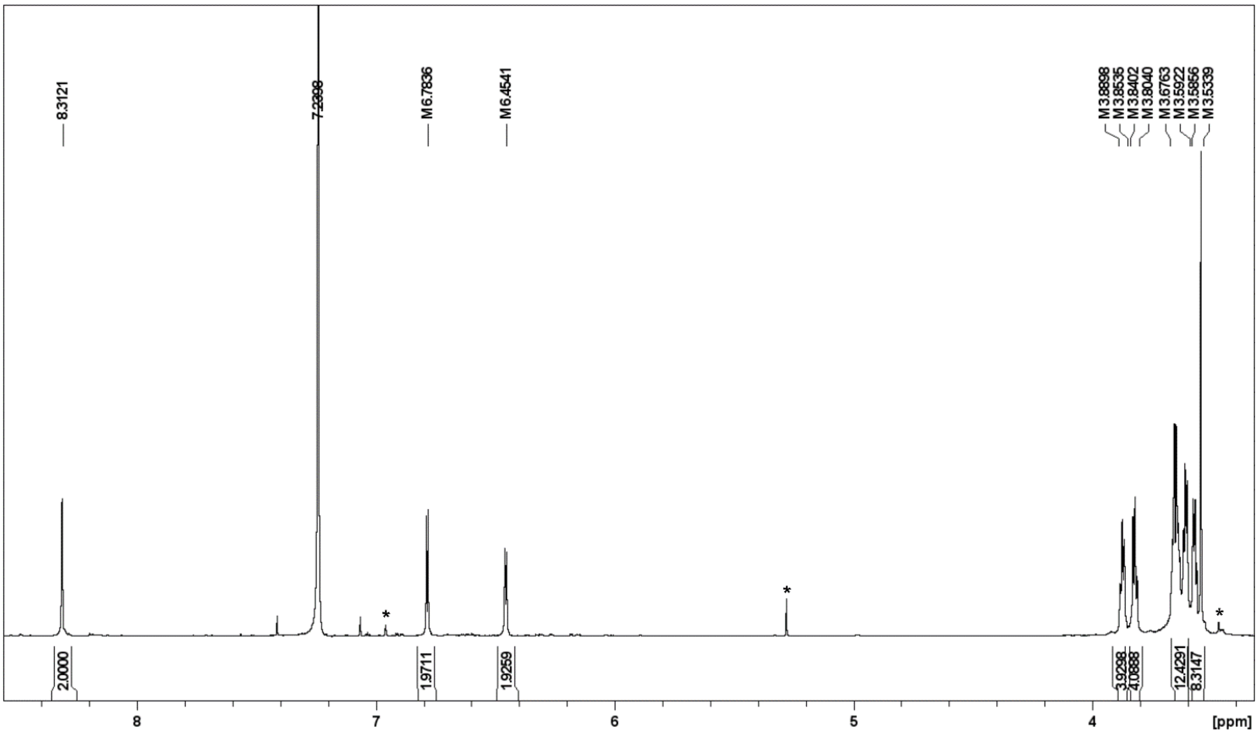

Figure S 85. The ^{1}H NMR spectrum of **18b-H** ([D]chloroform, 300 K, 600 MHz). Signals corresponding to impurities were marked with asterisks.

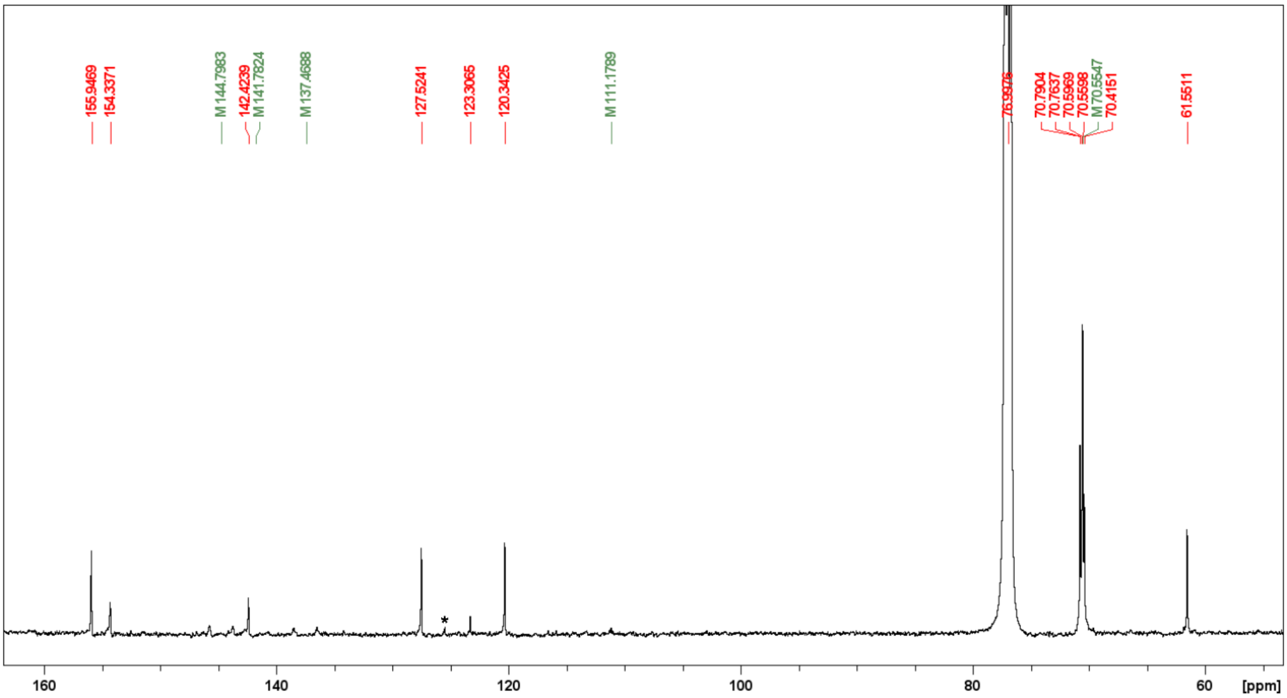

Figure S 86. The ^{13}C NMR spectrum of **18b-H** ([D]chloroform, 300 K, 125 MHz). Signals corresponding to impurities were marked with asterisks.

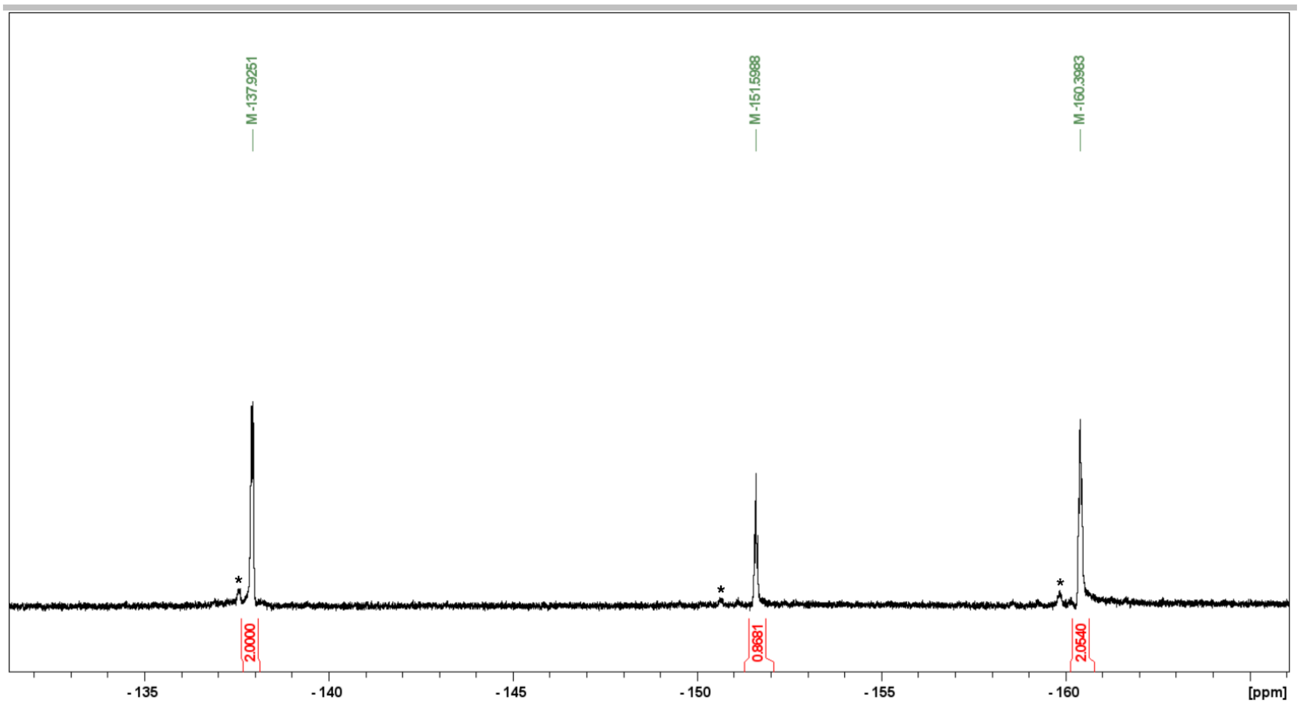

Figure S 87. The ^{13}C NMR spectrum of **18b-H** ([D]chloroform, 300 K, 471 MHz).

The NMR spectra of 19b-H

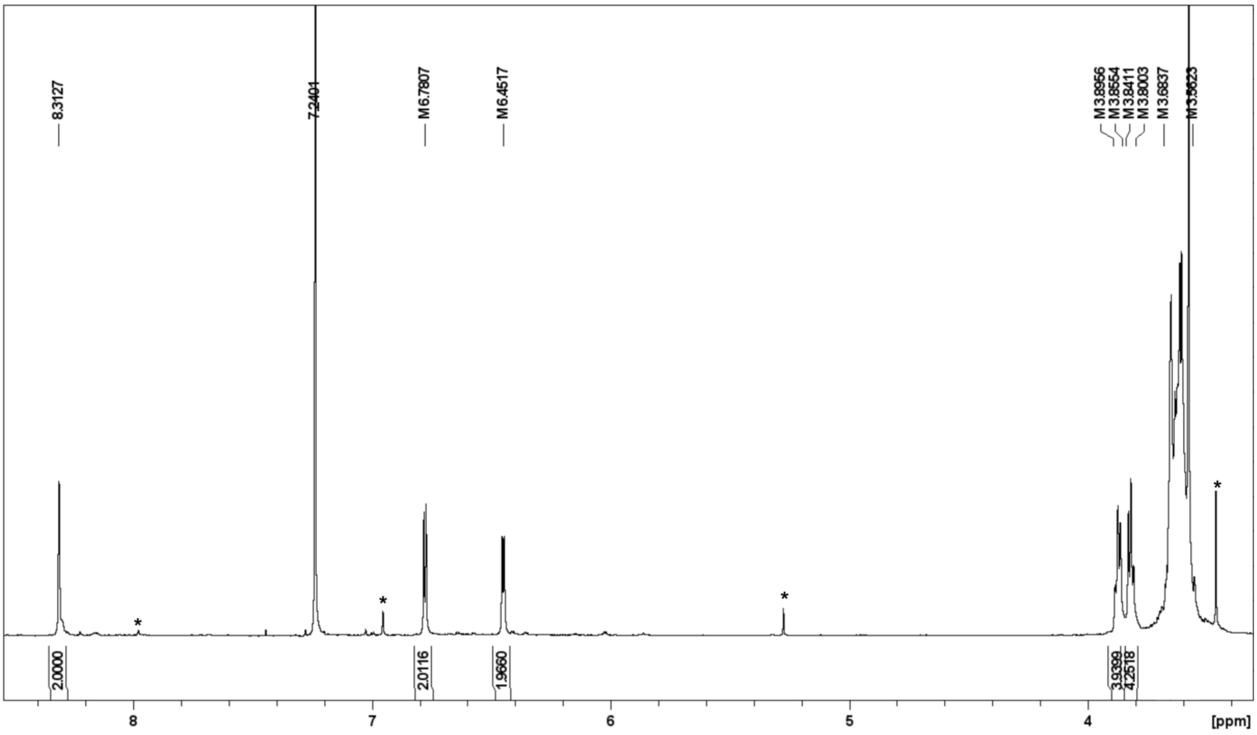

Figure S 88. The ^{1}H NMR spectrum of **19b-H** ([D]chloroform, 300 K, 500 MHz). Signals corresponding to impurities were marked with asterisks.

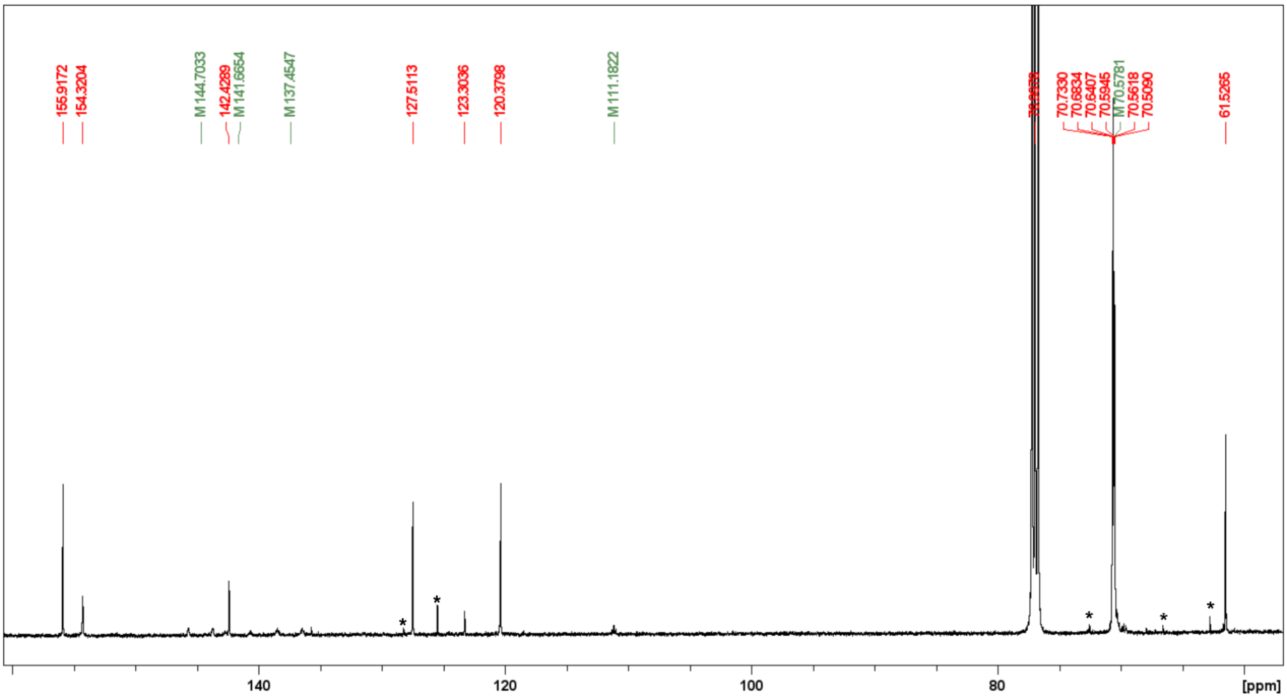

Figure S 89. The ^{13}C NMR spectrum of **19b-H** ([D]chloroform, 300 K, 125 MHz). Signals corresponding to impurities were marked with asterisks.

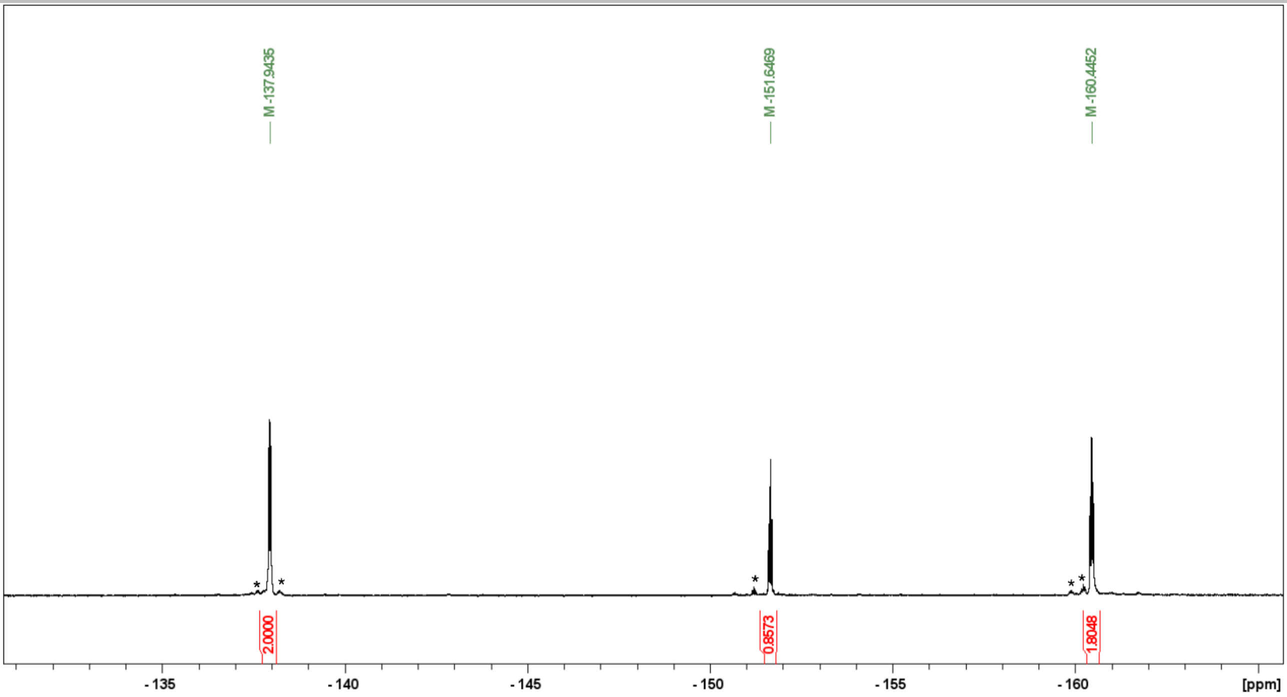

Figure S 90. The ^{19}F NMR spectrum of **19b-H** ([D]chloroform, 300 K, 471 MHz).

The NMR spectra of 20a-Pb

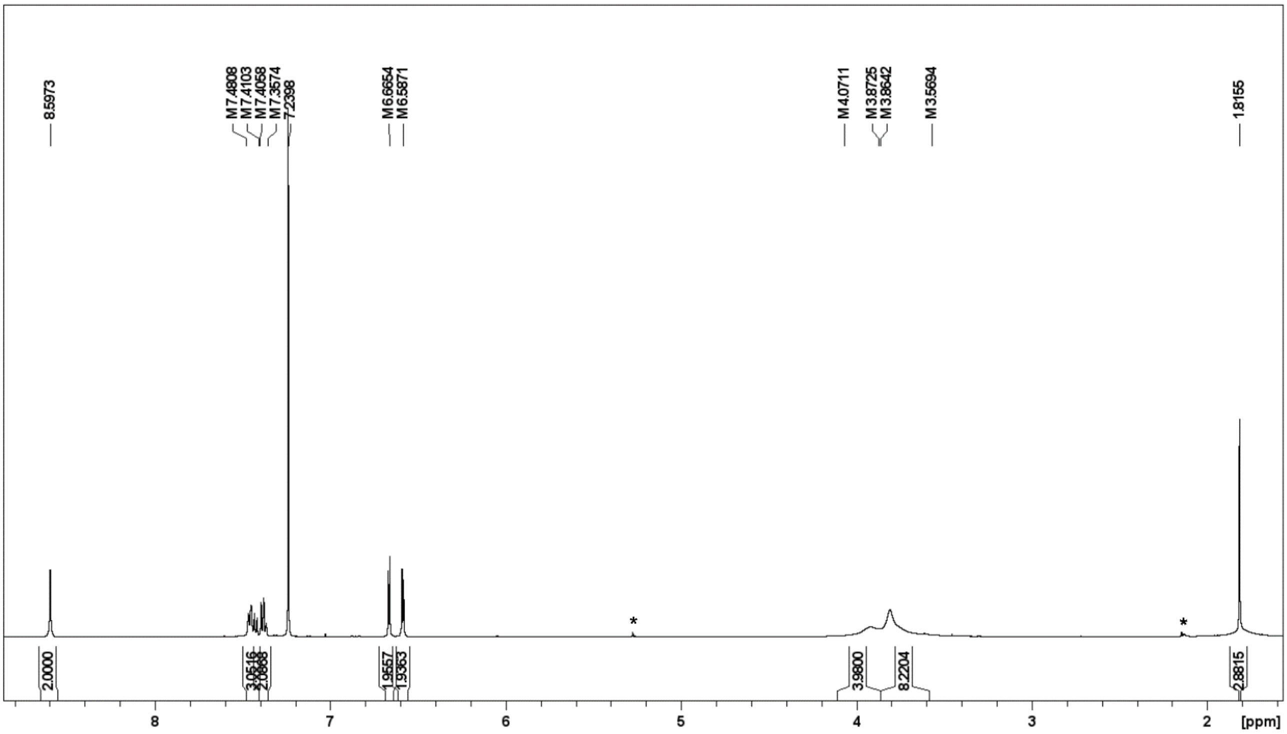

Figure S 91. The ^{1}H NMR spectrum of **20a-Pb** ([D]chloroform, 300 K, 500 MHz). Signals corresponding to impurities were marked with asterisks.

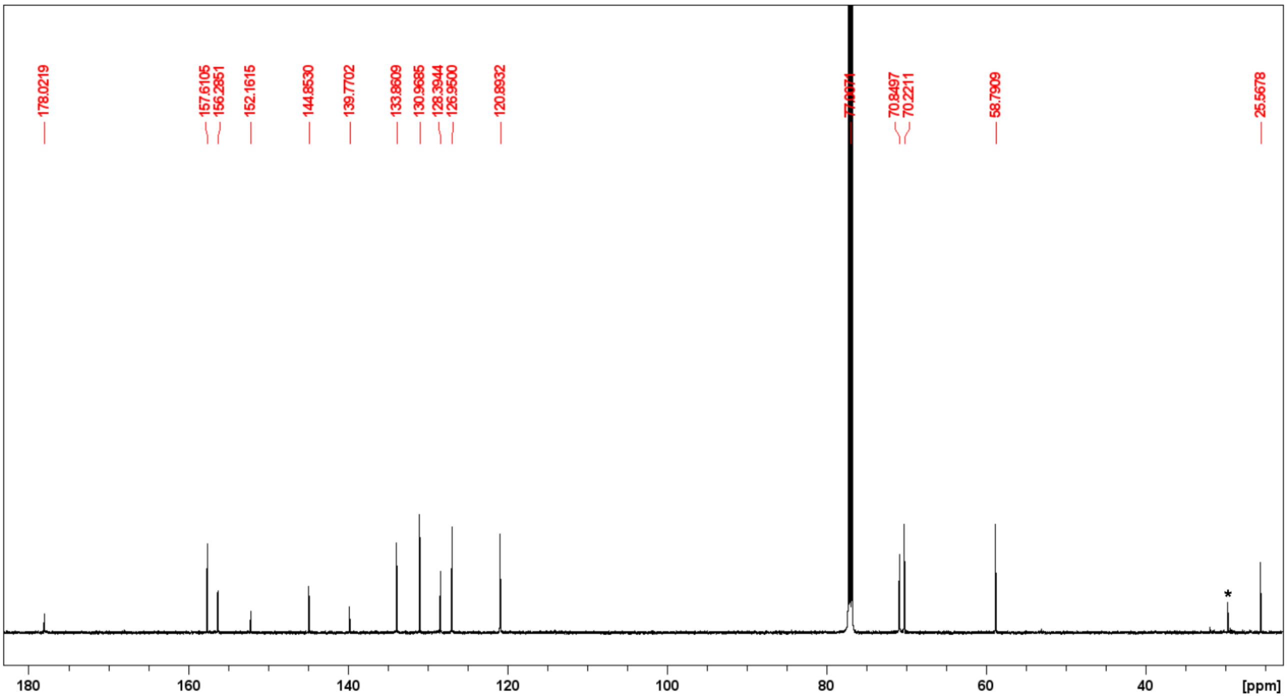

Figure S 92. The ^{13}C NMR spectrum of **20a-Pb** ([D]chloroform, 300 K, 151 MHz). Signals corresponding to impurities were marked with asterisks.

The NMR spectra of 20b-Pb

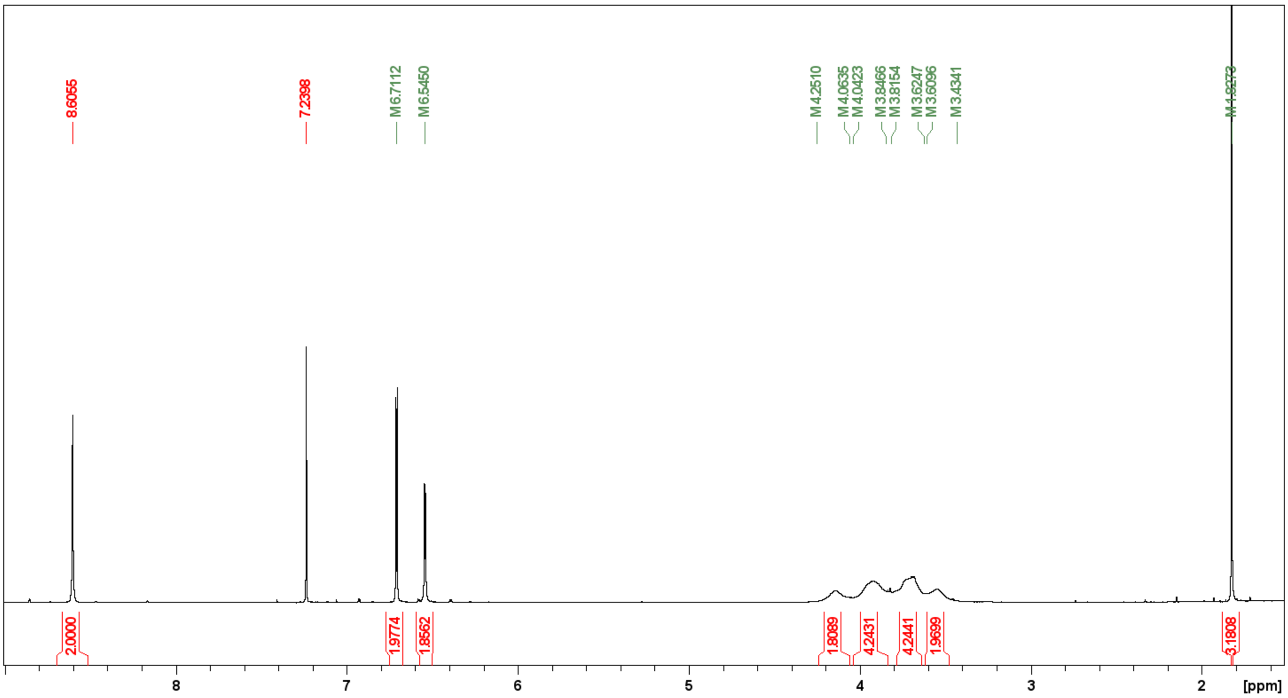

Figure S 93. The ^{1}H NMR spectrum of **20b-Pb** ([D]chloroform, 300 K, 600 MHz).

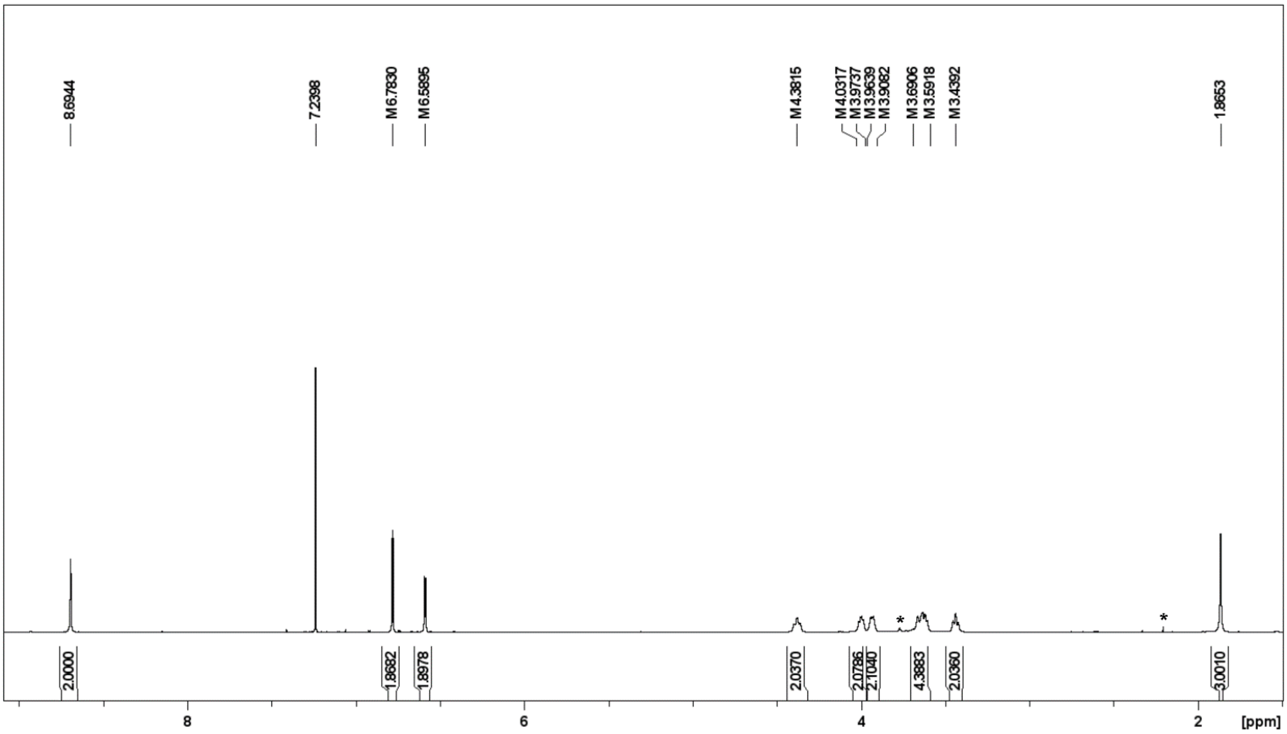

Figure S 94. The ^{1}H NMR spectrum of **20b-Pb** ([D]chloroform, 210 K, 600 MHz).

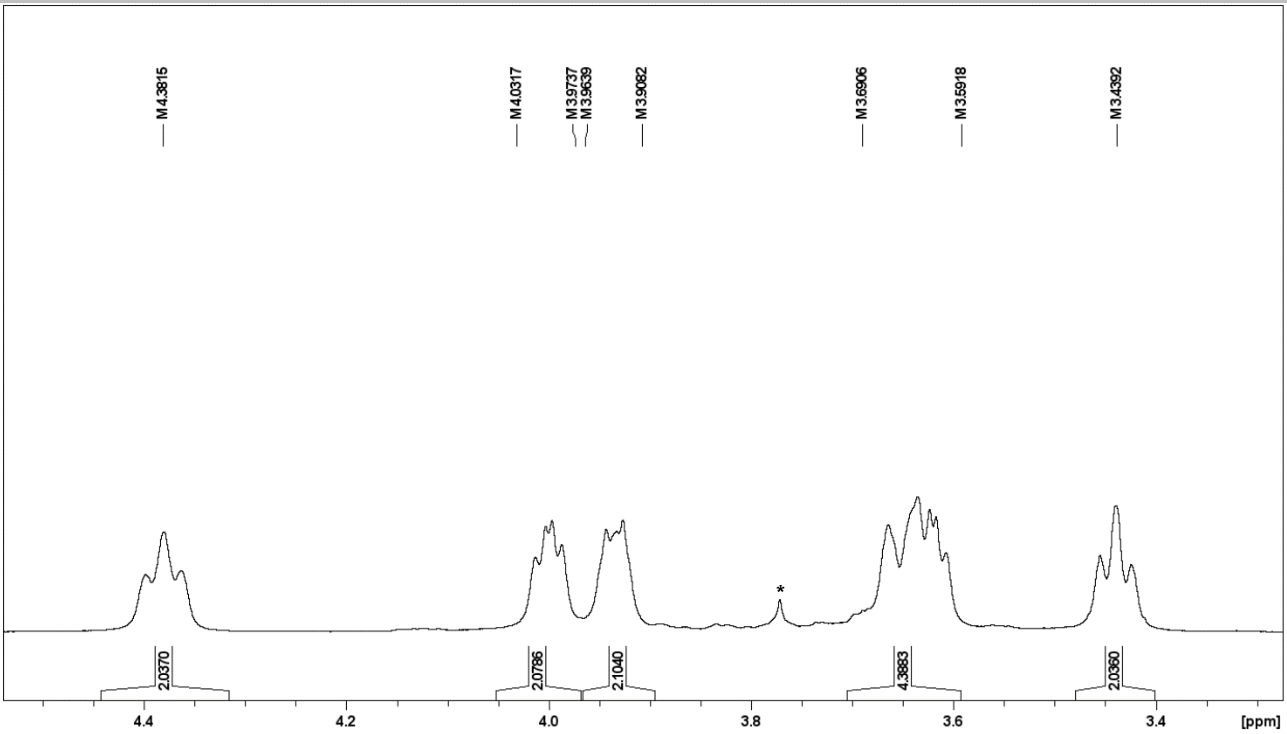

Figure S 95. The oligo(ethylene glycol) chain region of the ^{1}H NMR spectrum of **20b-Pb** ([D]chloroform, 210 K, 600 MHz).

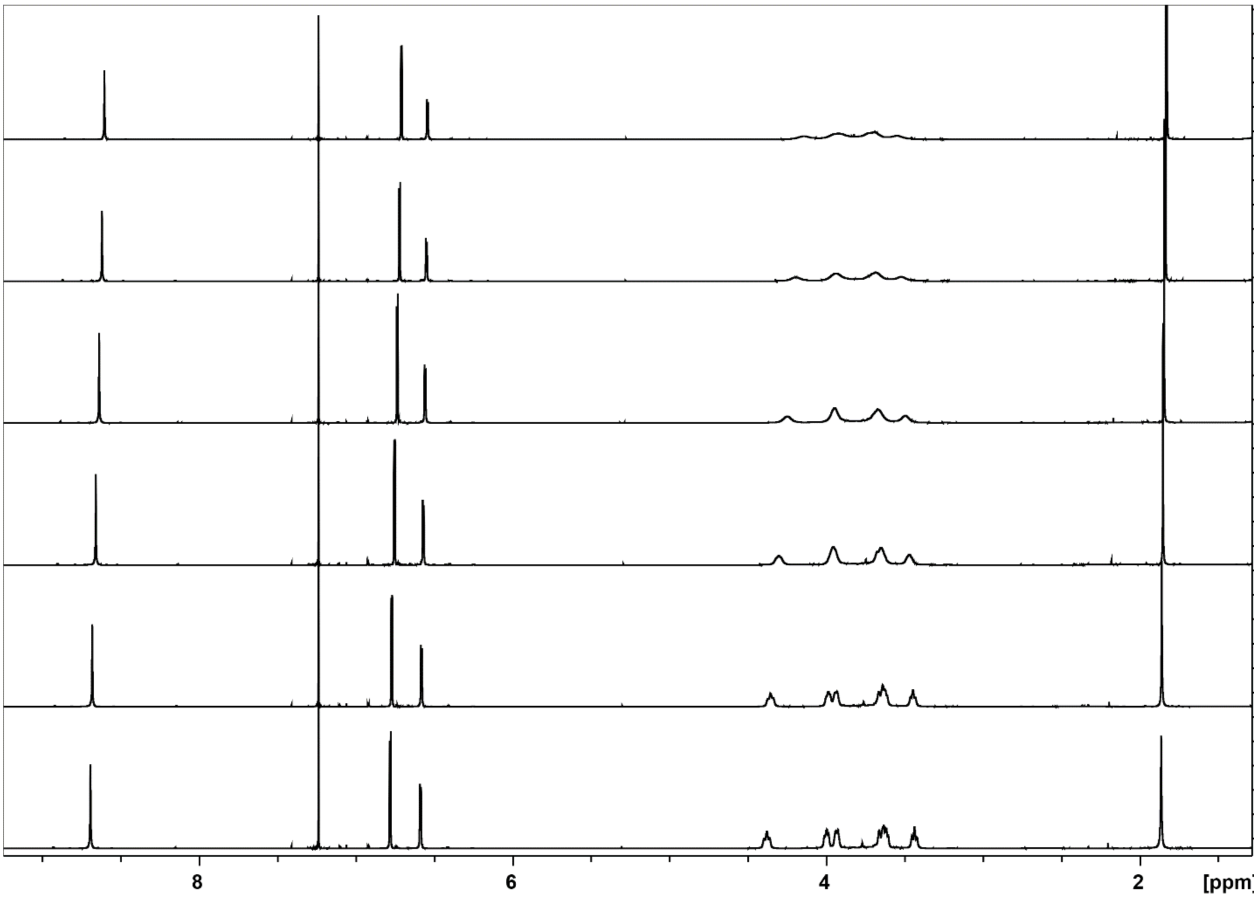

Figure S 96. ^{1}H NMR spectra of **20b-Pb** recorded in 300 K (top) – 210 K (bottom) temperature range every 20 K, and 10 K between 220 K and 210 K ([D]chloroform, 600 MHz).

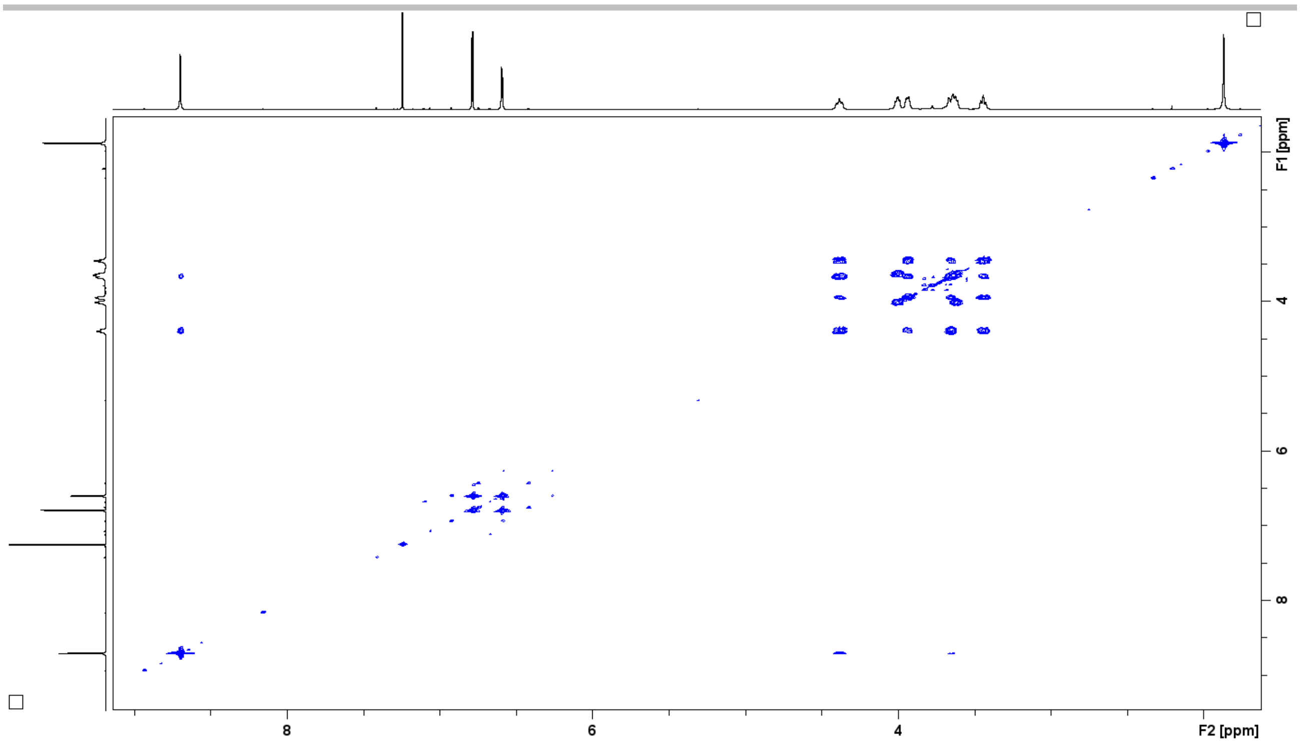

Figure S 97. The ^1H-^1H COSY spectrum of **20b-Pb** ([D]chloroform, 210 K, 600 MHz).

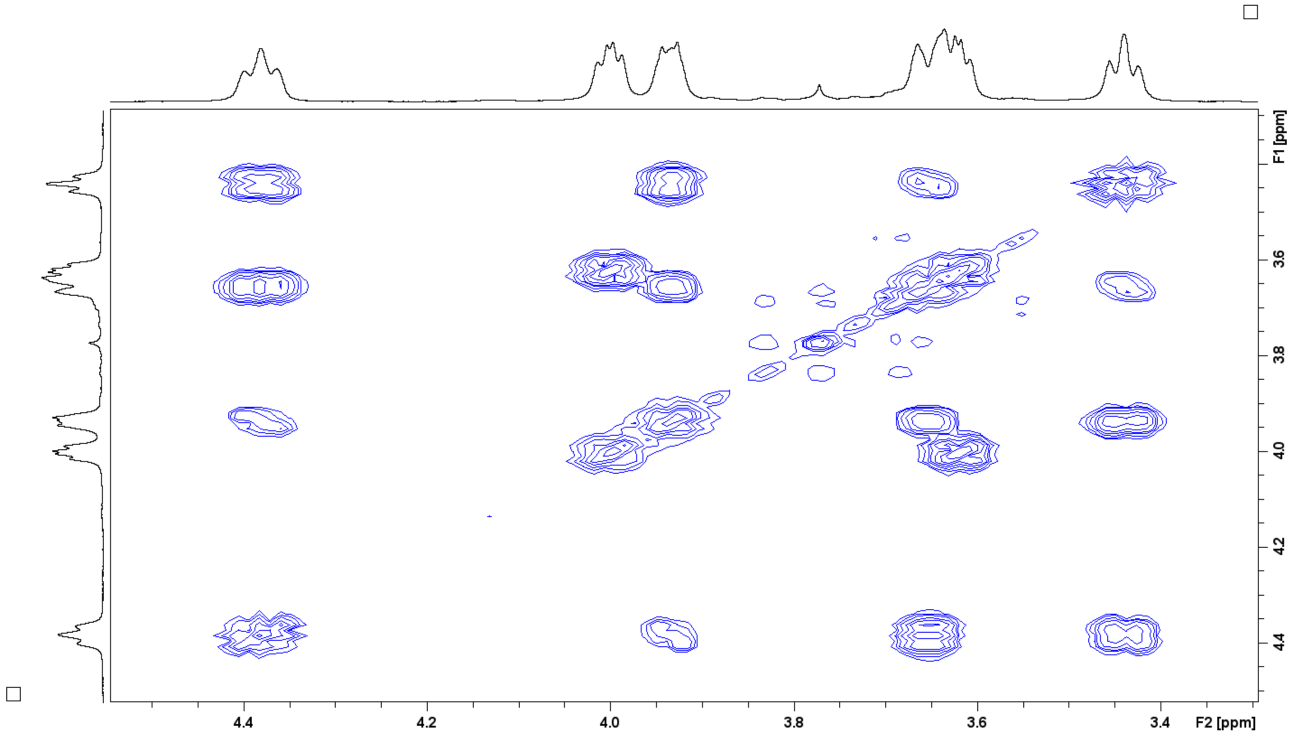

Figure S 98. The oligo(ethylene glycol) chain region of the ^1H-^1H COSY spectrum of **20b-Pb** ([D]chloroform, 300 K, 600 MHz).

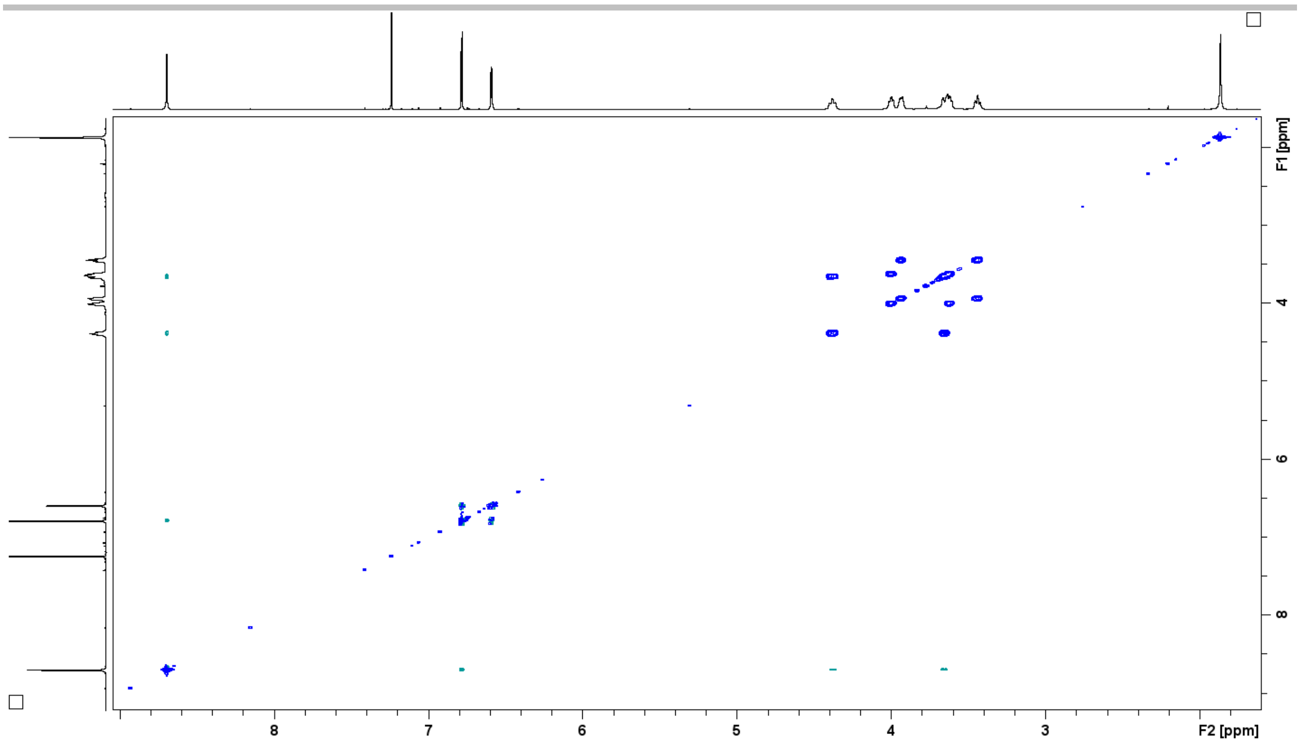

Figure S 99. The ^{1}H-^{1}H ROESY spectrum of **20b-Pb** ([D]chloroform, 210 K, 600 MHz).

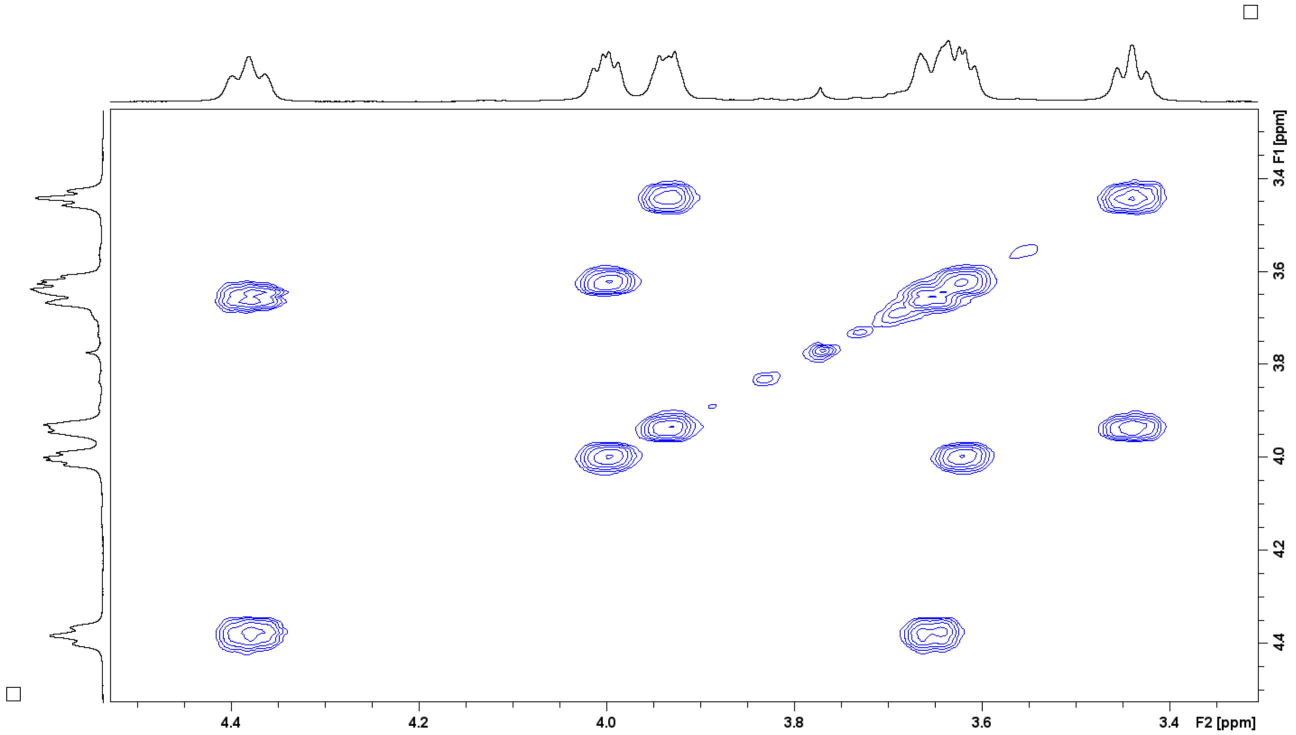

Figure S 100. The oligo(ethylene glycol) chain region of the ROESY spectrum of **20b-Pb** ([D]chloroform, 210 K, 600 MHz).

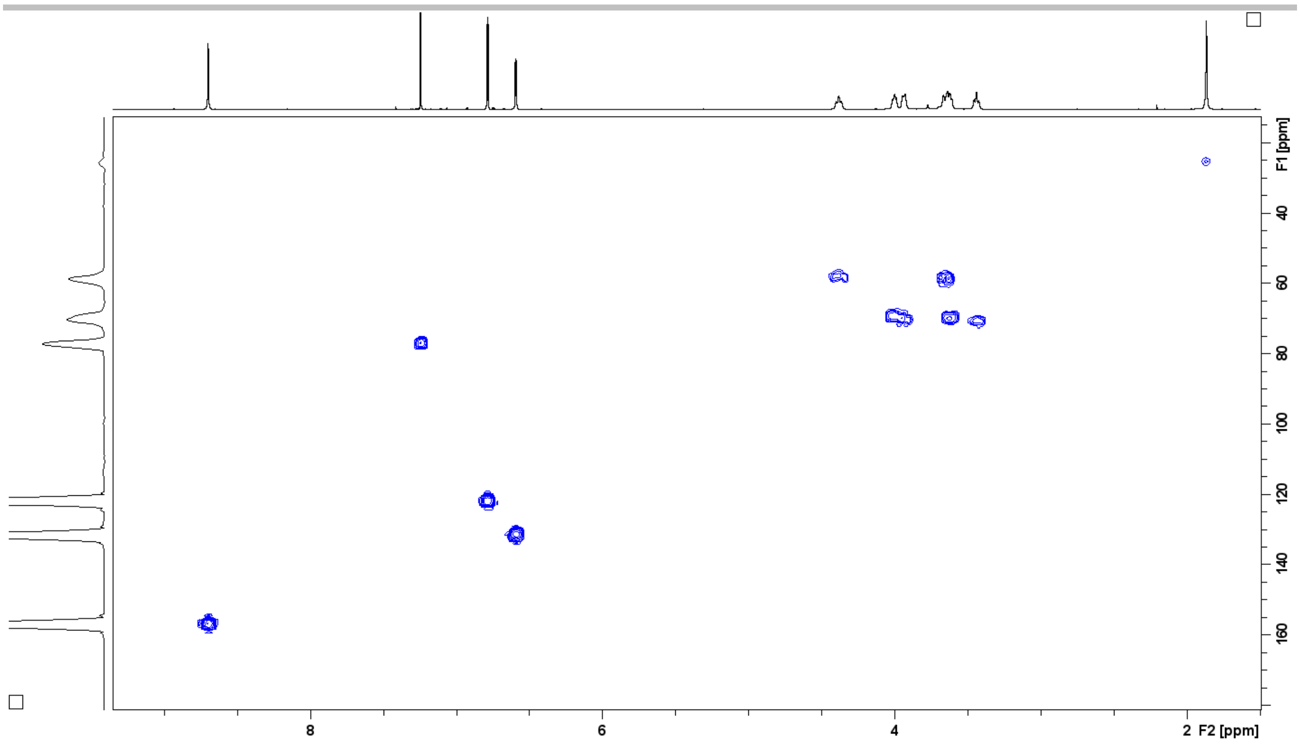

Figure S 101. The ^{1}H-^{13}C HMQC spectrum of **20b-Pb** ([D]chloroform, 210 K, 600 MHz).

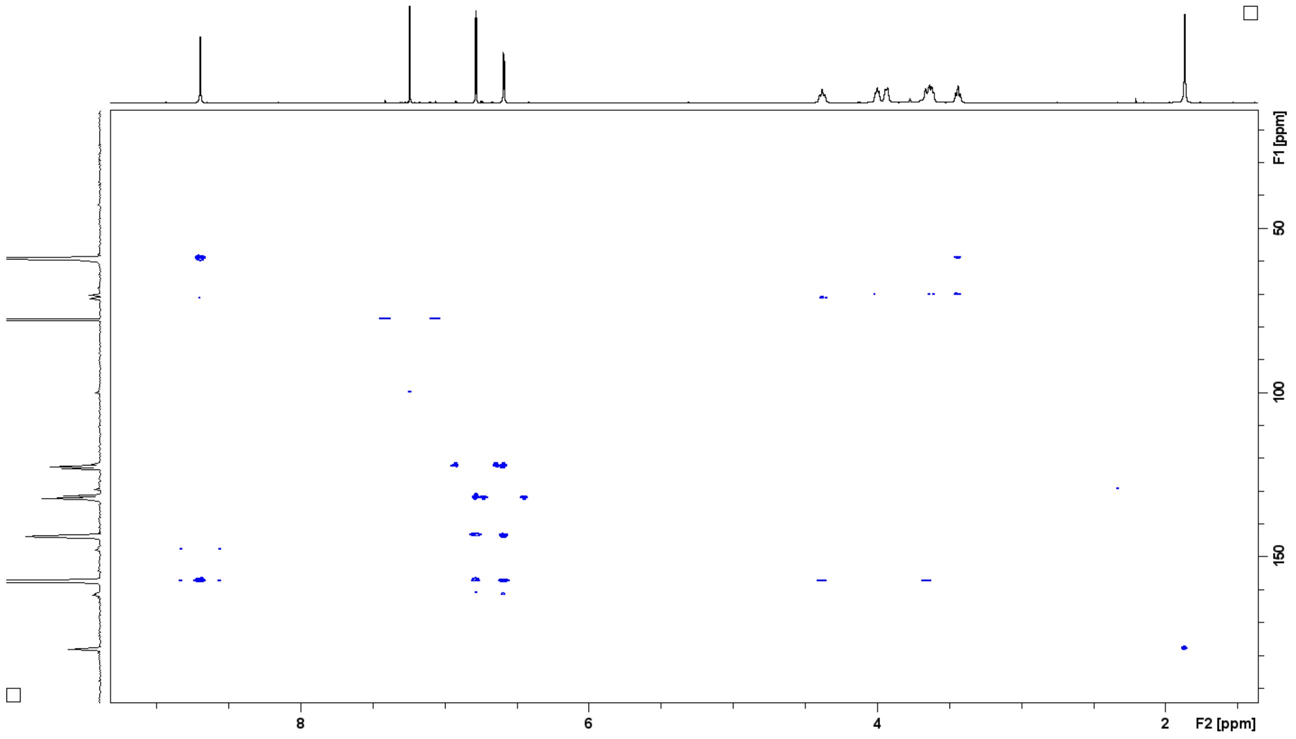

Figure S 102. The ^{1}H-^{13}C HMBC spectrum of **20b-Pb** ([D]chloroform, 210 K, 600 MHz).

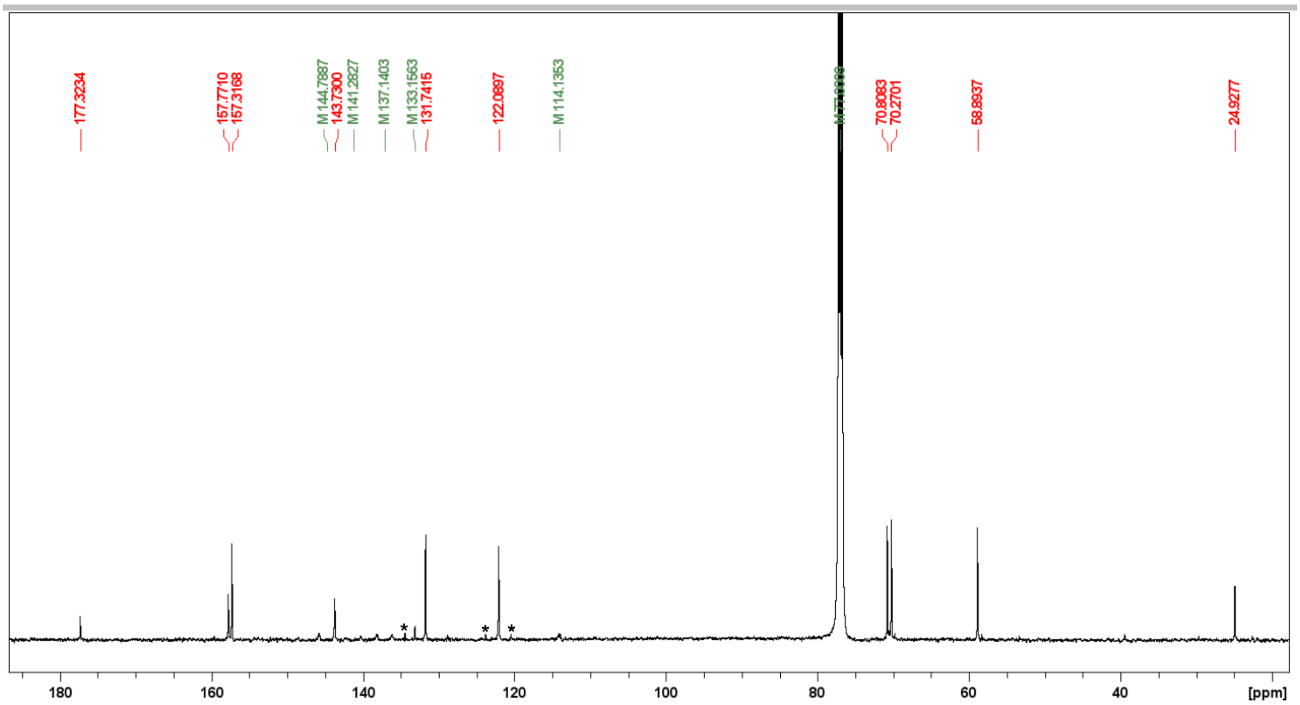

Figure S 103. The ^{13}C NMR spectrum of **20b-Pb** ([D]chloroform, 300 K, 125 MHz). Signals corresponding to impurities were marked with asterisks.

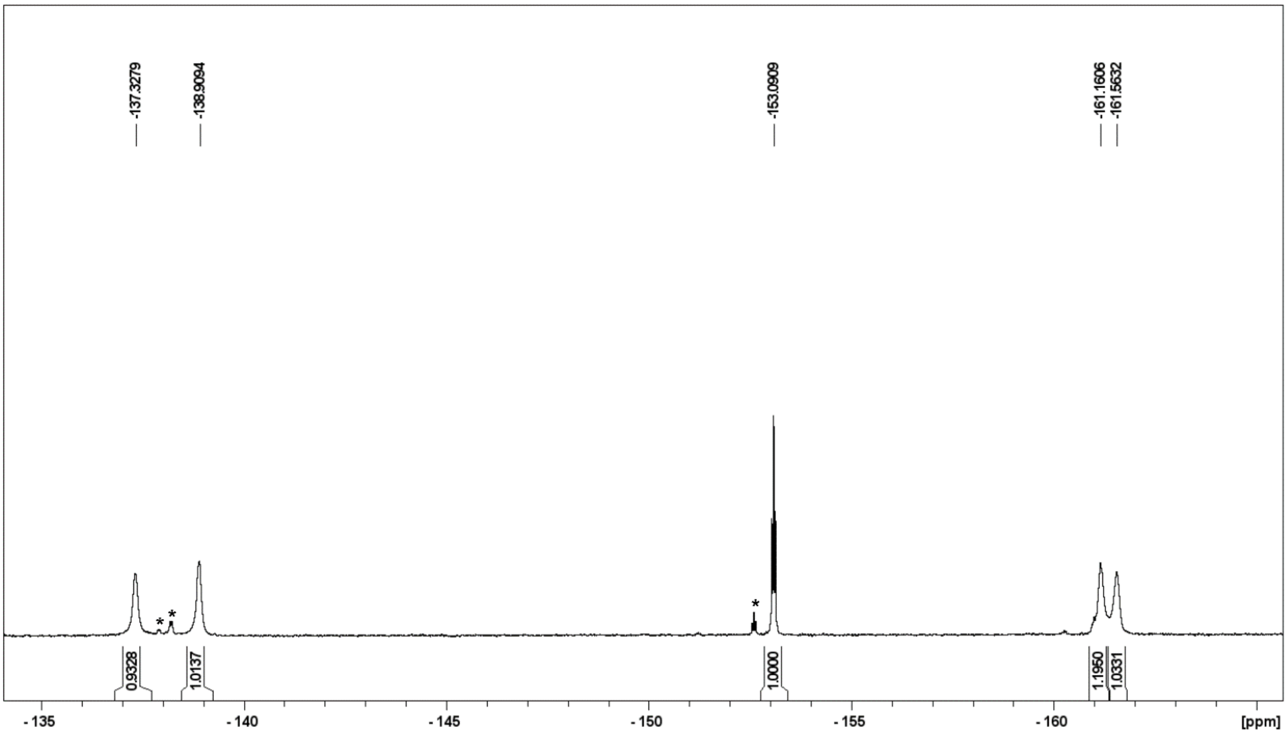

Figure S 104. The ^{19}F NMR spectrum of **20b-Pb** ([D]chloroform, 300 K, 471 MHz). Signals corresponding to impurities were marked with asterisks.

The NMR spectra of 21b-Pb

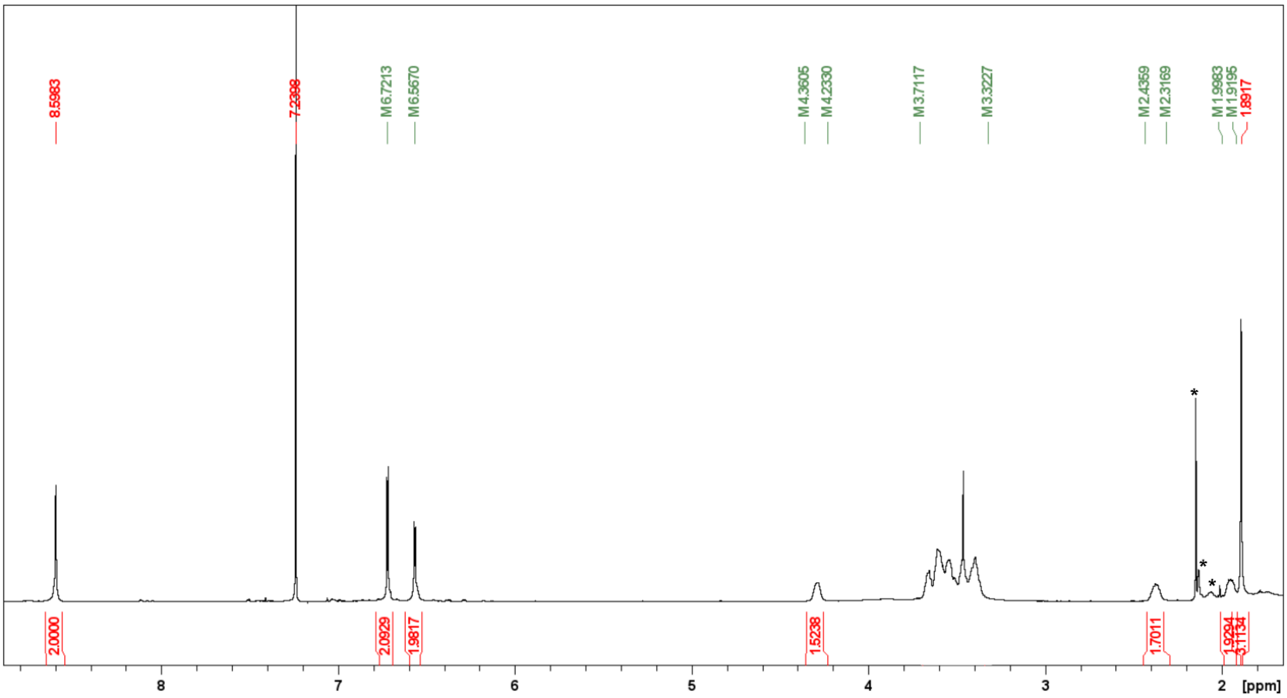

Figure S 105. The ^{1}H NMR spectrum of **21b-Pb** ([D]chloroform, 300 K, 600 MHz). Signals corresponding to impurities were marked with asterisks.

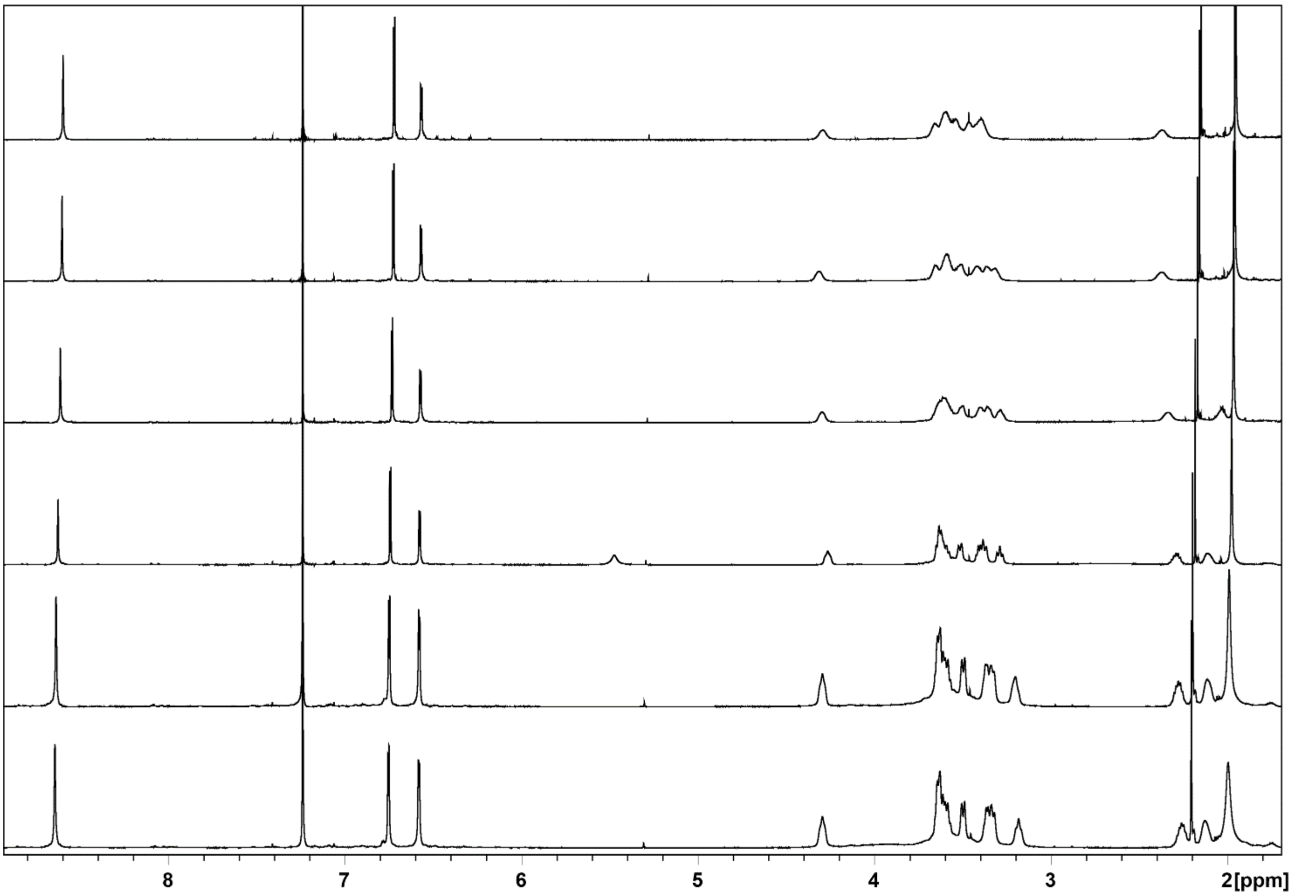

Figure S 106. ^{1}H NMR spectra of **21b-Pb** recorded in 300 K (top) – 210 K (bottom) temperature range every 20 K, and 10 K between 220 K and 210 K ([D]chloroform, 600 MHz).

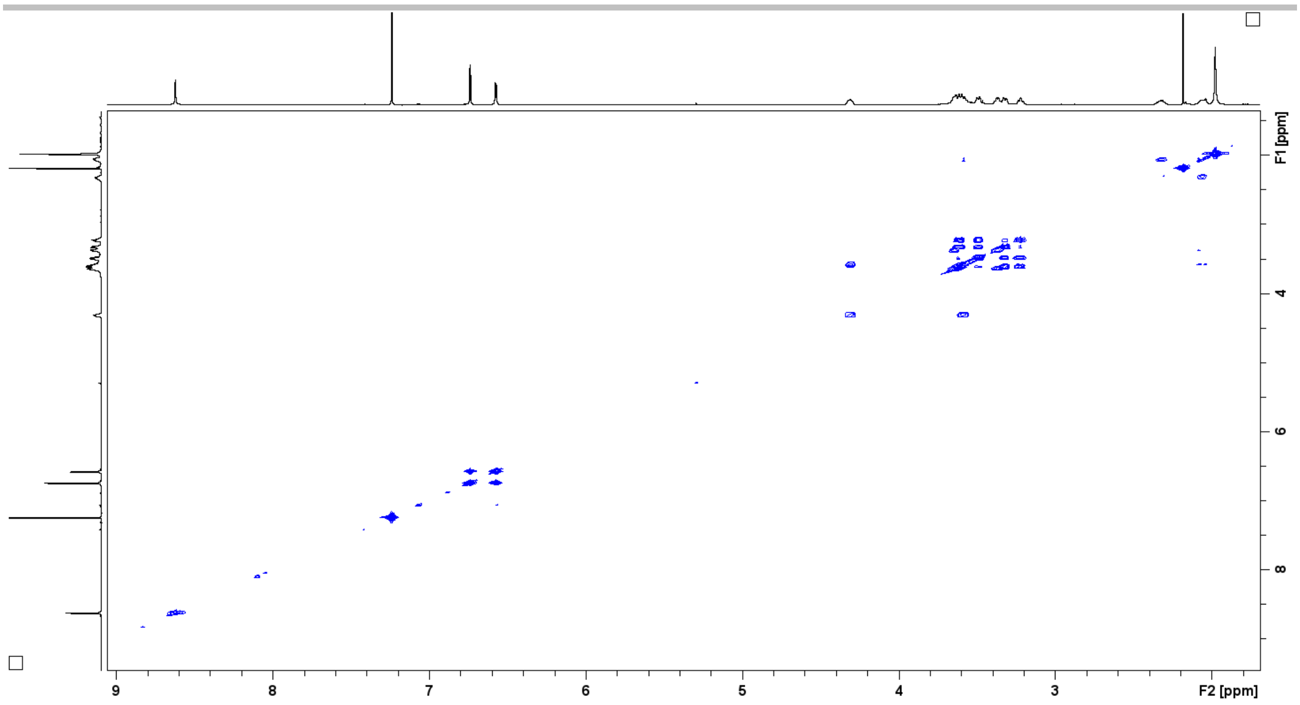

Figure S 107. The ^{1}H-^{1}H COSY spectrum of **21b-Pb** ([D]chloroform, 240 K, 600 MHz).

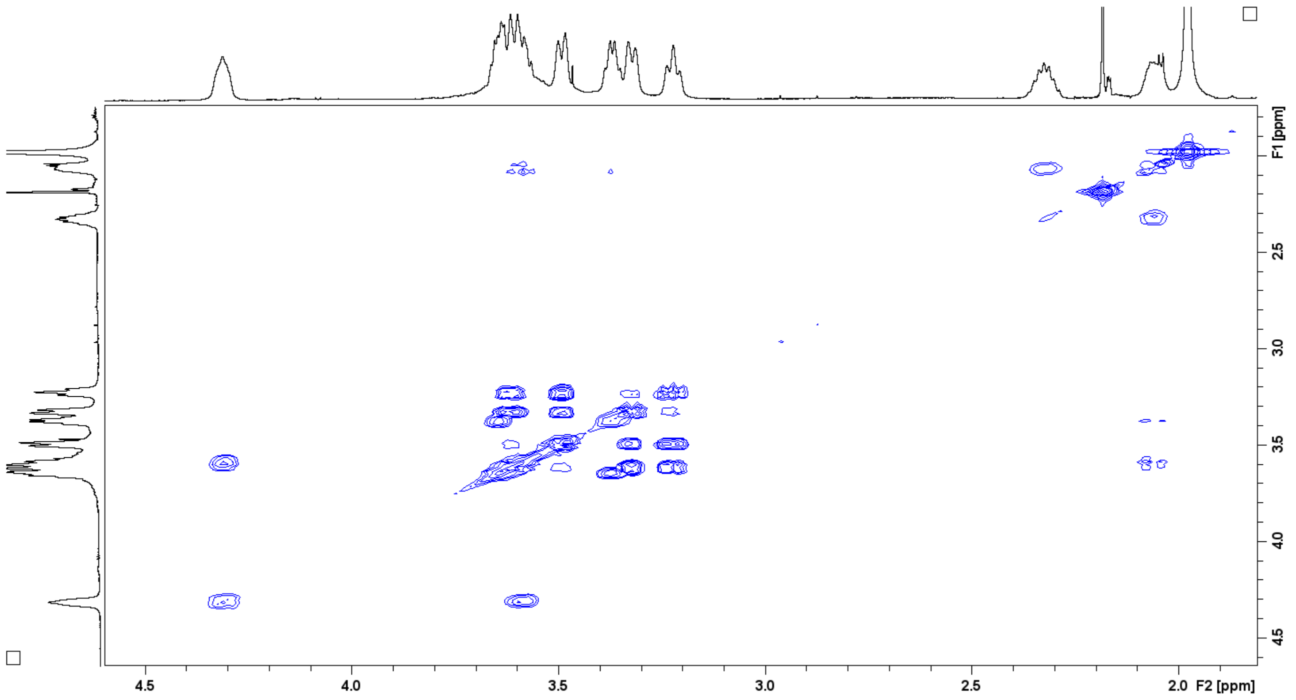

Figure S 108. The oligo(ethylene glycol) chain region of the ^{1}H-^{1}H COSY spectrum of **21b-Pb** ([D]chloroform, 240 K, 600 MHz).

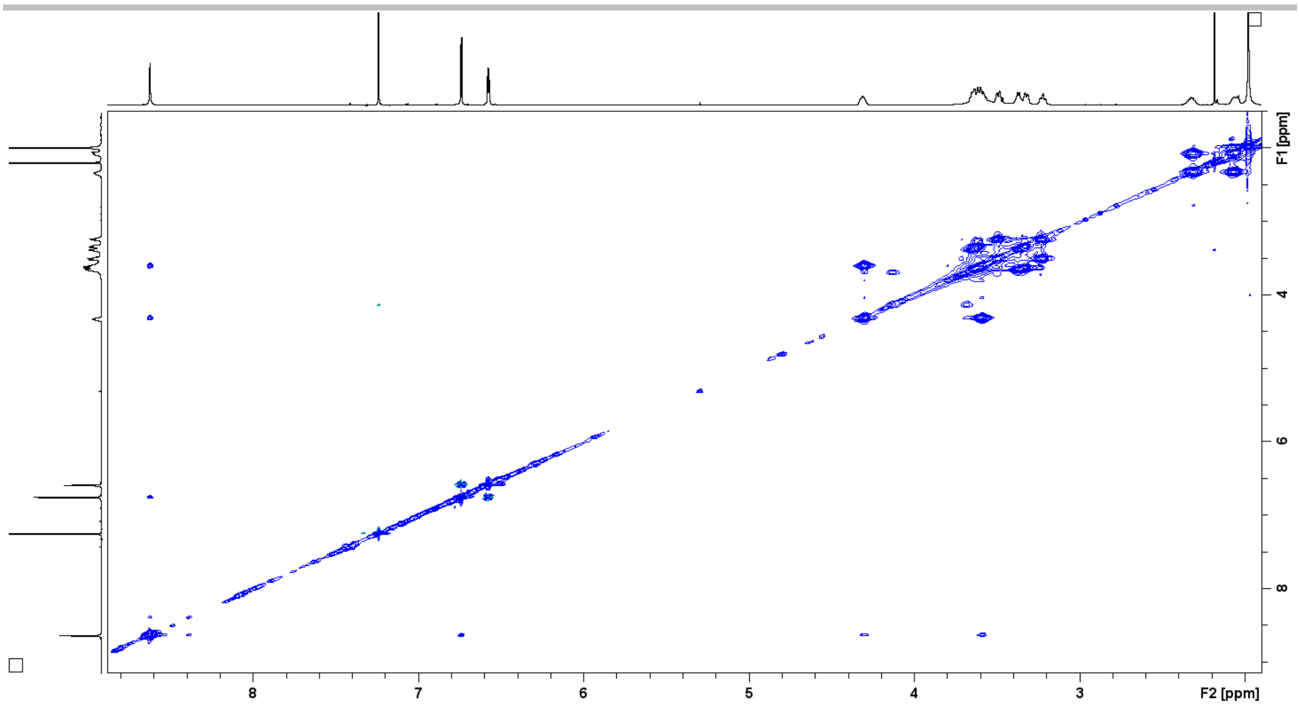

Figure S 109. The ^{1}H-^{1}H NOESY spectrum of **21b-Pb** ([D]chloroform, 240 K, 600 MHz).

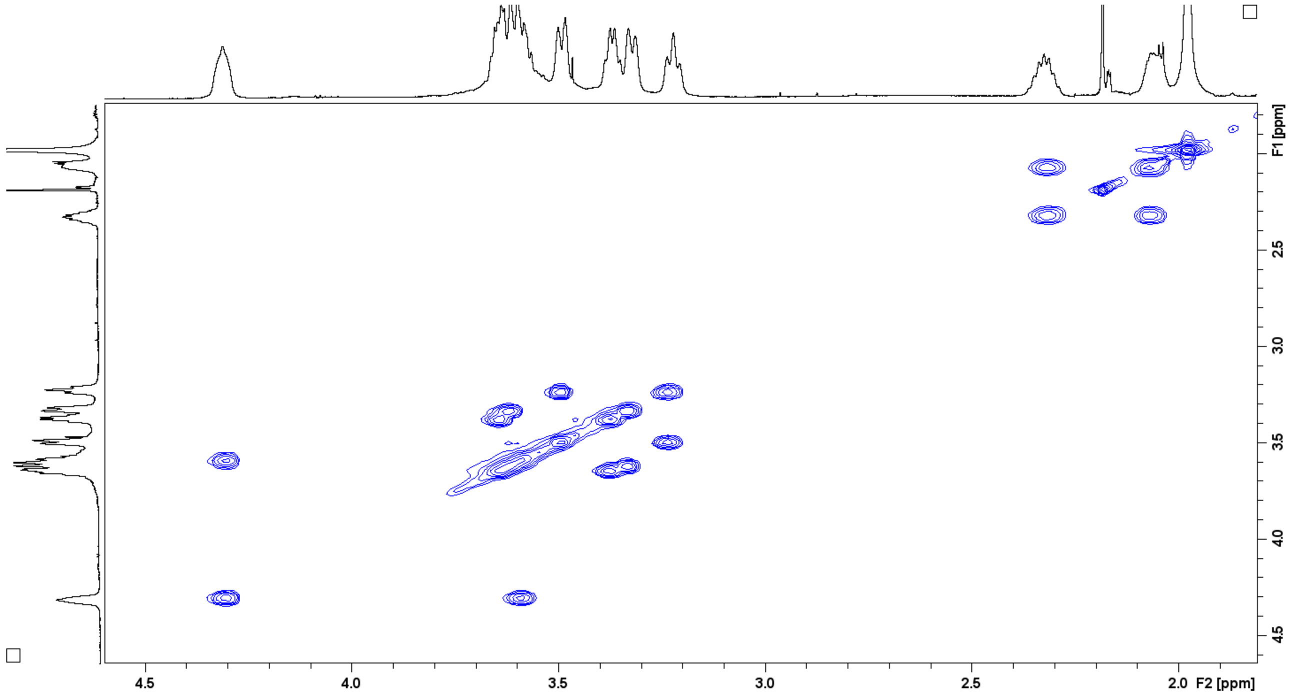

Figure S 110. The oligo(ethylene glycol) chain region of the NOESY spectrum of **21b-Pb** ([D]chloroform, 240 K, 600 MHz).

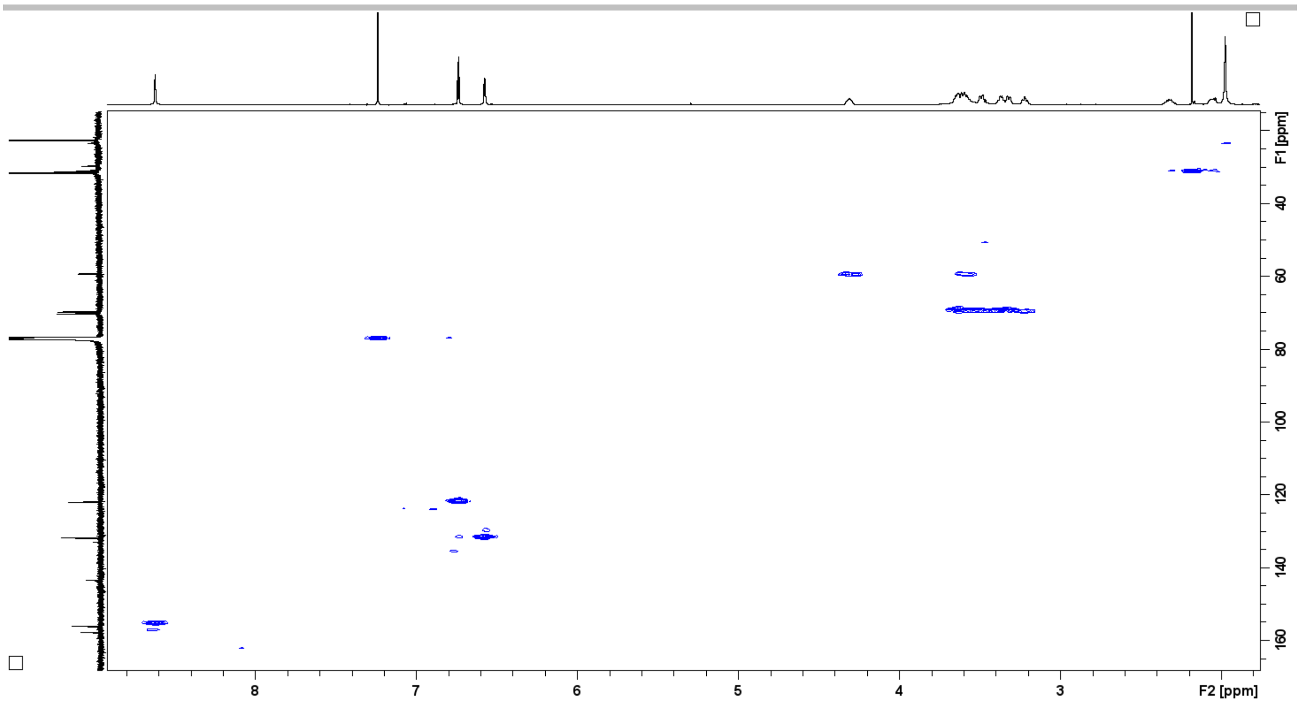

Figure S 111. The ^{1}H-^{13}C HMQC spectrum of **21b-Pb** ([D]chloroform, 240 K, 600 MHz).

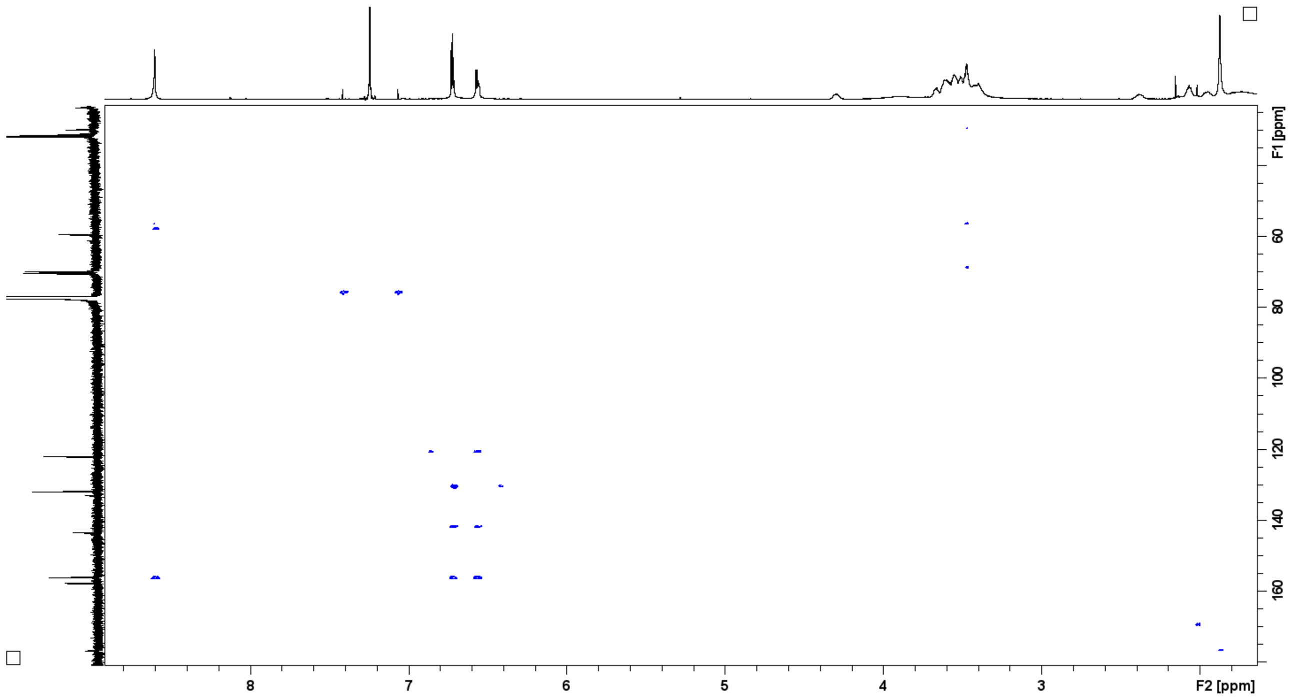

Figure S 112. The ^{1}H-^{13}C HMBC spectrum of **21b-Pb** ([D]chloroform, 300 K, 600 MHz).

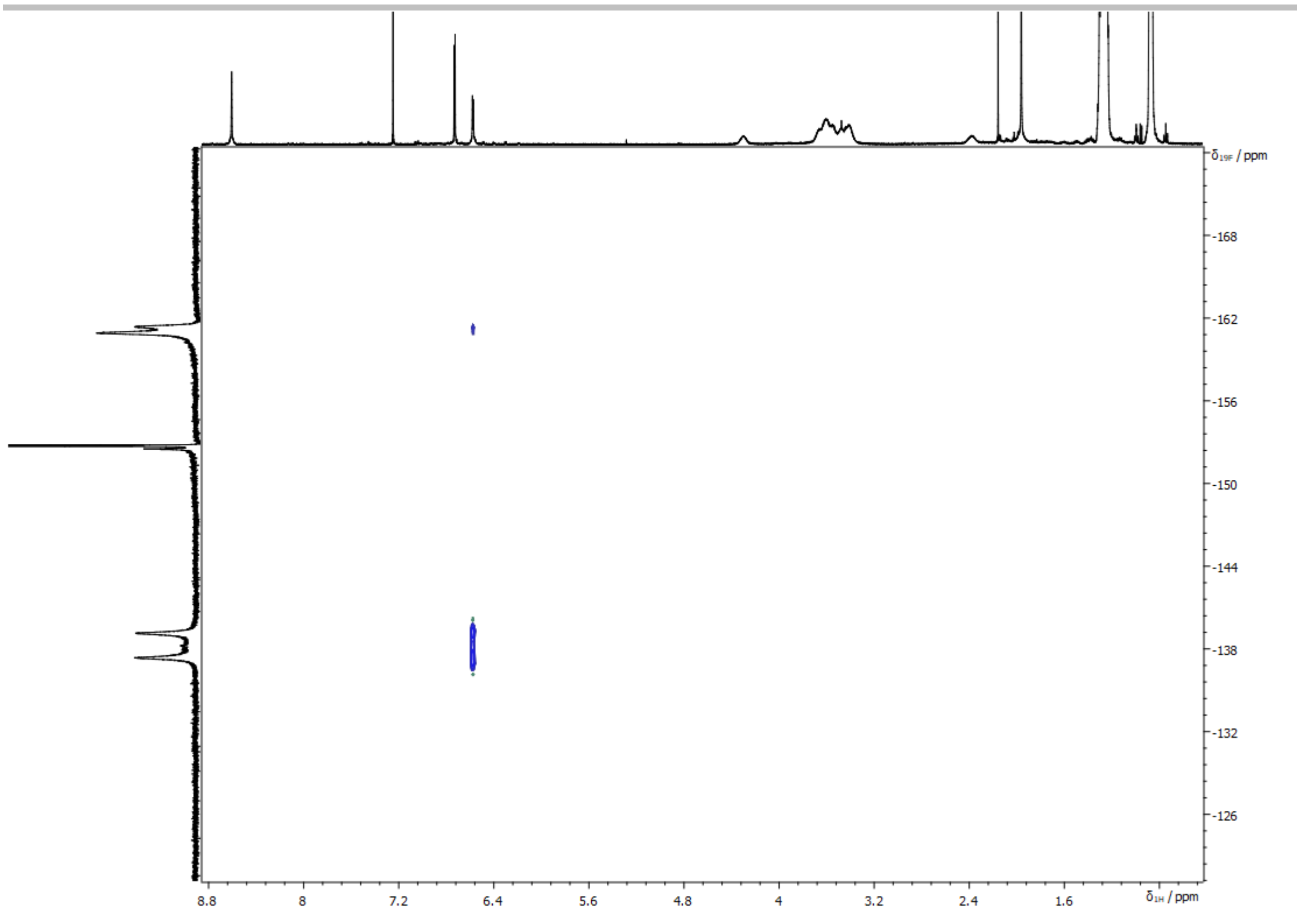

Figure S 113. The ^{1}H-^{19}F HOESY spectrum of **21b-Pb** ([D]chloroform, 300 K, 500 MHz).

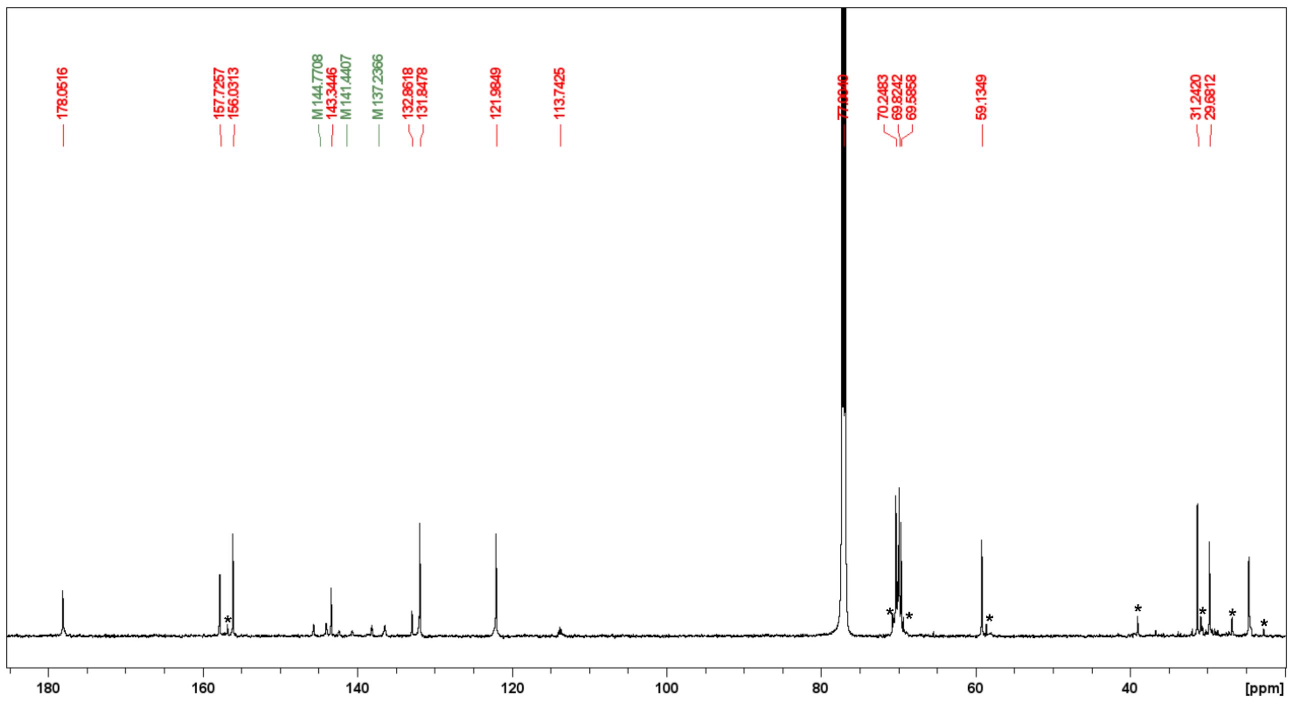

Figure S 114. The ^{13}C NMR spectrum of **21b-Pb** ([D]chloroform, 300 K, 151 MHz). Signals corresponding to impurities were marked with asterisks.

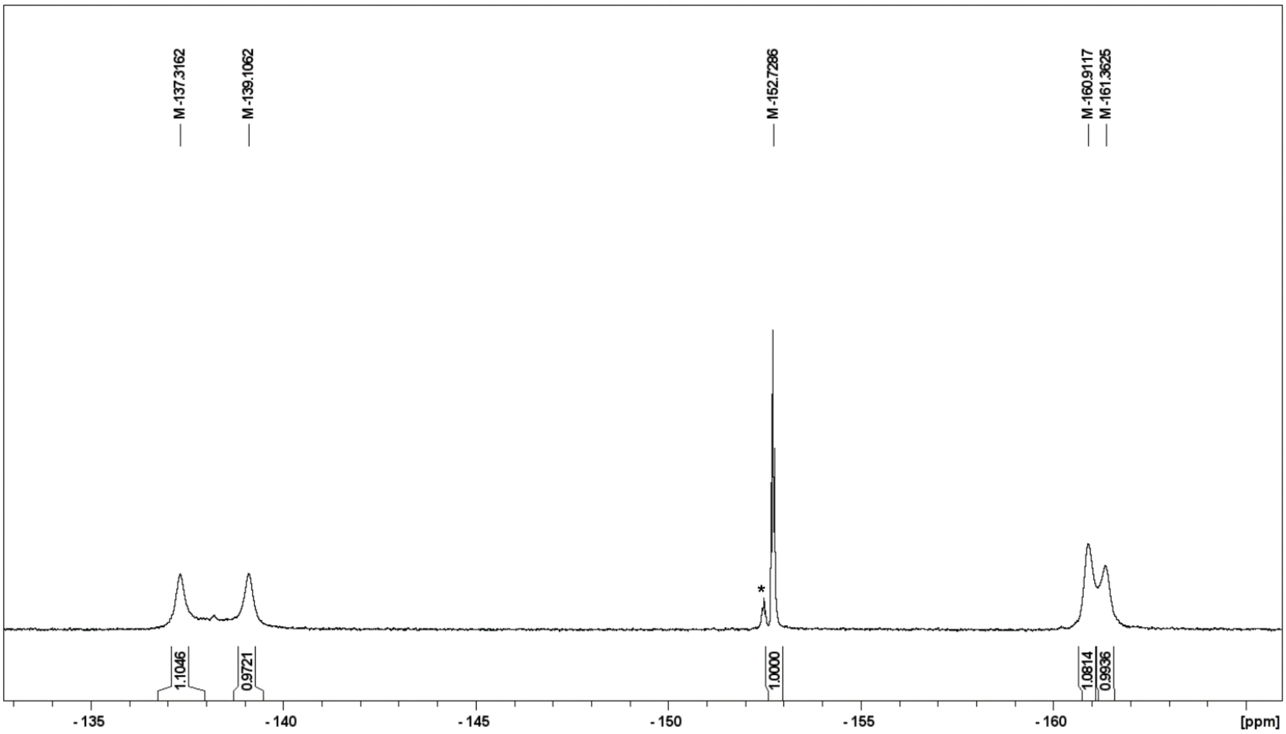

Figure S 115. The ^{19}F NMR spectrum of **21b-Pb** ([D]chloroform, 300 K, 471 MHz).

The NMR spectra of 21b-Cd

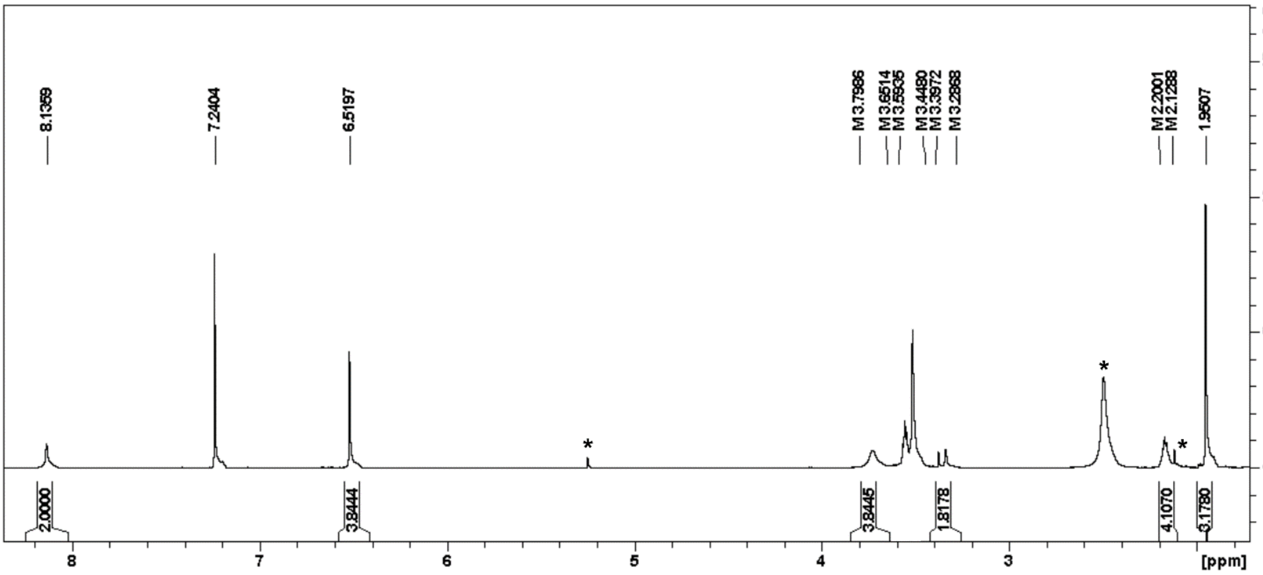

Figure S 116. The ^{1}H NMR spectrum of **21b-Cd** ([D]chloroform, 300 K, 500 MHz). Signals corresponding to impurities were marked with asterisks.

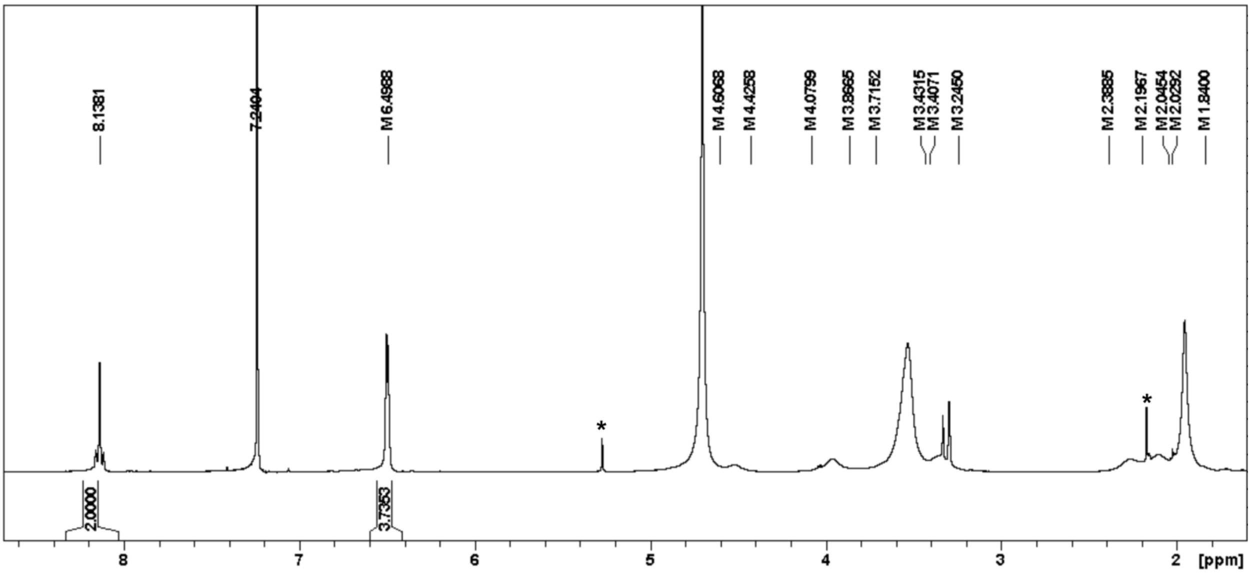

Figure S 117. The ^{1}H NMR spectrum of **21b-Cd** ([D]chloroform, 220 K, 600 MHz). Signals corresponding to impurities were marked with asterisks.

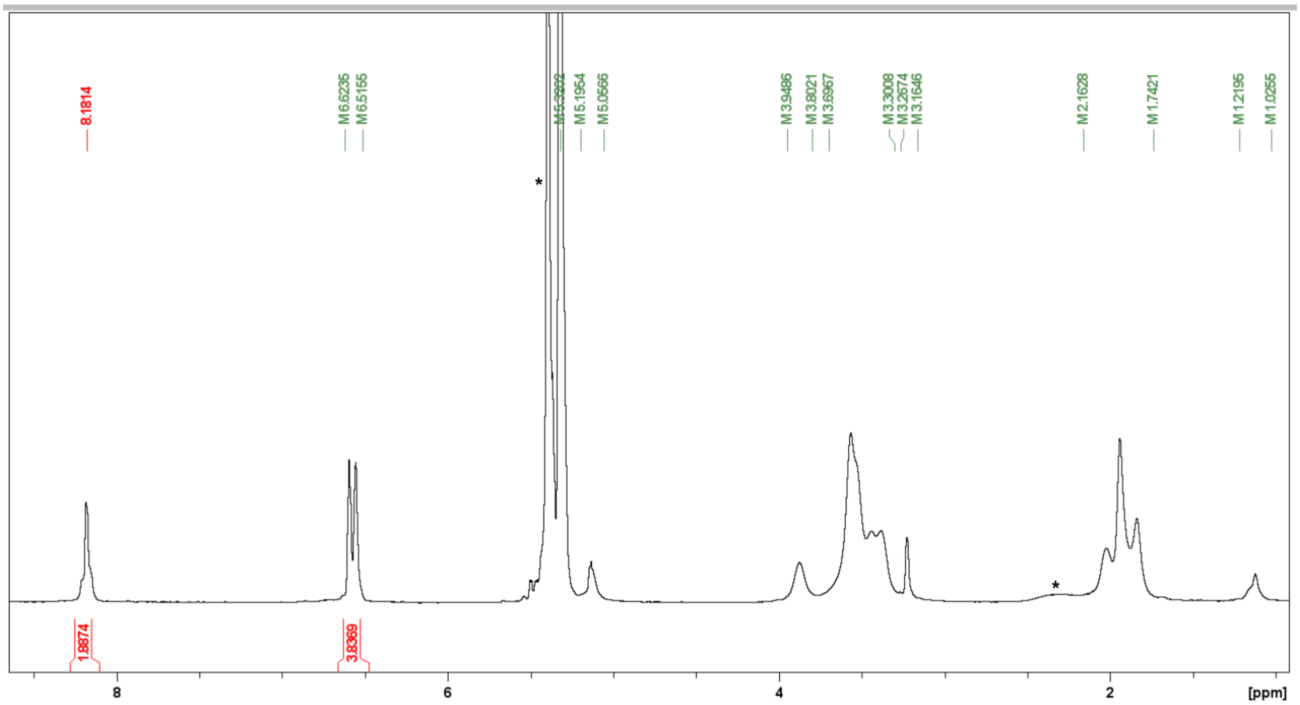

Figure S 118. The ^{1}H NMR spectrum of **21b-Cd** ([D_2]dichloromethane, 178 K, 500 MHz). Signals corresponding to impurities were marked with asterisks.

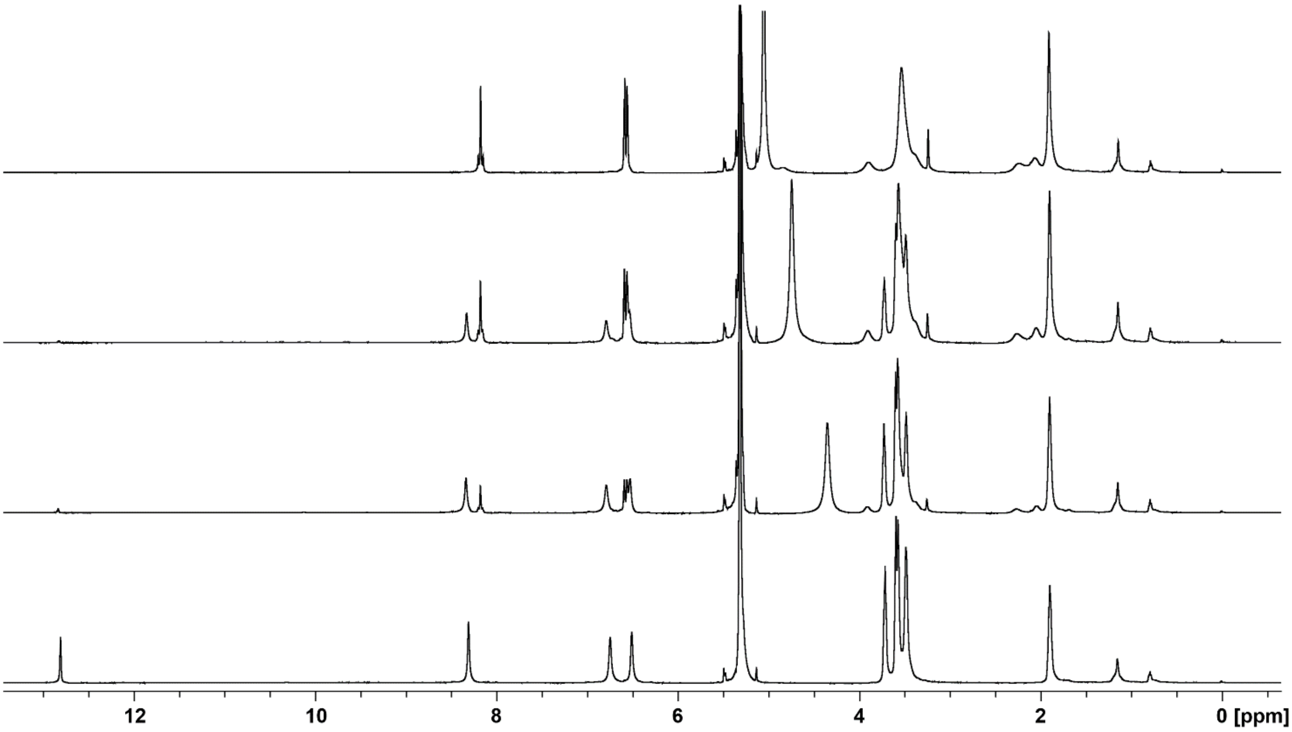

Figure S 119. The ^{1}H NMR spectra recorded in the course of the titration of **15b-H** with $Cd(OAc)_2$ in CD_3OD ([D_2]dichloromethane, 178 K, 600 MHz). The specrtum on the top corresponds to **21b-Cd**, the spectrum at the bottom corresponds to **15b-H**.

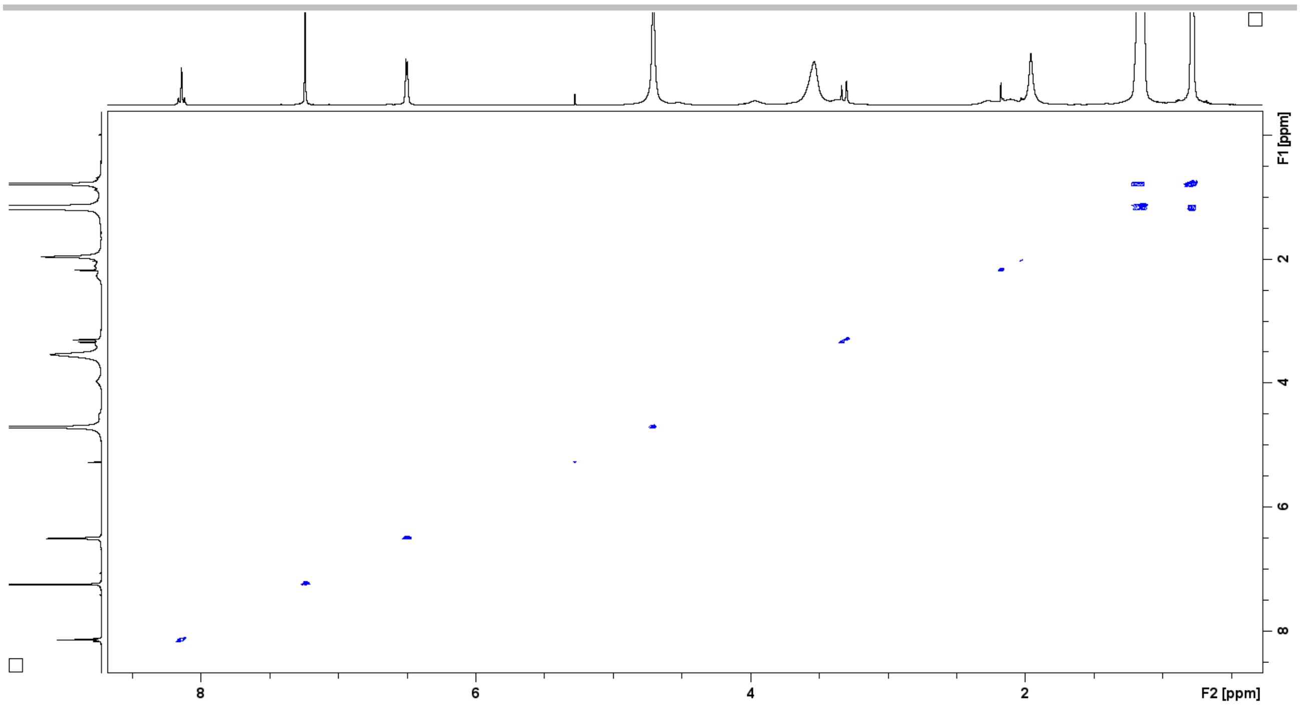

Figure S 120. The ${}^{1}H$-${}^{1}H$ COSY spectrum of **21b-Cd** ([D]chloroform, 220 K, 600 MHz).

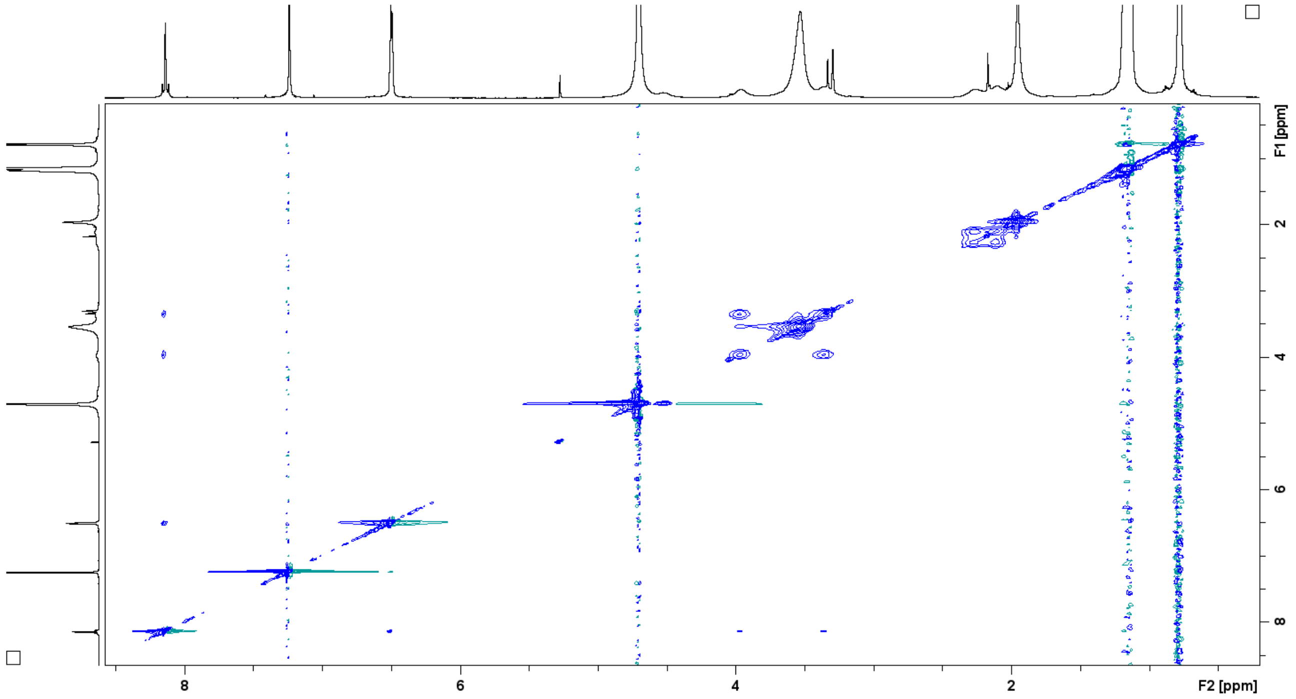

Figure S 121. The ${}^{1}H$-${}^{1}H$ NOESY spectrum of **21b-Cd** ([D]chloroform, 220 K, 600 MHz).

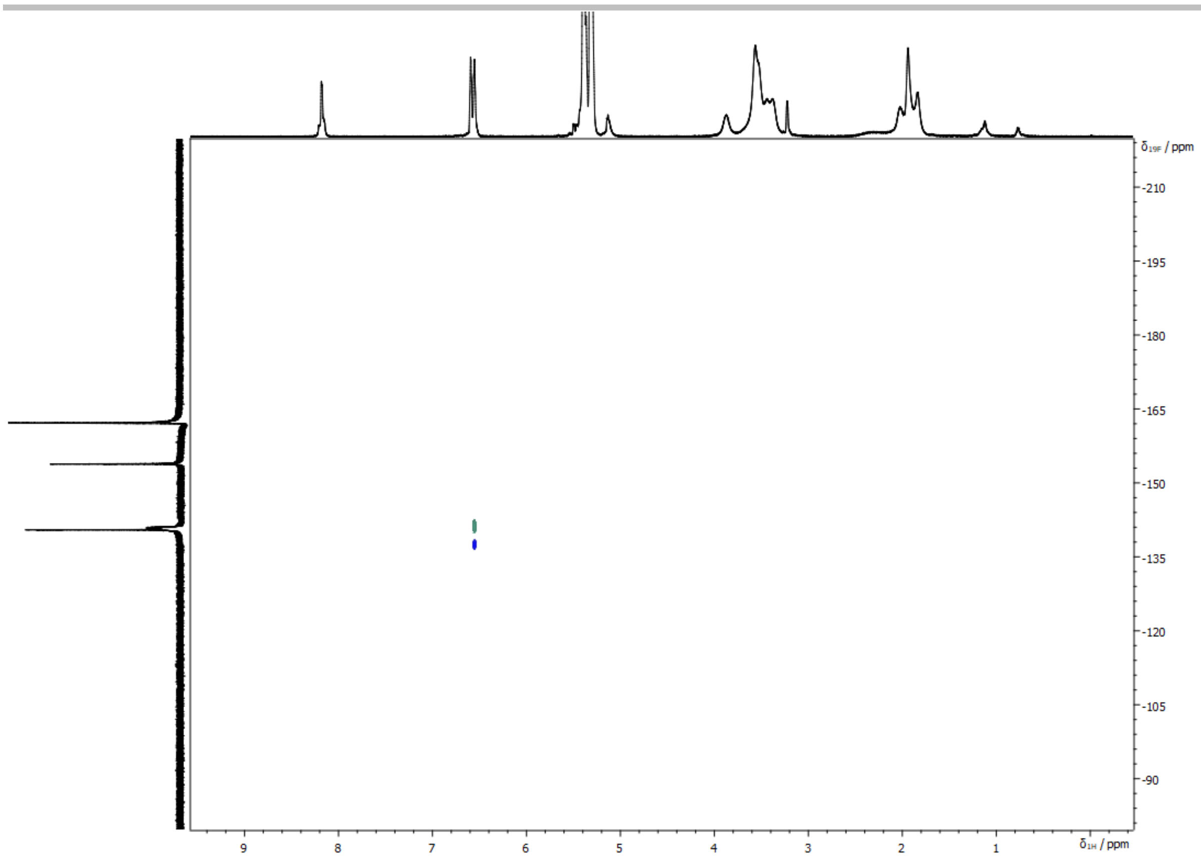

Figure S 122. The ^{1}H-^{19}F HOESY spectrum of **21b-Cd** ([D$_2$]dichloromethane, 178 K, 500 MHz).

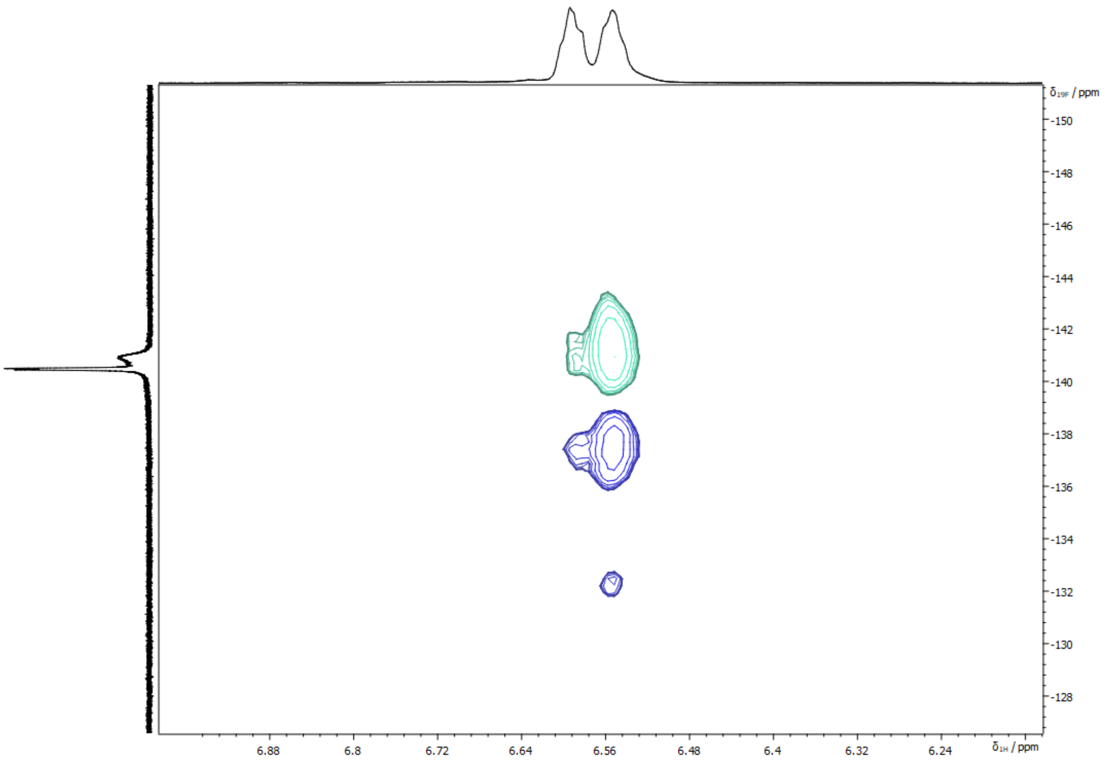

Figure S 123. The aromatic region of the ^{1}H-^{19}F HOESY spectrum of **21b-Cd** ([D$_2$]dichloromethane, 178 K, 500 MHz).

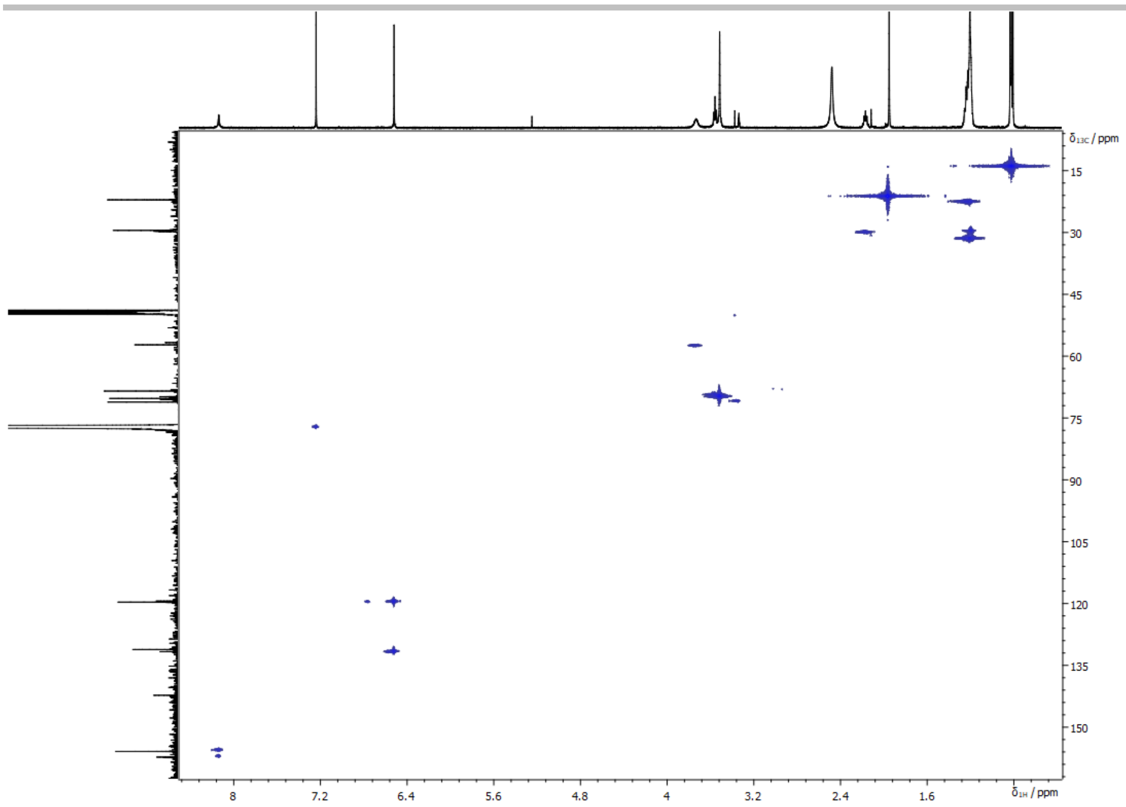

Figure S 124. The ^{1}H-^{13}C HMQC spectrum of **21b-Cd** ([D]chloroform, 300 K, 600 MHz).

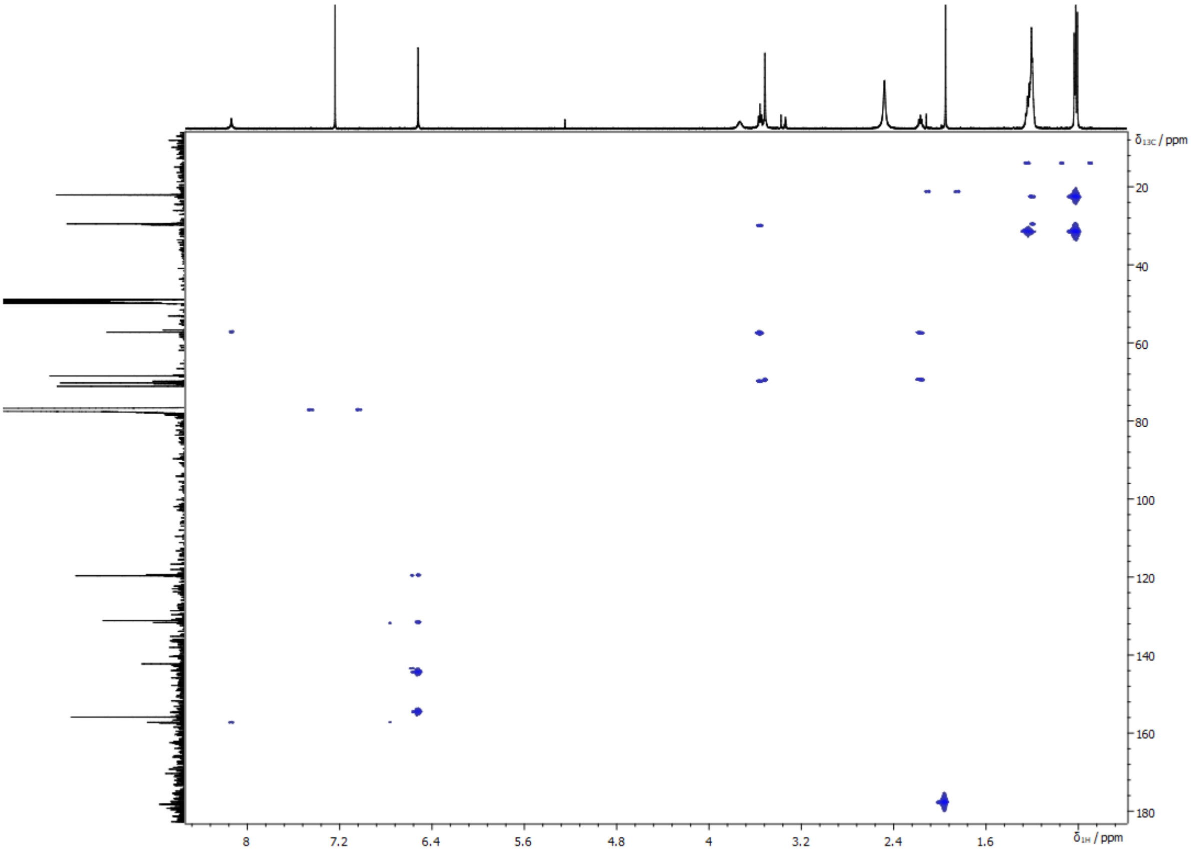

Figure S 125. The ^{1}H-^{13}C HMBC spectrum of **21b-Cd** ([D]chloroform, 300 K, 600 MHz).

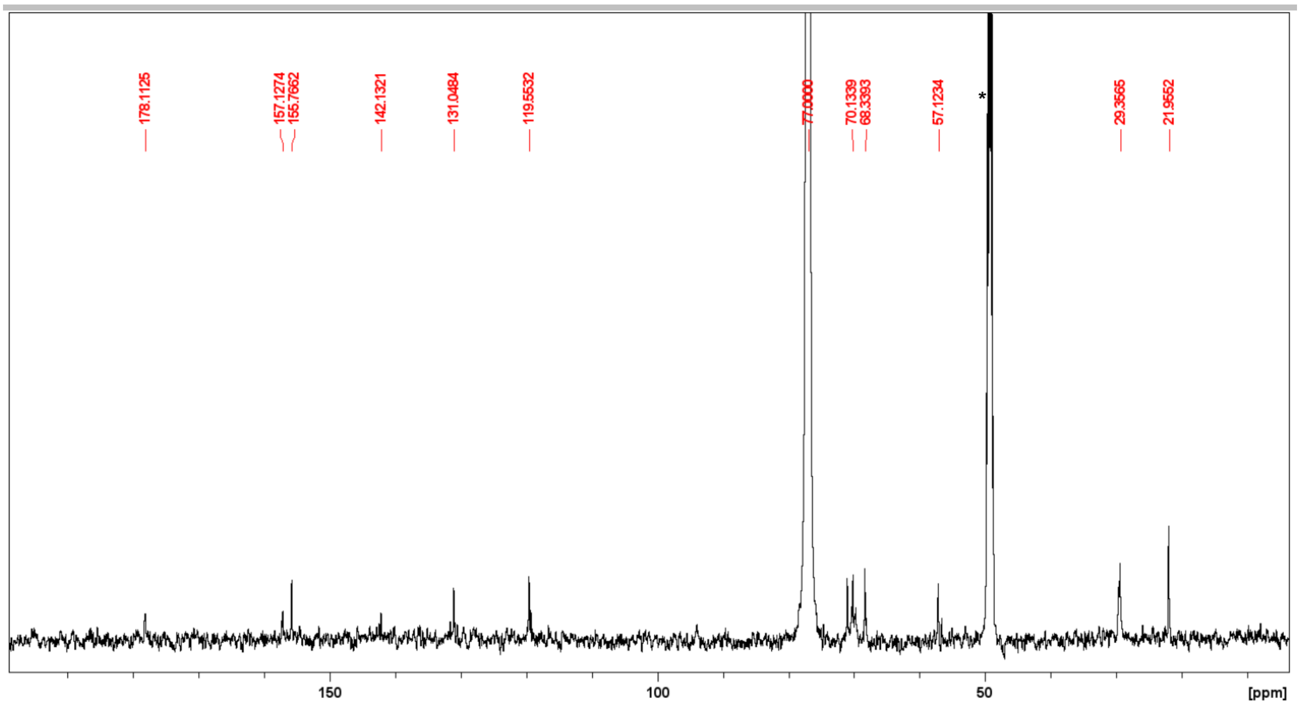

Figure S 126. The ^{13}C NMR spectrum of **21b-Cd** ([D]chloroform, 300 K, 125 MHz). Signals corresponding to impurities were marked with asterisks.

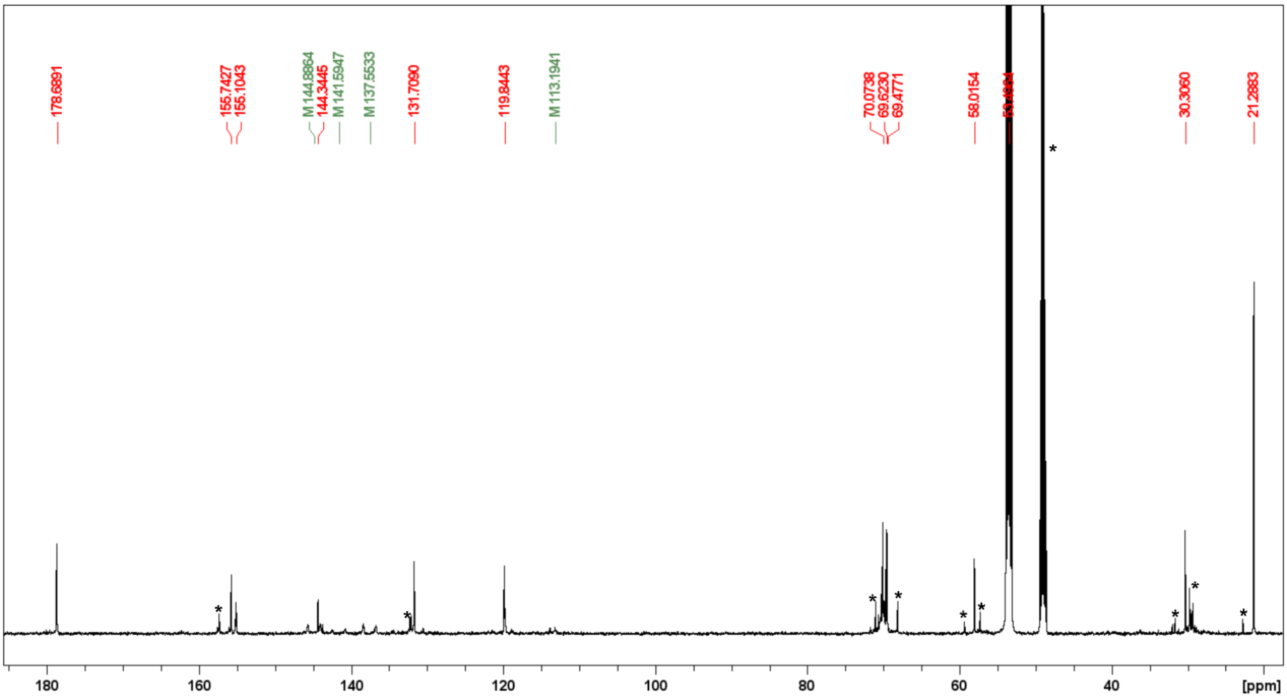

Figure S 127. The ^{13}C NMR spectrum of **21b-Cd** ([D$_2$]dichloromethane, 300 K, 151 MHz). Signals corresponding to impurities were marked with asterisks.

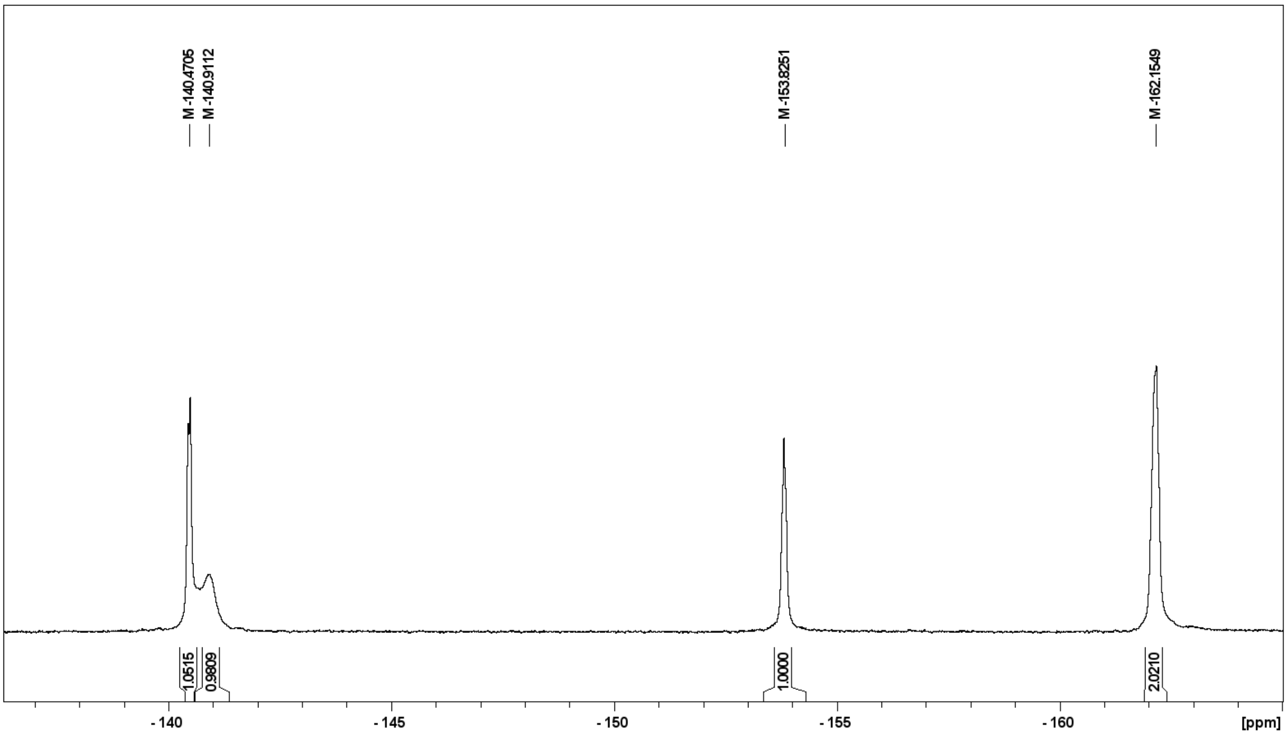

Figure S 128. The ^{19}F NMR spectrum of **21b-Cd** ([D2]dichloromethane, 178 K, 471 MHz).

The NMR spectra of 21b-Hg

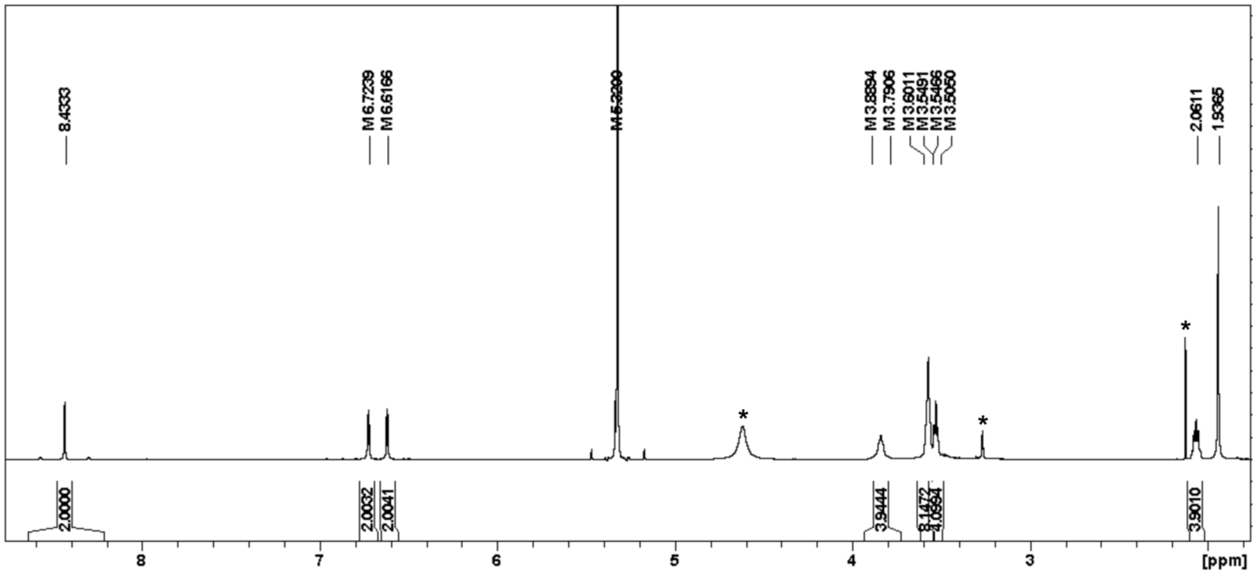

Figure S 129. The ^{1}H NMR spectrum of **21b-Hg** ([D$_2$]dichloromethane, 220 K, 600 MHz). Signals corresponding to impurities were marked with asterisks.

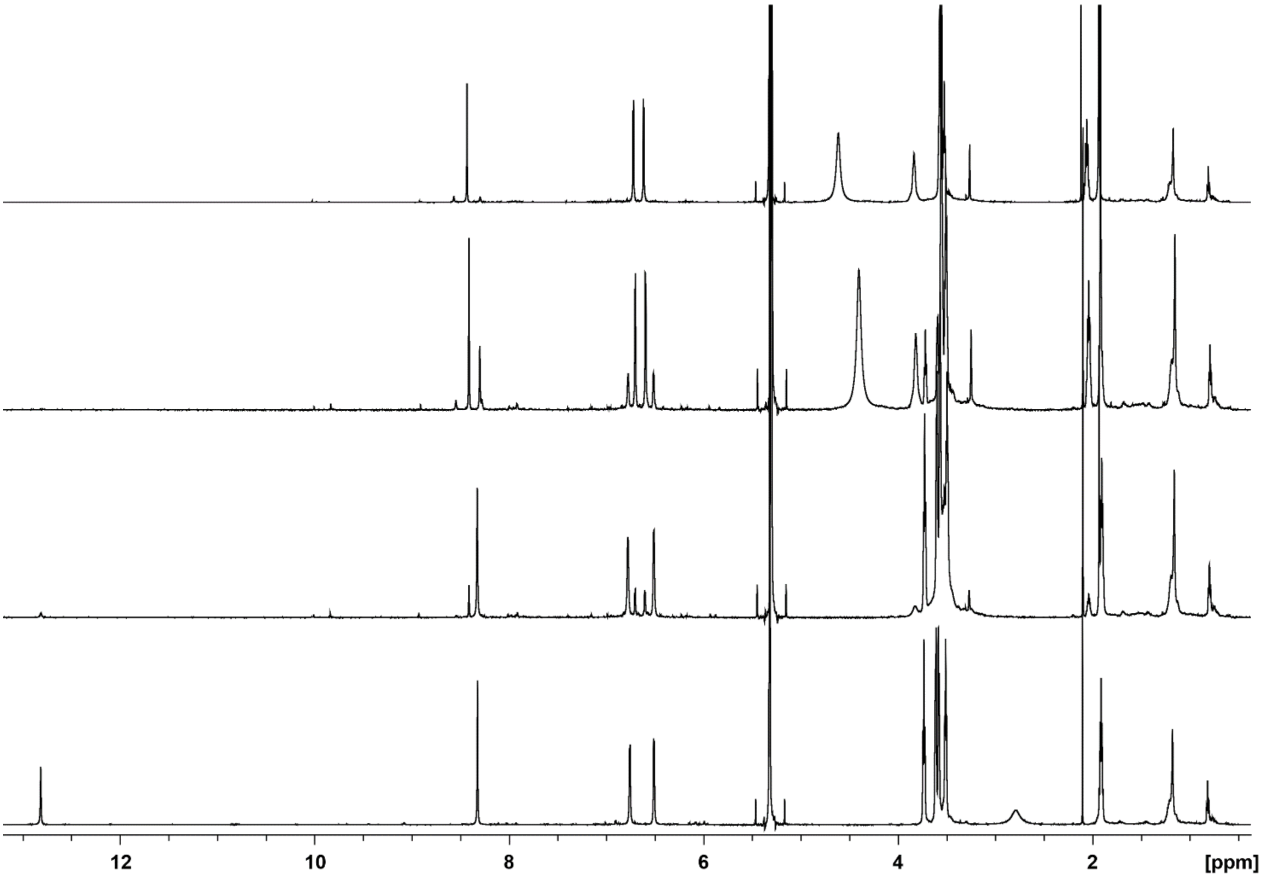

Figure S 130. The ^{1}H NMR spectra recorded in the course of the titration of **15b-H** with Hg^{2+} ($Hg(OAc)_2$ in CD_3OD) ([D$_2$]dichloromethane, 220 K, 600 MHz). The spectrum on the top corresponds to **21b-Hg**, the spectrum at the bottom corresponds to **15b-H**.

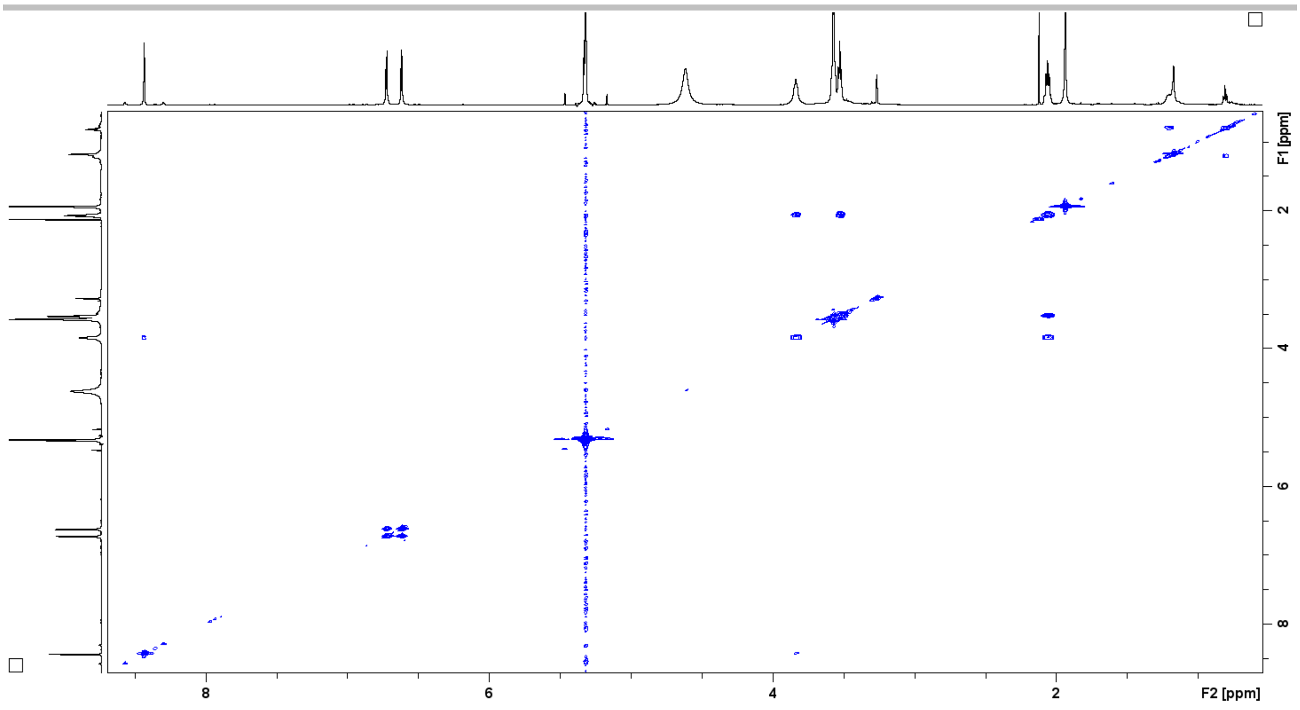

Figure S 131. The ^{1}H-^{1}H COSY spectrum of **21b-Hg** ([D_2]dichloromethane, 220 K, 600 MHz).

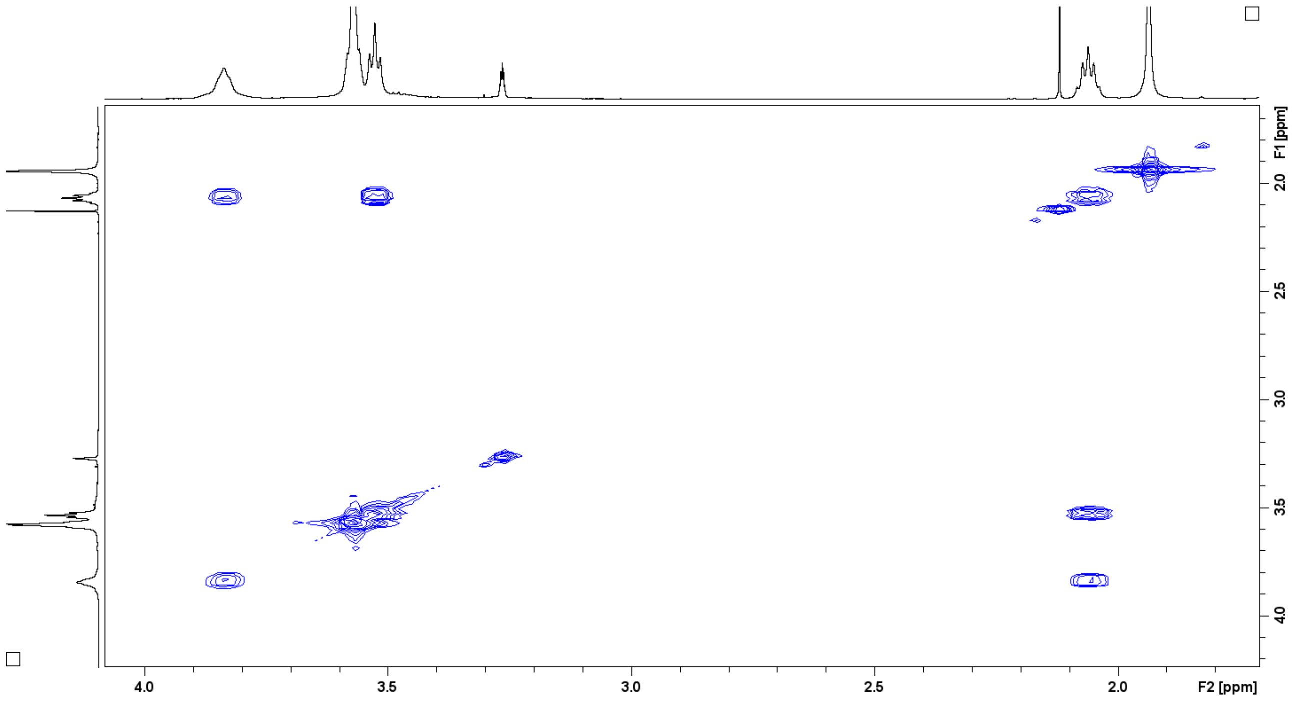

Figure S 132. The oligo(ethylene glycol) chain region of the ^{1}H-^{1}H COSY spectrum of **21b-Hg** ([D_2]dichloromethane, 220 K, 600 MHz).

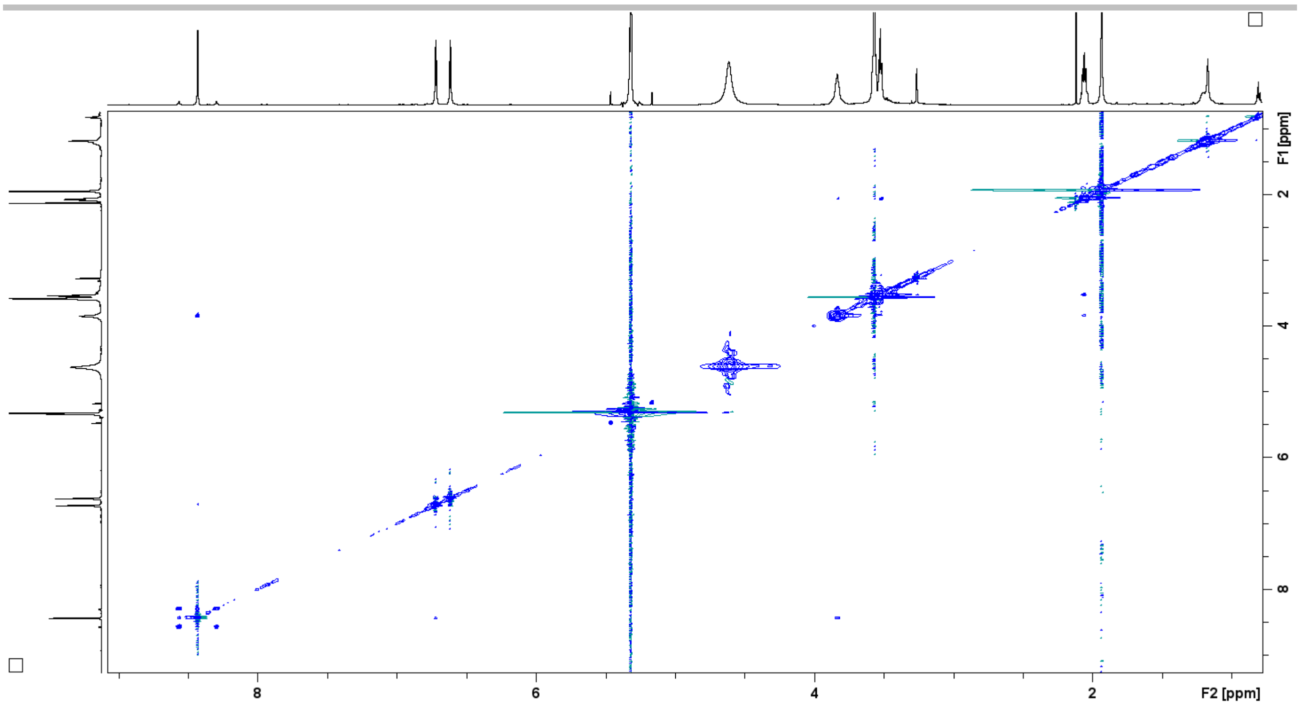

Figure S 133. The ^{1}H-^{1}H NOESY spectrum of **21b-Hg** ([D$_2$]dichloromethane, 220 K, 600 MHz).

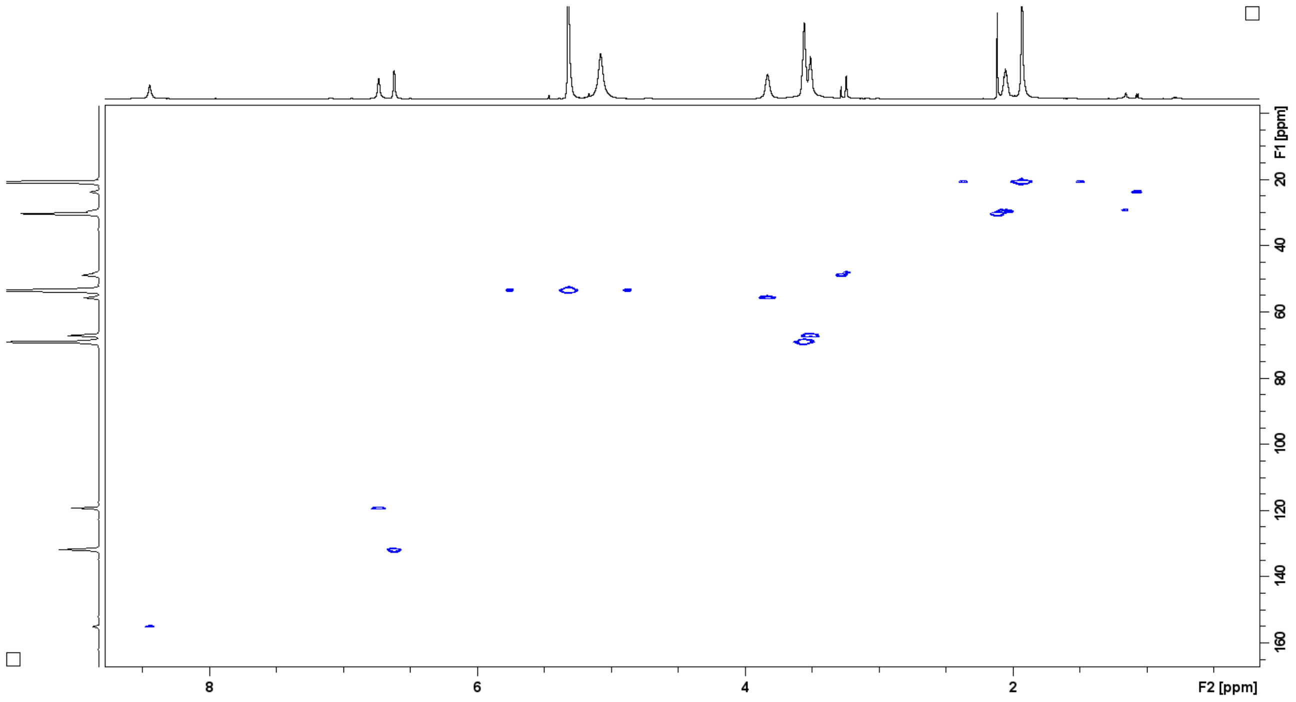

Figure S 134. The ^{1}H-^{13}C HMQC spectrum of **21b-Hg** ([D$_2$]dichloromethane, 220 K, 600 MHz).

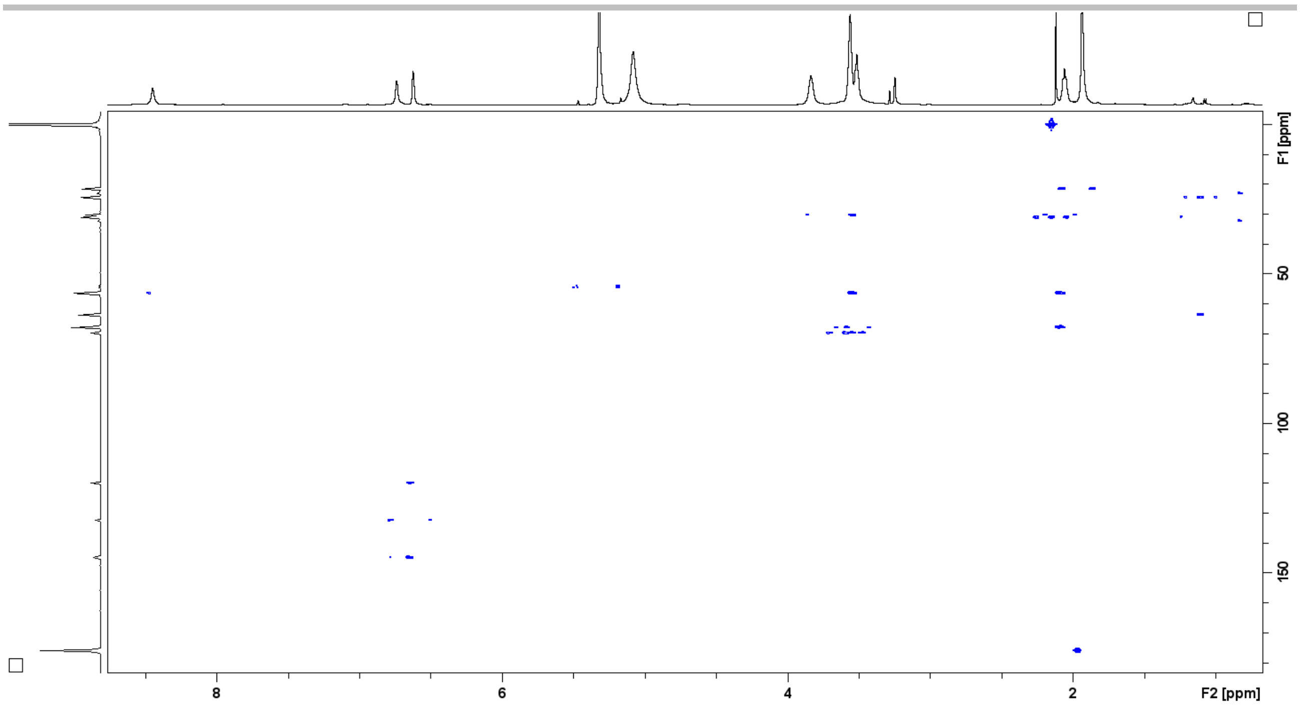

Figure S 135. The ^{1}H-^{13}C HMBC spectrum of **21b-Hg** ([D_2]dichloromethane, 220 K, 600 MHz).

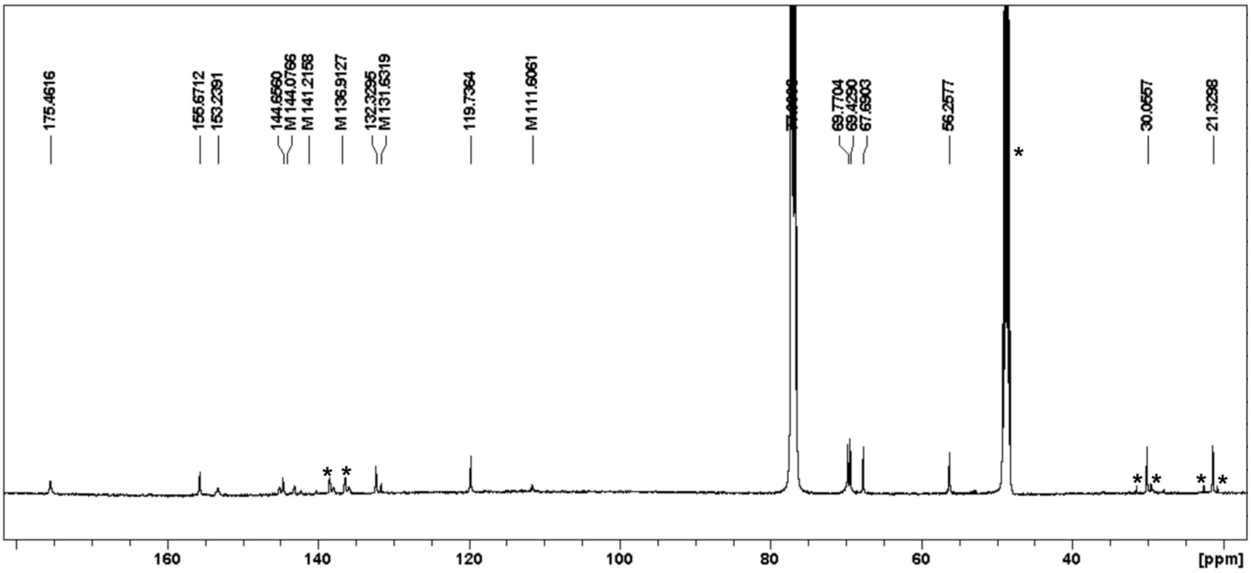

Figure S 136. The ^{13}C NMR spectrum of **21b-Hg** ([D]chloroform, 300 K, 125 MHz). Signals corresponding to impurities were marked with asterisks.

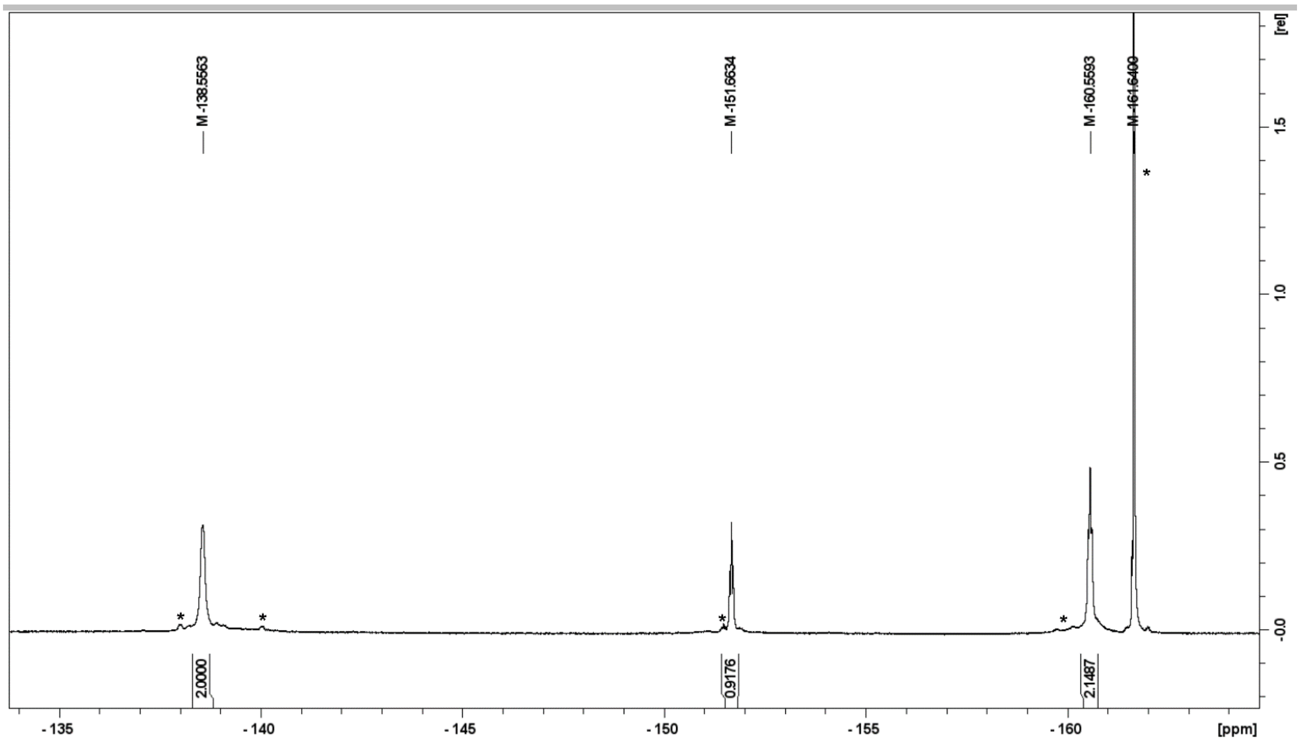

Figure S 137. The ^{19}F NMR spectrum of **21b-Hg** ([D]chloroform, 233 K, 471 MHz). Signals corresponding to impurities were marked with asterisks.

The NMR spectra of 22a-Zn

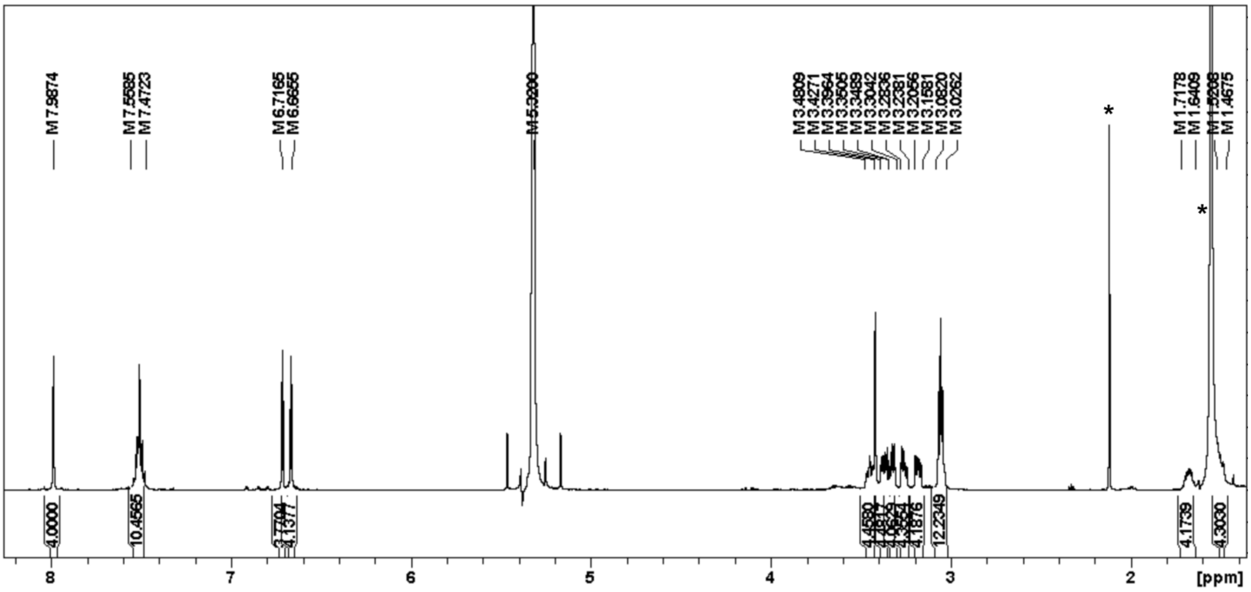

Figure S 138. The ^{1}H NMR spectrum of **22a-Zn** ([D_2]dichloromethane, 300 K, 600 MHz). Signals corresponding to impurities were marked with asterisks.

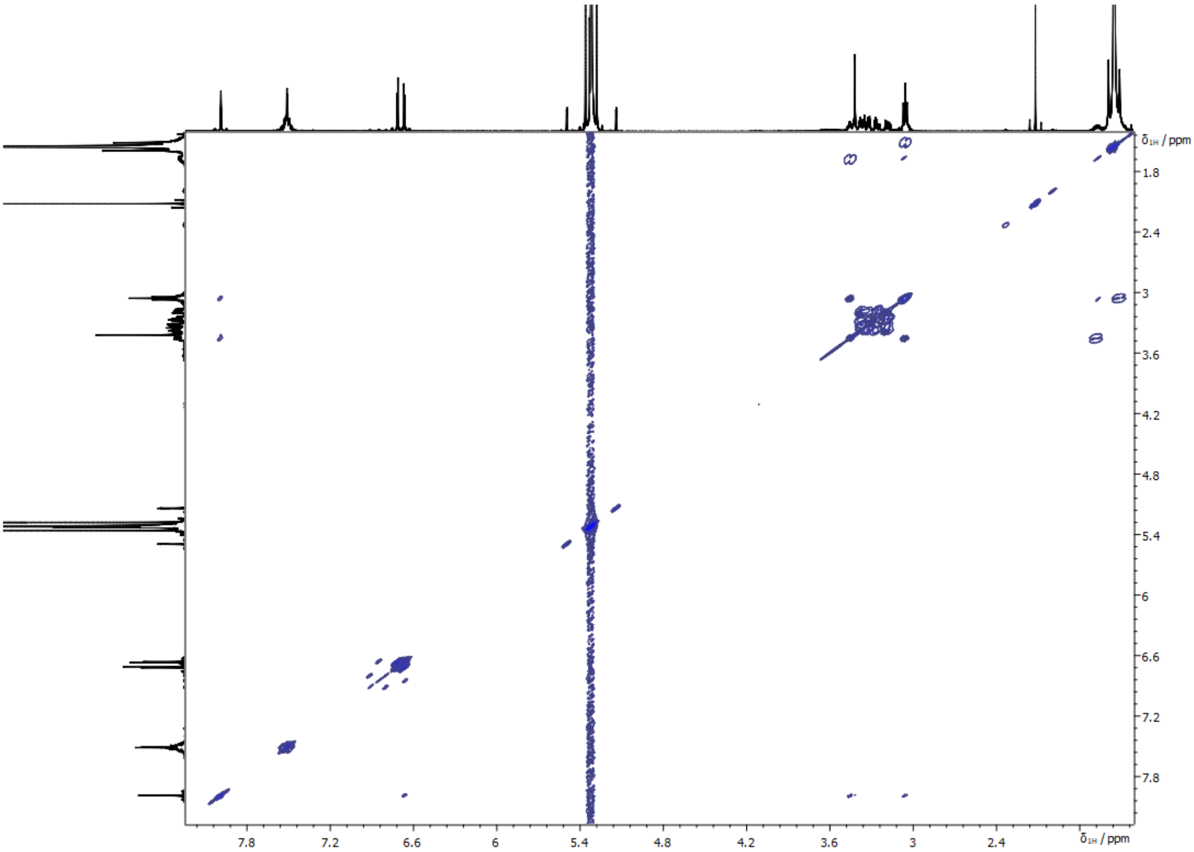

Figure S 139. The ^{1}H-^{1}H COSY spectrum of **22a-Zn** ([D_2]dichloromethane, 300 K, 500 MHz).

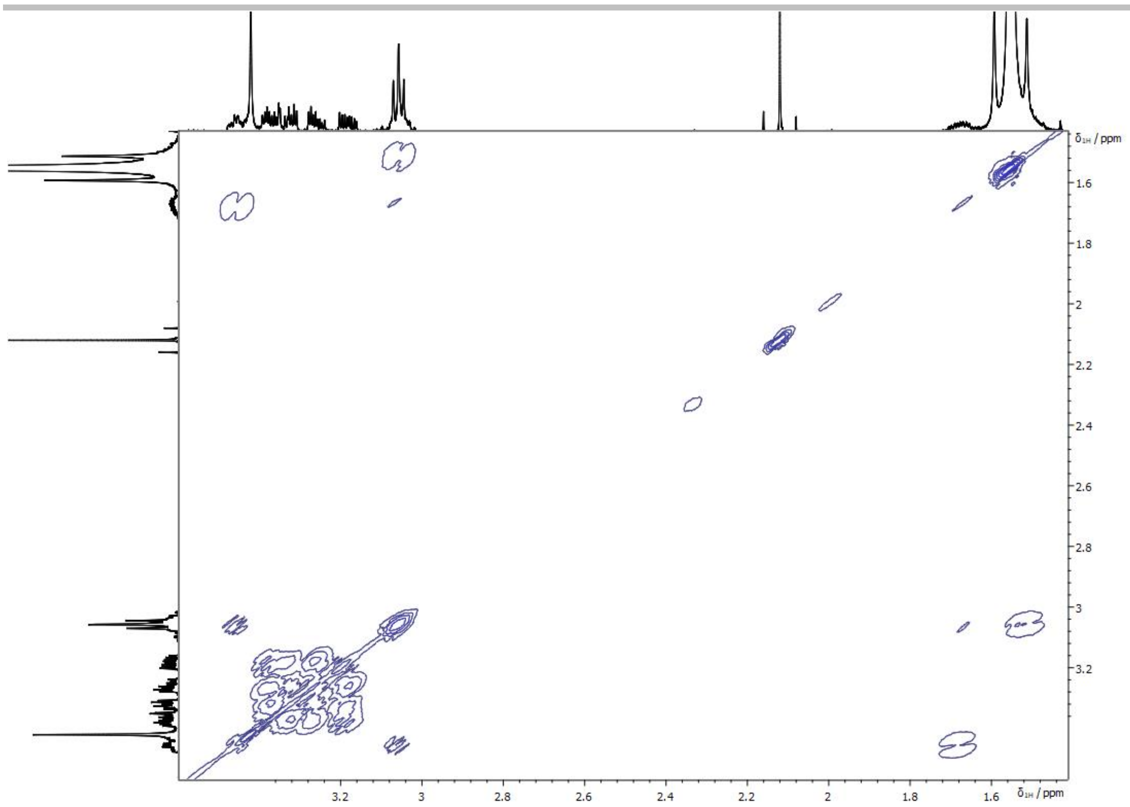

Figure S 140. The oligo(ethylene glycol) chain region of the ^{1}H-^{1}H COSY spectrum of **22a-Zn** ([D$_2$]dichloromethane, 300 K, 600 MHz).

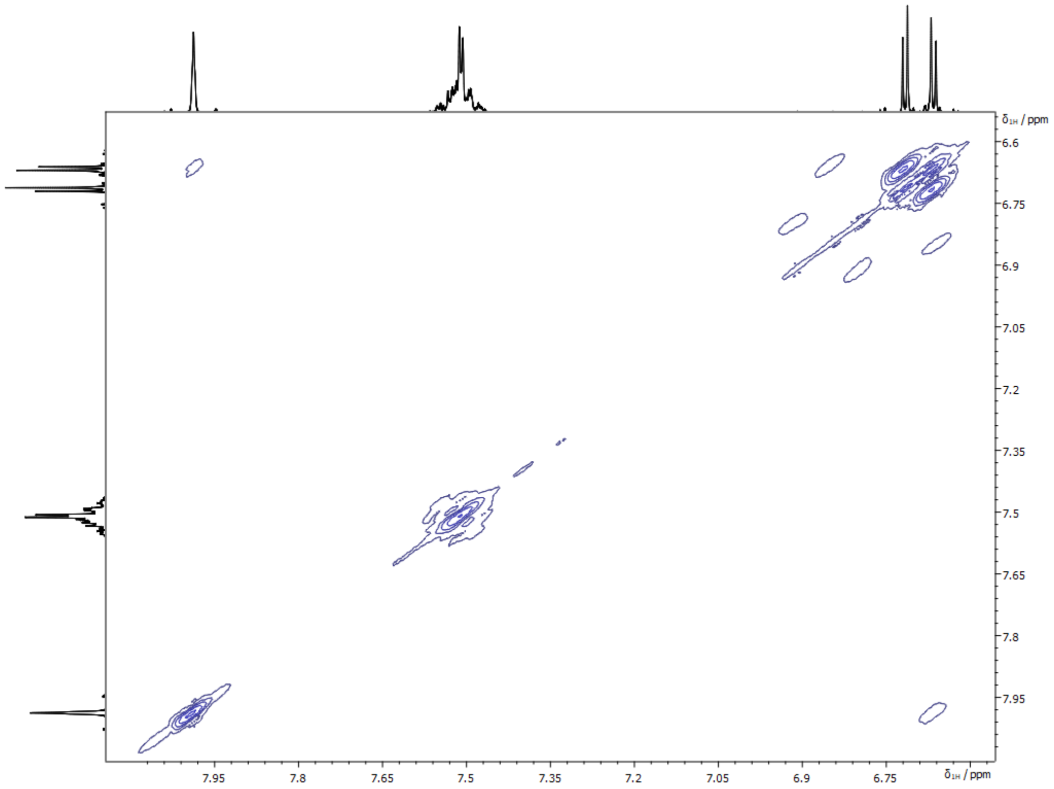

Figure S 141. The aromatic region of the ^{1}H-^{1}H COSY spectrum of **22a-Zn** ([D$_2$]dichloromethane, 300 K, 600 MHz).

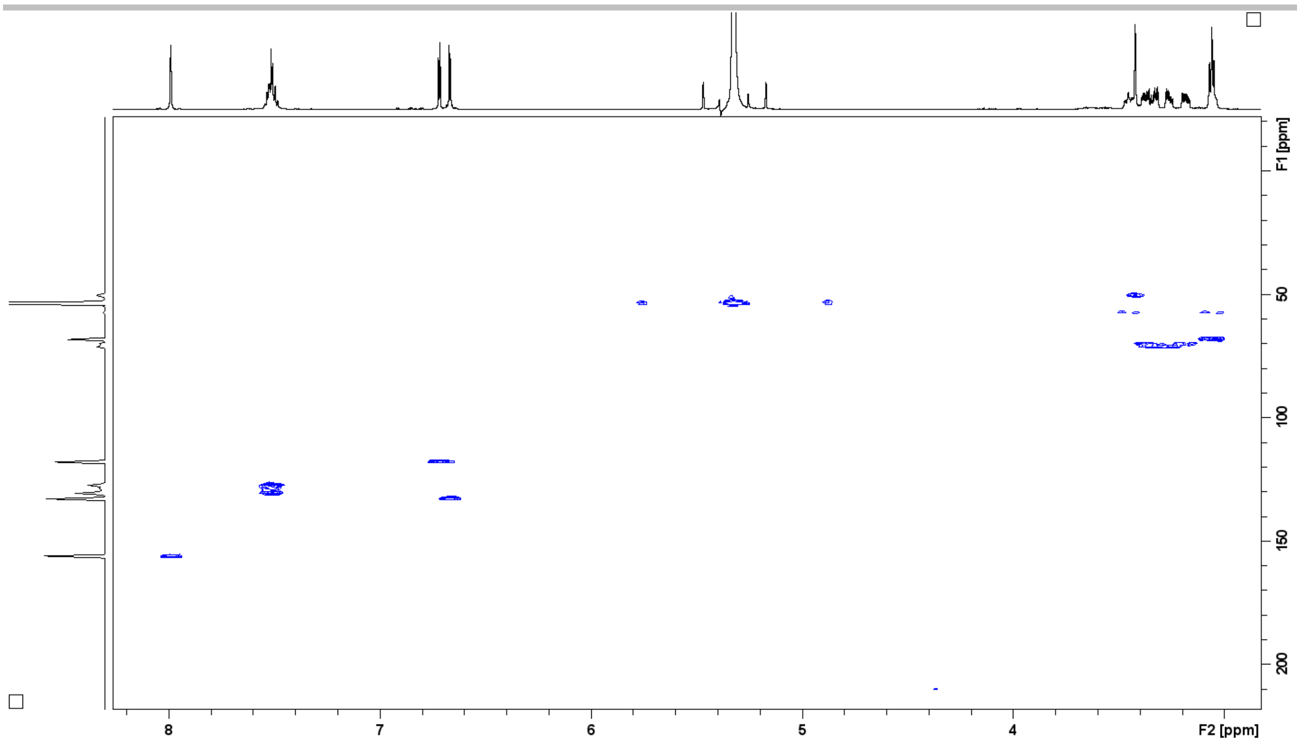

Figure S 142. The ^{1}H-^{13}C HMQC spectrum of **22a-Zn** ([D_2]dichloromethane, 300 K, 600 MHz).

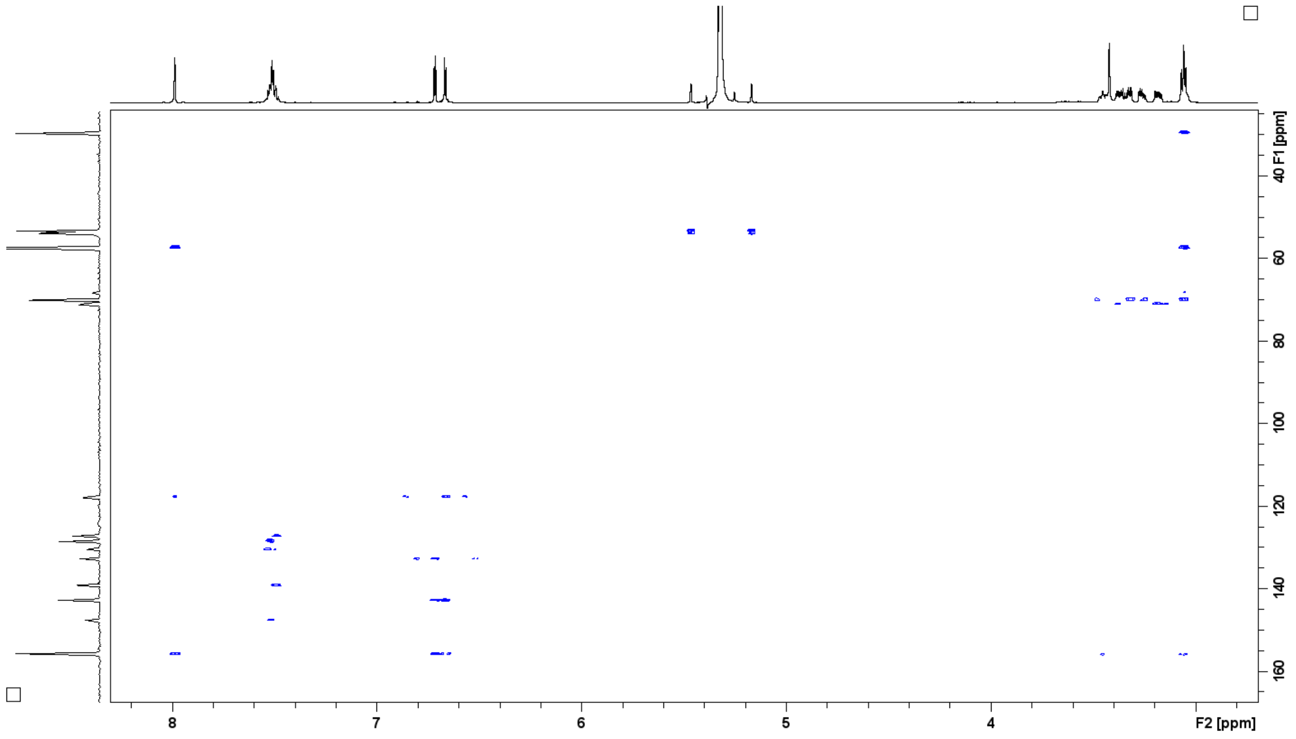

Figure S 143. The ^{1}H-^{13}C HMBC spectrum of **22a-Zn** ([D_2]dichloromethane, 300 K, 600 MHz).

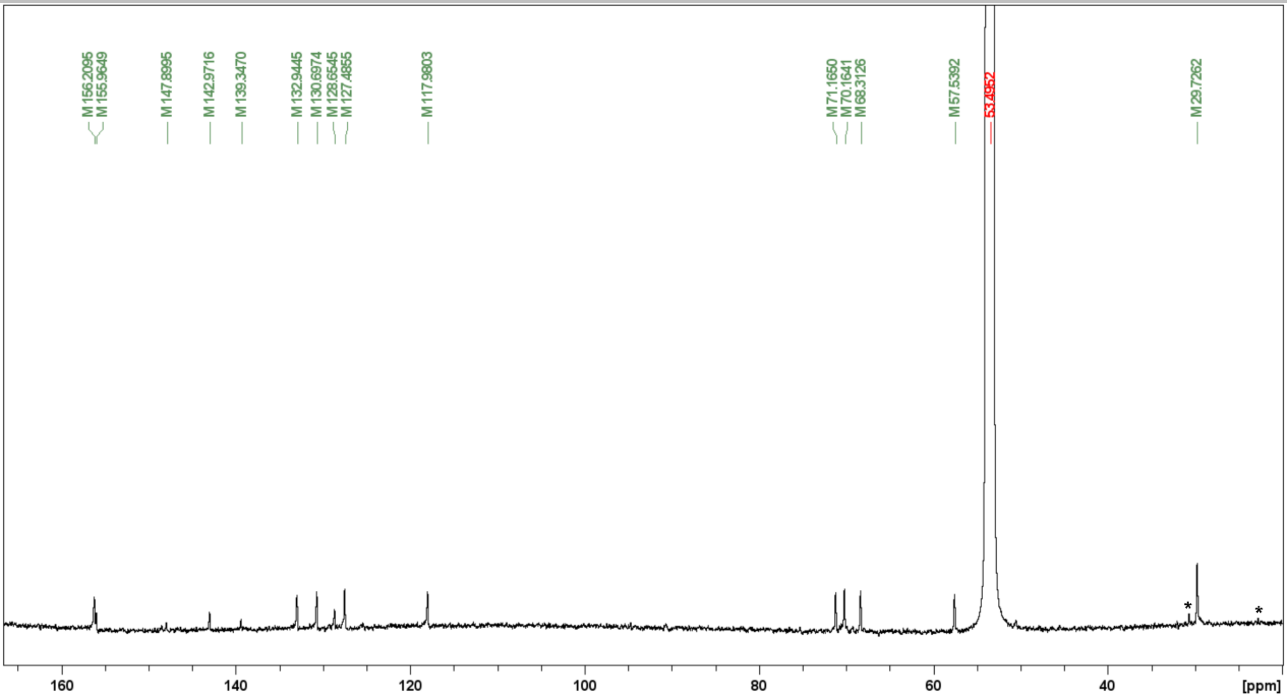

Figure S 144. The ^{13}C NMR spectrum of **22a-Zn** ([D₂]dichloromethane, 300 K, 125 MHz). Signals corresponding to impurities were marked with asterisks.

The NMR spectra of 22b-Zn

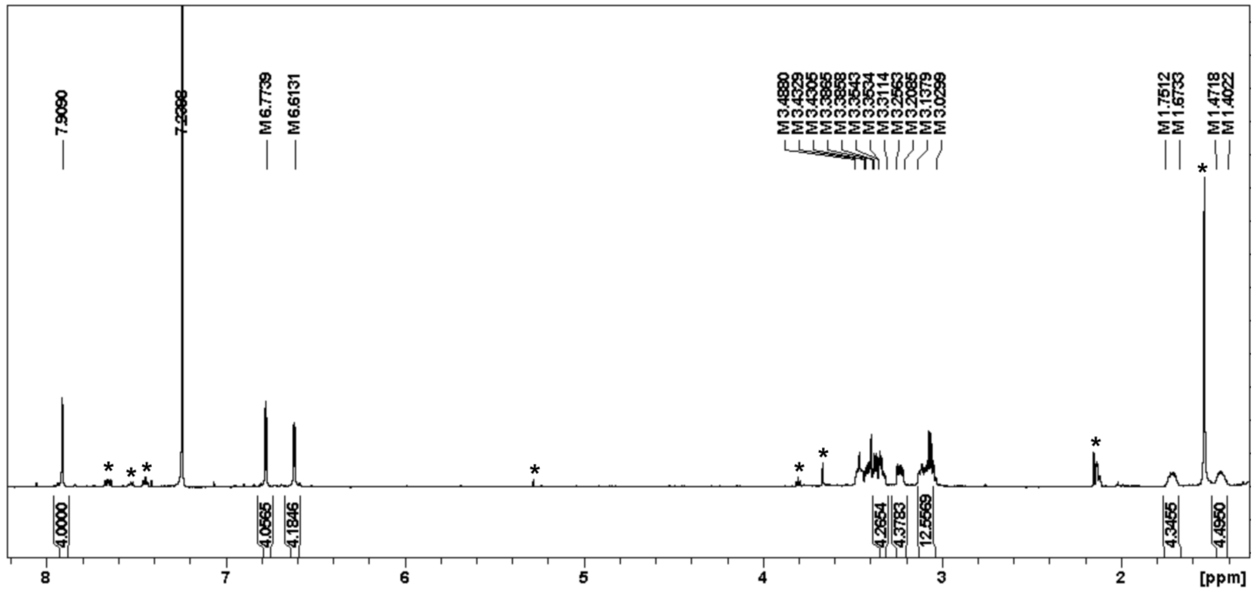

Figure S 145. The ^{1}H NMR spectrum of **22b-Zn** ([D]chloroform, 300 K, 600 MHz). Signals corresponding to impurities were marked with asterisks.

The NMR spectra of 22b-Cd

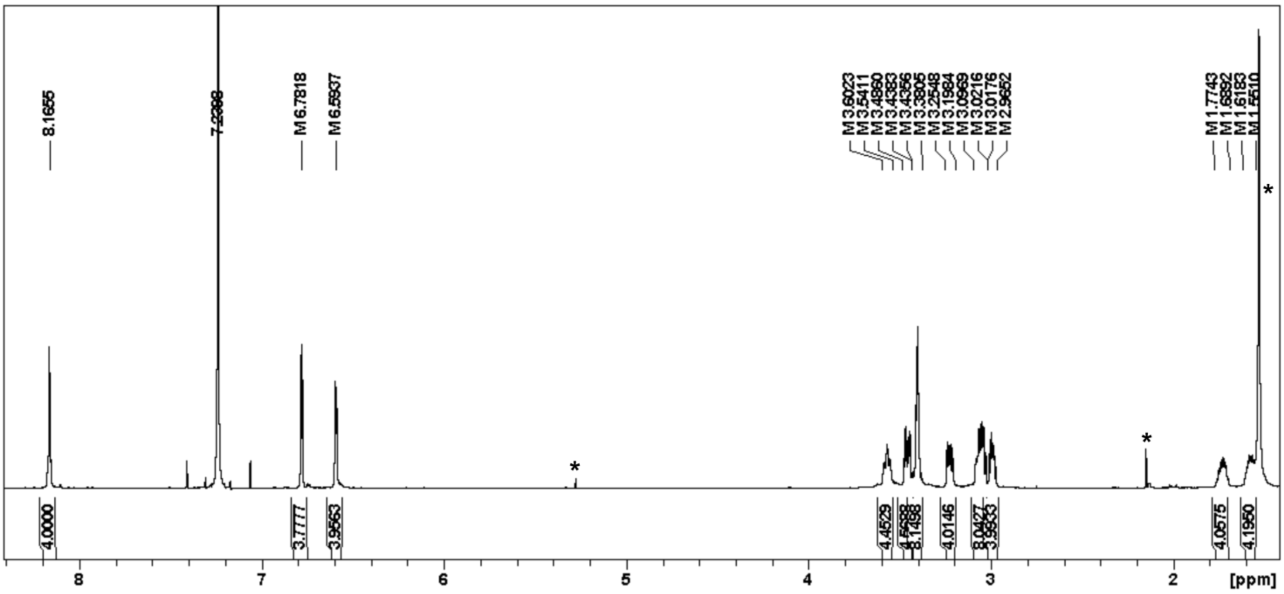

Figure S 146. The ^{1}H NMR spectrum of **22b-Cd** ([D]chloroform, 300 K, 600 MHz). Signals corresponding to impurities were marked with asterisks.

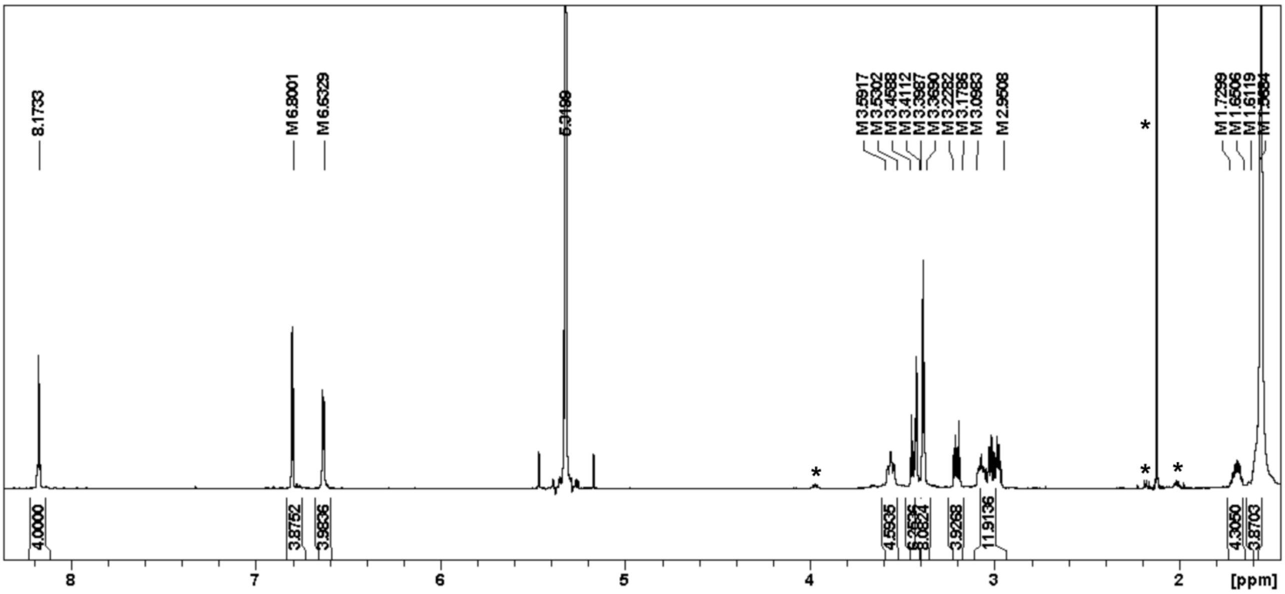

Figure S 147. The ^{1}H NMR spectrum of **22b-Cd** ([D_2]dichloromethane, 300 K, 600 MHz). Signals corresponding to impurities were marked with asterisks.

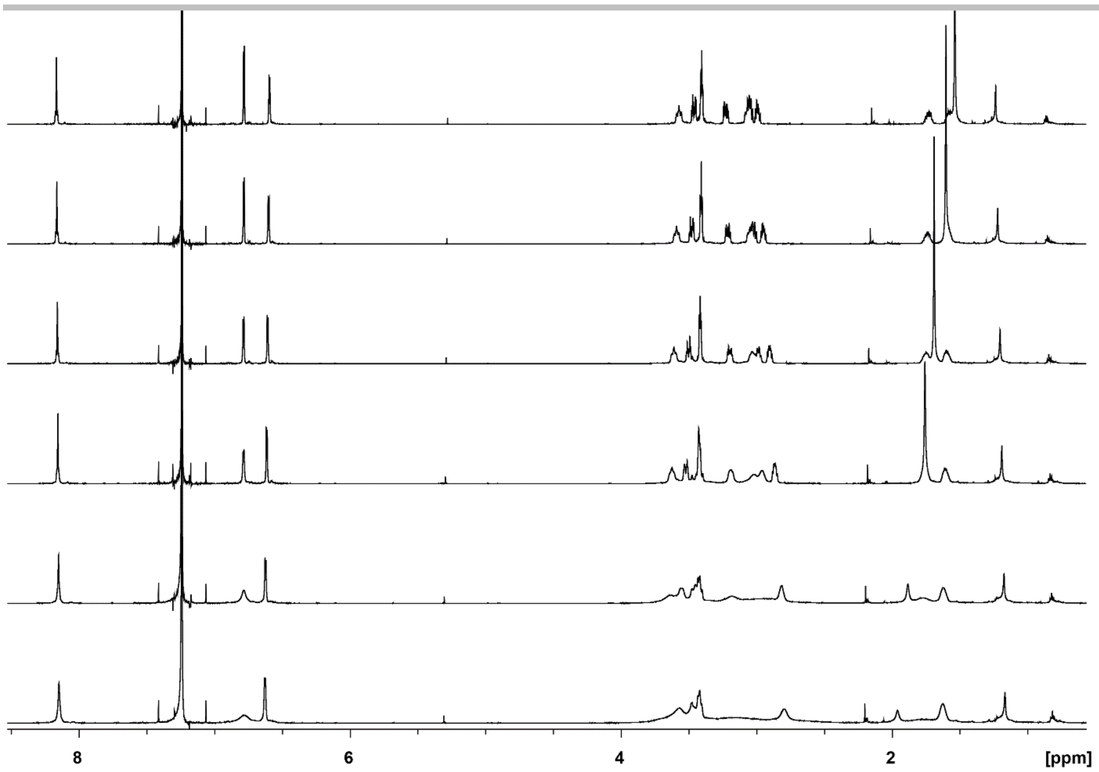

Figure S 148. ^{1}H NMR spectra of **22b-Cd** recorded in 300 K (top) – 210 K (bottom) temperature range every 20 K ([D]chloroform, 600 MHz).

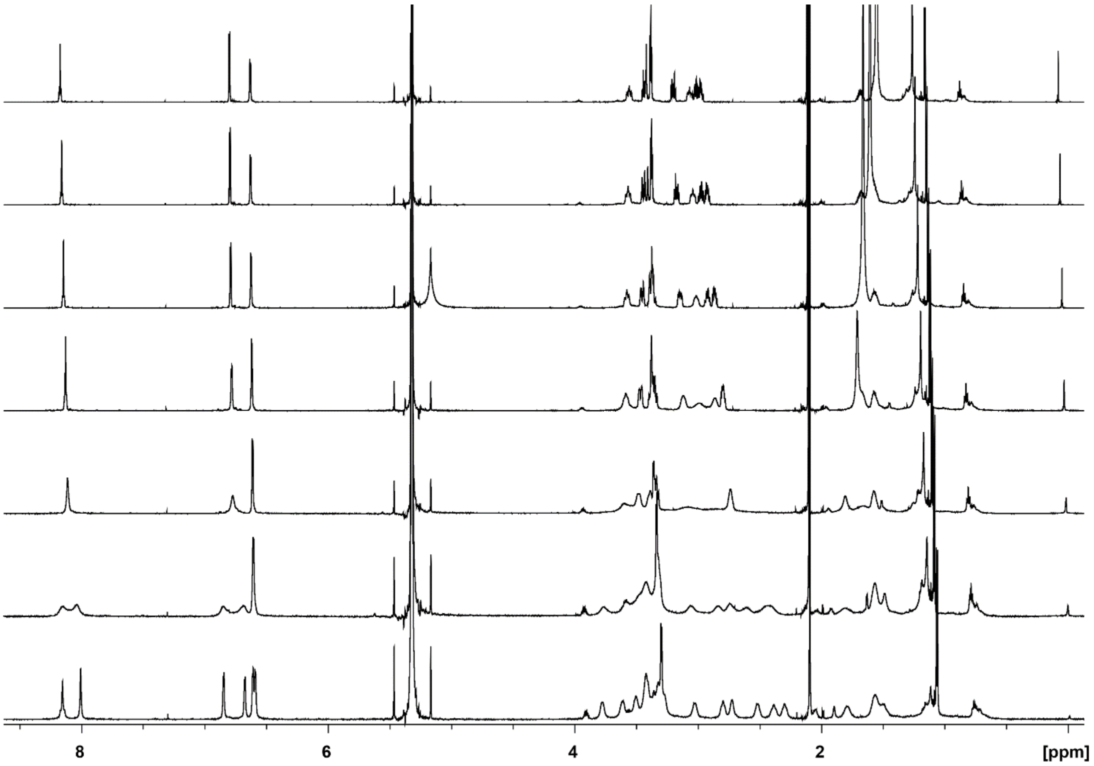

Figure S 149. ^{1}H NMR spectra of **22b-Cd** recorded in 300 K (top) – 180 K (bottom) temperature range every 20 K ([D$_2$]dichloromethane, 600 MHz).

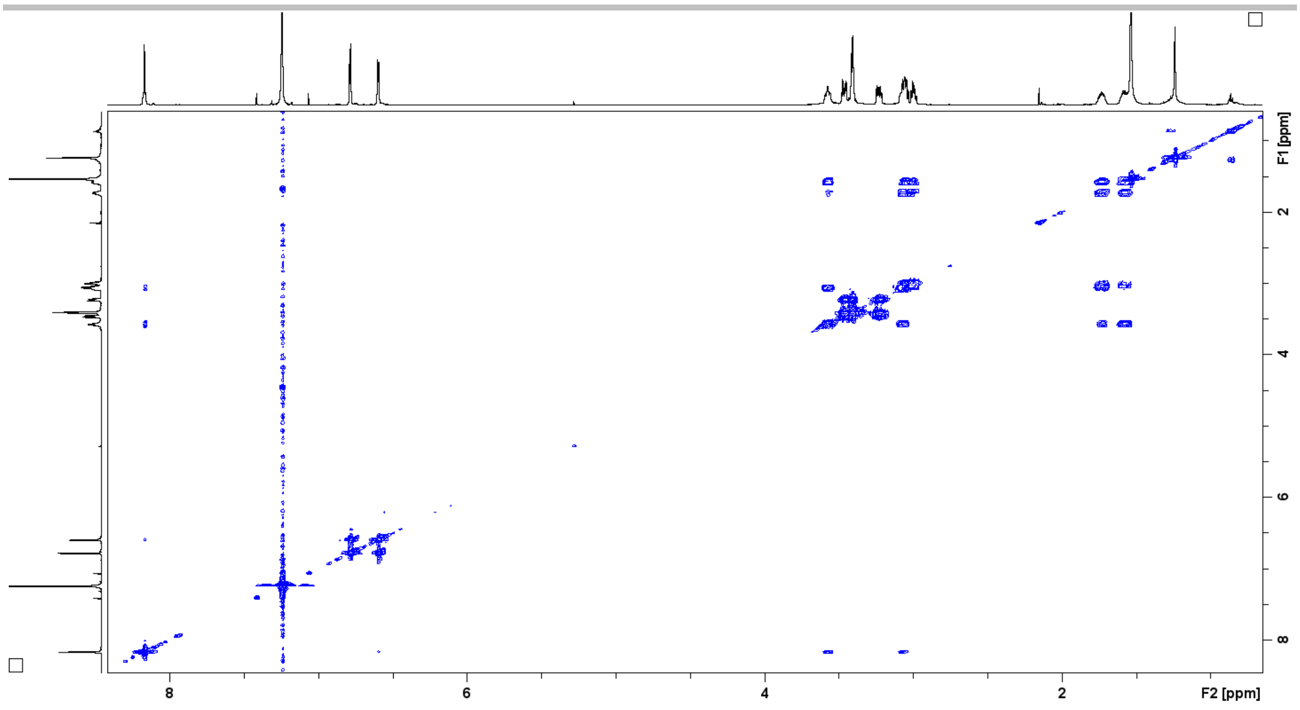

Figure S 150. The ^1H-^1H COSY spectrum of **22b-Cd** ([D]chloroform, 300 K, 600 MHz).

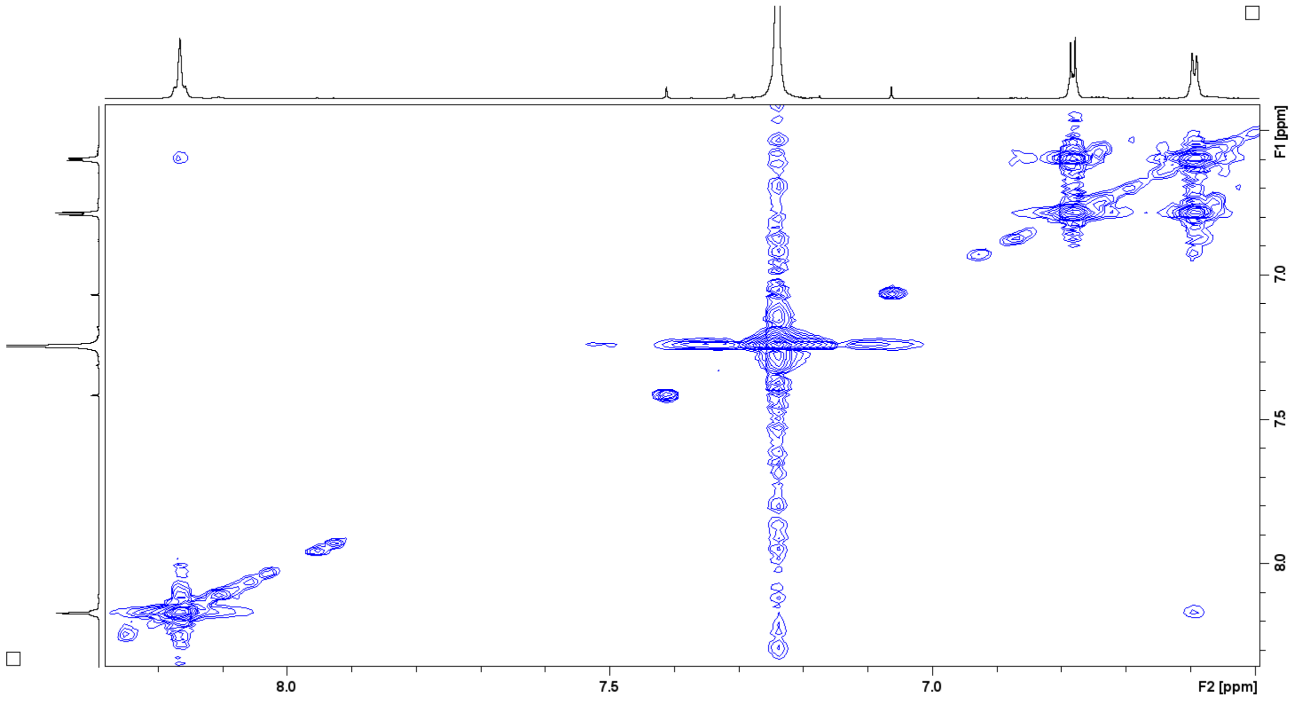

Figure S 151. The aromatic region of the ^1H-^1H COSY spectrum of **22b-Cd** ([D]chloroform, 300 K, 600 MHz).

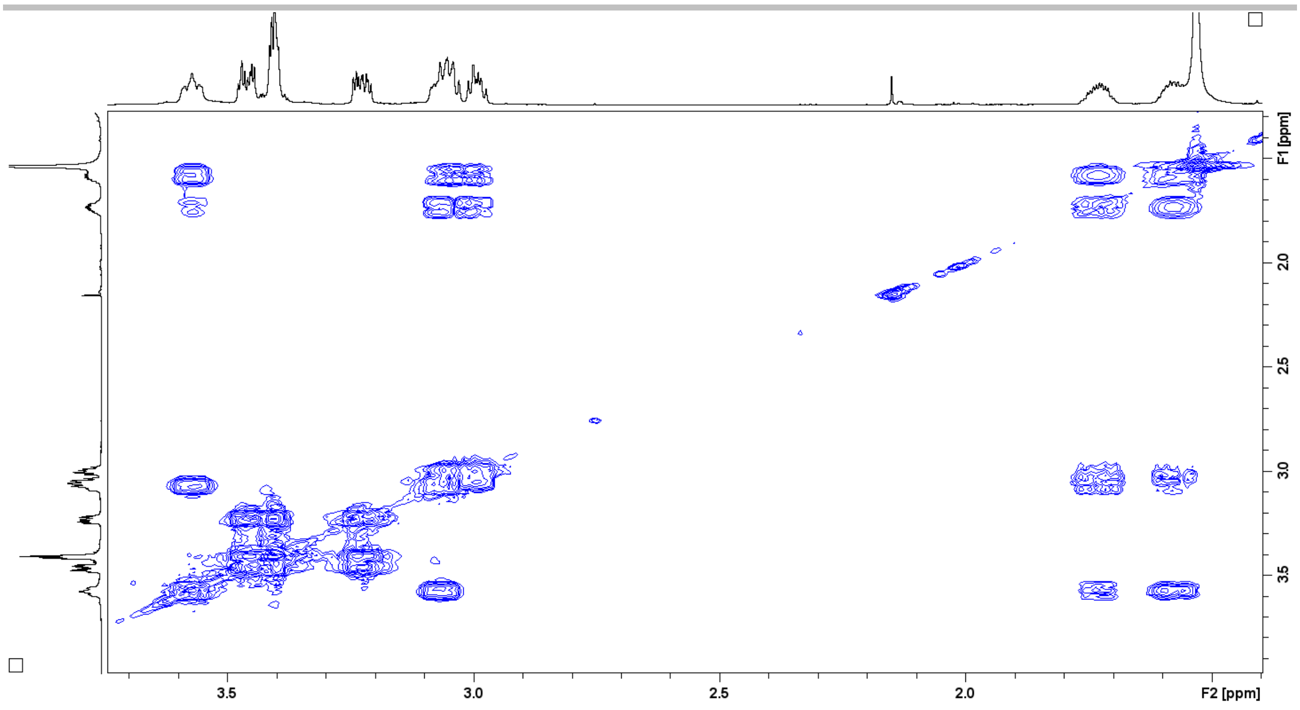

Figure S 152. The oligo(ethylene glycol) chain region of the ^{1}H-^{1}H COSY spectrum of **22b-Cd** ([D]chloroform, 300 K, 600 MHz).

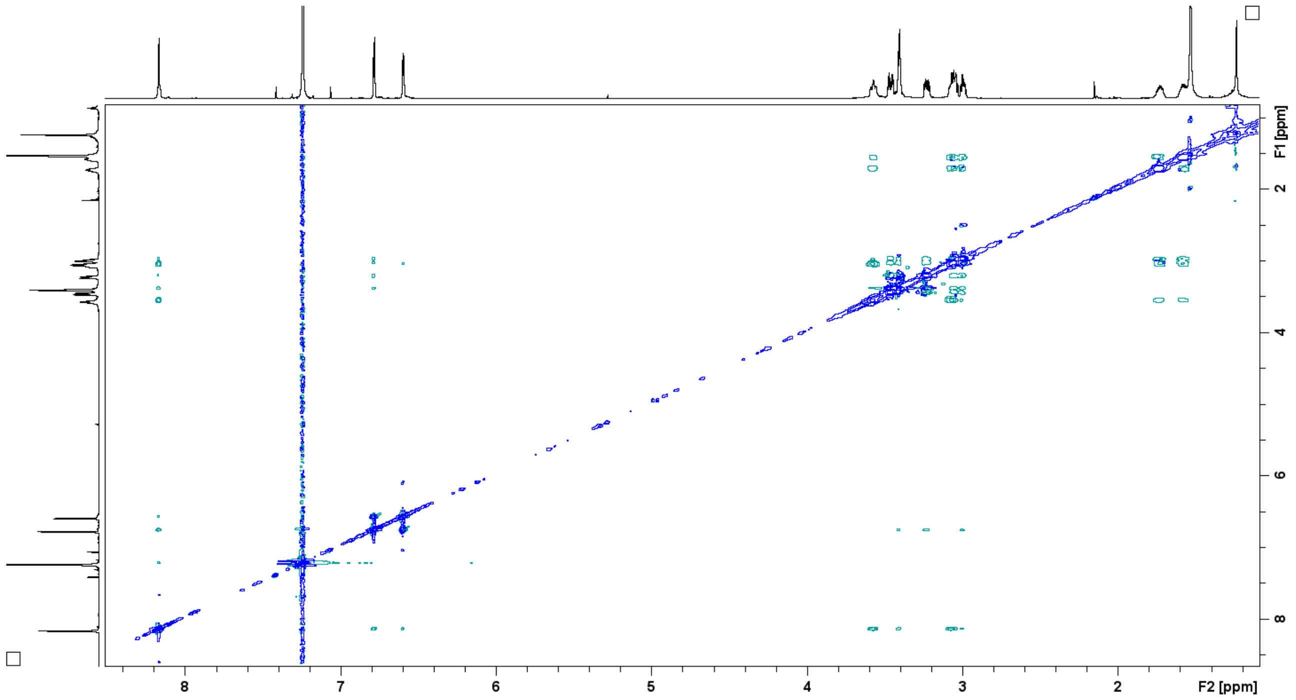

Figure S 153. The ^{1}H-^{1}H ROESY spectrum of **22b-Cd** ([D]chloroform, 300 K, 600 MHz).

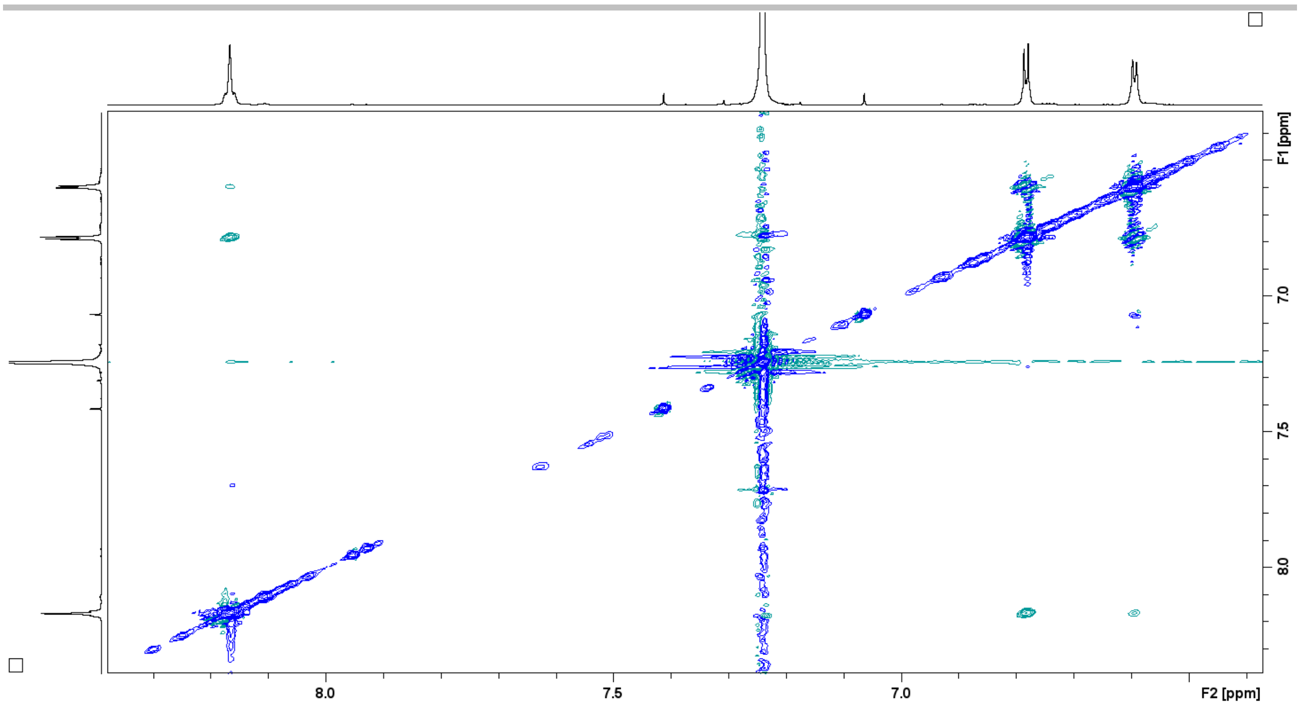

Figure S 154. The aromatic region of the ROESY spectrum of **22b-Cd** ([D]chloroform, 300 K, 600 MHz).

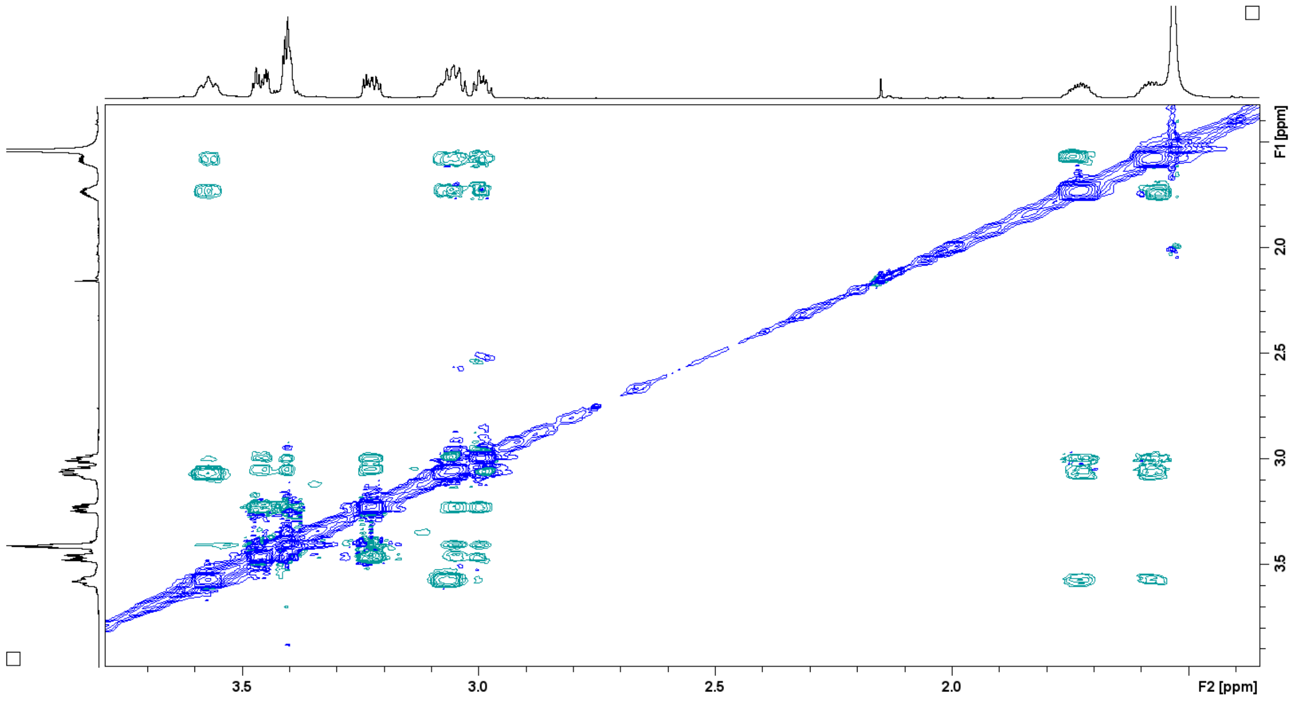

Figure S 155. The oligo(ethylene glycol) chain region of the ROESY spectrum of **22b-Cd** ([D]chloroform, 300 K, 600 MHz).

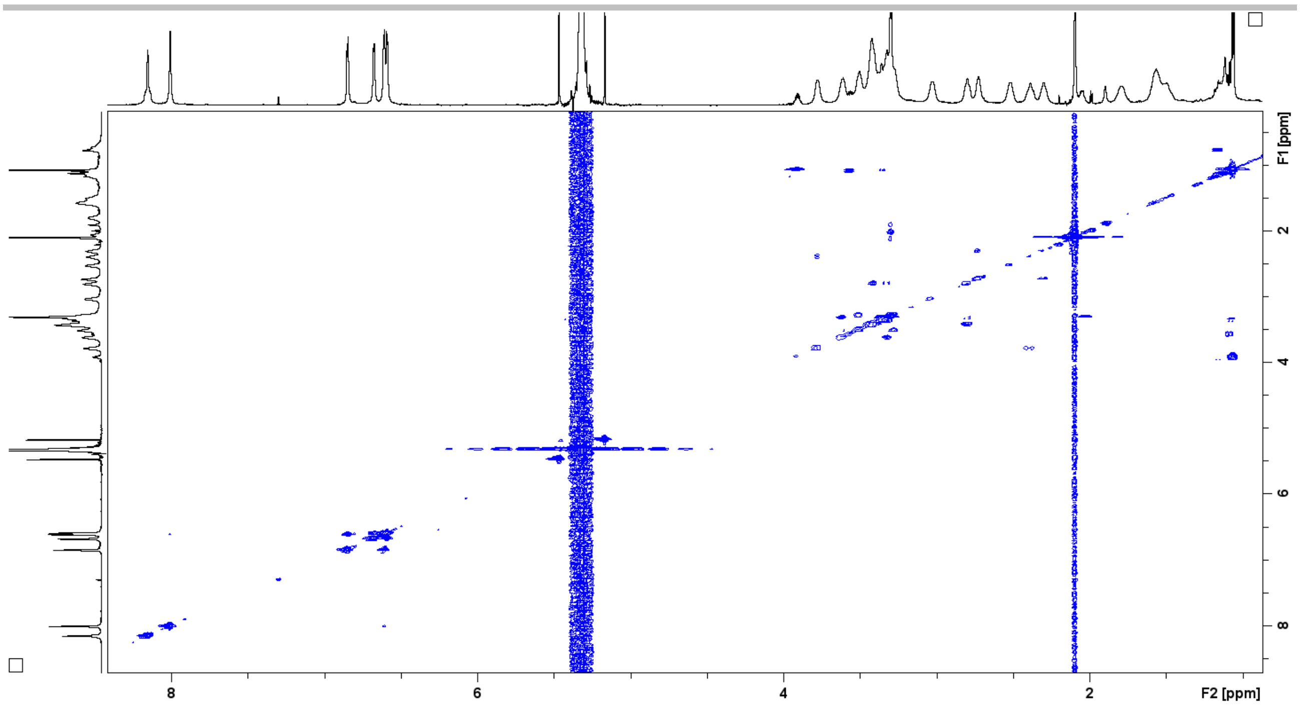

Figure S 156. The ^{1}H-^{1}H COSY spectrum of **22b-Cd** ([D$_2$]dichloromethane, 180 K, 600 MHz).

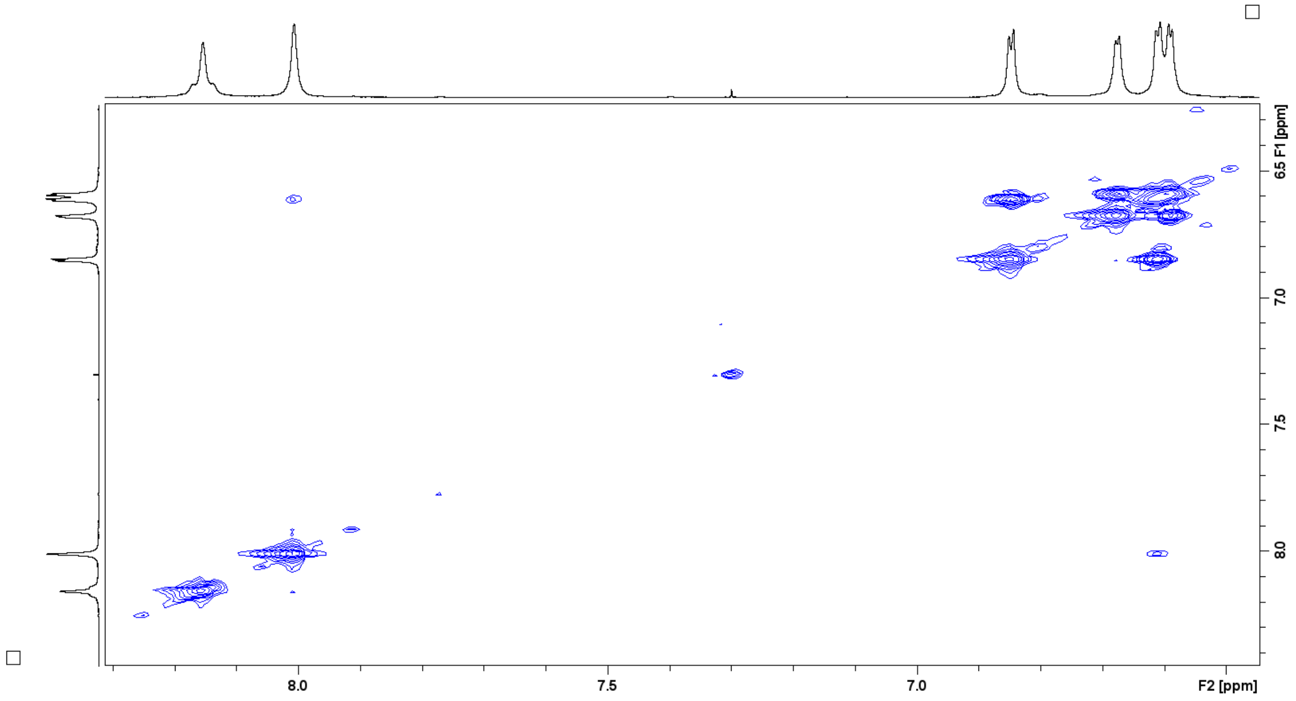

Figure S 157. The imine and aromatic region of the ^{1}H-^{1}H COSY spectrum of **22b-Cd** ([D$_2$]dichloromethane, 180 K, 600 MHz).

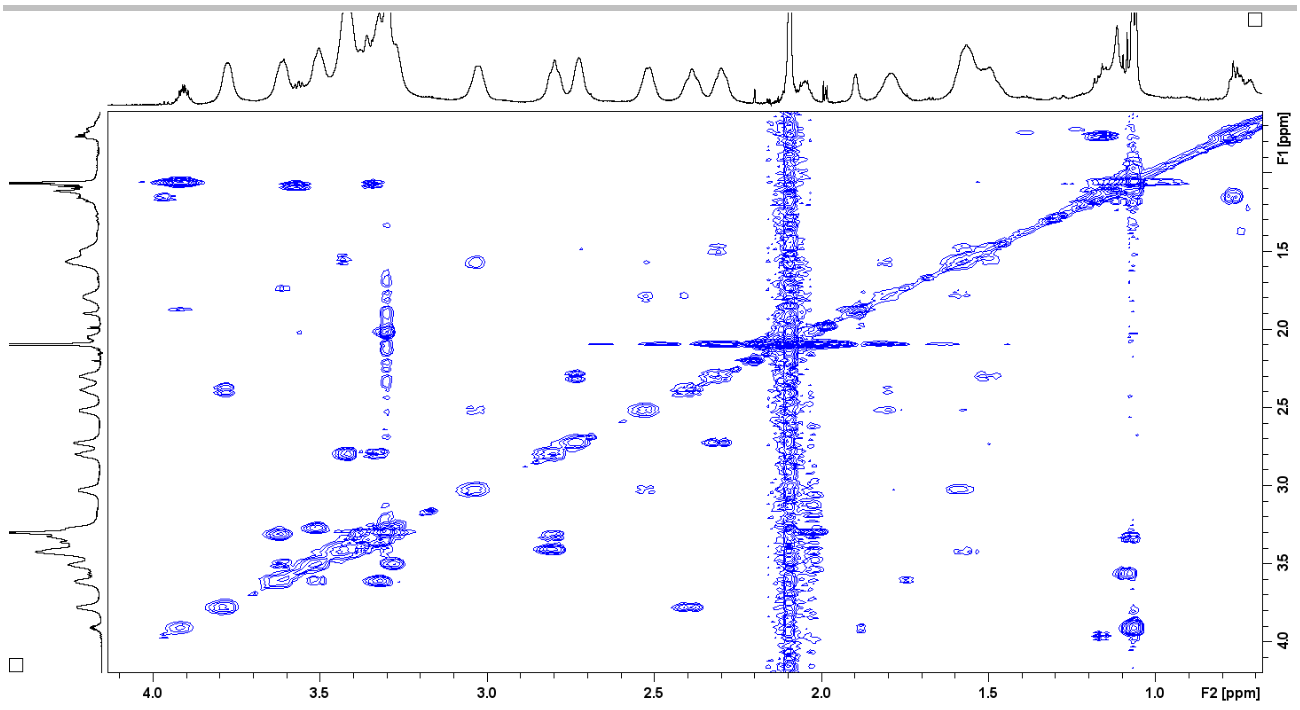

Figure S 158. The oligo(ethylene glycol) chain region of the ^{1}H-^{1}H COSY spectrum of **22b-Cd** ([D_2]dichloromethane, 180 K, 600 MHz).

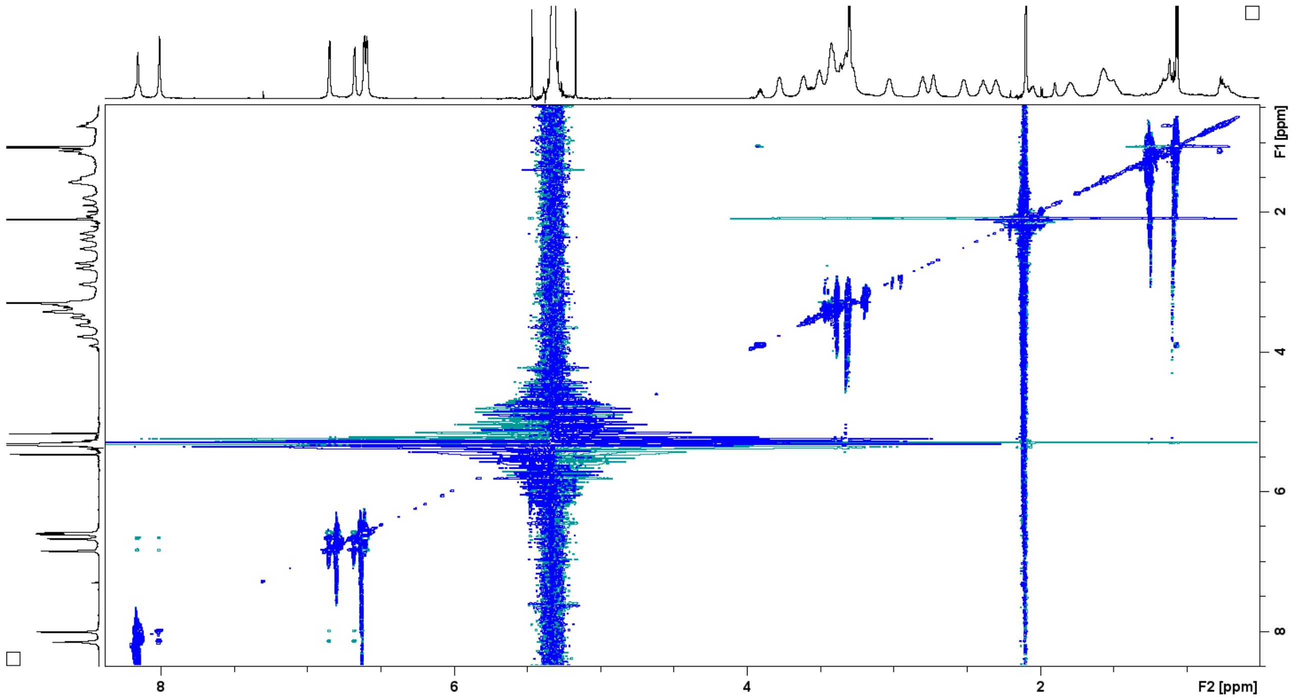

Figure S 159. The ^{1}H-^{1}H ROESY spectrum of **22b-Cd** ([D_2]dichloromethane, 180 K, 600 MHz).

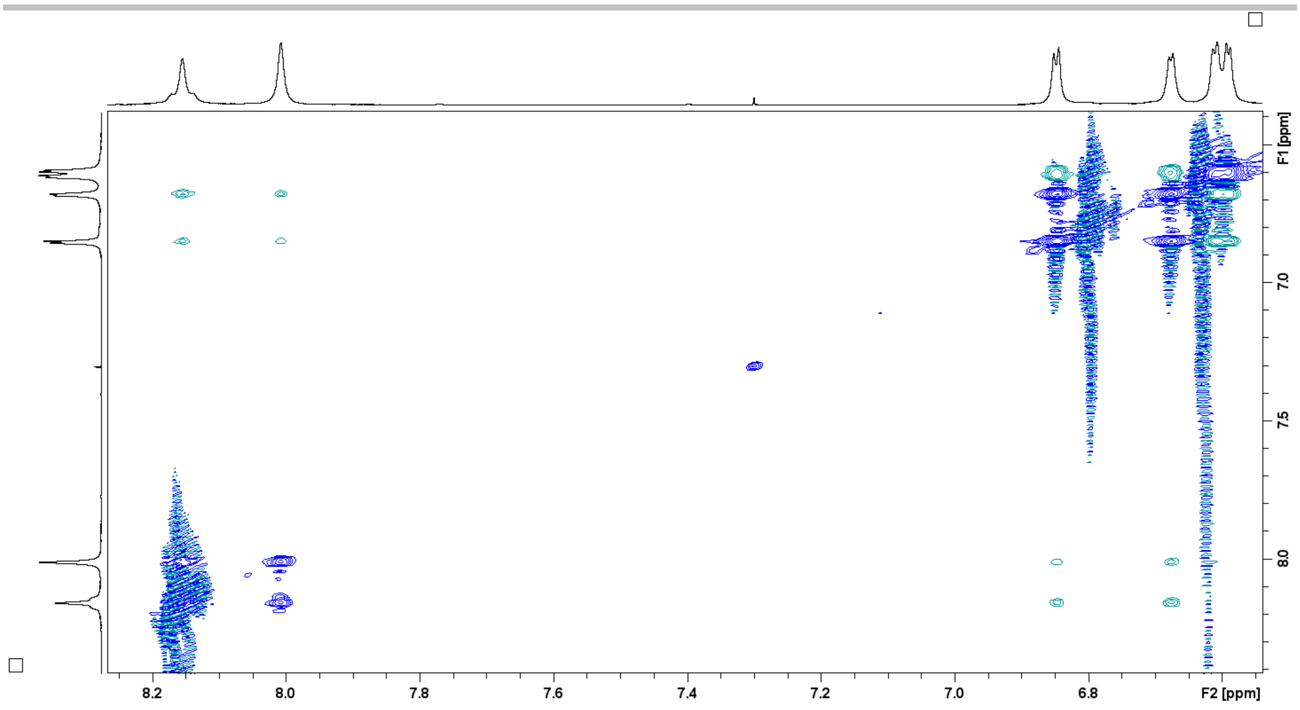

Figure S 160. The imine and aromatic region of the ^1H-^1H ROESY spectrum of **22b-Cd** ([D_2]dichloromethane, 180 K, 600 MHz).

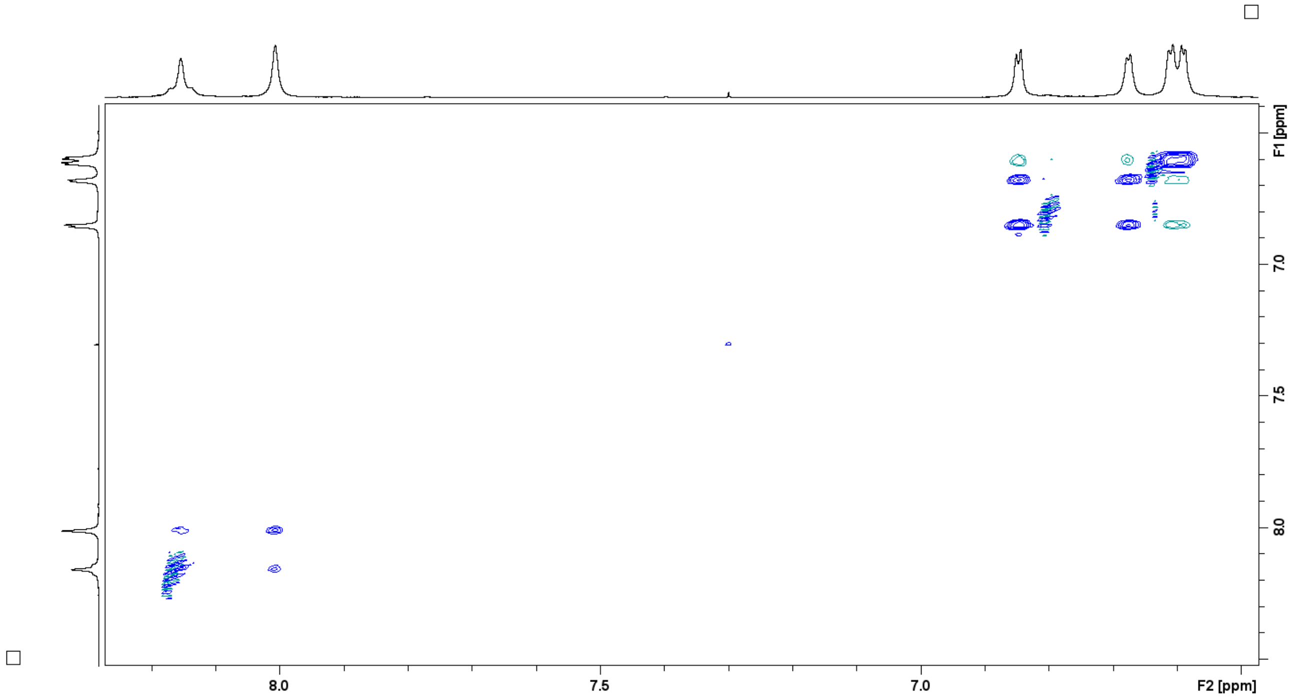

Figure S 161. The imine and aromatic region of the ^1H-^1H ROESY spectrum of lower intensity of **22b-Cd** ([D_2]dichloromethane, 180 K, 600 MHz).

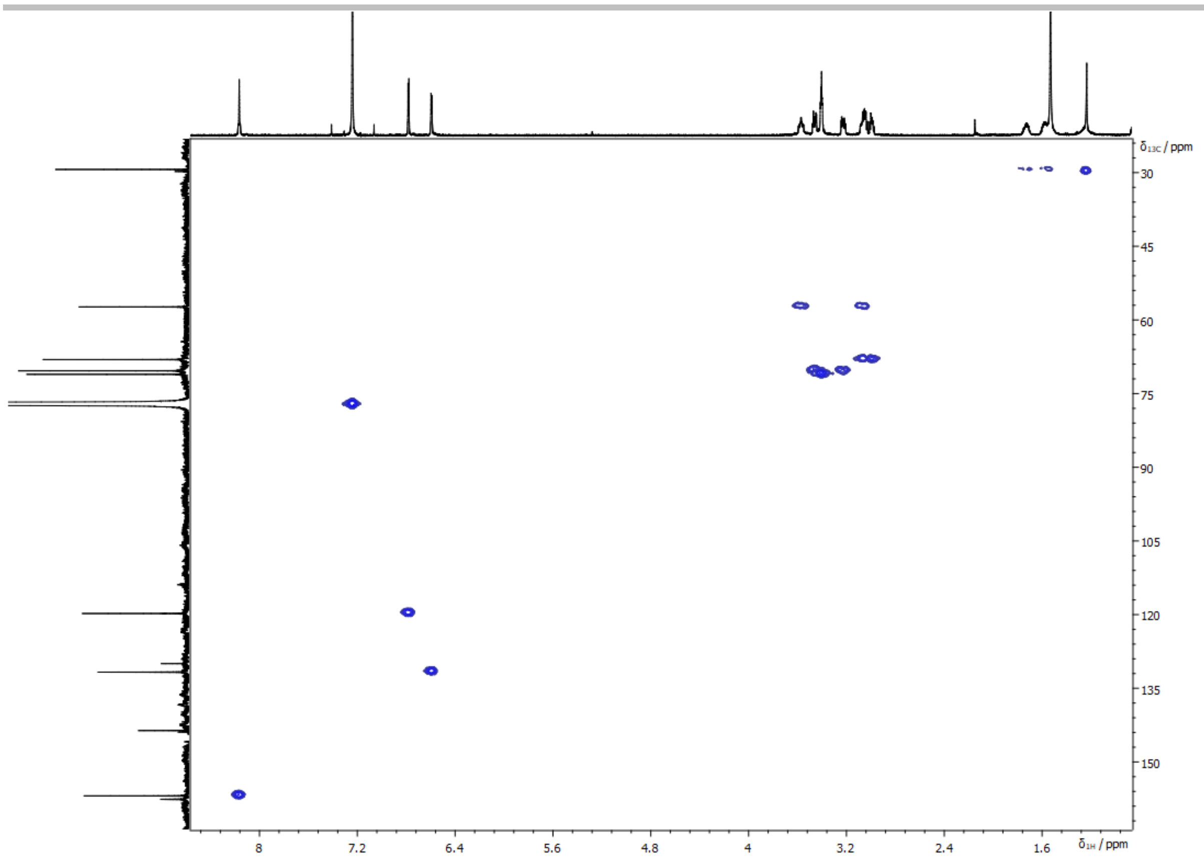

Figure S 162. The ^{1}H-^{13}C HMQC spectrum of **22b-Cd** ([D]chloroform, 300 K, 600 MHz).

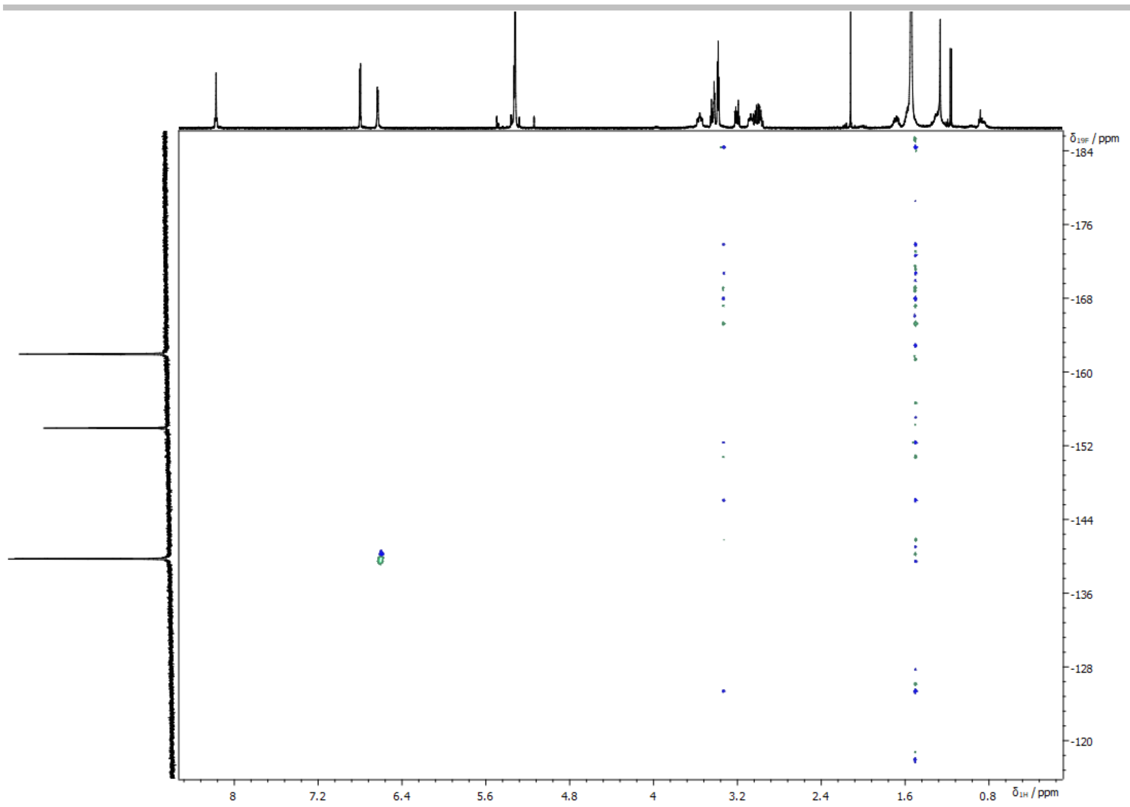

Figure S 163. The ^{1}H-^{19}F HOESY spectrum of **22b-Cd** ([D_2]dichloromethane, 300 K, 600 MHz).

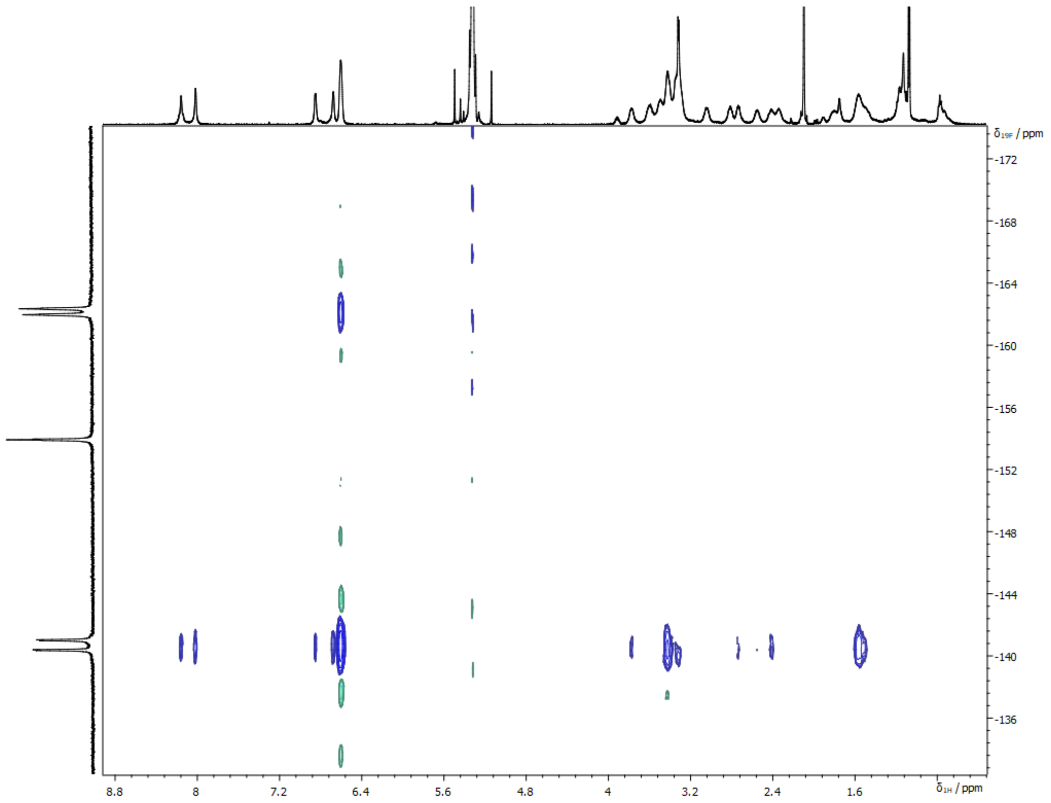

Figure S 164. The ^{1}H-^{19}F HOESY spectrum of **22b-Cd** ([D_2]dichloromethane, 180 K, 600 MHz).

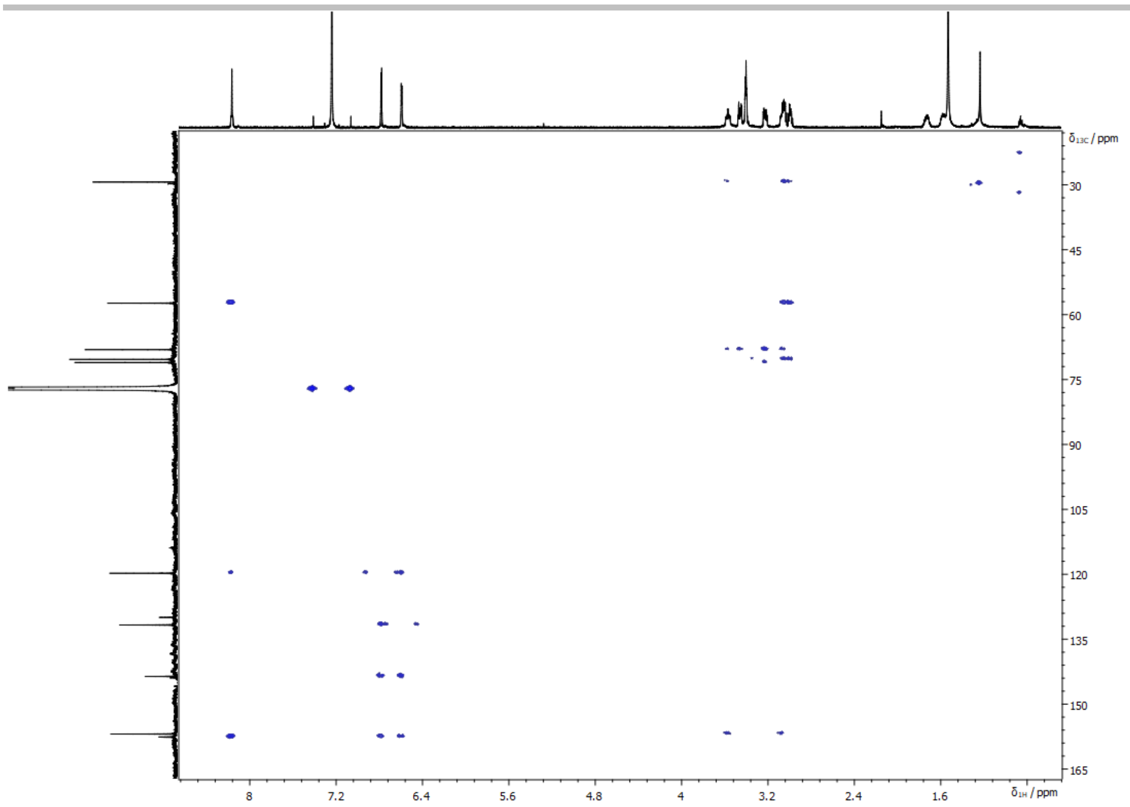

Figure S 165. The ^{1}H-^{13}C HMBC spectrum of **22b-Cd** ([D]chloroform, 300 K, 600 MHz).

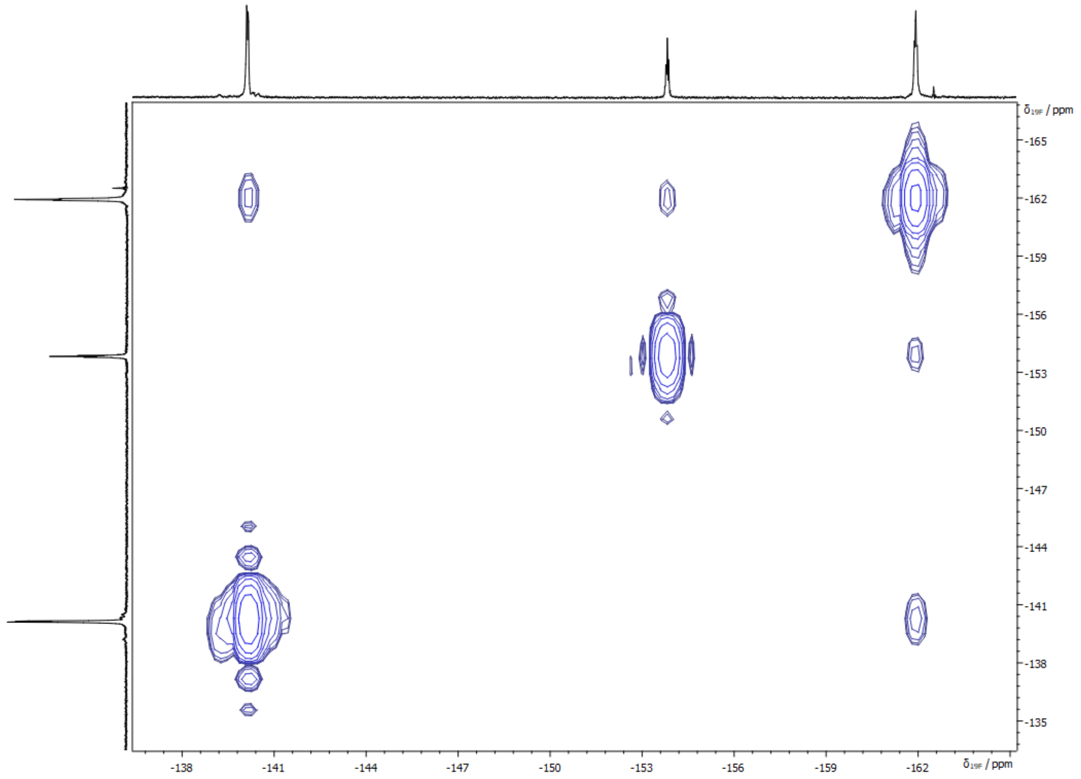

Figure S 166. The ^{19}F-^{19}F COSY spectrum of **22b-Cd** ([D_2]dichloromethane, 230 K, 500 MHz).

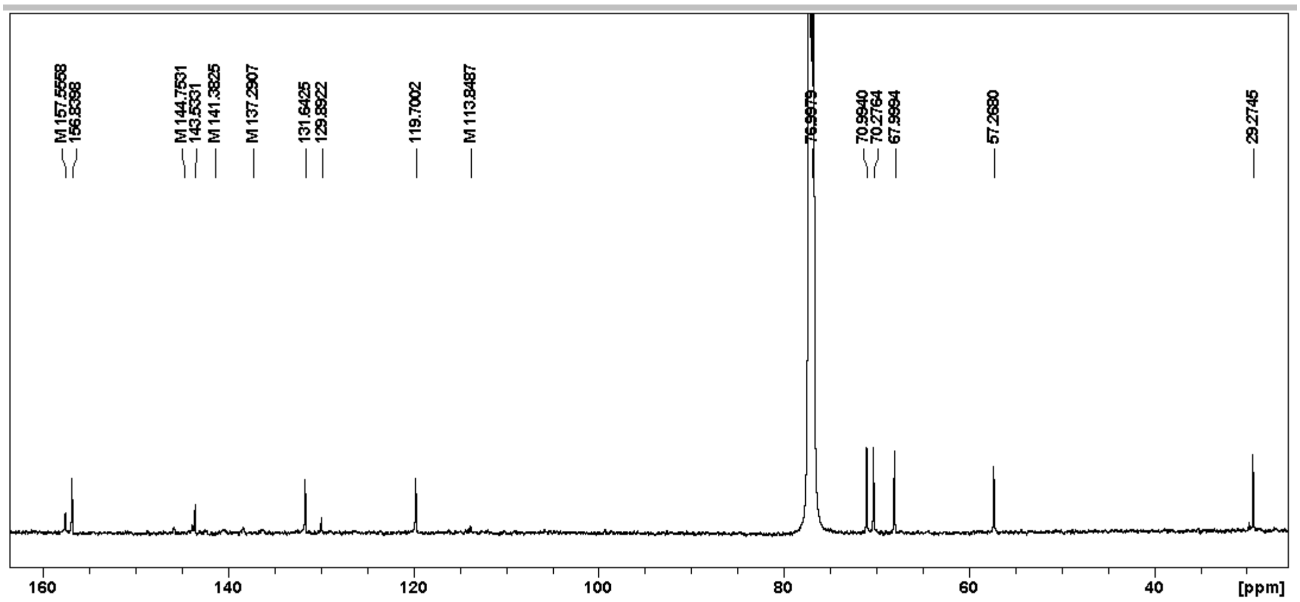

Figure S 167. The ^{13}C NMR spectrum of **22b-Cd** ([D]chloroform, 300 K, 151 MHz).

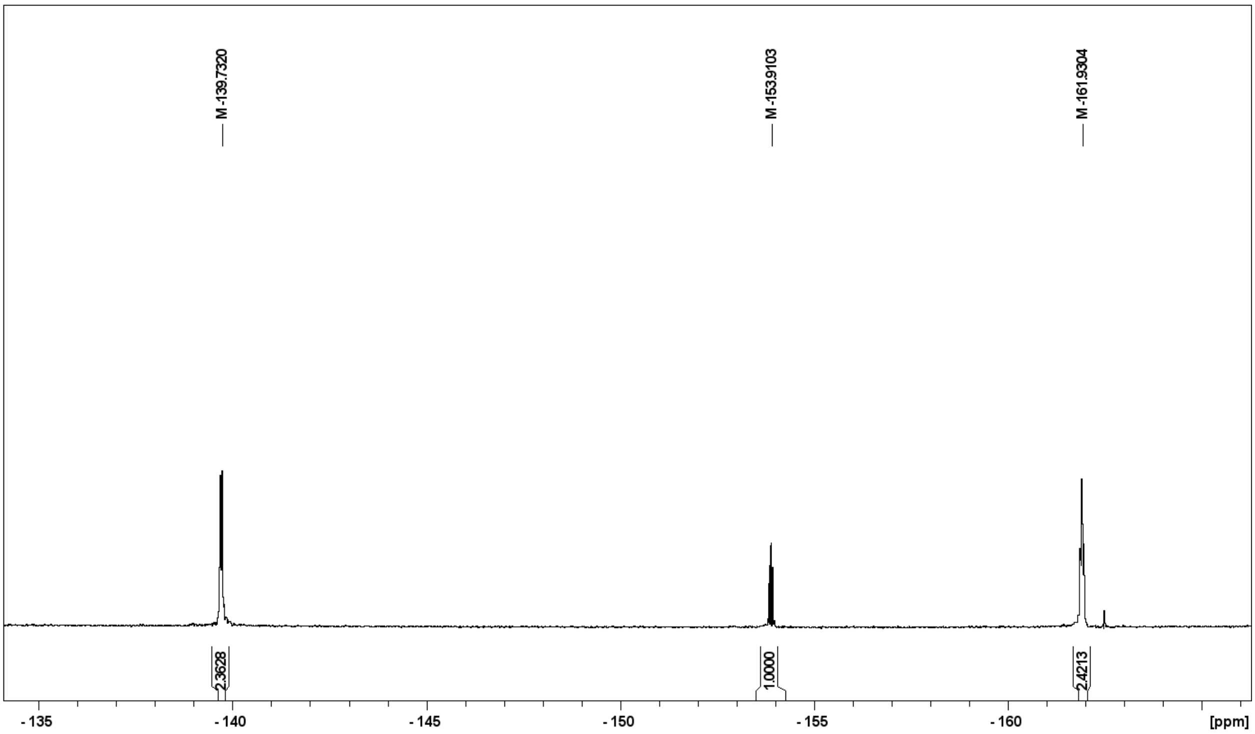

Figure S 168. The ^{19}F NMR spectrum of **22b-Cd** ([D_2]dichloromethane, 300 K, 471 MHz).

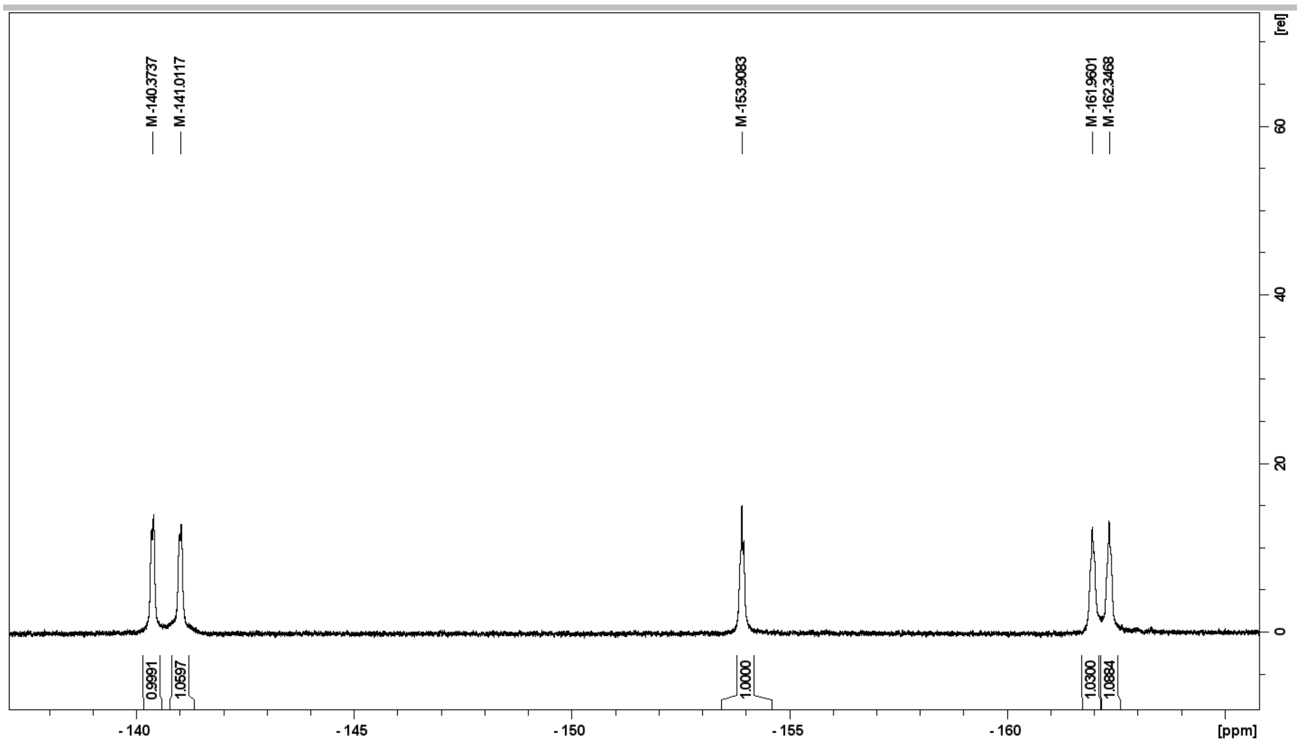

Figure S 169. The ^{19}F NMR spectrum of **22b-Cd** ([D_2]dichloromethane, 180 K, 471 MHz).

The NMR spectra of 22b-Hg

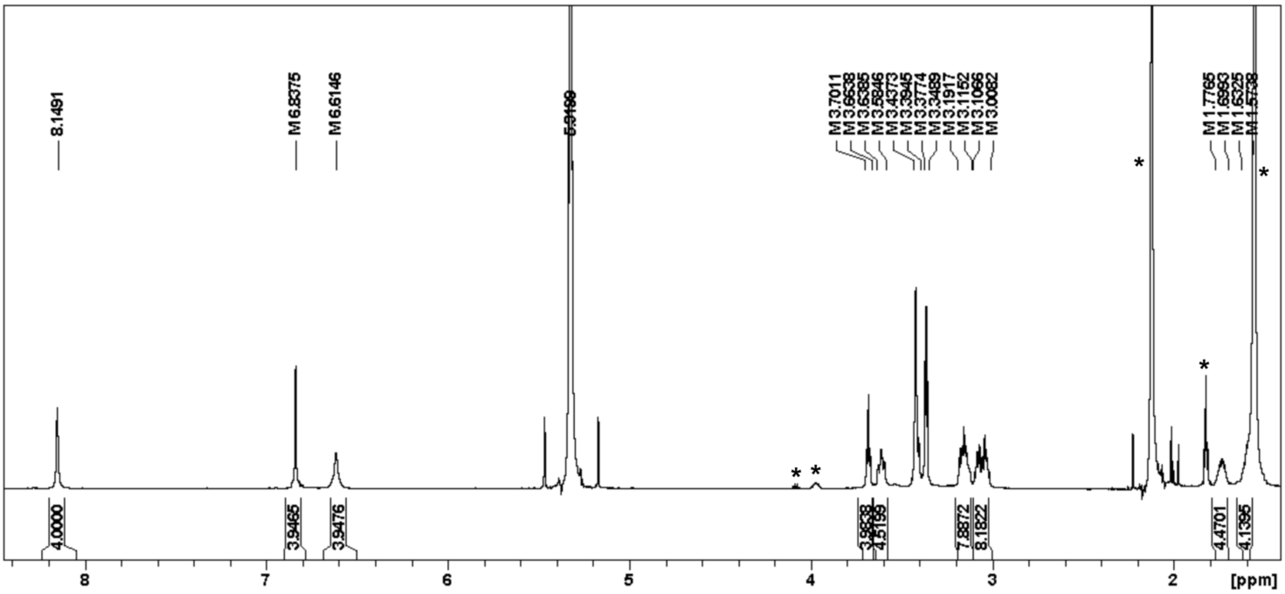

Figure S 170. The ^{1}H NMR spectrum of **22b-Hg** ([D_2]dichloromethane, 300 K, 600 MHz). Signals corresponding to impurities were marked with asterisks.

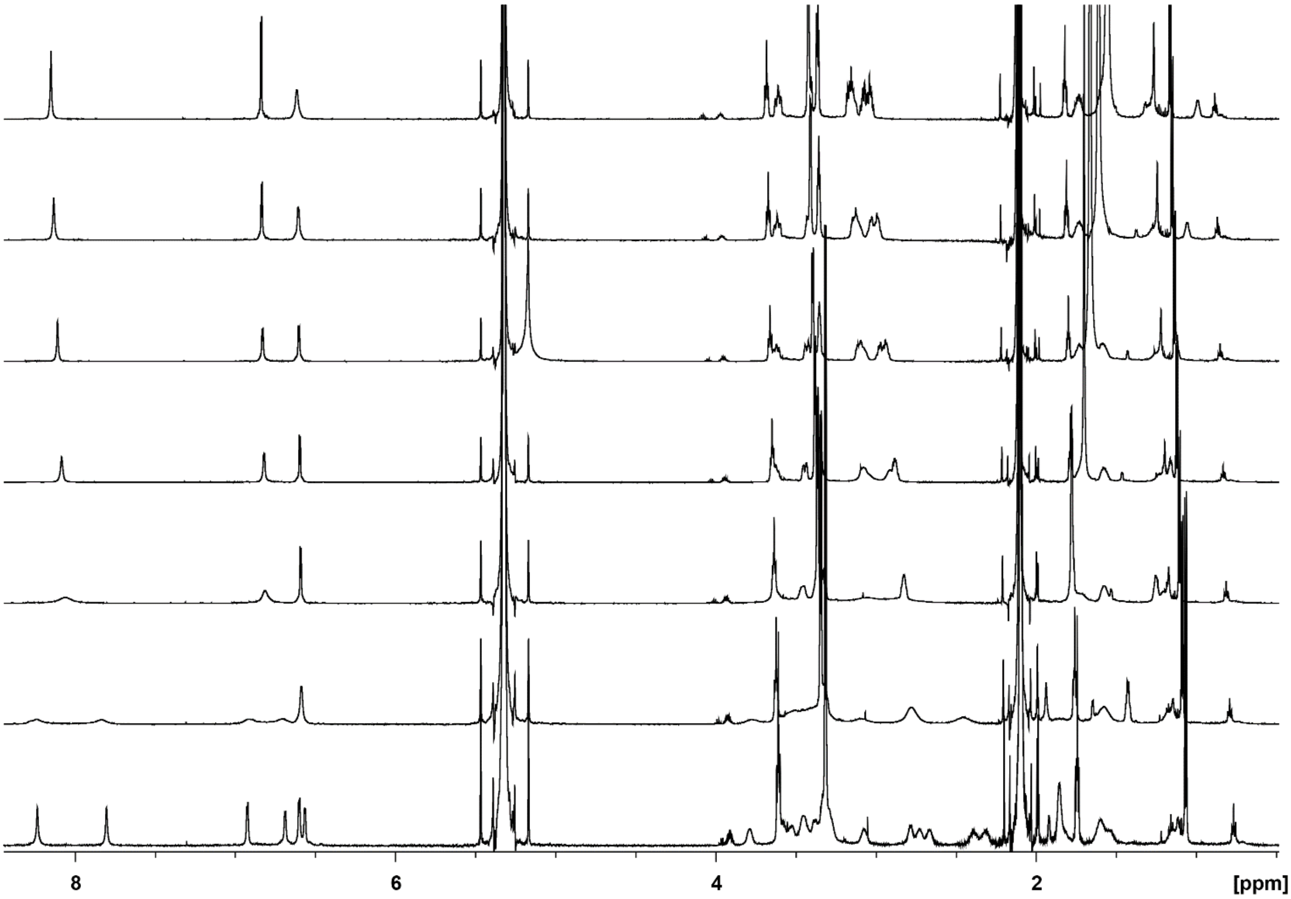

Figure S 171. ^{1}H NMR spectra of **22b-Hg** recorded in 300 K (top) – 180 K (bottom) temperature range every 20 K ([D_2]dichloromethane, 600 MHz).

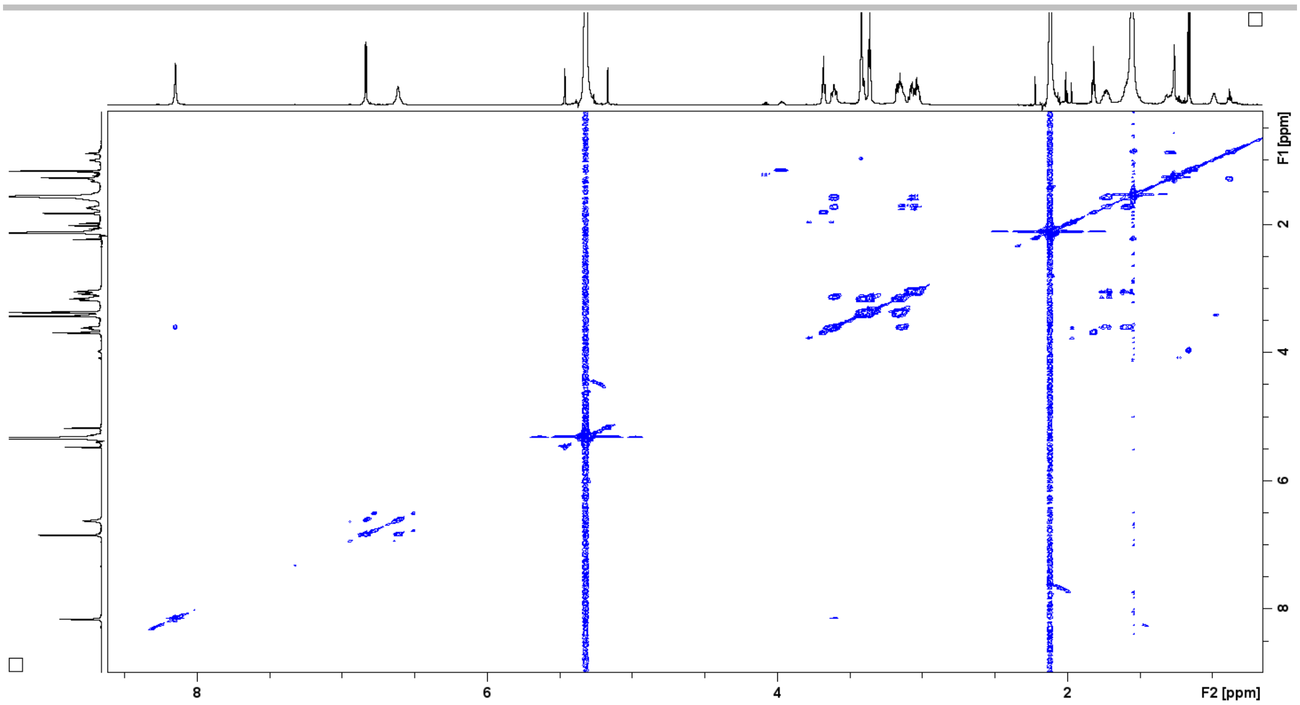

Figure S 172. The ^{1}H-^{1}H COSY spectrum of **22b-Hg** ([D_2]dichloromethane, 300 K, 600 MHz).

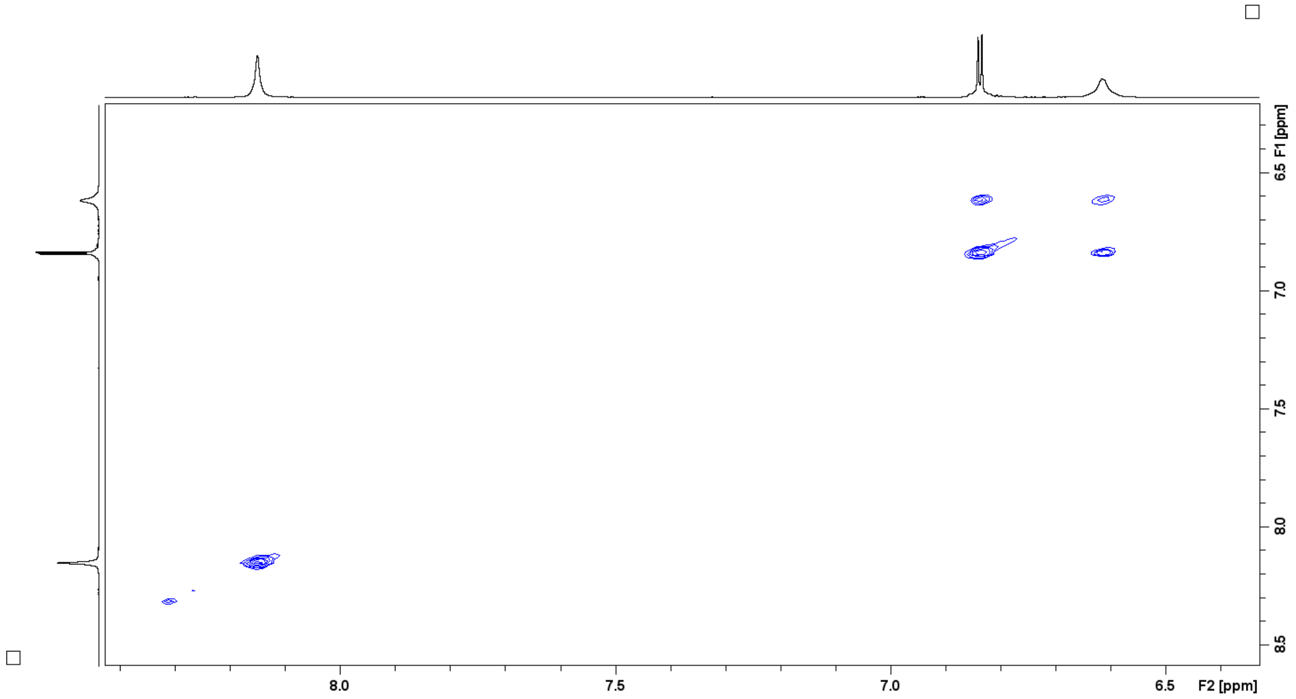

Figure S 173. The imine and aromatic region of the ^{1}H-^{1}H COSY spectrum of **22b-Hg** ([D_2]dichloromethane, 300 K, 600 MHz).

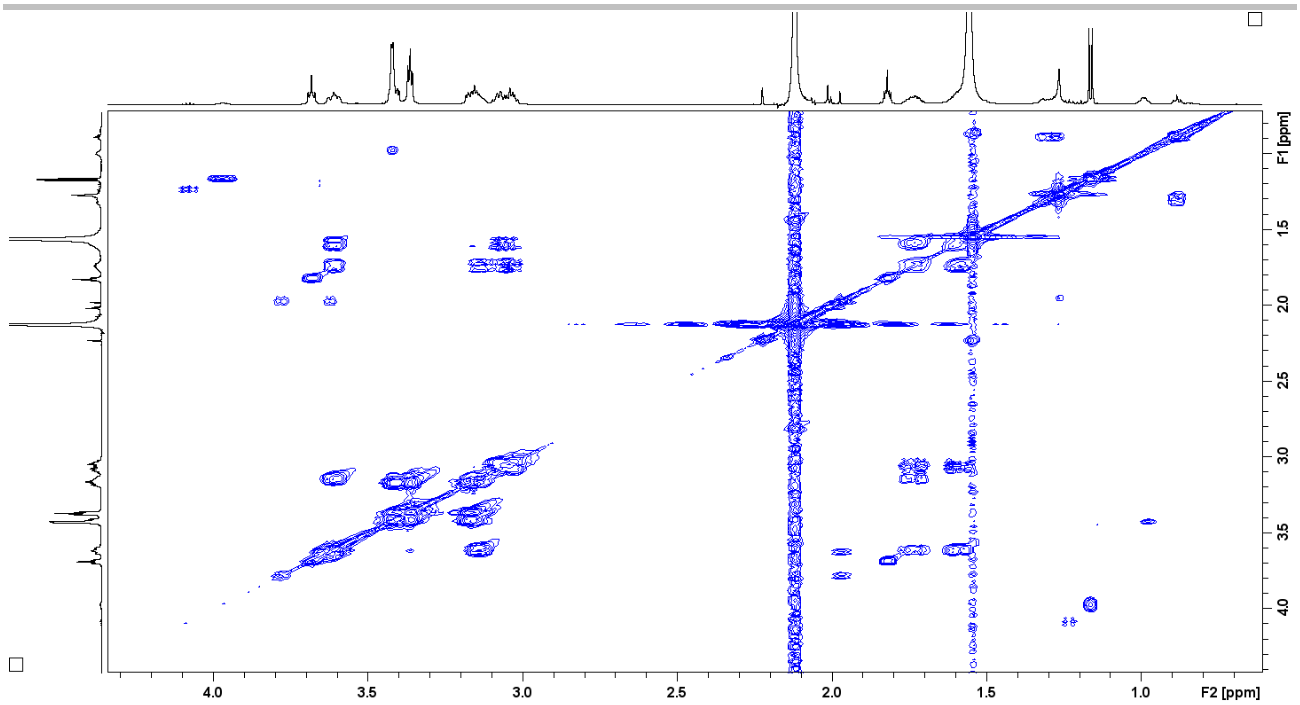

Figure S 174. The oligo(ethylene glycol) chain region of the ^{1}H-^{1}H COSY spectrum of **22b-Hg** ([D$_2$]dichloromethane, 300 K, 600 MHz).

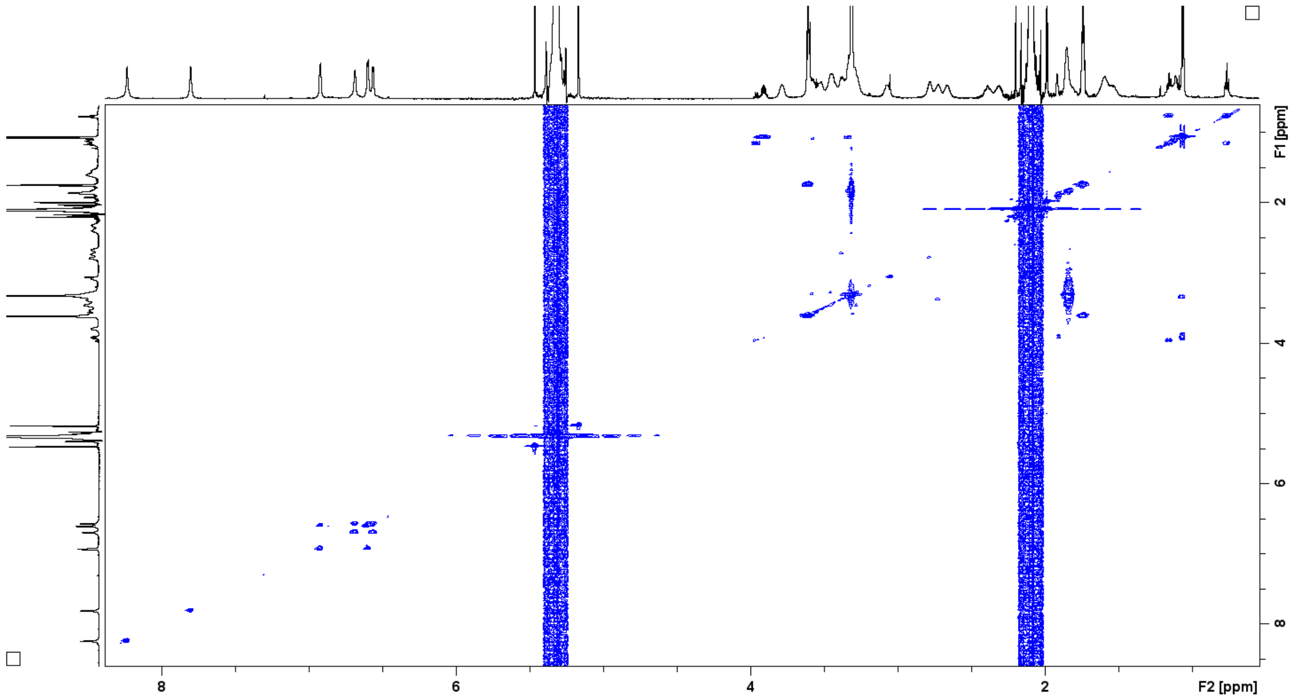

Figure S 175. The ^{1}H-^{1}H COSY spectrum of **22b-Hg** ([D$_2$]dichloromethane, 180 K, 600 MHz).

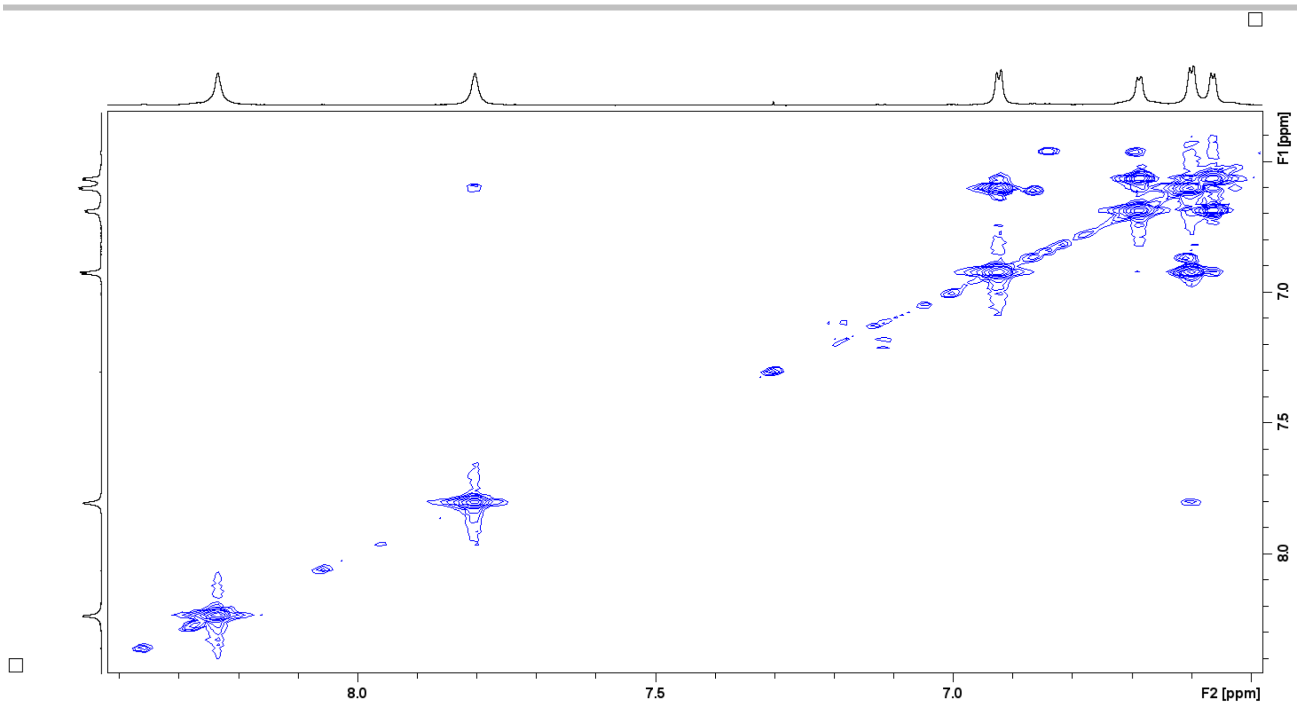

Figure S 176. The imine and aromatic region of the ^{1}H-^{1}H COSY spectrum of **22b-Hg** ([D$_2$]dichloromethane, 180 K, 600 MHz).

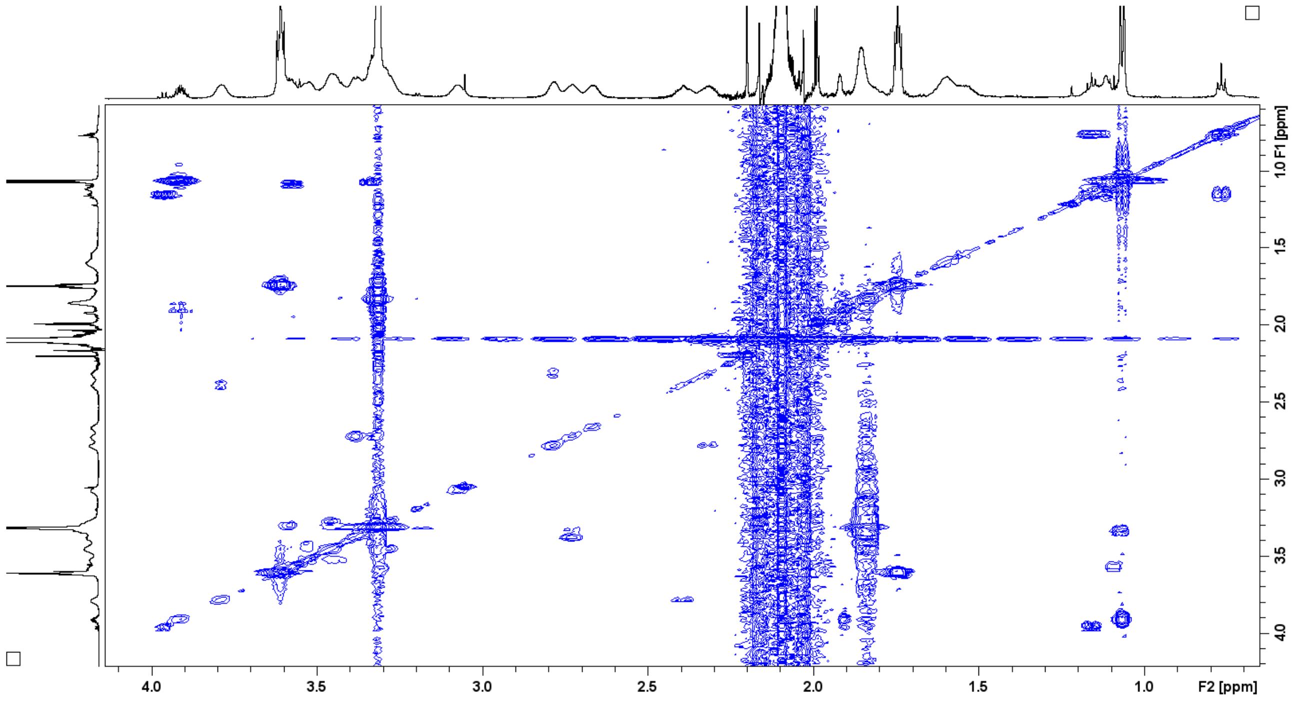

Figure S 177. The oligo(ethylene glycol) chain region of the ^{1}H-^{1}H COSY spectrum of **22b-Hg** ([D$_2$]dichloromethane, 180 K, 600 MHz).

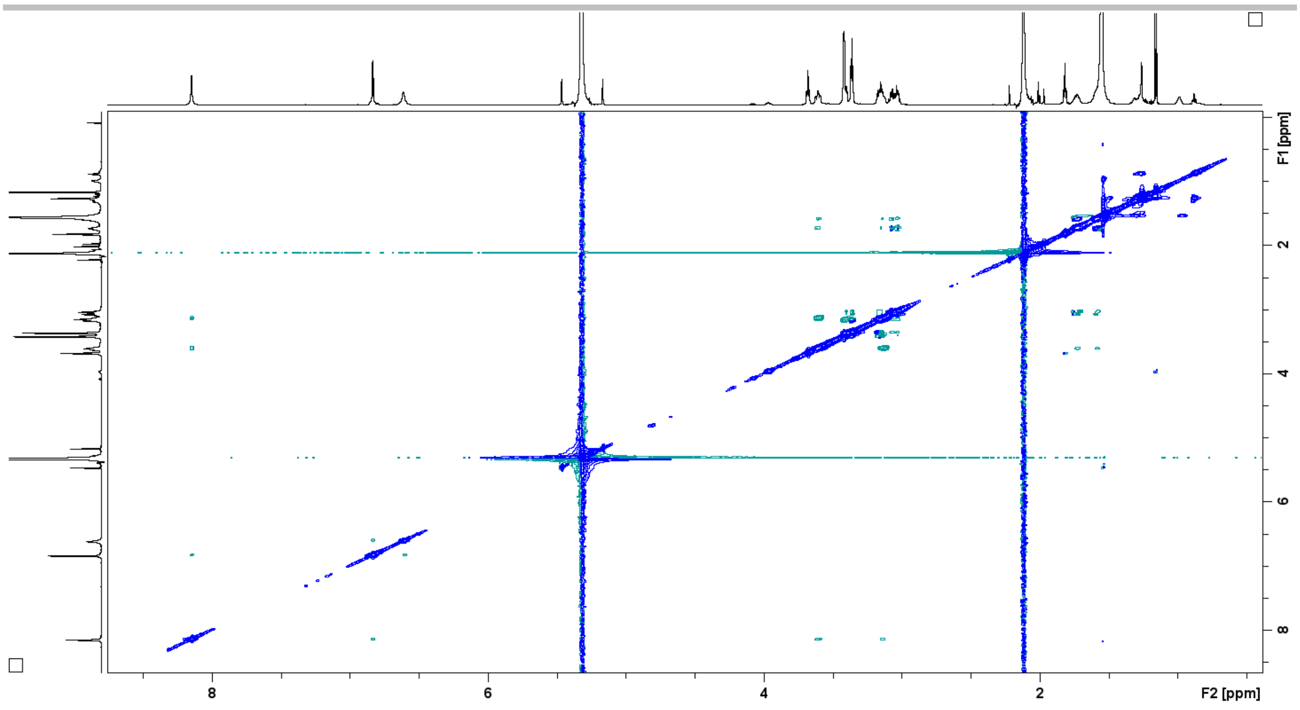

Figure S 178. The ^{1}H-^{1}H ROESY spectrum of **22b-Hg** ([D$_2$]dichloromethane, 300 K, 600 MHz).

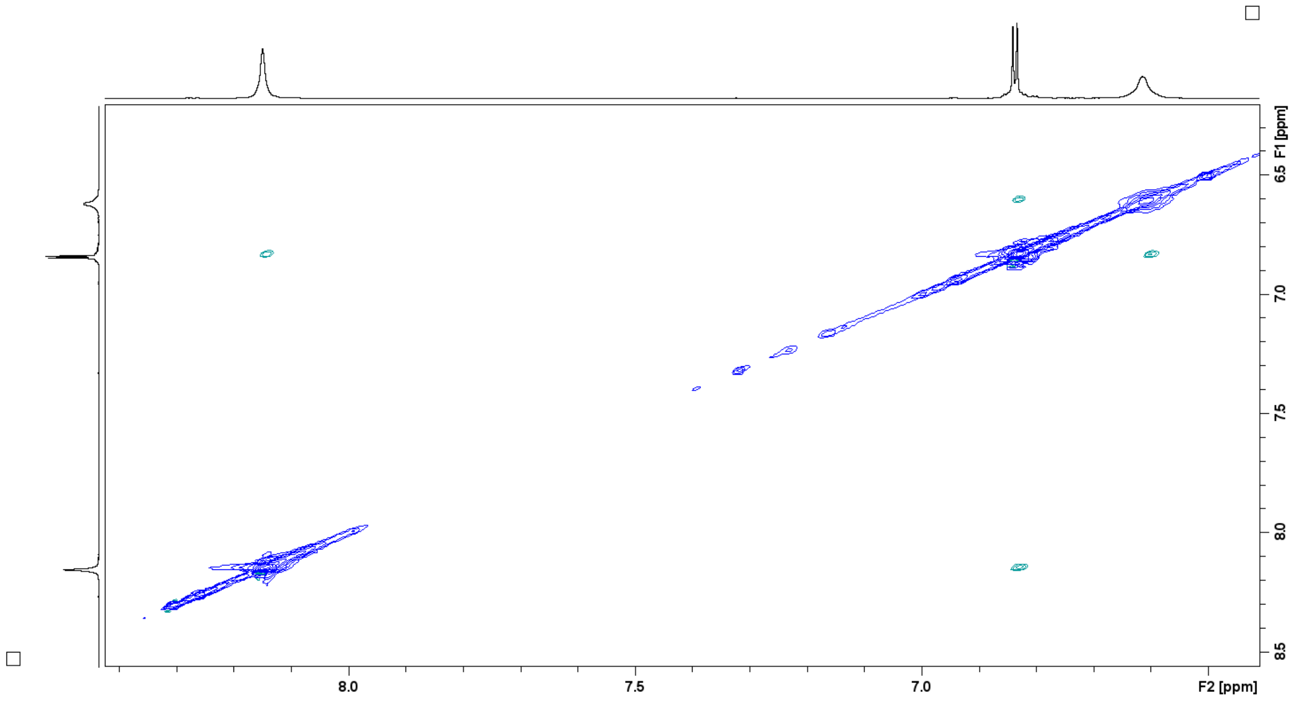

Figure S 179. The imine and aromatic region of the ROESY spectrum of **22b-Hg** ([D$_2$]dichloromethane, 300 K, 600 MHz).

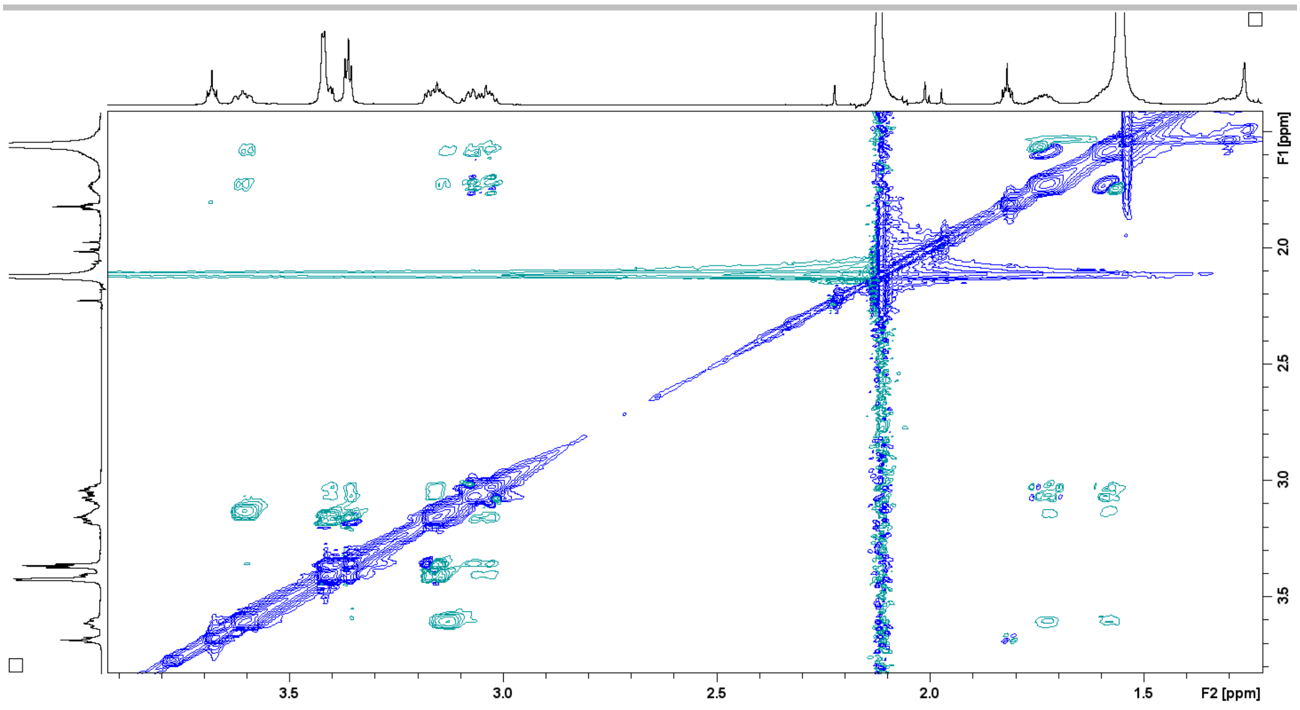

Figure S 180. The oligo(ethylene glycol) chain region of the ROESY spectrum of **22b-Hg** ([D$_2$]dichloromethane, 300 K, 600 MHz).

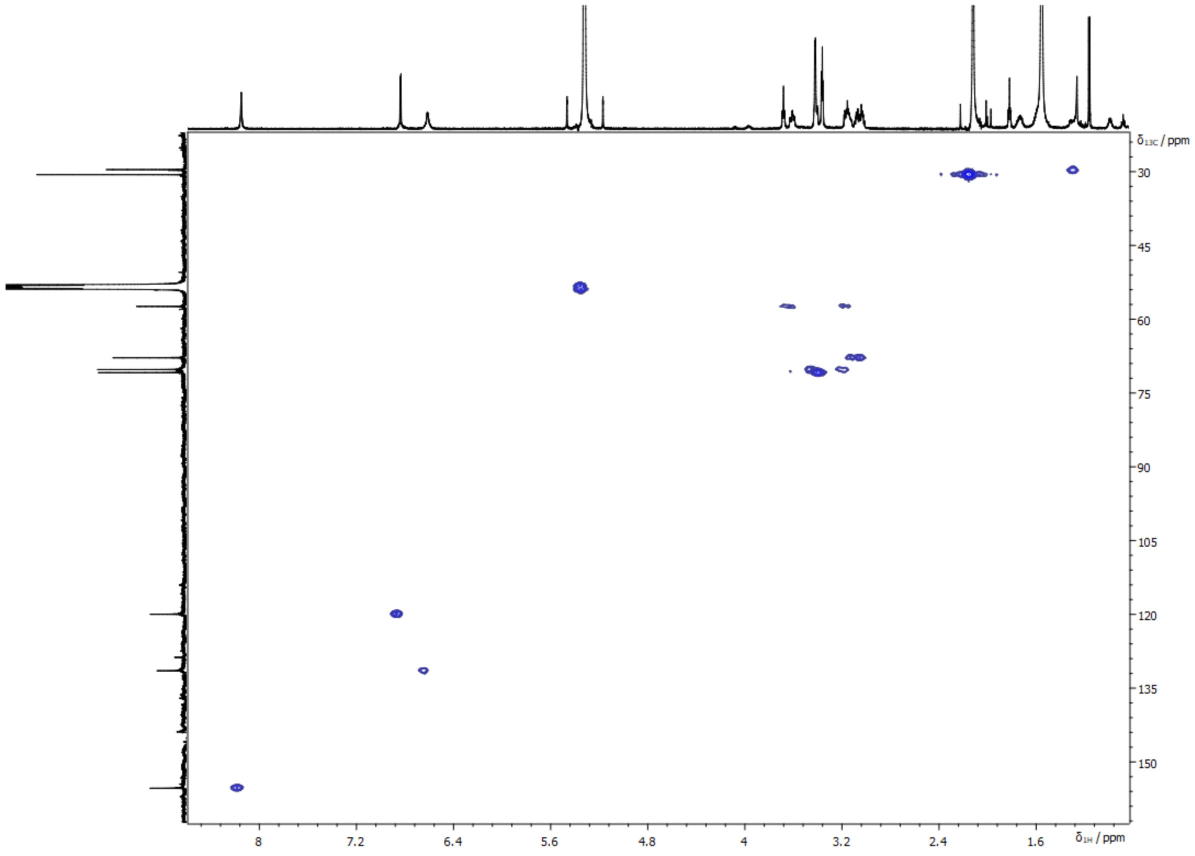

Figure S 181. The ^{1}H-^{13}C HMQC spectrum of **22b-Hg** ([D$_2$]dichloromethane, 300 K, 600 MHz).

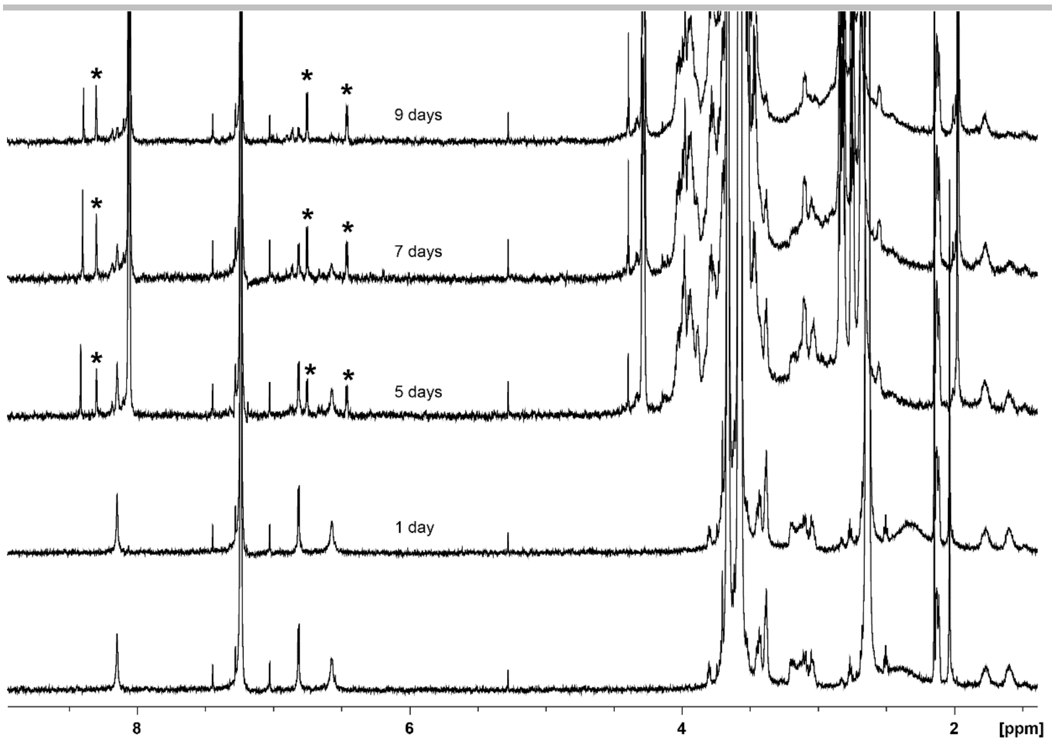

Figure S 182. The ^{1}H NMR spectra demonstrating the conversion of **22b-Hg** to **15b-H** (imine and β-pyrrole signals marked as asterisks), upon the reaction with the excess of [2.2.2]cryptand; bottom spectrum: directly after cryptand addition, top: after 9 days ([D]chloroform, 300 K, 600 MHz).

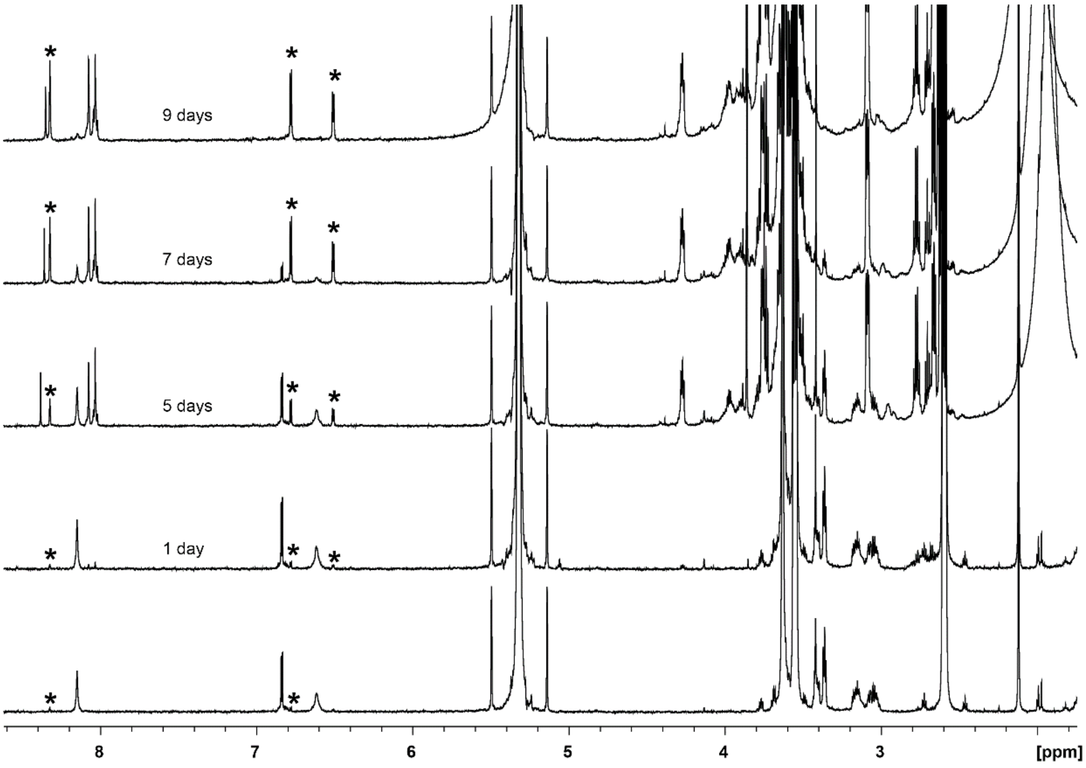

Figure S 183. The ^{1}H NMR spectra demonstrating the conversion of **22b-Hg** to **15b-H** (imine and β-pyrrole signals marked as asterisks), upon the reaction with the excess of [2.2.2]cryptand; bottom spectrum: directly after cryptand addition, top: after 9 days ([D_2]dichloromethane, 300 K, 600 MHz).

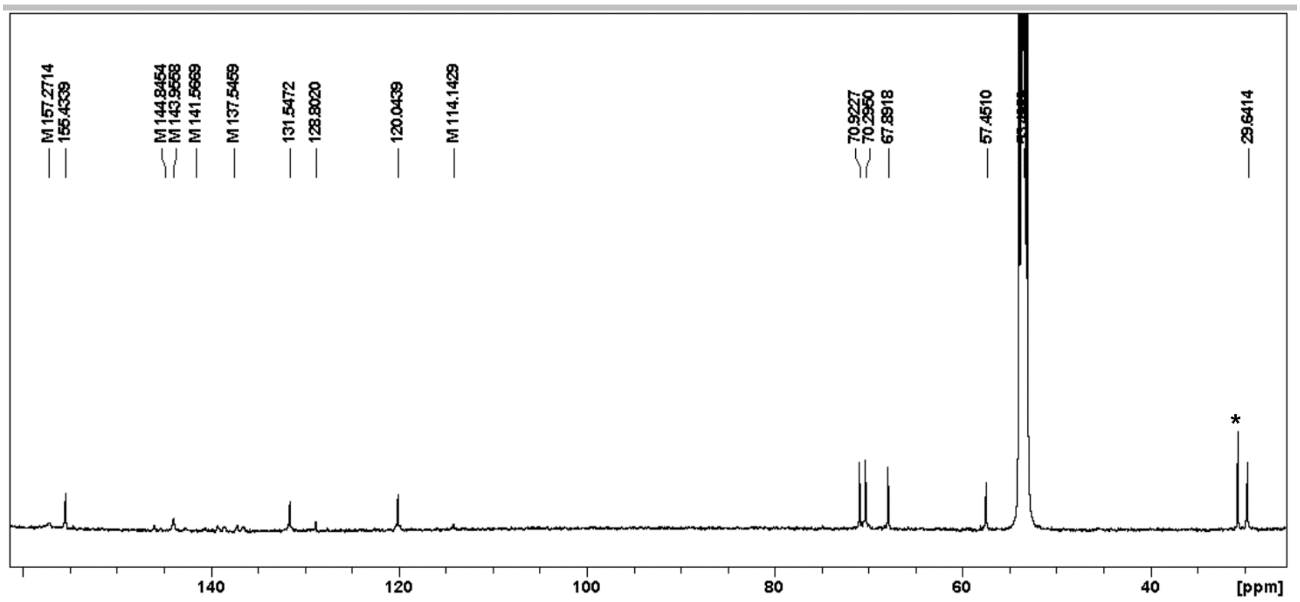

Figure S 184. The ^{13}C NMR spectrum of **22b-Hg** ([D_2]dichloromethane, 300 K, 600 MHz). Signals corresponding to impurities were marked with asterisks.

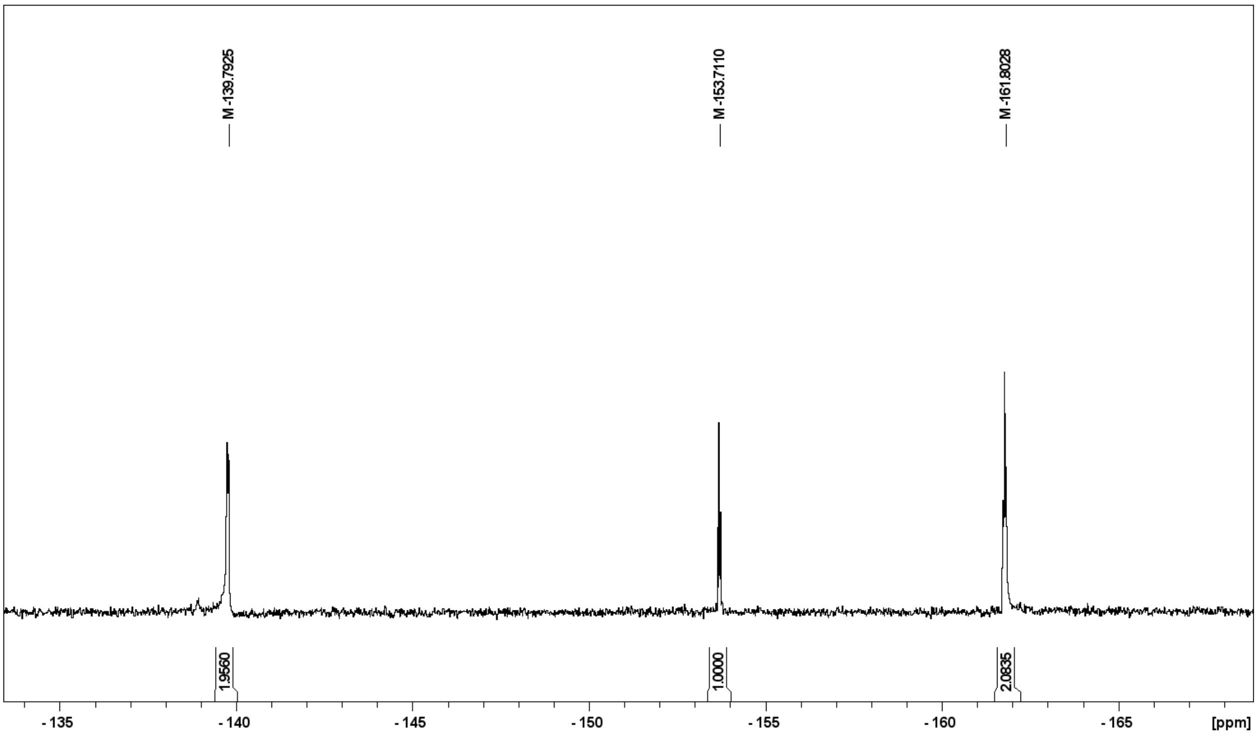

Figure S 185. The ^{19}F NMR spectrum of **22b-Hg** ([D_2]dichloromethane, 300 K, 471 MHz).

UV-vis spectra

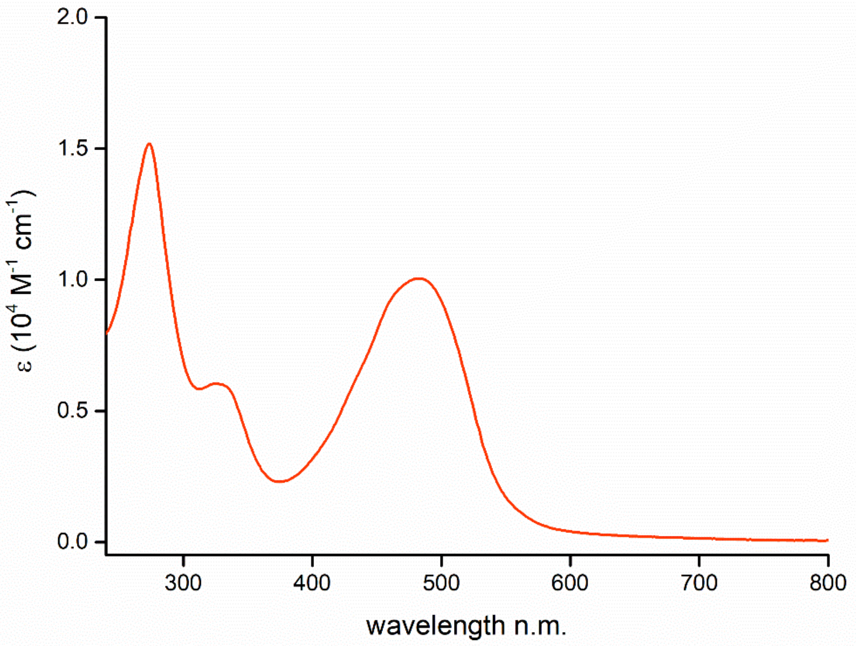

Figure S 186. The UV–vis absorption spectrum of **14b-H** (dichloromethane, 298 K).

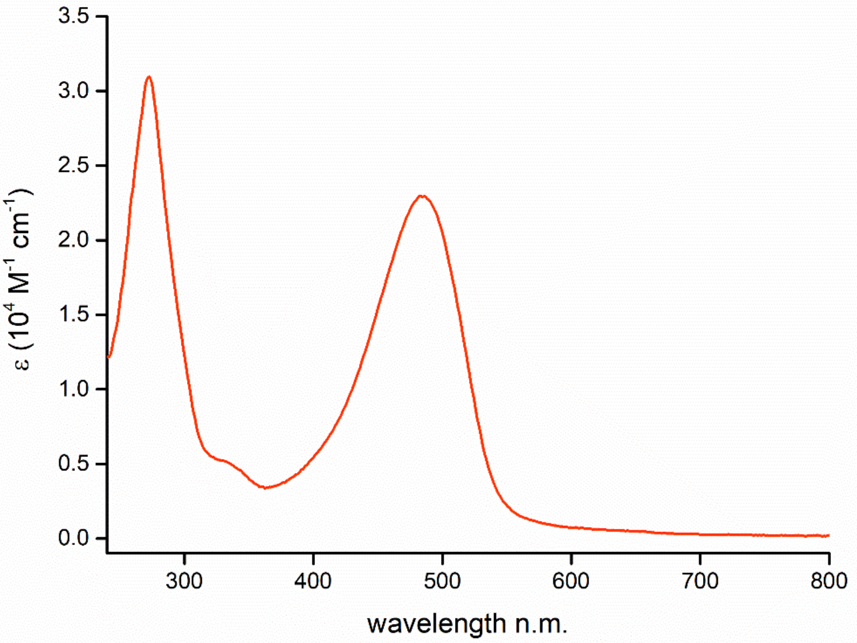

Figure S 187. The UV–vis absorption spectra of **15b-H** (dichloromethane, 298 K).

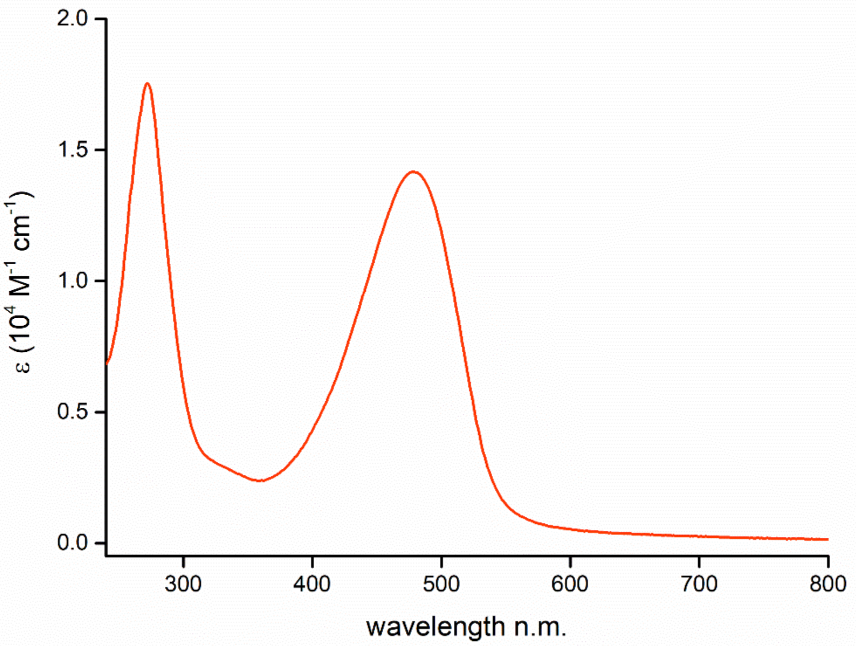

Figure S 188. The UV–vis absorption spectra of **16b-H** (dichloromethane, 298 K).

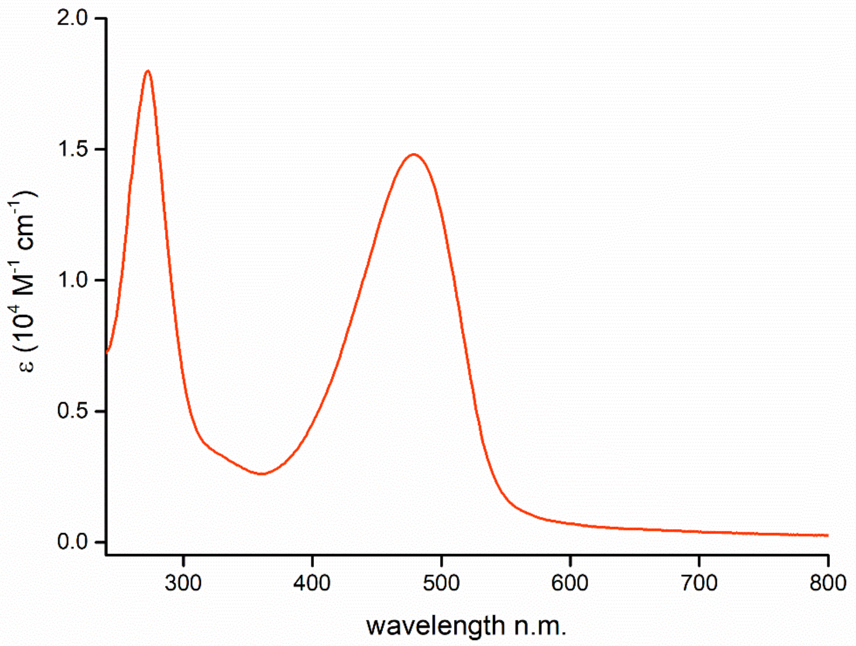

Figure S 189. The UV–vis absorption spectra of **17b-H** (dichloromethane, 298 K).

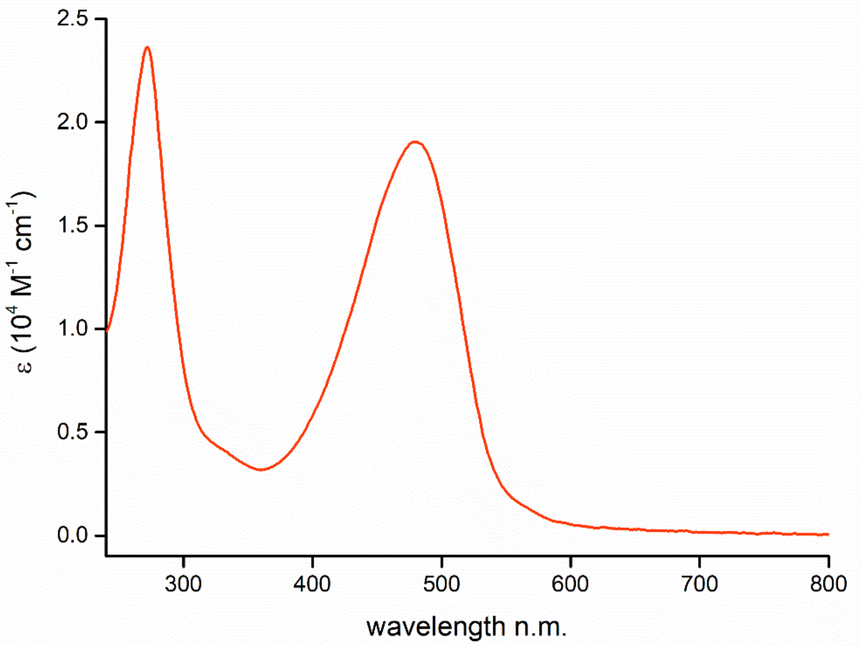

Figure S 190. The UV–vis absorption spectra of **18b-H** (dichloromethane, 298 K).

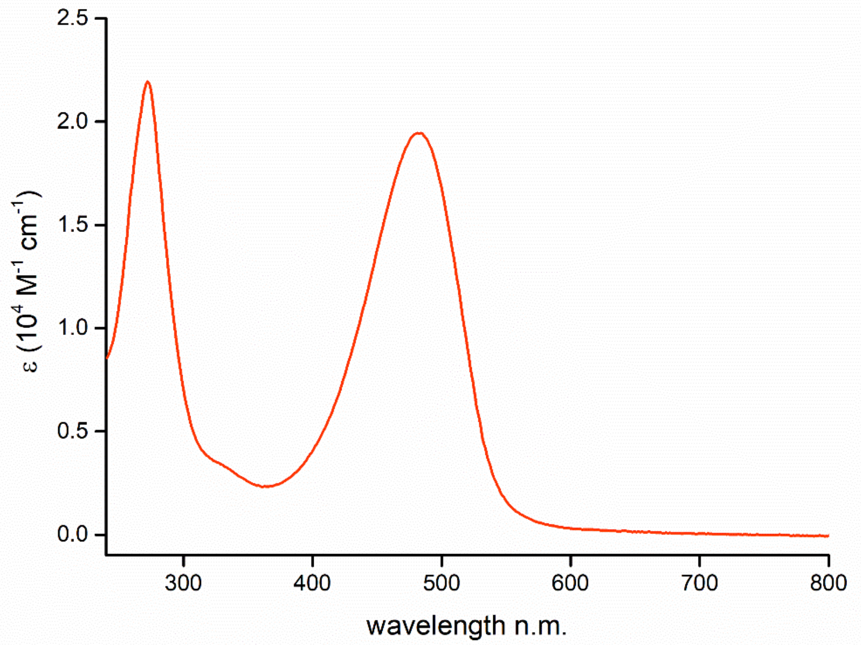

Figure S 191. The UV–vis absorption spectra of **19b-H** (dichloromethane, 298 K).

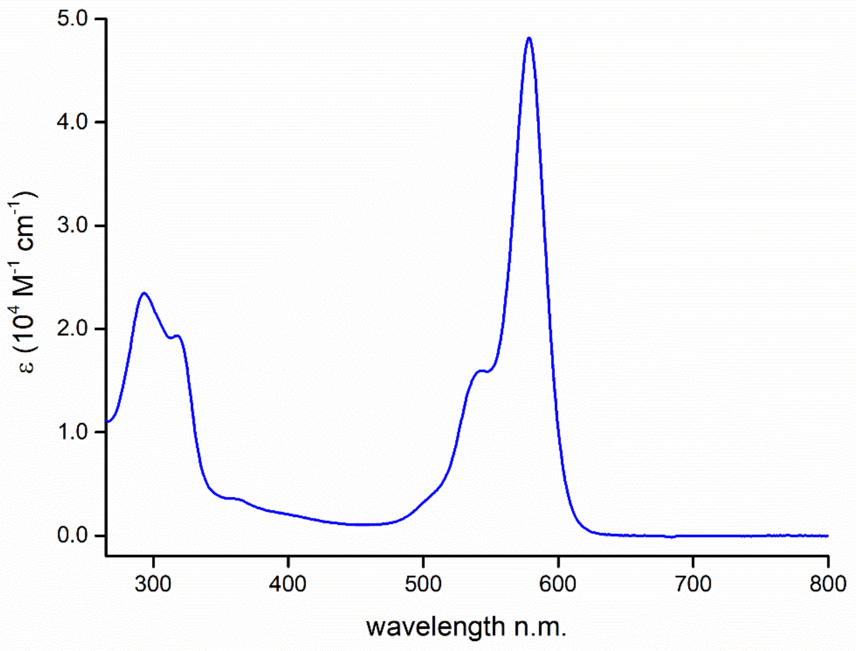

Figure S 192. The UV–vis absorption spectra of **20a-Pb** (dichloromethane, 298 K).

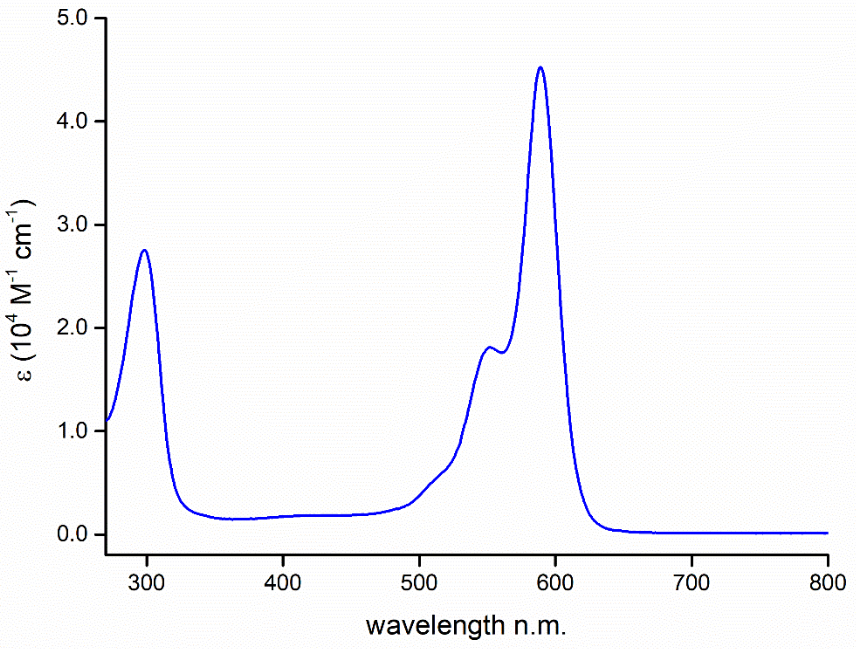

Figure S 193. The UV–vis absorption spectra of **20b-Pb** (dichloromethane, 298 K).

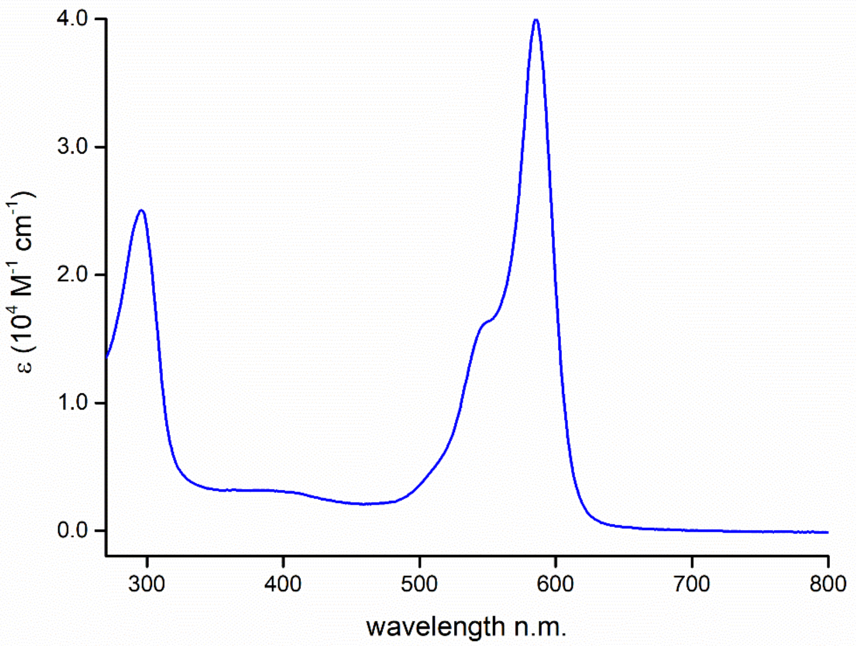

Figure S 194. The UV–vis absorption spectra of **21b-Pb** (dichloromethane, 298 K).

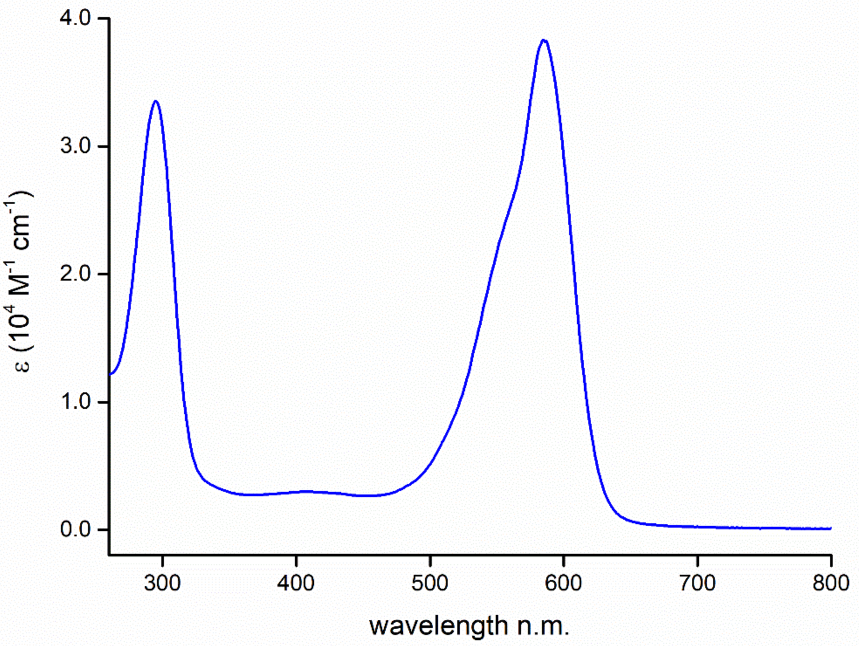

Figure S 195. The UV–vis absorption spectra of **21b-Cd** (dichloromethane, 298 K).

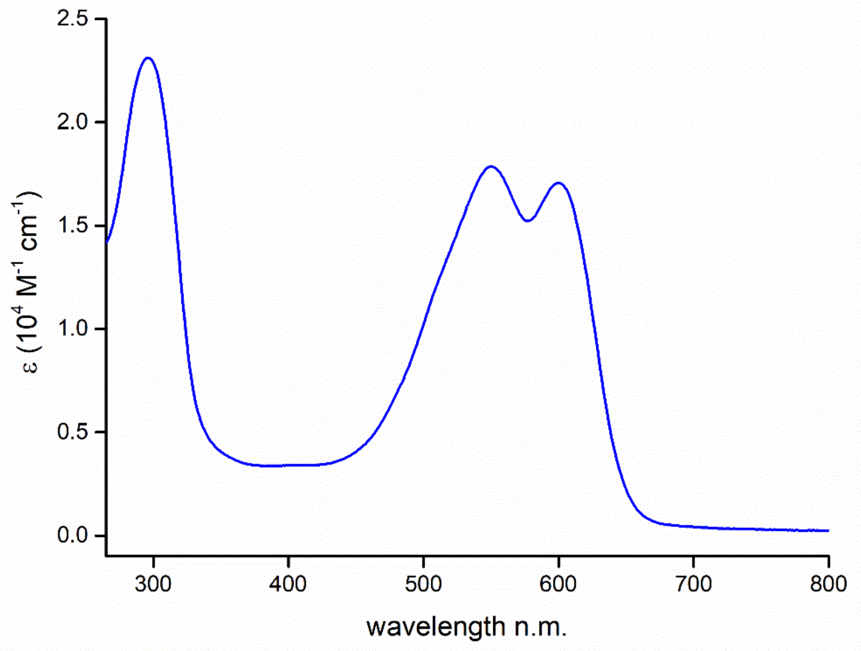

Figure S 196. The UV–vis absorption spectra of **21b-Hg** (dichloromethane, 298 K).

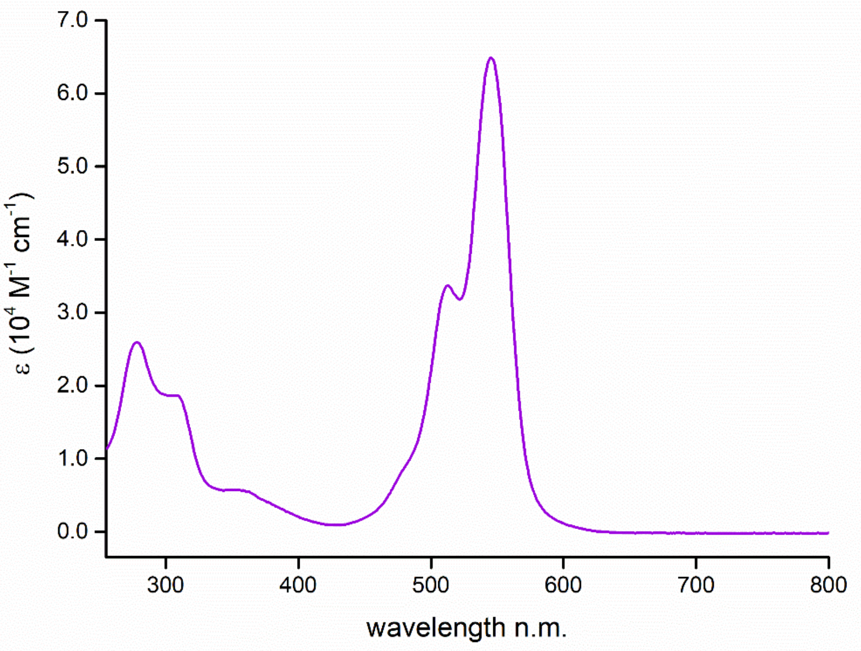

Figure S 197. The UV–vis absorption spectra of **22a-Zn** (dichloromethane, 298 K).

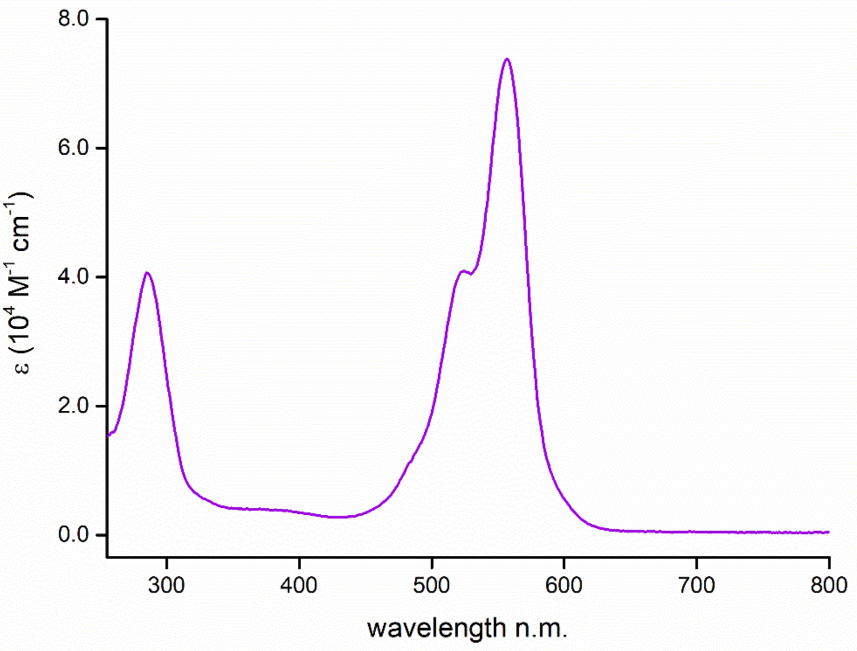

Figure S 198. The UV–vis absorption spectra of **22b-Cd** (dichloromethane, 298 K).

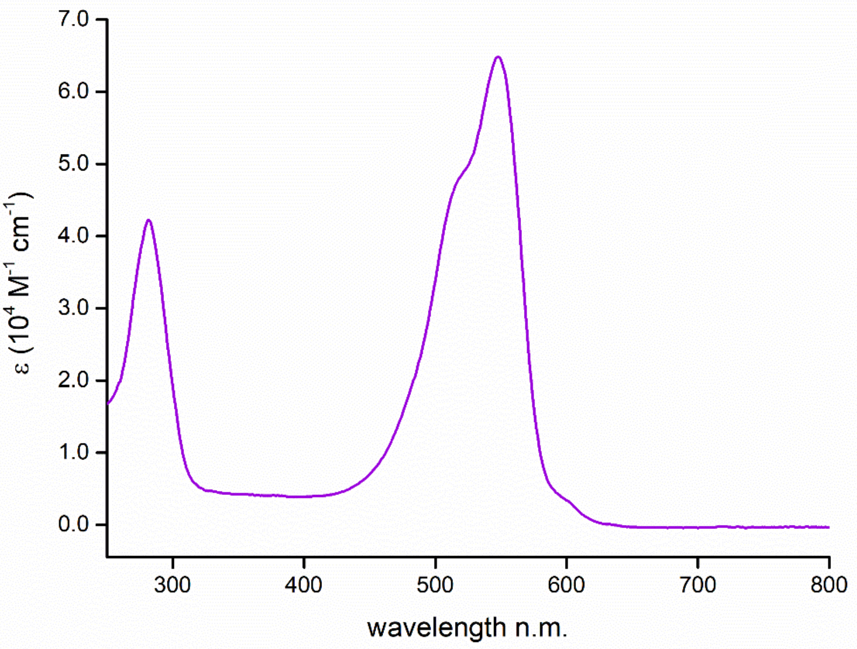

Figure S 199. The UV–vis absorption spectra of **22b-Hg** (dichloromethane, 298 K).

X-ray structure models

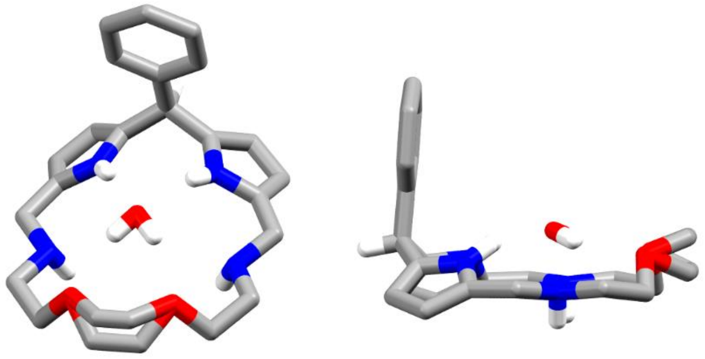

Figure S 200. The X-ray molecular structure of **14a-H₇** (selected hydrogen atoms omitted for clarity). Left: front view, right: side view.

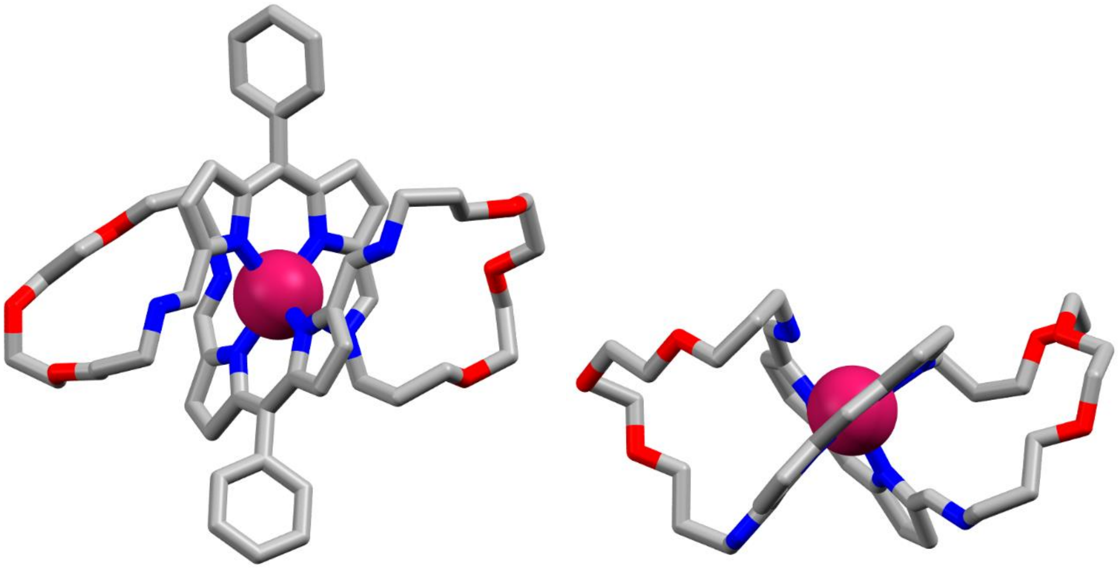

Figure S 201. The X-ray molecular structure of **22a-Zn** (hydrogen atoms omitted for clarity). Left: front view, right: side view (*meso*-Ph rings omitted for clarity).

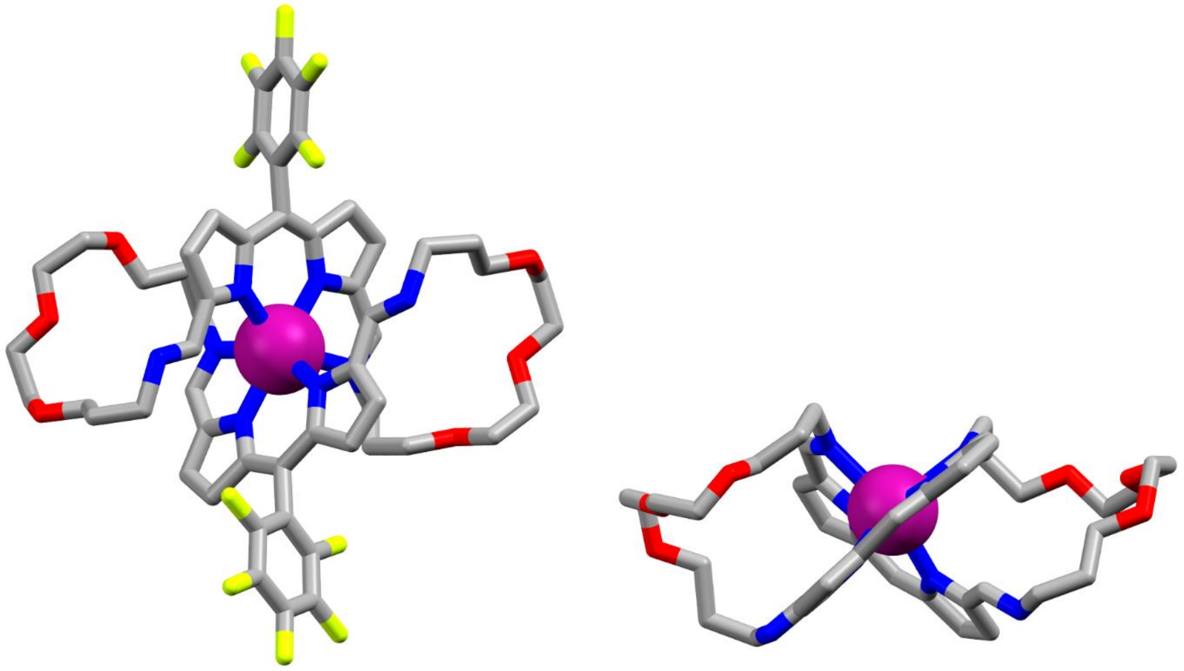

Figure S 202. The X-ray molecular structure of **22b-Cd** (hydrogen atoms omitted for clarity). Left: front view, right: side view (*meso*-C_6F_5 rings omitted for clarity).

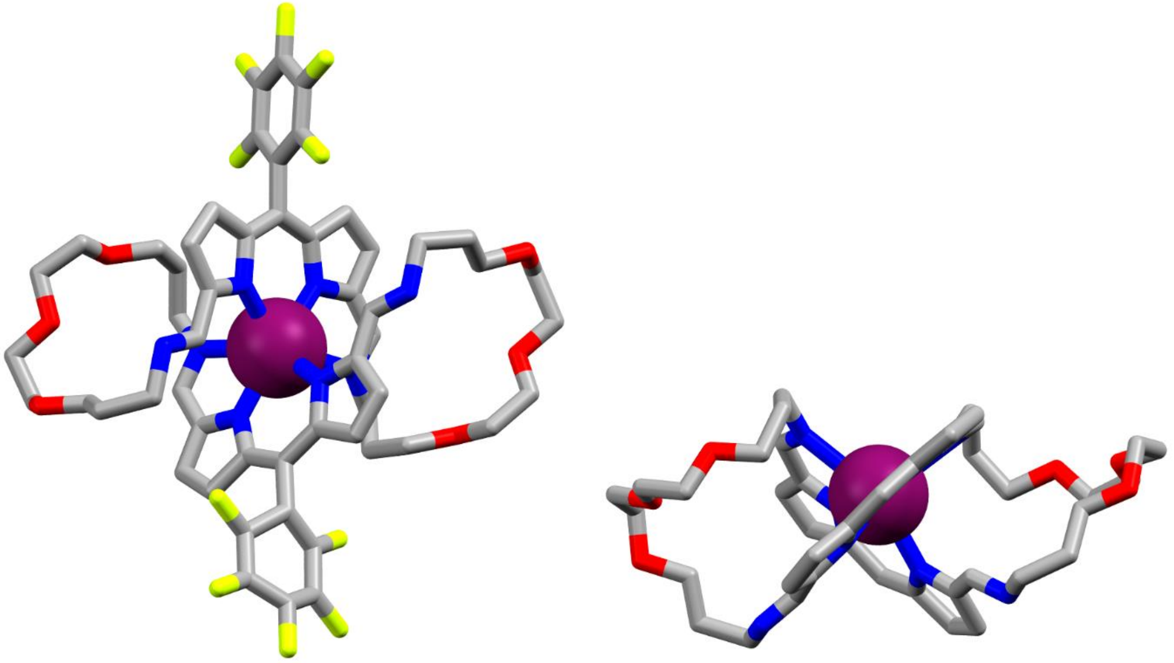

Figure S 203. The X-ray molecular structure of **22b-Hg** (hydrogen atoms omitted for clarity). Left: front view, right: side view (*meso*-C_6F_5 rings omitted for clarity).

DFT-calculated Cartesian coordinates of 14b-H.

C	-0.469700	-3.447500	-0.530600
C	0.723600	-2.705300	-0.567600
N	0.405000	-1.390200	-0.463500
C	-0.958700	-1.242800	-0.349200
C	-1.520600	-2.537900	-0.409200
C	-1.596000	0.016600	-0.131600
C	-3.084100	0.013000	-0.007400
C	-0.919100	1.219100	0.001500
N	0.466600	1.307500	-0.117000
C	0.773100	2.556700	0.186300
C	-0.406500	3.345400	0.517200
C	-1.472700	2.509300	0.380000
C	2.138900	3.087900	0.224500
C	2.093600	-3.173400	-0.682100
N	3.117500	2.577000	-0.413300
N	3.051500	-2.368800	-0.933600
O	5.493500	1.332700	0.707400
C	5.564300	2.260800	-0.358800
C	4.404300	3.240500	-0.273400
C	4.396300	-2.910600	-1.013600
C	5.424300	-1.887900	-0.550900
O	5.209600	-1.602000	0.816800
C	6.233000	-0.848400	1.440500
C	6.605800	0.461000	0.752800
C	-3.893500	0.600500	-0.985900
C	-5.282800	0.608700	-0.884700
C	-5.895900	0.019700	0.218200

C	-5.117400	-0.572700	1.209700
C	-3.730100	-0.569400	1.088400
F	-3.013400	-1.137200	2.066700
F	-5.704200	-1.132500	2.273300
F	-7.226500	0.022800	0.325300
F	-6.028800	1.174100	-1.839900
F	-3.340400	1.169500	-2.063700
H	-0.539600	-4.524000	-0.597000
H	1.040300	-0.587900	-0.432700
H	-2.574700	-2.766800	-0.361000
H	-0.412700	4.389300	0.803700
H	-2.517000	2.733700	0.546100
H	2.257500	3.990200	0.848300
H	2.236600	-4.254400	-0.532600
H	6.508200	2.831600	-0.324000
H	5.526200	1.732700	-1.324800
H	4.522600	3.940200	-1.114300
H	4.477900	3.828700	0.655700
H	4.618100	-3.133200	-2.068100
H	4.509100	-3.847500	-0.445400
H	6.437000	-2.295600	-0.717600
H	5.312500	-0.983200	-1.164300
H	5.867500	-0.640400	2.450400
H	7.158500	-1.446600	1.527600
H	7.442700	0.916900	1.310400
H	6.979000	0.258700	-0.262500

DFT-calculated Cartesian coordinates of S1.

C	-1.425700	3.508700	0.080500
C	-0.221700	2.817400	-0.142900
N	-0.508100	1.494900	-0.238500
C	-1.858300	1.289300	-0.080700
C	-2.447400	2.558500	0.121600
C	-2.458300	-0.007200	-0.111300
C	-1.744600	-1.185500	-0.277800
N	-0.361000	-1.191900	-0.429100
C	-0.017600	-2.460400	-0.572400
C	-1.172500	-3.346600	-0.517200
C	-2.259000	-2.544700	-0.337200
C	1.359300	-2.921500	-0.767000
C	1.138400	3.314300	-0.253900
N	2.350700	-2.131100	-0.912000
C	3.660000	-2.726200	-1.099800
N	2.109000	2.504100	-0.431900
C	3.455000	3.023200	-0.539100
O	5.698700	2.619600	0.115700
O	7.088100	0.107400	-0.011700
O	5.912700	-2.729300	-0.345600
C	7.763400	-1.139500	0.044200
C	6.940400	-2.328700	0.541800
C	4.675200	-2.051400	-0.177000
C	4.362100	2.307500	0.463700
C	6.677600	2.218700	1.052200
C	6.823100	0.710400	1.241800
C	-3.939800	-0.072400	0.055800
C	-4.523600	-0.640700	1.193400
C	-5.905300	-0.705100	1.357900
C	-6.741000	-0.191400	0.369500

C	-6.190600	0.382000	-0.774300
C	-4.806600	0.435900	-0.918300
F	-4.312400	0.984500	-2.035300
F	-6.991200	0.870100	-1.727700
F	-8.066100	-0.248800	0.517400
F	-6.431800	-1.250700	2.459400
F	-3.753400	-1.135400	2.170200
H	-1.522000	4.578700	0.199500
H	0.144300	0.718000	-0.382700
H	-3.498600	2.742900	0.286700
H	-1.150900	-4.424800	-0.610700
H	-3.295700	-2.841100	-0.261900
H	1.481300	-4.017200	-0.787200
H	1.266100	4.404300	-0.175600
H	3.676500	-3.814800	-0.924700
H	3.975300	-2.552500	-2.137300
H	3.837000	2.786000	-1.540400
H	3.519000	4.115200	-0.403900
H	8.666400	-1.056400	0.675200
H	8.083500	-1.345300	-0.981900
H	7.623200	-3.183500	0.630000
H	6.532600	-2.132900	1.546500
H	4.761700	-0.987200	-0.426200
H	4.333800	-2.132400	0.868400
H	4.161900	1.229200	0.406100
H	4.134400	2.638100	1.491300
H	6.483200	2.668800	2.042100
H	7.621800	2.620200	0.672200
H	7.655200	0.541800	1.946300
H	5.919200	0.284800	1.703400

DFT-calculated Cartesian coordinates of 16b-H.

O	7.249500	1.417600	-0.318400
O	7.162600	-1.401500	-1.107500
O	5.082600	-2.105600	0.734600
O	4.810000	1.948900	1.054600
C	7.272200	-2.405800	-0.115500
C	5.907900	-3.042500	0.072000
C	3.721800	-2.464100	0.864200
C	2.960300	-2.529600	-0.480900
C	2.797100	2.731000	-0.183000
C	3.459100	2.340600	1.161500
C	5.720100	2.960100	0.671800
C	7.116200	2.367900	0.719300

C	8.348400	0.546900	-0.156300
C	8.282900	-0.544100	-1.219900
C	-2.105300	3.495800	0.032100
C	-0.907800	2.764100	-0.068700
N	-1.225000	1.447000	-0.107000
C	-2.587200	1.279900	-0.035700
C	-3.153400	2.573000	0.058000
C	-3.211200	-0.006700	-0.073100
C	-2.504100	-1.198600	-0.168500
N	-1.115600	-1.213400	-0.213500
C	-0.762900	-2.476900	-0.369000
C	-1.922000	-3.356700	-0.431700

C	-3.016700	-2.554100	-0.297700
C	0.637700	-2.891900	-0.497200
C	0.479400	3.197600	-0.125300
N	1.598000	-2.077000	-0.292300
N	1.410000	2.323400	-0.152600
C	-4.701000	-0.047700	-0.014500
C	-5.372300	-0.608900	1.078000
C	-6.762900	-0.653700	1.144500
C	-7.519500	-0.128500	0.100000
C	-6.881500	0.436800	-1.001500
C	-5.490200	0.471300	-1.047500
F	-4.912200	1.010400	-2.128500
F	-7.605900	0.934700	-2.009400
F	-8.852600	-0.167300	0.153900
F	-7.373600	-1.191700	2.205600
F	-4.680900	-1.114300	2.106600
H	8.002700	-3.175300	-0.420400
H	7.598500	-1.994300	0.850300
H	6.009700	-3.968900	0.664100
H	5.507500	-3.315100	-0.915500
H	3.611400	-3.418500	1.406300
H	3.268300	-1.675300	1.469600
H	3.454800	-1.819800	-1.155800
H	3.018900	-3.535500	-0.930600
H	2.924400	3.804900	-0.399400
H	3.300900	2.155200	-0.968500
H	3.340600	3.164600	1.886000
H	2.930900	1.466400	1.550300
H	5.667900	3.821300	1.361100
H	5.515400	3.328700	-0.344900
H	7.261700	1.894200	1.702700
H	7.863600	3.174400	0.618500
H	9.307400	1.085500	-0.267700
H	8.343400	0.106400	0.853900
H	9.226300	-1.115800	-1.197000
H	8.200300	-0.069600	-2.202900
H	-2.182000	4.572800	0.083400
H	-0.585700	0.646600	-0.166300
H	-4.207800	2.792700	0.138200
H	-1.900000	-4.431600	-0.558300
H	-4.056900	-2.848500	-0.304100
H	0.804500	-3.939300	-0.799200
H	0.659900	4.282900	-0.147700

DFT-calculated Cartesian coordinates of 17b-H.

C	-2.835800	3.499800	0.156900
C	-1.622100	2.810000	-0.011800
N	-1.901500	1.484900	-0.106700
C	-3.259100	1.280100	-0.009900
C	-3.856900	2.549200	0.162000
C	-3.867300	-0.010600	-0.099100
C	-3.155200	-1.191000	-0.252900
N	-1.765400	-1.204000	-0.296300
C	-1.419300	-2.464800	-0.489400
C	-2.580300	-3.339100	-0.587200
C	-3.672300	-2.539100	-0.430400
C	-0.027400	-2.908600	-0.595100
C	-0.273000	3.338700	-0.100400
N	0.956200	-2.131200	-0.356900
N	0.740700	2.571400	-0.225900
C	2.955500	2.707000	0.806900
O	4.258300	3.199500	0.544300
C	2.050900	3.181300	-0.332100
O	4.347500	-2.712300	0.921300
C	2.935400	-2.723400	0.933800
C	2.297900	-2.663600	-0.471300
O	6.865900	-2.735300	-0.428300
C	6.464000	-3.706800	0.515000
C	4.961200	-3.884900	0.420100
C	5.155400	3.039800	1.634400
C	6.544900	3.526000	1.231900
O	7.338100	2.569200	0.556900
C	6.931900	2.272200	-0.771000
C	7.863600	1.218100	-1.333500
O	7.652900	0.005600	-0.639700
C	8.426500	-1.068100	-1.128200
C	8.191000	-2.279400	-0.247500
C	-5.356700	-0.067300	-0.027900
C	-6.012500	-0.668300	1.052500
C	-7.402000	-0.728400	1.129500
C	-8.172600	-0.178500	0.108200
C	-7.549800	0.426700	-0.980800
C	-6.159400	0.476000	-1.037600
F	-5.595200	1.054400	-2.105300
F	-8.287800	0.948600	-1.966300
F	-9.504600	-0.231800	0.172300
F	-7.998200	-1.304900	2.178500
F	-5.306600	-1.198800	2.058400
H	-2.937800	4.570800	0.261000
H	-1.246400	0.704600	-0.214100
H	-4.914600	2.733200	0.278700
H	-2.558900	-4.409300	-0.748000
H	-4.713700	-2.828400	-0.449700
H	0.113900	-3.960200	-0.895700
H	-0.192400	4.435900	-0.064200
H	2.579500	3.093800	1.768300
H	2.945300	1.608500	0.862700
H	2.507000	2.855300	-1.275400
H	2.018600	4.283300	-0.337300
H	2.631700	-1.827300	1.481700
H	2.549200	-3.601600	1.478600
H	2.325400	-3.651100	-0.962200
H	2.897300	-1.964900	-1.067300
H	6.721500	-3.389200	1.537500
H	6.960600	-4.675200	0.326900
H	4.659500	-4.767800	1.010100
H	4.688800	-4.070800	-0.629200
H	4.799000	3.627300	2.498300
H	5.218300	1.985800	1.945900
H	6.435200	4.448700	0.638300
H	7.103500	3.772100	2.141200
H	5.900200	1.903700	-0.800100
H	6.983600	3.176100	-1.402000
H	8.910000	1.551400	-1.231100
H	7.653300	1.097900	-2.410500
H	8.158000	-1.307700	-2.170900
H	9.502600	-0.821900	-1.109100
H	8.364100	-1.994500	0.802000
H	8.921200	-3.065800	-0.508700

DFT-calculated Cartesian coordinates of 18b-H.

C	3.471100	2.826600	-1.941800
C	2.254900	2.321800	-1.447000
N	2.532200	1.239300	-0.679400
C	3.886900	1.010000	-0.649500
C	4.491000	2.016800	-1.437400
C	4.471900	-0.089000	0.053700
C	3.728400	-1.085700	0.671900
N	2.339900	-1.107300	0.598400
C	1.954000	-2.183300	1.260800
C	3.085900	-2.911600	1.817900
C	4.201900	-2.222900	1.445200
C	0.553400	-2.601200	1.369800
C	0.889700	2.788600	-1.623000
N	-0.397500	-1.971900	0.796900
N	-0.052100	2.285800	-0.920400
O	-5.857400	3.428600	0.327900
O	-7.557200	1.183800	0.931600
O	-8.456500	-1.244000	-0.231000
O	-6.519000	-3.178300	-1.177300
O	-3.634500	-3.230800	-0.336000
O	-3.099900	3.892000	0.349800
C	-7.232000	3.550600	0.633400

C	-7.661500	2.439200	1.569300
C	-3.909500	4.326000	-0.725700
C	-5.305400	4.612200	-0.208200
C	-2.309900	-2.752200	-0.476300
C	-1.740700	-2.500500	0.922000
C	-8.152300	0.153500	1.692600
C	-7.884300	-1.200100	1.064200
C	-5.577600	-4.228600	-1.308700
C	-4.195300	-3.661000	-1.562600
C	-8.799000	-2.527400	-0.710300
C	-7.730700	-3.610300	-0.595000
C	-1.403000	2.777900	-1.090300
C	-1.725400	3.766600	0.054300
C	5.960700	-0.152200	0.099000
C	6.670900	-1.187600	-0.520200
C	8.061900	-1.251400	-0.483400
C	8.779500	-0.262400	0.184400
C	8.102600	0.782200	0.809500
C	6.711900	0.827500	0.759900
F	6.095400	1.839000	1.383500
F	8.789300	1.728900	1.457900
F	10.112400	-0.315500	0.226000
F	8.710400	-2.250900	-1.090800
F	6.019700	-2.149700	-1.184800
H	3.579100	3.692500	-2.579600
H	1.867300	0.597300	-0.236400
H	5.550700	2.119100	-1.619800
H	3.032100	-3.821000	2.402600
H	5.231600	-2.455700	1.678100
H	0.377700	-3.512100	1.965800
H	0.732900	3.573300	-2.378500
H	-7.436400	4.509600	1.139700
H	-7.838900	3.521300	-0.286200
H	-7.039700	2.466400	2.479500
H	-8.704900	2.637400	1.872500
H	-3.505100	5.247600	-1.180400
H	-3.970700	3.559000	-1.511500
H	-5.265400	5.403000	0.559500
H	-5.911600	4.992100	-1.048900
H	-1.682700	-3.493700	-0.999600
H	-2.278200	-1.821800	-1.062900
H	-2.369700	-1.748400	1.414900
H	-1.796100	-3.430300	1.512800
H	-7.746400	0.139800	2.719700
H	-9.241500	0.313700	1.768500
H	-8.333800	-1.958300	1.727700
H	-6.804000	-1.391300	1.009500
H	-5.862800	-4.891400	-2.143500
H	-5.537600	-4.841400	-0.394500
H	-4.264700	-2.831200	-2.283300
H	-3.564500	-4.445800	-2.014300
H	-9.698500	-2.913300	-0.195400
H	-9.053400	-2.381000	-1.764500
H	-7.563400	-3.891000	0.458100
H	-8.123300	-4.508900	-1.103500
H	-1.558300	3.255700	-2.071100
H	-2.091000	1.929400	-1.004400
H	-1.281700	4.749000	-0.185700
H	-1.255600	3.398400	0.970100

DFT-calculated Cartesian coordinates of 19b-H.

C	-4.239400	-3.382700	-0.945100
C	-2.982100	-2.784500	-0.748000
N	-3.184000	-1.506100	-0.339800
C	-4.532300	-1.242900	-0.259500
C	-5.207100	-2.424600	-0.642100
C	-5.062600	0.018700	0.149100
C	-4.282800	1.124300	0.456600
N	-2.895400	1.096600	0.354900
C	-2.481500	2.294100	0.733200
C	-3.590400	3.161000	1.108000
C	-4.723900	2.426400	0.930900
C	-1.077000	2.707900	0.760600
C	-1.656700	-3.355100	-0.907600
N	-0.117500	1.964600	0.366600
N	-0.606000	-2.668400	-0.673300
O	6.276600	-2.628500	1.674000
O	7.163000	-0.047500	2.584200
O	7.976500	1.522400	0.322900
O	6.067500	2.908900	-1.407500
O	3.146400	3.047200	-0.867900
O	2.856700	-3.097900	-1.735000
O	5.667900	-2.568400	-1.086500
C	8.319100	2.453200	-0.682400
C	7.274700	3.516000	-1.002300
C	5.137200	3.842600	-1.922000
C	3.791700	3.174200	-2.119200
C	1.851100	2.487600	-0.967600
C	1.222300	2.515300	0.426400
C	0.683500	-3.309800	-0.826100
C	1.582500	-2.488600	-1.750900
C	3.806100	-2.464200	-2.570700
C	5.167100	-3.077500	-2.304300
C	6.744700	-3.316800	-0.565000
C	7.285100	-2.638800	0.679500
C	6.712000	-2.379000	2.993800
C	7.684000	-1.217800	3.176400
C	8.046700	1.053200	2.662500
C	7.694500	2.075100	1.598000
C	-6.547700	0.130800	0.244100
C	-7.275300	0.952600	-0.623900
C	-8.661500	1.064500	-0.543200
C	-9.355300	0.342500	0.424500
C	-8.659800	-0.485000	1.303100
C	-7.274200	-0.582200	1.204700
F	-6.637800	-1.377900	2.073200
F	-9.323500	-1.173700	2.237700
F	-10.683400	0.443400	0.510600
F	-9.328100	1.854900	-1.391400
F	-6.645700	1.653700	-1.574500
H	-4.404300	-4.400600	-1.269100
H	-2.480700	-0.789700	-0.137300
H	-6.279000	-2.545500	-0.692500
H	-3.508500	4.183300	1.454300
H	-5.745300	2.728900	1.114500
H	-0.902700	3.727200	1.143800
H	-1.627200	-4.407600	-1.228100
H	9.254300	2.982600	-0.423000
H	8.505600	1.855300	-1.579500
H	7.690200	4.152600	-1.804600
H	7.101300	4.174100	-0.134100
H	5.491300	4.242500	-2.888200
H	5.009500	4.694700	-1.235300
H	3.184000	3.791600	-2.803400
H	3.936600	2.191100	-2.594700
H	1.227500	3.070300	-1.666900
H	1.887500	1.453100	-1.342300
H	1.831400	1.886300	1.087400
H	1.255000	3.547100	0.814700
H	1.171800	-3.342600	0.156200
H	0.613500	-4.344200	-1.200800
H	1.629200	-1.449100	-1.391300
H	1.162000	-2.469500	-2.771000
H	3.539800	-2.599600	-3.633400
H	3.852200	-1.383000	-2.367900
H	5.850600	-2.840900	-3.138800
H	5.056800	-4.173000	-2.263400
H	7.566500	-3.394900	-1.299600
H	6.418300	-4.340400	-0.318100
H	7.599400	-1.613400	0.442000
H	8.170500	-3.209800	1.010600
H	7.200800	-3.272400	3.425300
H	5.799600	-2.187200	3.566800
H	8.663300	-1.459500	2.731300
H	7.852400	-1.086100	4.260500
H	7.995200	1.526700	3.658500
H	9.086600	0.728100	2.499800
H	6.634800	2.354700	1.674600
H	8.302600	2.976600	1.781900

References

[1] CrysAlis PRO. CrysAlis PRO. version 171.37.35, Agilent Technologies. Agilent Technologies: Yanton, Oxfrodshire, England.
[2] G. M. Sheldrick, *Acta Crystallogr. A Found. Crystallogr.* **2008**, *64*, 112–122.
[3] M. C. Burla, R. Caliandro, B. Carrozzini, G. L. Cascarano, C. Cuocci, C. Giacovazzo, M. Mallamo, A. Mazzone, G. Polidori, *J. Appl. Crystallogr.* **2015**, *48*, 306–309.
[4] G. M. Sheldrick, *Acta Crystallogr. C Struct. Chem.* **2015**, *71*, 3–8.
[5] A. L. Spek, *Acta Crystallogr. C Struct. Chem.* **2015**, *71*, 9–18.
[6] J. Frisch, G. W. Trucks, H. B. Schlegel, G. E. Scuseria, M. A. Robb, J. R. Cheeseman, G. Scalmani, V. Barone, G. A. Petersson, H. Nakatsuji, X. Li, M. Caricato, A. V. Marenich, J. Bloino, B. G. Janesko, R. Gomperts, B. Mennucci, H. P. Hratchian, J. V. Ort, **2019**.
[7] C. Lee, W. Yang, R. G. Parr, *Phys. Rev. B* **1988**, *37*, 785–789.
[8] A. D. Becke, *J. Chem. Phys.* **1993**, *98*, 5648–5652.
[9] C. Mongin, A. M. Ardoy, R. Méreau, D. M. Bassani, B. Bibal, *Chem. Sci.* **2020**, *11*, 1478–1484.
