# Supplementary material for: Crownphyrins: Metal‐Mediated Transformations of the Porphyrin‐Crown Ether Hybrids
Source: Angew Chem Int Ed Engl. 2022 Nov 9;61(49):e202211671. doi: 10.1002/anie.202211671 (PMC10098552; doi:10.1002/anie.202211671)

# checkCIF/PLATON report

Structure factors have been supplied for datablock(s) 14aH3, 14aH7, 15aH72HCl, 15bH, 20aPb, 22aZn, 22bCd, 22bHg, 22bZn

THIS REPORT IS FOR GUIDANCE ONLY. IF USED AS PART OF A REVIEW PROCEDURE FOR PUBLICATION, IT SHOULD NOT REPLACE THE EXPERTISE OF AN EXPERIENCED CRYSTALLOGRAPHIC REFEREE.

No syntax errors found.  CIF dictionary  Interpreting this report

## Datablock: 14aH3

---

```
Bond precision:   C-C = 0.0034 A          Wavelength=0.71073

Cell:              a=12.185(3)     b=9.096(2)         c=20.334(3)
                   alpha=90        beta=102.14(3)     gamma=90
Temperature:       100 K

                 Calculated                   Reported
Volume           2203.3(8)                    2203.3(8)
Space group      P 21/n                       P 21/n
Hall group       -P 2yn                       -P 2yn
Moiety formula   C23 H26 N4 O2, H2 O          C23 H26 N4 O2, H2 O
Sum formula      C23 H28 N4 O3                C23 H28 N4 O3
Mr               408.49                       408.49
Dx,g cm-3        1.232                        1.231
Z                4                            4
Mu (mm-1)        0.083                        0.083
F000             872.0                        872.0
F000'            872.36
h,k,lmax         20,15,34                     18,11,33
Nref             11040                        6315
Tmin,Tmax        0.972,0.988                  0.879,1.000
Tmin'            0.952

Correction method= # Reported T Limits: Tmin=0.879 Tmax=1.000
AbsCorr = MULTI-SCAN

Data completeness= 0.572           Theta(max)= 36.782

                                                     wR2(reflections)=
R(reflections)= 0.0762( 3272)                        0.2137( 6315)

S = 1.033                  Npar= 275
```

The following ALERTS were generated. Each ALERT has the format
**test-name_ALERT_alert-type_alert-level.**
Click on the hyperlinks for more details of the test.

### Alert level C

```
PLAT906_ALERT_3_C Large K Value in the Analysis of Variance ......     19.879 Check
PLAT906_ALERT_3_C Large K Value in the Analysis of Variance ......      2.626 Check
PLAT906_ALERT_3_C Large K Value in the Analysis of Variance ......      4.662 Check
PLAT906_ALERT_3_C Large K Value in the Analysis of Variance ......      2.284 Check
PLAT910_ALERT_3_C Missing # of FCF Reflection(s) Below Theta(Min).          5 Note
```

### Alert level G

```
PLAT007_ALERT_5_G Number of Unrefined Donor-H Atoms .............           4 Report
PLAT883_ALERT_1_G No Info/Value for _atom_sites_solution_primary .     Please Do !
PLAT899_ALERT_4_G SHELXL97  is Deprecated and Succeeded by SHELXL/       2018 Note
PLAT912_ALERT_4_G Missing # of FCF Reflections Above STh/L=  0.600       3613 Note
PLAT941_ALERT_3_G Average HKL Measurement Multiplicity ...........        3.5 Low
PLAT950_ALERT_5_G Calculated (ThMax) and CIF-Reported Hmax Differ           2 Units
PLAT951_ALERT_5_G Calculated (ThMax) and CIF-Reported Kmax Differ           4 Units
PLAT956_ALERT_1_G Calculated (ThMax) and Actual (FCF) Hmax Differ           2 Units
PLAT957_ALERT_1_G Calculated (ThMax) and Actual (FCF) Kmax Differ           4 Units
PLAT978_ALERT_2_G Number C-C Bonds with Positive Residual Density.          2 Info
PLAT992_ALERT_5_G Repd & Actual _reflns_number_gt Values Differ by          2 Check
```

0 **ALERT level A** = Most likely a serious problem - resolve or explain
0 **ALERT level B** = A potentially serious problem, consider carefully
5 **ALERT level C** = Check. Ensure it is not caused by an omission or oversight
11 **ALERT level G** = General information/check it is not something unexpected

3 ALERT type 1 CIF construction/syntax error, inconsistent or missing data
1 ALERT type 2 Indicator that the structure model may be wrong or deficient
6 ALERT type 3 Indicator that the structure quality may be low
2 ALERT type 4 Improvement, methodology, query or suggestion
4 ALERT type 5 Informative message, check

# Datablock: 14aH7

| | | | |
|---|---|---|---|
| Bond precision: | C-C = 0.0000 A | | Wavelength=1.54184 |
| Cell: | a=8.520(2) | b=17.383(3) | c=15.051(3) |
| | alpha=90 | beta=90 | gamma=90 |
| Temperature: | 100 K | | |

| | Calculated | Reported |
|---|---|---|
| Volume | 2229.1(8) | 2229.1(8) |
| Space group | P n m a | P n m a |
| Hall group | -P 2ac 2n | -P 2ac 2n |
| Moiety formula | C20.38 H26.58 N3.54 O1.77, 0.114(C23 H30 N4 O2), H2 O | C23 H30 N4 O2, H2 O |
| Sum formula | C23 H32 N4 O3 | C23 H32 N4 O3 |
| Mr | 412.53 | 412.52 |
| Dx,g cm-3 | 1.229 | 1.229 |
| Z | 4 | 4 |
| Mu (mm-1) | 0.663 | 0.663 |
| F000 | 888.0 | 888.0 |
| F000' | 890.58 | |
| h,k,lmax | 10,21,18 | 10,21,18 |
| Nref | 2403 | 2398 |
| Tmin,Tmax | 0.895,0.930 | 0.590,1.000 |
| Tmin' | 0.825 | |

Correction method= # Reported T Limits: Tmin=0.590 Tmax=1.000
AbsCorr = MULTI-SCAN

Data completeness= 0.998 Theta(max)= 75.944

R(reflections)= 0.0746( 2308) wR2(reflections)= 0.1821( 2398)

S = 1.111 Npar= 276

---

The following ALERTS were generated. Each ALERT has the format
**test-name_ALERT_alert-type_alert-level.**
Click on the hyperlinks for more details of the test.

---

**Alert level B**

PLAT220_ALERT_2_B NonSolvent Resd 2 C Ueq(max)/Ueq(min) Range 7.7 Ratio

---

**Alert level C**

PLAT042_ALERT_1_C Calc. and Reported MoietyFormula Strings Differ Please Check
PLAT088_ALERT_3_C Poor Data / Parameter Ratio .................... 8.69 Note
PLAT222_ALERT_3_C NonSolvent Resd 2 H Uiso(max)/Uiso(min) Range 7.7 Ratio
PLAT906_ALERT_3_C Large K Value in the Analysis of Variance ...... 6.512 Check
PLAT918_ALERT_3_C Reflection(s) with I(obs) much Smaller I(calc) . 1 Check
PLAT934_ALERT_3_C Number of (Iobs-Icalc)/Sigma(W) > 10 Outliers .. 1 Check
PLAT939_ALERT_3_C Large Value of Not (SHELXL) Weight Optimized S . 16.66 Check

---

**Alert level G**

PLAT003_ALERT_2_G Number of Uiso or Uij Restrained non-H Atoms ... 17 Report

```
PLAT007_ALERT_5_G Number of Unrefined Donor-H Atoms .............          6 Report
PLAT012_ALERT_1_G N.O.K.   _shelx_res_checksum Found in CIF ......     Please Check
PLAT013_ALERT_1_G N.O.K.   _shelx_hkl_checksum Found in CIF ......     Please Check
PLAT169_ALERT_4_G The CIF-Embedded .res File Contains AFIX 1 Recds          2 Report
PLAT171_ALERT_4_G The CIF-Embedded .res File Contains EADP Records          2 Report
PLAT186_ALERT_4_G The CIF-Embedded .res File Contains ISOR Records          1 Report
PLAT300_ALERT_4_G Atom Site Occupancy of H1W       Constrained at      0.886 Check
PLAT300_ALERT_4_G Atom Site Occupancy of H11W      Constrained at      0.114 Check
PLAT301_ALERT_3_G Main Residue  Disorder .............(Resd  1  )       100% Note
PLAT301_ALERT_3_G Main Residue  Disorder .............(Resd  2  )       100% Note
PLAT302_ALERT_4_G Anion/Solvent/Minor-Residue Disorder (Resd  3  )       100% Note
PLAT302_ALERT_4_G Anion/Solvent/Minor-Residue Disorder (Resd  4  )       100% Note
PLAT304_ALERT_4_G Non-Integer Number of Atoms in ..... (Resd  1  )      52.27 Check
PLAT304_ALERT_4_G Non-Integer Number of Atoms in ..... (Resd  2  )       6.73 Check
PLAT304_ALERT_4_G Non-Integer Number of Atoms in ..... (Resd  3  )       2.66 Check
PLAT304_ALERT_4_G Non-Integer Number of Atoms in ..... (Resd  4  )       0.34 Check
PLAT398_ALERT_2_G Deviating  C-O-C   Angle From 120 for O9       .       93.7 Degree
PLAT720_ALERT_4_G Number of Unusual/Non-Standard Labels .........          9 Note
PLAT773_ALERT_2_G Check long C-C Bond in CIF: C1A      --C2A              1.82 Ang.
PLAT779_ALERT_4_G Suspect or Irrelevant (Bond) Angle(s) in CIF ...      37.80 Deg.
              C10  -O9   -C100    1_555   1_555   7_565 ......     #   15 Check
PLAT779_ALERT_4_G Suspect or Irrelevant (Bond) Angle(s) in CIF ...      37.40 Deg.
              C100 -C10  -C100    7_565   1_555   1_555 ......     #   19 Check
PLAT779_ALERT_4_G Suspect or Irrelevant (Bond) Angle(s) in CIF ...      40.00 Deg.
              C10  -C10  -C100    7_565   1_555   1_555 ......     #   20 Check
PLAT779_ALERT_4_G Suspect or Irrelevant (Bond) Angle(s) in CIF ...      40.00 Deg.
              C100 -C100 -C10     7_565   1_555   1_555 ......     #   23 Check
PLAT860_ALERT_3_G Number of Least-Squares Restraints ............        102 Note
PLAT883_ALERT_1_G No Info/Value for _atom_sites_solution_primary .     Please Do !
PLAT912_ALERT_4_G Missing # of FCF Reflections Above STh/L=  0.600          5 Note
PLAT965_ALERT_2_G The SHELXL WEIGHT Optimisation has not Converged     Please Check
PLAT978_ALERT_2_G Number C-C Bonds with Positive Residual Density.          3 Info
```

---

0 **ALERT level A** = Most likely a serious problem - resolve or explain
1 **ALERT level B** = A potentially serious problem, consider carefully
7 **ALERT level C** = Check. Ensure it is not caused by an omission or oversight
29 **ALERT level G** = General information/check it is not something unexpected

4 ALERT type 1 CIF construction/syntax error, inconsistent or missing data
6 ALERT type 2 Indicator that the structure model may be wrong or deficient
9 ALERT type 3 Indicator that the structure quality may be low
17 ALERT type 4 Improvement, methodology, query or suggestion
1 ALERT type 5 Informative message, check

---

# Datablock: 15aH72HCl

---

Bond precision: C-C = 0.0057 A    Wavelength=1.54184

| | | | |
|---|---|---|---|
| Cell: | a=13.411(5) | b=10.170(3) | c=20.383(6) |
| | alpha=90 | beta=97.98(3) | gamma=90 |
| Temperature: | 100 K | | |

| | Calculated | Reported |
|---|---|---|
| Volume | 2753.1(16) | 2753.1(16) |
| Space group | P 21/c | P 21/c |
| Hall group | -P 2ybc | -P 2ybc |
| Moiety formula | C27 H40 N4 O3, 2(Cl) | C27 H40 N4 O3, 2Cl |
| Sum formula | C27 H40 Cl2 N4 O3 | C27 H40 Cl2 N4 O3 |
| Mr | 539.53 | 539.53 |
| Dx,g cm-3 | 1.302 | 1.302 |
| Z | 4 | 4 |
| Mu (mm-1) | 2.403 | 2.403 |
| F000 | 1152.0 | 1152.0 |
| F000' | 1157.79 | |
| h,k,lmax | 16,12,24 | 16,12,24 |
| Nref | 4900 | 4896 |
| Tmin,Tmax | 0.771,0.845 | 0.755,0.841 |
| Tmin' | 0.714 | |

Correction method= # Reported T Limits: Tmin=0.755 Tmax=0.841
AbsCorr = ANALYTICAL

Data completeness= 0.999 Theta(max)= 66.994

R(reflections)= 0.0751( 3240) wR2(reflections)= 0.2295( 4896)

S = 1.087 Npar= 489

---

The following ALERTS were generated. Each ALERT has the format
**test-name_ALERT_alert-type_alert-level.**
Click on the hyperlinks for more details of the test.

---

## Alert level C

```
PLAT042_ALERT_1_C Calc. and Reported MoietyFormula Strings  Differ     Please Check
PLAT094_ALERT_2_C Ratio of Maximum / Minimum Residual Density ....       2.29 Report
PLAT220_ALERT_2_C NonSolvent   Resd 1  C   Ueq(max)/Ueq(min) Range        5.6 Ratio
PLAT222_ALERT_3_C NonSolvent Resd 1  H   Uiso(max)/Uiso(min) Range        5.6 Ratio
PLAT230_ALERT_2_C Hirshfeld Test Diff for    N27      --C4       .        5.3 s.u.
PLAT234_ALERT_4_C Large Hirshfeld Difference N25      --C26      .       0.18 Ang.
PLAT234_ALERT_4_C Large Hirshfeld Difference C19      --C20      .       0.20 Ang.
PLAT340_ALERT_3_C Low Bond Precision on  C-C Bonds ..............     0.00569 Ang.
PLAT906_ALERT_3_C Large K Value in the Analysis of Variance ......      6.788 Check
PLAT906_ALERT_3_C Large K Value in the Analysis of Variance ......      2.009 Check
PLAT911_ALERT_3_C Missing FCF Refl Between Thmin & STh/L=    0.597          6 Report
```

---

## Alert level G

```
PLAT002_ALERT_2_G Number of Distance or Angle Restraints on AtSite         38 Note
PLAT003_ALERT_2_G Number of Uiso or Uij Restrained non-H Atoms ...         36 Report
PLAT007_ALERT_5_G Number of Unrefined Donor-H Atoms .............          10 Report
```

```
PLAT168_ALERT_4_G The CIF-Embedded .res File Contains EXYZ Records          2 Report
PLAT171_ALERT_4_G The CIF-Embedded .res File Contains EADP Records          2 Report
PLAT175_ALERT_4_G The CIF-Embedded .res File Contains SAME Records          1 Report
PLAT186_ALERT_4_G The CIF-Embedded .res File Contains ISOR Records          1 Report
PLAT230_ALERT_2_G Hirshfeld Test Diff for    O21      --C20      .        5.3 s.u.
PLAT230_ALERT_2_G Hirshfeld Test Diff for    O21      --C22      .        7.8 s.u.
PLAT301_ALERT_3_G Main Residue  Disorder .............(Resd  1  )        56% Note
PLAT302_ALERT_4_G Anion/Solvent/Minor-Residue Disorder (Resd  2  )      100% Note
PLAT302_ALERT_4_G Anion/Solvent/Minor-Residue Disorder (Resd  4  )      100% Note
PLAT304_ALERT_4_G Non-Integer Number of Atoms in ..... (Resd  2  )      0.65 Check
PLAT304_ALERT_4_G Non-Integer Number of Atoms in ..... (Resd  4  )      0.34 Check
PLAT811_ALERT_5_G No ADDSYM Analysis: Too Many Excluded Atoms ....         ! Info
PLAT860_ALERT_3_G Number of Least-Squares Restraints ............        251 Note
PLAT883_ALERT_1_G No Info/Value for _atom_sites_solution_primary .     Please Do !
PLAT909_ALERT_3_G Percentage of I>2sig(I) Data at Theta(Max) Still       33% Note
PLAT941_ALERT_3_G Average HKL Measurement Multiplicity ..........        4.5 Low
PLAT961_ALERT_5_G Dataset Contains no Negative Intensities .......     Please Check
PLAT967_ALERT_5_G Note: Two-Theta Cutoff Value in Embedded .res ..      134.0 Degree
PLAT978_ALERT_2_G Number C-C Bonds with Positive Residual Density.          1 Info
```

---

 0 **ALERT level A** = Most likely a serious problem - resolve or explain
 0 **ALERT level B** = A potentially serious problem, consider carefully
11 **ALERT level C** = Check. Ensure it is not caused by an omission or oversight
22 **ALERT level G** = General information/check it is not something unexpected

 2 ALERT type 1 CIF construction/syntax error, inconsistent or missing data
 8 ALERT type 2 Indicator that the structure model may be wrong or deficient
 9 ALERT type 3 Indicator that the structure quality may be low
10 ALERT type 4 Improvement, methodology, query or suggestion
 4 ALERT type 5 Informative message, check

---

# Datablock: 15bH

---

Bond precision: C-C = 0.0071 A     Wavelength=1.54184

| | | | |
|---|---|---|---|
| Cell: | a=22.711(3) | b=14.166(3) | c=8.134(2) |
| | alpha=90 | beta=90 | gamma=90 |
| Temperature: | 100 K | | |

| | Calculated | Reported |
|---|---|---|
| Volume | 2616.9(9) | 2616.9(9) |
| Space group | P c a 21 | P c a 21 |
| Hall group | P 2c -2ac | P 2c -2ac |
| Moiety formula | C27 H27 F5 N4 O3 | C27 H27 F5 N4 O3 |
| Sum formula | C27 H27 F5 N4 O3 | C27 H27 F5 N4 O3 |
| Mr | 550.53 | 550.52 |
| Dx,g cm-3 | 1.397 | 1.397 |
| Z | 4 | 4 |
| Mu (mm-1) | 1.000 | 0.999 |
| F000 | 1144.0 | 1144.0 |
| F000' | 1148.30 | |
| h,k,lmax | 28,17,10 | 28,17,9 |
| Nref | 5315[ 2859] | 4232 |
| Tmin,Tmax | 0.976,0.990 | 0.139,1.000 |
| Tmin' | 0.698 | |

Correction method= # Reported T Limits: Tmin=0.139 Tmax=1.000
AbsCorr = MULTI-SCAN

Data completeness= 1.48/0.80 Theta(max)= 74.013

R(reflections)= 0.0581( 3197) wR2(reflections)= 0.1507( 4232)

S = 0.997 Npar= 371

---

The following ALERTS were generated. Each ALERT has the format
**test-name_ALERT_alert-type_alert-level.**
Click on the hyperlinks for more details of the test.

---

### Alert level C

```
STRVA01_ALERT_4_C           Flack parameter is too small
           From the CIF: _refine_ls_abs_structure_Flack   -0.300
           From the CIF: _refine_ls_abs_structure_Flack_su    0.200
PLAT340_ALERT_3_C Low Bond Precision on  C-C Bonds ...............    0.00714 Ang.
```

---

### Alert level G

```
PLAT003_ALERT_2_G Number of Uiso or Uij Restrained non-H Atoms ...          4 Report
PLAT007_ALERT_5_G Number of Unrefined Donor-H Atoms ..............          1 Report
PLAT178_ALERT_4_G The CIF-Embedded .res File Contains SIMU Records          1 Report
PLAT301_ALERT_3_G Main Residue  Disorder ..............(Resd  1  )         5% Note
PLAT398_ALERT_2_G Deviating  C-O-C   Angle From 120 for O21      .      102.8 Degree
PLAT410_ALERT_2_G Short Intra H...H Contact  H16B      ..H17D     .       2.14 Ang.
                                                   x,y,z  =      1_555 Check
PLAT860_ALERT_3_G Number of Least-Squares Restraints .............         13 Note
PLAT883_ALERT_1_G No Info/Value for _atom_sites_solution_primary .     Please Do !
PLAT910_ALERT_3_G Missing # of FCF Reflection(s) Below Theta(Min).          1 Note
```

```
PLAT912_ALERT_4_G Missing # of FCF Reflections Above STh/L=  0.600         63 Note
PLAT915_ALERT_3_G No Flack x Check Done: Low Friedel Pair Coverage         59 %
PLAT941_ALERT_3_G Average HKL Measurement Multiplicity ..........         3.9 Low
PLAT978_ALERT_2_G Number C-C Bonds with Positive Residual Density.          1 Info
```

---

```
   0 ALERT level A = Most likely a serious problem - resolve or explain
   0 ALERT level B = A potentially serious problem, consider carefully
   2 ALERT level C = Check. Ensure it is not caused by an omission or oversight
  13 ALERT level G = General information/check it is not something unexpected

   1 ALERT type 1 CIF construction/syntax error, inconsistent or missing data
   4 ALERT type 2 Indicator that the structure model may be wrong or deficient
   6 ALERT type 3 Indicator that the structure quality may be low
   3 ALERT type 4 Improvement, methodology, query or suggestion
   1 ALERT type 5 Informative message, check
```

---

# Datablock: 20aPb

---

Bond precision: C-C = 0.0134 A Wavelength=1.54184

| | | | |
|---|---|---|---|
| Cell: | a=19.442(2) | b=13.794(2) | c=19.055(2) |
| | alpha=90 | beta=113.33(3) | gamma=90 |
| Temperature: | 100 K | | |

| | Calculated | Reported |
|---|---|---|
| Volume | 4692.4(14) | 4692.4(14) |
| Space group | P 21/c | P 21/c |
| Hall group | -P 2ybc | -P 2ybc |
| Moiety formula | C25 H26 N4 O4 Pb | C25 H26 N4 O4 Pb |
| Sum formula | C25 H26 N4 O4 Pb | C25 H26 N4 O4 Pb |
| Mr | 653.70 | 653.69 |
| Dx,g cm-3 | 1.851 | 1.851 |
| Z | 8 | 8 |
| Mu (mm-1) | 14.316 | 14.316 |
| F000 | 2544.0 | 2544.0 |
| F000' | 2517.83 | |
| h,k,lmax | 24,17,23 | 24,17,23 |
| Nref | 9447 | 17047 |
| Tmin,Tmax | 0.291,0.651 | 0.275,1.000 |
| Tmin' | 0.066 | |

Correction method= # Reported T Limits: Tmin=0.275 Tmax=1.000
AbsCorr = MULTI-SCAN

Data completeness= 1.804 Theta(max)= 73.420

R(reflections)= 0.0509( 14374) wR2(reflections)= 0.1457( 17047)

S = 1.073 Npar= 614

---

The following ALERTS were generated. Each ALERT has the format
**test-name_ALERT_alert-type_alert-level.**
Click on the hyperlinks for more details of the test.

---

**Alert level C**

```
PLAT241_ALERT_2_C High   'MainMol' Ueq as Compared to Neighbors of        O2 Check
PLAT342_ALERT_3_C Low Bond Precision on  C-C Bonds ...............    0.01338 Ang.
PLAT790_ALERT_4_C Centre of Gravity not Within Unit Cell: Resd.  #          1 Note
              C25 H26 N4 O4 Pb
PLAT906_ALERT_3_C Large K Value in the Analysis of Variance ......      5.184 Check
PLAT911_ALERT_3_C Missing FCF Refl Between Thmin & STh/L=    0.600         21 Report
PLAT918_ALERT_3_C Reflection(s) with I(obs) much Smaller I(calc) .         17 Check
PLAT939_ALERT_3_C Large Value of Not (SHELXL) Weight Optimized S .      24.31 Check
```

---

**Alert level G**

```
PLAT083_ALERT_2_G SHELXL Second Parameter in WGHT  Unusually Large      41.28 Why ?
PLAT380_ALERT_4_G Incorrectly? Oriented X(sp2)-Methyl Moiety .....       C31A Check
PLAT720_ALERT_4_G Number of Unusual/Non-Standard Labels ..........          4 Note
PLAT790_ALERT_4_G Centre of Gravity not Within Unit Cell: Resd.  #          2 Note
              C25 H26 N4 O4 Pb
PLAT794_ALERT_5_G Tentative Bond Valency for Pb1       (II)      .       2.22 Info
PLAT794_ALERT_5_G Tentative Bond Valency for Pb2       (II)      .       2.12 Info
PLAT870_ALERT_4_G ALERTS Related to Twinning Effects Suppressed ..          ! Info
PLAT883_ALERT_1_G No Info/Value for _atom_sites_solution_primary .     Please Do !
PLAT912_ALERT_4_G Missing # of FCF Reflections Above STh/L=  0.600        294 Note
PLAT941_ALERT_3_G Average HKL Measurement Multiplicity ..........         1.9 Low
PLAT965_ALERT_2_G The SHELXL WEIGHT Optimisation has not Converged     Please Check
```

---

0 **ALERT level A** = Most likely a serious problem - resolve or explain
0 **ALERT level B** = A potentially serious problem, consider carefully
7 **ALERT level C** = Check. Ensure it is not caused by an omission or oversight
11 **ALERT level G** = General information/check it is not something unexpected

1 ALERT type 1 CIF construction/syntax error, inconsistent or missing data
3 ALERT type 2 Indicator that the structure model may be wrong or deficient
6 ALERT type 3 Indicator that the structure quality may be low
6 ALERT type 4 Improvement, methodology, query or suggestion
2 ALERT type 5 Informative message, check

---

# Datablock: 22aZn

---

Bond precision: C-C = 0.0021 A Wavelength=1.54184

| | | | |
|---|---|---|---|
| Cell: | a=8.507(2) | b=17.186(3) | c=17.472(3) |
| | alpha=100.29(2) | beta=96.09(2) | gamma=102.48(3) |
| Temperature: | 100 K | | |

| | Calculated | Reported |
|---|---|---|
| Volume | 2426.0(9) | 2426.0(9) |
| Space group | P -1 | P -1 |
| Hall group | -P 1 | -P 1 |
| Moiety formula | C54 H62 N8 O6 Zn | C54 H62 N8 O6 Zn |
| Sum formula | C54 H62 N8 O6 Zn | C54 H62 N8 O6 Zn |
| Mr | 984.51 | 984.48 |
| Dx,g cm-3 | 1.348 | 1.348 |
| Z | 2 | 2 |
| Mu (mm-1) | 1.187 | 1.187 |
| F000 | 1040.0 | 1040.0 |
| F000' | 1039.73 | |
| h,k,lmax | 10,21,21 | 10,21,21 |
| Nref | 10117 | 9909 |
| Tmin,Tmax | 0.843,0.965 | 0.888,1.000 |
| Tmin' | 0.770 | |

Correction method= # Reported T Limits: Tmin=0.888 Tmax=1.000
AbsCorr = MULTI-SCAN

Data completeness= 0.979 Theta(max)= 75.835

R(reflections)= 0.0338( 8945) wR2(reflections)= 0.0912( 9909)

S = 1.081 Npar= 650

---

The following ALERTS were generated. Each ALERT has the format
**test-name_ALERT_alert-type_alert-level.**
Click on the hyperlinks for more details of the test.

---

### Alert level C

```
PLAT220_ALERT_2_C NonSolvent   Resd 1  C   Ueq(max)/Ueq(min) Range        3.2 Ratio
PLAT911_ALERT_3_C Missing FCF Refl Between Thmin & STh/L=    0.600          7 Report
```

---

### Alert level G

```
PLAT003_ALERT_2_G Number of Uiso or Uij Restrained non-H Atoms ...          4 Report
PLAT178_ALERT_4_G The CIF-Embedded .res File Contains SIMU Records          1 Report
PLAT230_ALERT_2_G Hirshfeld Test Diff for    O21      --C22       .       6.9 s.u.
PLAT301_ALERT_3_G Main Residue  Disorder .............(Resd  1  )          4% Note
PLAT398_ALERT_2_G Deviating  C-O-C   Angle From 120 for O21A      .     108.1 Degree
PLAT410_ALERT_2_G Short Intra H...H Contact  H16B     ..H19D      .      1.86 Ang.
```

```
                                                     x,y,z  =      1_555 Check
PLAT410_ALERT_2_G Short Intra H...H Contact  H17B     ..H19C     .       1.77 Ang.
                                                     x,y,z  =      1_555 Check
PLAT410_ALERT_2_G Short Intra H...H Contact  H17B     ..H19D     .       2.01 Ang.
                                                     x,y,z  =      1_555 Check
PLAT794_ALERT_5_G Tentative Bond Valency for Zn1       (II)      .       1.75 Info
PLAT860_ALERT_3_G Number of Least-Squares Restraints ............          12 Note
PLAT883_ALERT_1_G No Info/Value for _atom_sites_solution_primary .     Please Do !
PLAT912_ALERT_4_G Missing # of FCF Reflections Above STh/L=  0.600        201 Note
PLAT941_ALERT_3_G Average HKL Measurement Multiplicity ..........         4.2 Low
PLAT965_ALERT_2_G The SHELXL WEIGHT Optimisation has not Converged     Please Check
PLAT978_ALERT_2_G Number C-C Bonds with Positive Residual Density.         15 Info
```

---

0 **ALERT level A** = Most likely a serious problem - resolve or explain
0 **ALERT level B** = A potentially serious problem, consider carefully
2 **ALERT level C** = Check. Ensure it is not caused by an omission or oversight
15 **ALERT level G** = General information/check it is not something unexpected

1 ALERT type 1 CIF construction/syntax error, inconsistent or missing data
9 ALERT type 2 Indicator that the structure model may be wrong or deficient
4 ALERT type 3 Indicator that the structure quality may be low
2 ALERT type 4 Improvement, methodology, query or suggestion
1 ALERT type 5 Informative message, check

---

# Datablock: 22bZn

---

Bond precision: C-C = 0.0053 A Wavelength=1.54184

| | | | |
|---|---|---|---|
| Cell: | a=13.301(3) | b=15.701(3) | c=28.077(4) |
| | alpha=90 | beta=94.99(2) | gamma=90 |
| Temperature: | 100 K | | |

| | Calculated | Reported |
|---|---|---|
| Volume | 5841.4(19) | 5841.3(19) |
| Space group | P 21/c | P 21/c |
| Hall group | -P 2ybc | -P 2ybc |
| Moiety formula | C54 H52 F10 N8 O6 Zn [+ solvent] | C54 H52 F10 N8 O6 Zn [+ solvent] |
| Sum formula | C54 H52 F10 N8 O6 Zn [+ solvent] | C54 H52 F10 N8 O6 Zn [+ solvent] |
| Mr | 1164.43 | 1164.40 |
| Dx,g cm-3 | 1.324 | 1.324 |
| Z | 4 | 4 |
| Mu (mm-1) | 1.326 | 1.326 |
| F000 | 2400.0 | 2400.0 |
| F000' | 2402.35 | |
| h,k,lmax | 16,19,35 | 16,19,35 |
| Nref | 12199 | 12000 |
| Tmin,Tmax | 0.867,0.961 | 0.576,1.000 |
| Tmin' | 0.864 | |

Correction method= # Reported T Limits: Tmin=0.576 Tmax=1.000
AbsCorr = MULTI-SCAN

Data completeness= 0.984 Theta(max)= 76.077

R(reflections)= 0.0617( 7895) wR2(reflections)= 0.1912( 12000)

S = 1.058 Npar= 713

---

The following ALERTS were generated. Each ALERT has the format
**test-name_ALERT_alert-type_alert-level.**
Click on the hyperlinks for more details of the test.

---

### Alert level C

```
PLAT230_ALERT_2_C Hirshfeld Test Diff for    C19      --C20      .        5.2 s.u.
PLAT790_ALERT_4_C Centre of Gravity not Within Unit Cell: Resd.  #          1 Note
              C54 H52 F10 N8 O6 Zn
PLAT911_ALERT_3_C Missing FCF Refl Between Thmin & STh/L=    0.600          5 Report
PLAT934_ALERT_3_C Number of (Iobs-Icalc)/Sigma(W) > 10 Outliers ..          1 Check
```

---

### Alert level G

```
CELLZ01_ALERT_1_G Difference between formula and atom_site contents detected.
CELLZ01_ALERT_1_G ALERT: Large difference may be due to a
            symmetry error - see SYMMG tests
           From the CIF: _cell_formula_units_Z    4
           From the CIF: _chemical_formula_sum  C54 H52 F10 N8 O6 Zn [+ solvent]
           TEST: Compare cell contents of formula and atom_site data
```

```
          atom    Z*formula  cif sites diff
          C        216.00    216.00    0.00
          H        208.00    208.00    0.00
          F         40.00     40.00    0.00
          N         32.00     32.00    0.00
          O         24.00     24.00    0.00
          Zn         4.00      4.00    0.00
          [+         4.00      0.00    4.00
          solve      4.00      0.00    4.00
PLAT434_ALERT_2_G Short Inter HL..HL Contact F60      ..F60      .       2.80 Ang.
                                                 2-x,-y,2-z  =      3_757 Check
PLAT606_ALERT_4_G Solvent Accessible VOID(S) in Structure ........          ! Info
PLAT794_ALERT_5_G Tentative Bond Valency for Zn1       (II)      .       1.79 Info
PLAT869_ALERT_4_G ALERTS Related to the Use of SQUEEZE  Suppressed          ! Info
PLAT883_ALERT_1_G No Info/Value for _atom_sites_solution_primary .     Please Do !
PLAT912_ALERT_4_G Missing # of FCF Reflections Above STh/L=  0.600        194 Note
PLAT965_ALERT_2_G The SHELXL WEIGHT Optimisation has not Converged     Please Check
PLAT978_ALERT_2_G Number C-C Bonds with Positive Residual Density.          1 Info
```

---

0 **ALERT level A** = Most likely a serious problem - resolve or explain
0 **ALERT level B** = A potentially serious problem, consider carefully
4 **ALERT level C** = Check. Ensure it is not caused by an omission or oversight
10 **ALERT level G** = General information/check it is not something unexpected

3 ALERT type 1 CIF construction/syntax error, inconsistent or missing data
4 ALERT type 2 Indicator that the structure model may be wrong or deficient
2 ALERT type 3 Indicator that the structure quality may be low
4 ALERT type 4 Improvement, methodology, query or suggestion
1 ALERT type 5 Informative message, check

---

# Datablock: 22bHg

---

| | | | |
|---|---|---|---|
| Bond precision: | C-C = 0.0153 A | | Wavelength=1.54184 |
| Cell: | a=23.216(3) | b=8.308(2) | c=28.491(3) |
| | alpha=90 | beta=106.91(2) | gamma=90 |
| Temperature: | 100 K | | |

| | Calculated | Reported |
|---|---|---|
| Volume | 5257.7(16) | 5257.7(16) |
| Space group | P 21/c | P 21/c |
| Hall group | -P 2ybc | -P 2ybc |
| Moiety formula | C54 H52 F10 Hg N8 O6, O | C54 H54 F10 Hg N8 O7 |
| Sum formula | C54 H52 F10 Hg N8 O7 | C54 H54 F10 Hg N8 O7 |
| Mr | 1315.63 | 1317.64 |
| Dx,g cm-3 | 1.662 | 1.665 |
| Z | 4 | 4 |
| Mu (mm-1) | 6.100 | 6.100 |
| F000 | 2632.0 | 2640.0 |
| F000' | 2623.70 | |
| h,k,lmax | 28,10,35 | 28,10,35 |
| Nref | 10566 | 10246 |
| Tmin,Tmax | 0.572,0.918 | 0.633,1.000 |
| Tmin' | 0.442 | |

Correction method= # Reported T Limits: Tmin=0.633 Tmax=1.000
AbsCorr = MULTI-SCAN

Data completeness= 0.970 Theta(max)= 73.318

R(reflections)= 0.0696( 8013) wR2(reflections)= 0.2087( 10246)

S = 1.046 Npar= 967

---

The following ALERTS were generated. Each ALERT has the format
**test-name_ALERT_alert-type_alert-level.**
Click on the hyperlinks for more details of the test.

---

### Alert level B

```
PLAT919_ALERT_3_B Reflection # Likely Affected by the Beamstop ...          1 Check
```

---

### Alert level C

```
PLAT041_ALERT_1_C Calc. and Reported SumFormula    Strings  Differ     Please Check
PLAT042_ALERT_1_C Calc. and Reported MoietyFormula Strings  Differ     Please Check
PLAT043_ALERT_1_C Calculated and Reported Mol. Weight Differ by ..       2.01 Check
PLAT068_ALERT_1_C Reported F000 Differs from Calcd (or Missing)...     Please Check
PLAT234_ALERT_4_C Large Hirshfeld Difference C7       --C8       .       0.16 Ang.
PLAT234_ALERT_4_C Large Hirshfeld Difference C38      --C39      .       0.19 Ang.
PLAT234_ALERT_4_C Large Hirshfeld Difference C39      --C40      .       0.19 Ang.
PLAT234_ALERT_4_C Large Hirshfeld Difference C39      --C40A     .       0.20 Ang.
PLAT234_ALERT_4_C Large Hirshfeld Difference C60      --C61      .       0.18 Ang.
PLAT241_ALERT_2_C High   'MainMol' Ueq as Compared to Neighbors of        C28 Check
PLAT241_ALERT_2_C High   'MainMol' Ueq as Compared to Neighbors of        C38 Check
PLAT241_ALERT_2_C High   'MainMol' Ueq as Compared to Neighbors of        C50 Check
PLAT241_ALERT_2_C High   'MainMol' Ueq as Compared to Neighbors of        C60 Check
```

PLAT242_ALERT_2_C Low 'MainMol' Ueq as Compared to Neighbors of C49 Check
PLAT242_ALERT_2_C Low 'MainMol' Ueq as Compared to Neighbors of C58 Check
PLAT260_ALERT_2_C Large Average Ueq of Residue Including Hg1 0.102 Check
PLAT260_ALERT_2_C Large Average Ueq of Residue Including O70 0.136 Check
PLAT260_ALERT_2_C Large Average Ueq of Residue Including O70A 0.137 Check
PLAT342_ALERT_3_C Low Bond Precision on C-C Bonds ............... 0.01533 Ang.
PLAT430_ALERT_2_C Short Inter D...A Contact O70A ..N37 . 2.88 Ang.
x,y,z = 1_555 Check
PLAT906_ALERT_3_C Large K Value in the Analysis of Variance ...... 7.408 Check
PLAT911_ALERT_3_C Missing FCF Refl Between Thmin & STh/L= 0.600 24 Report
PLAT918_ALERT_3_C Reflection(s) with I(obs) much Smaller I(calc) . 2 Check
PLAT934_ALERT_3_C Number of (Iobs-Icalc)/Sigma(W) > 10 Outliers .. 1 Check
PLAT939_ALERT_3_C Large Value of Not (SHELXL) Weight Optimized S . 10.87 Check
PLAT971_ALERT_2_C Check Calcd Resid. Dens. 0.87Ang From Hg1 1.98 eA-3
PLAT971_ALERT_2_C Check Calcd Resid. Dens. 0.97Ang From Hg1 1.78 eA-3
PLAT971_ALERT_2_C Check Calcd Resid. Dens. 1.81Ang From O70A 1.67 eA-3
PLAT972_ALERT_2_C Check Calcd Resid. Dens. 0.77Ang From Hg1 -1.70 eA-3
PLAT977_ALERT_2_C Check Negative Difference Density on H19B . -0.64 eA-3

---

## Alert level G

FORMU01_ALERT_2_G There is a discrepancy between the atom counts in the
_chemical_formula_sum and the formula from the _atom_site* data.
Atom count from _chemical_formula_sum:C54 H54 F10 Hg1 N8 O7
Atom count from the _atom_site data: C54 H52 F10 Hg1 N8 O7
CELLZ01_ALERT_1_G Difference between formula and atom_site contents detected.
CELLZ01_ALERT_1_G WARNING: H atoms missing from atom site list. Is this intentional?
From the CIF: _cell_formula_units_Z 4
From the CIF: _chemical_formula_sum C54 H54 F10 Hg N8 O7
TEST: Compare cell contents of formula and atom_site data

| atom | Z*formula | cif sites | diff |
|---|---|---|---|
| C | 216.00 | 216.00 | 0.00 |
| H | 216.00 | 208.00 | 8.00 |
| F | 40.00 | 40.00 | 0.00 |
| Hg | 4.00 | 4.00 | 0.00 |
| N | 32.00 | 32.00 | 0.00 |
| O | 28.00 | 28.00 | 0.00 |

PLAT002_ALERT_2_G Number of Distance or Angle Restraints on AtSite 19 Note
PLAT003_ALERT_2_G Number of Uiso or Uij Restrained non-H Atoms ... 54 Report
PLAT083_ALERT_2_G SHELXL Second Parameter in WGHT Unusually Large 15.58 Why ?
PLAT176_ALERT_4_G The CIF-Embedded .res File Contains SADI Records 5 Report
PLAT178_ALERT_4_G The CIF-Embedded .res File Contains SIMU Records 3 Report
PLAT186_ALERT_4_G The CIF-Embedded .res File Contains ISOR Records 2 Report
PLAT230_ALERT_2_G Hirshfeld Test Diff for F61A --C61A . 5.4 s.u.
PLAT230_ALERT_2_G Hirshfeld Test Diff for C57 --C62A . 8.8 s.u.
PLAT230_ALERT_2_G Hirshfeld Test Diff for C57 --C62 . 5.5 s.u.
PLAT232_ALERT_2_G Hirshfeld Test Diff (M-X) Hg1 --N25A . 21.5 s.u.
PLAT300_ALERT_4_G Atom Site Occupancy of O15 Constrained at 0.5 Check
PLAT300_ALERT_4_G Atom Site Occupancy of O15A Constrained at 0.5 Check
PLAT300_ALERT_4_G Atom Site Occupancy of O18 Constrained at 0.5 Check
PLAT300_ALERT_4_G Atom Site Occupancy of O18A Constrained at 0.5 Check
PLAT300_ALERT_4_G Atom Site Occupancy of O21 Constrained at 0.5 Check
PLAT300_ALERT_4_G Atom Site Occupancy of O21A Constrained at 0.5 Check
PLAT300_ALERT_4_G Atom Site Occupancy of O41 Constrained at 0.5 Check
PLAT300_ALERT_4_G Atom Site Occupancy of O41A Constrained at 0.5 Check
PLAT300_ALERT_4_G Atom Site Occupancy of O44 Constrained at 0.5 Check

```
PLAT300_ALERT_4_G Atom Site Occupancy of O44A       Constrained at        0.5 Check
PLAT300_ALERT_4_G Atom Site Occupancy of O47        Constrained at        0.5 Check
PLAT300_ALERT_4_G Atom Site Occupancy of O47A       Constrained at        0.5 Check
PLAT300_ALERT_4_G Atom Site Occupancy of N25        Constrained at        0.5 Check
PLAT300_ALERT_4_G Atom Site Occupancy of N25A       Constrained at        0.5 Check
PLAT300_ALERT_4_G Atom Site Occupancy of C14        Constrained at        0.5 Check
PLAT300_ALERT_4_G Atom Site Occupancy of C14A       Constrained at        0.5 Check
PLAT300_ALERT_4_G Atom Site Occupancy of C16        Constrained at        0.5 Check
PLAT300_ALERT_4_G Atom Site Occupancy of C16A       Constrained at        0.5 Check
PLAT300_ALERT_4_G Atom Site Occupancy of C17        Constrained at        0.5 Check
PLAT300_ALERT_4_G Atom Site Occupancy of C17A       Constrained at        0.5 Check
PLAT300_ALERT_4_G Atom Site Occupancy of C19        Constrained at        0.5 Check
PLAT300_ALERT_4_G Atom Site Occupancy of C19A       Constrained at        0.5 Check
PLAT300_ALERT_4_G Atom Site Occupancy of C20        Constrained at        0.5 Check
PLAT300_ALERT_4_G Atom Site Occupancy of C20A       Constrained at        0.5 Check
PLAT300_ALERT_4_G Atom Site Occupancy of C22        Constrained at        0.5 Check
PLAT300_ALERT_4_G Atom Site Occupancy of C22A       Constrained at        0.5 Check
PLAT300_ALERT_4_G Atom Site Occupancy of C23        Constrained at        0.5 Check
PLAT300_ALERT_4_G Atom Site Occupancy of C23A       Constrained at        0.5 Check
PLAT300_ALERT_4_G Atom Site Occupancy of C24        Constrained at        0.5 Check
PLAT300_ALERT_4_G Atom Site Occupancy of C24A       Constrained at        0.5 Check
PLAT300_ALERT_4_G Atom Site Occupancy of C26        Constrained at        0.5 Check
PLAT300_ALERT_4_G Atom Site Occupancy of C26A       Constrained at        0.5 Check
PLAT300_ALERT_4_G Atom Site Occupancy of C40        Constrained at        0.5 Check
PLAT300_ALERT_4_G Atom Site Occupancy of C40A       Constrained at        0.5 Check
PLAT300_ALERT_4_G Atom Site Occupancy of C42        Constrained at        0.5 Check
PLAT300_ALERT_4_G Atom Site Occupancy of C42A       Constrained at        0.5 Check
PLAT300_ALERT_4_G Atom Site Occupancy of C43        Constrained at        0.5 Check
PLAT300_ALERT_4_G Atom Site Occupancy of C43A       Constrained at        0.5 Check
PLAT300_ALERT_4_G Atom Site Occupancy of C45        Constrained at        0.5 Check
PLAT300_ALERT_4_G Atom Site Occupancy of C45A       Constrained at        0.5 Check
PLAT300_ALERT_4_G Atom Site Occupancy of C46        Constrained at        0.5 Check
PLAT300_ALERT_4_G Atom Site Occupancy of C46A       Constrained at        0.5 Check
PLAT300_ALERT_4_G Atom Site Occupancy of C48        Constrained at        0.5 Check
PLAT300_ALERT_4_G Atom Site Occupancy of C48A       Constrained at        0.5 Check
PLAT300_ALERT_4_G Atom Site Occupancy of H14A       Constrained at        0.5 Check
PLAT300_ALERT_4_G Atom Site Occupancy of H14B       Constrained at        0.5 Check
PLAT300_ALERT_4_G Atom Site Occupancy of H14C       Constrained at        0.5 Check
PLAT300_ALERT_4_G Atom Site Occupancy of H14D       Constrained at        0.5 Check
PLAT300_ALERT_4_G Atom Site Occupancy of H16A       Constrained at        0.5 Check
PLAT300_ALERT_4_G Atom Site Occupancy of H16B       Constrained at        0.5 Check
PLAT300_ALERT_4_G Atom Site Occupancy of H16C       Constrained at        0.5 Check
PLAT300_ALERT_4_G Atom Site Occupancy of H16D       Constrained at        0.5 Check
PLAT300_ALERT_4_G Atom Site Occupancy of H17A       Constrained at        0.5 Check
PLAT300_ALERT_4_G Atom Site Occupancy of H17B       Constrained at        0.5 Check
PLAT300_ALERT_4_G Atom Site Occupancy of H17C       Constrained at        0.5 Check
PLAT300_ALERT_4_G Atom Site Occupancy of H17D       Constrained at        0.5 Check
PLAT300_ALERT_4_G Atom Site Occupancy of H19A       Constrained at        0.5 Check
PLAT300_ALERT_4_G Atom Site Occupancy of H19B       Constrained at        0.5 Check
PLAT300_ALERT_4_G Atom Site Occupancy of H19C       Constrained at        0.5 Check
PLAT300_ALERT_4_G Atom Site Occupancy of H19D       Constrained at        0.5 Check
PLAT300_ALERT_4_G Atom Site Occupancy of H20A       Constrained at        0.5 Check
PLAT300_ALERT_4_G Atom Site Occupancy of H20B       Constrained at        0.5 Check
PLAT300_ALERT_4_G Atom Site Occupancy of H20C       Constrained at        0.5 Check
PLAT300_ALERT_4_G Atom Site Occupancy of H20D       Constrained at        0.5 Check
PLAT300_ALERT_4_G Atom Site Occupancy of H22A       Constrained at        0.5 Check
PLAT300_ALERT_4_G Atom Site Occupancy of H22B       Constrained at        0.5 Check
```

```
PLAT300_ALERT_4_G Atom Site Occupancy of H22C       Constrained at        0.5 Check
PLAT300_ALERT_4_G Atom Site Occupancy of H22D       Constrained at        0.5 Check
PLAT300_ALERT_4_G Atom Site Occupancy of H23A       Constrained at        0.5 Check
PLAT300_ALERT_4_G Atom Site Occupancy of H23B       Constrained at        0.5 Check
PLAT300_ALERT_4_G Atom Site Occupancy of H23C       Constrained at        0.5 Check
PLAT300_ALERT_4_G Atom Site Occupancy of H23D       Constrained at        0.5 Check
PLAT300_ALERT_4_G Atom Site Occupancy of H24A       Constrained at        0.5 Check
PLAT300_ALERT_4_G Atom Site Occupancy of H24B       Constrained at        0.5 Check
PLAT300_ALERT_4_G Atom Site Occupancy of H24C       Constrained at        0.5 Check
PLAT300_ALERT_4_G Atom Site Occupancy of H24D       Constrained at        0.5 Check
PLAT300_ALERT_4_G Atom Site Occupancy of H26A       Constrained at        0.5 Check
PLAT300_ALERT_4_G Atom Site Occupancy of H26B       Constrained at        0.5 Check
PLAT300_ALERT_4_G Atom Site Occupancy of H40A       Constrained at        0.5 Check
PLAT300_ALERT_4_G Atom Site Occupancy of H40B       Constrained at        0.5 Check
PLAT300_ALERT_4_G Atom Site Occupancy of H40C       Constrained at        0.5 Check
PLAT300_ALERT_4_G Atom Site Occupancy of H40D       Constrained at        0.5 Check
PLAT300_ALERT_4_G Atom Site Occupancy of H42A       Constrained at        0.5 Check
PLAT300_ALERT_4_G Atom Site Occupancy of H42B       Constrained at        0.5 Check
PLAT300_ALERT_4_G Atom Site Occupancy of H42C       Constrained at        0.5 Check
PLAT300_ALERT_4_G Atom Site Occupancy of H42D       Constrained at        0.5 Check
PLAT300_ALERT_4_G Atom Site Occupancy of H43A       Constrained at        0.5 Check
PLAT300_ALERT_4_G Atom Site Occupancy of H43B       Constrained at        0.5 Check
PLAT300_ALERT_4_G Atom Site Occupancy of H43C       Constrained at        0.5 Check
PLAT300_ALERT_4_G Atom Site Occupancy of H43D       Constrained at        0.5 Check
PLAT300_ALERT_4_G Atom Site Occupancy of H45A       Constrained at        0.5 Check
PLAT300_ALERT_4_G Atom Site Occupancy of H45B       Constrained at        0.5 Check
PLAT300_ALERT_4_G Atom Site Occupancy of H45C       Constrained at        0.5 Check
PLAT300_ALERT_4_G Atom Site Occupancy of H45D       Constrained at        0.5 Check
PLAT300_ALERT_4_G Atom Site Occupancy of H46A       Constrained at        0.5 Check
PLAT300_ALERT_4_G Atom Site Occupancy of H46B       Constrained at        0.5 Check
PLAT300_ALERT_4_G Atom Site Occupancy of H46C       Constrained at        0.5 Check
PLAT300_ALERT_4_G Atom Site Occupancy of H46D       Constrained at        0.5 Check
PLAT300_ALERT_4_G Atom Site Occupancy of H48A       Constrained at        0.5 Check
PLAT300_ALERT_4_G Atom Site Occupancy of H48B       Constrained at        0.5 Check
PLAT300_ALERT_4_G Atom Site Occupancy of H48C       Constrained at        0.5 Check
PLAT300_ALERT_4_G Atom Site Occupancy of H48D       Constrained at        0.5 Check
PLAT300_ALERT_4_G Atom Site Occupancy of O70        Constrained at        0.5 Check
PLAT300_ALERT_4_G Atom Site Occupancy of O70A       Constrained at        0.5 Check
PLAT301_ALERT_3_G Main Residue  Disorder .............(Resd  1  )        33% Note
PLAT302_ALERT_4_G Anion/Solvent/Minor-Residue Disorder (Resd  2  )       100% Note
PLAT302_ALERT_4_G Anion/Solvent/Minor-Residue Disorder (Resd  3  )       100% Note
PLAT304_ALERT_4_G Non-Integer Number of Atoms in ..... (Resd  2  )       0.50 Check
PLAT304_ALERT_4_G Non-Integer Number of Atoms in ..... (Resd  3  )       0.50 Check
PLAT311_ALERT_2_G Isolated Disordered Oxygen Atom (No H's ?) .....        O70 Check
PLAT311_ALERT_2_G Isolated Disordered Oxygen Atom (No H's ?) .....       O70A Check
PLAT367_ALERT_2_G Long?  C(sp?)-C(sp?) Bond  C38      - C39      .       1.58 Ang.
PLAT398_ALERT_2_G Deviating  C-O-C   Angle From 120 for O18A     .      136.4 Degree
PLAT410_ALERT_2_G Short Intra H...H Contact  H48A     ..H49B     .       2.13 Ang.
                                                     x,y,z  =      1_555 Check
PLAT410_ALERT_2_G Short Intra H...H Contact  H48B     ..H49B     .       2.07 Ang.
                                                     x,y,z  =      1_555 Check
PLAT410_ALERT_2_G Short Intra H...H Contact  H48C     ..H49A     .       2.05 Ang.
                                                     x,y,z  =      1_555 Check
PLAT410_ALERT_2_G Short Intra H...H Contact  H48C     ..H49B     .       2.05 Ang.
                                                     x,y,z  =      1_555 Check
PLAT410_ALERT_2_G Short Intra H...H Contact  H48D     ..H49B     .       2.07 Ang.
                                                     x,y,z  =      1_555 Check
```

```
PLAT411_ALERT_2_G Short Inter H...H Contact  H8A      ..H24B     .       1.75 Ang.
                                                 x,-1+y,z  =     1_545 Check
PLAT411_ALERT_2_G Short Inter H...H Contact  H8A      ..H24D     .       2.11 Ang.
                                                 x,-1+y,z  =     1_545 Check
PLAT431_ALERT_2_G Short Inter HL..A Contact  F60      ..O18A     .       2.58 Ang.
                                         2-x,-1/2+y,3/2-z  =     2_746 Check
PLAT432_ALERT_2_G Short Inter X...Y Contact  O18A     ..C60      .       2.68 Ang.
                                          2-x,1/2+y,3/2-z  =     2_756 Check
PLAT432_ALERT_2_G Short Inter X...Y Contact  F61      ..C60      .       2.79 Ang.
                                              2-x,1-y,1-z  =     3_766 Check
PLAT432_ALERT_2_G Short Inter X...Y Contact  C19A     ..C60      .       3.20 Ang.
                                          2-x,1/2+y,3/2-z  =     2_756 Check
PLAT432_ALERT_2_G Short Inter X...Y Contact  C60      ..C61      .       3.16 Ang.
                                              2-x,1-y,1-z  =     3_766 Check
PLAT811_ALERT_5_G No ADDSYM Analysis: Too Many Excluded Atoms ....          ! Info
PLAT860_ALERT_3_G Number of Least-Squares Restraints ............        1032 Note
PLAT883_ALERT_1_G No Info/Value for _atom_sites_solution_primary .     Please Do !
PLAT912_ALERT_4_G Missing # of FCF Reflections Above STh/L=  0.600        297 Note
PLAT941_ALERT_3_G Average HKL Measurement Multiplicity ..........         3.8 Low
PLAT965_ALERT_2_G The SHELXL WEIGHT Optimisation has not Converged     Please Check
PLAT978_ALERT_2_G Number C-C Bonds with Positive Residual Density.          4 Info
```

---

  0 **ALERT level A** = Most likely a serious problem - resolve or explain
  1 **ALERT level B** = A potentially serious problem, consider carefully
 30 **ALERT level C** = Check. Ensure it is not caused by an omission or oversight
145 **ALERT level G** = General information/check it is not something unexpected

  7 ALERT type 1 CIF construction/syntax error, inconsistent or missing data
 41 ALERT type 2 Indicator that the structure model may be wrong or deficient
 10 ALERT type 3 Indicator that the structure quality may be low
117 ALERT type 4 Improvement, methodology, query or suggestion
  1 ALERT type 5 Informative message, check

---

# Datablock: 22bCd

---

Bond precision: C-C = 0.0176 A Wavelength=1.54184

| | | | |
|---|---|---|---|
| Cell: | a=23.482(3) | b=8.277(2) | c=28.339(3) |
| | alpha=90 | beta=106.77(2) | gamma=90 |
| Temperature: | 100 K | | |

| | Calculated | Reported |
|---|---|---|
| Volume | 5273.7(16) | 5273.7(16) |
| Space group | P 21/c | P 21/c |
| Hall group | -P 2ybc | -P 2ybc |
| Moiety formula | C54 H52 Cd F10 N8 O6 | C54 H52 Cd F10 N8 O6 |
| Sum formula | C54 H52 Cd F10 N8 O6 | C54 H52 Cd F10 N8 O6 |
| Mr | 1211.45 | 1211.43 |
| Dx,g cm-3 | 1.526 | 1.526 |
| Z | 4 | 4 |
| Mu (mm-1) | 4.140 | 4.140 |
| F000 | 2472.0 | 2472.0 |
| F000' | 2481.18 | |
| h,k,lmax | 29,10,35 | 29,10,35 |
| Nref | 10606 | 10124 |
| Tmin,Tmax | 0.756,0.980 | 0.791,1.000 |
| Tmin' | 0.601 | |

Correction method= # Reported T Limits: Tmin=0.791 Tmax=1.000
AbsCorr = MULTI-SCAN

Data completeness= 0.955 Theta(max)= 73.374

R(reflections)= 0.1015( 5483) wR2(reflections)= 0.3487( 10124)

S = 1.202 Npar= 1037

---

The following ALERTS were generated. Each ALERT has the format
**test-name_ALERT_alert-type_alert-level.**
Click on the hyperlinks for more details of the test.

---

**Alert level B**

PLAT230_ALERT_2_B Hirshfeld Test Diff for F68 --C68 . 8.4 s.u.

---

**Alert level C**

PLAT084_ALERT_3_C High wR2 Value (i.e. > 0.25) .................. 0.35 Report
PLAT088_ALERT_3_C Poor Data / Parameter Ratio .................... 9.76 Note
PLAT234_ALERT_4_C Large Hirshfeld Difference F65 --C65 . 0.19 Ang.
PLAT234_ALERT_4_C Large Hirshfeld Difference O15 --C16 . 0.16 Ang.
PLAT234_ALERT_4_C Large Hirshfeld Difference O15 --C16A . 0.16 Ang.
PLAT234_ALERT_4_C Large Hirshfeld Difference C6 --C5A . 0.18 Ang.
PLAT234_ALERT_4_C Large Hirshfeld Difference C7 --C8 . 0.19 Ang.
PLAT234_ALERT_4_C Large Hirshfeld Difference C9 --C10 . 0.19 Ang.
PLAT234_ALERT_4_C Large Hirshfeld Difference C13 --C14 . 0.18 Ang.
PLAT234_ALERT_4_C Large Hirshfeld Difference C23 --C24 . 0.21 Ang.
PLAT234_ALERT_4_C Large Hirshfeld Difference C31 --C32 . 0.17 Ang.
PLAT241_ALERT_2_C High 'MainMol' Ueq as Compared to Neighbors of O15 Check
PLAT241_ALERT_2_C High 'MainMol' Ueq as Compared to Neighbors of N37 Check

```
PLAT241_ALERT_2_C High   'MainMol' Ueq as Compared to Neighbors of        C34 Check
PLAT242_ALERT_2_C Low    'MainMol' Ueq as Compared to Neighbors of        C66 Check
PLAT260_ALERT_2_C Large Average Ueq of Residue Including       Cd1      0.124 Check
PLAT334_ALERT_2_C Small <C-C> Benzene Dist.   C63      -C68      .       1.37 Ang.
PLAT342_ALERT_3_C Low Bond Precision on  C-C Bonds ..............    0.01763 Ang.
PLAT906_ALERT_3_C Large K Value in the Analysis of Variance ......      2.938 Check
PLAT911_ALERT_3_C Missing FCF Refl Between Thmin & STh/L=    0.600         53 Report
PLAT934_ALERT_3_C Number of (Iobs-Icalc)/Sigma(W) > 10 Outliers ..          1 Check
PLAT977_ALERT_2_C Check Negative Difference Density on H42B      .      -0.34 eA-3
```

---

**Alert level G**

```
PLAT002_ALERT_2_G Number of Distance or Angle Restraints on AtSite         70 Note
PLAT003_ALERT_2_G Number of Uiso or Uij Restrained non-H Atoms ...         78 Report
PLAT174_ALERT_4_G The CIF-Embedded .res File Contains FLAT Records          1 Report
PLAT176_ALERT_4_G The CIF-Embedded .res File Contains SADI Records         19 Report
PLAT178_ALERT_4_G The CIF-Embedded .res File Contains SIMU Records          5 Report
PLAT186_ALERT_4_G The CIF-Embedded .res File Contains ISOR Records          1 Report
PLAT230_ALERT_2_G Hirshfeld Test Diff for    N51      --C50      .        7.5 s.u.
PLAT230_ALERT_2_G Hirshfeld Test Diff for    C5       --C6       .        7.0 s.u.
PLAT230_ALERT_2_G Hirshfeld Test Diff for    C5       --C57      .        6.0 s.u.
PLAT232_ALERT_2_G Hirshfeld Test Diff (M-X)  Cd1      --N53A     .        5.0 s.u.
PLAT301_ALERT_3_G Main Residue  Disorder ..............(Resd  1  )        49% Note
PLAT410_ALERT_2_G Short Intra H...H Contact  H14A     ..H16C     .       1.99 Ang.
                                                      x,y,z  =     1_555 Check
PLAT410_ALERT_2_G Short Intra H...H Contact  H23A     ..H22D     .       1.98 Ang.
                                                      x,y,z  =     1_555 Check
PLAT410_ALERT_2_G Short Intra H...H Contact  H36A     ..H38B     .       2.02 Ang.
                                                      x,y,z  =     1_555 Check
PLAT411_ALERT_2_G Short Inter H...H Contact  H34A     ..H50D     .       1.79 Ang.
                                                   x,-1+y,z  =     1_545 Check
PLAT432_ALERT_2_G Short Inter X...Y Contact  F66      ..C43A     .       2.96 Ang.
                                           2-x,-1/2+y,1/2-z  =     2_745 Check
PLAT432_ALERT_2_G Short Inter X...Y Contact  F68      ..C46      .       2.93 Ang.
                                            2-x,1/2+y,1/2-z  =     2_755 Check
PLAT432_ALERT_2_G Short Inter X...Y Contact  O15      ..C42A     .       2.96 Ang.
                                             x,1/2-y,-1/2+z  =     4_565 Check
PLAT432_ALERT_2_G Short Inter X...Y Contact  O44A     ..C66      .       2.99 Ang.
                                            2-x,1/2+y,1/2-z  =     2_755 Check
PLAT773_ALERT_2_G Check long C-C Bond in CIF: C50      --C52             1.98 Ang.
PLAT773_ALERT_2_G Check long C-C Bond in CIF: C4A      --C5A             2.03 Ang.
PLAT779_ALERT_4_G Suspect or Irrelevant (Bond) Angle(s) in CIF ...      33.60 Deg.
              N51  -C50  -C52     1_555   1_555   1_555 ......     #   98 Check
PLAT779_ALERT_4_G Suspect or Irrelevant (Bond) Angle(s) in CIF ...      42.80 Deg.
              N51  -C52  -C50     1_555   1_555   1_555 ......     #  128 Check
PLAT811_ALERT_5_G No ADDSYM Analysis: Too Many Excluded Atoms ....          ! Info
PLAT860_ALERT_3_G Number of Least-Squares Restraints ............        1743 Note
PLAT883_ALERT_1_G No Info/Value for _atom_sites_solution_primary .     Please Do !
PLAT910_ALERT_3_G Missing # of FCF Reflection(s) Below Theta(Min).          1 Note
PLAT912_ALERT_4_G Missing # of FCF Reflections Above STh/L=  0.600        426 Note
PLAT941_ALERT_3_G Average HKL Measurement Multiplicity ..........         3.4 Low
PLAT955_ALERT_1_G Reported (CIF) and Actual (FCF) Lmax Differ by .          1 Units
PLAT978_ALERT_2_G Number C-C Bonds with Positive Residual Density.          4 Info
```

---

0 **ALERT level A** = Most likely a serious problem - resolve or explain
1 **ALERT level B** = A potentially serious problem, consider carefully

```
22 ALERT level C = Check. Ensure it is not caused by an omission or oversight
31 ALERT level G = General information/check it is not something unexpected

 2 ALERT type 1 CIF construction/syntax error, inconsistent or missing data
25 ALERT type 2 Indicator that the structure model may be wrong or deficient
10 ALERT type 3 Indicator that the structure quality may be low
16 ALERT type 4 Improvement, methodology, query or suggestion
 1 ALERT type 5 Informative message, check
```

---

It is advisable to attempt to resolve as many as possible of the alerts in all categories. Often the minor alerts point to easily fixed oversights, errors and omissions in your CIF or refinement strategy, so attention to these fine details can be worthwhile. In order to resolve some of the more serious problems it may be necessary to carry out additional measurements or structure refinements. However, the purpose of your study may justify the reported deviations and the more serious of these should normally be commented upon in the discussion or experimental section of a paper or in the "special_details" fields of the CIF. checkCIF was carefully designed to identify outliers and unusual parameters, but every test has its limitations and alerts that are not important in a particular case may appear. Conversely, the absence of alerts does not guarantee there are no aspects of the results needing attention. It is up to the individual to critically assess their own results and, if necessary, seek expert advice.

**Publication of your CIF in IUCr journals**

A basic structural check has been run on your CIF. These basic checks will be run on all CIFs submitted for publication in IUCr journals (*Acta Crystallographica*, *Journal of Applied Crystallography*, *Journal of Synchrotron Radiation*); however, if you intend to submit to *Acta Crystallographica Section C* or *E* or *IUCrData*, you should make sure that full publication checks are run on the final version of your CIF prior to submission.

**Publication of your CIF in other journals**

Please refer to the *Notes for Authors* of the relevant journal for any special instructions relating to CIF submission.

---

**PLATON version of 18/05/2022; check.def file version of 17/05/2022**

**Datablock 14aH3** - ellipsoid plot

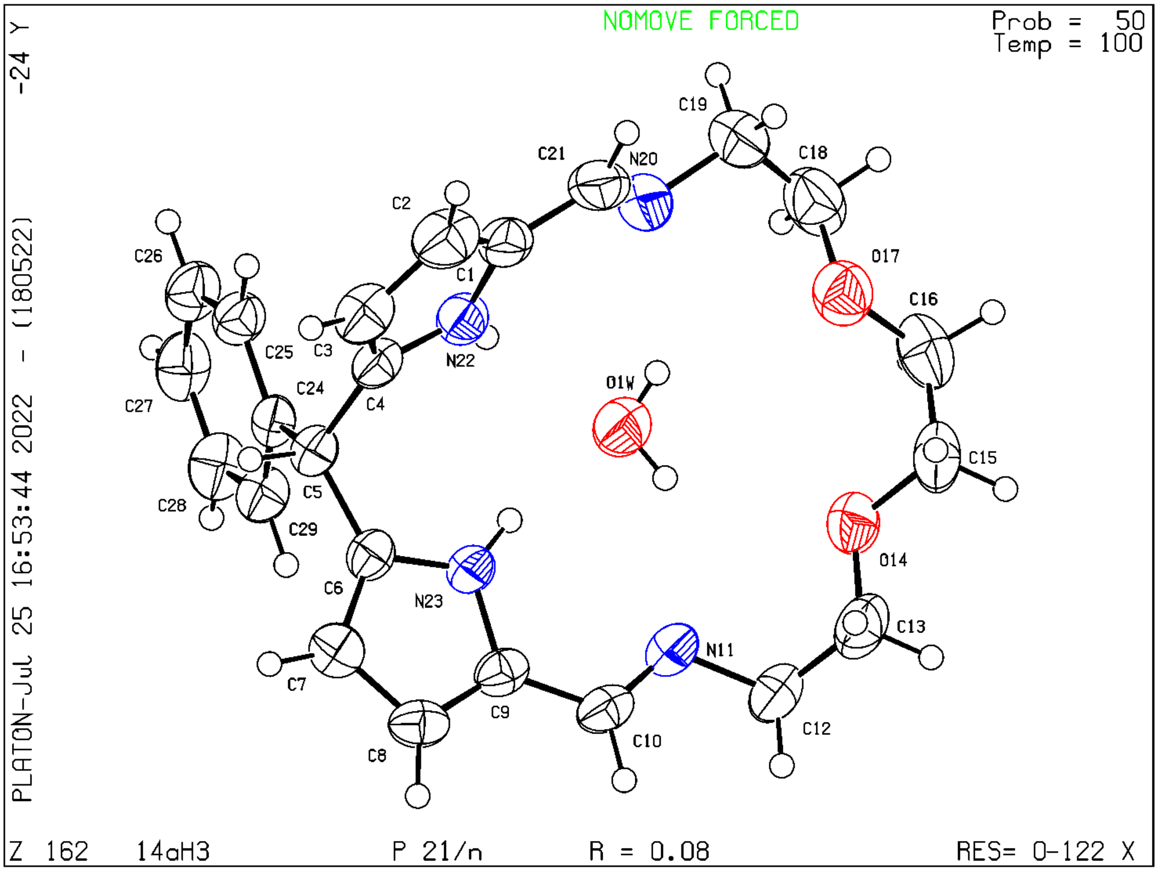

**Datablock 14aH7** - ellipsoid plot

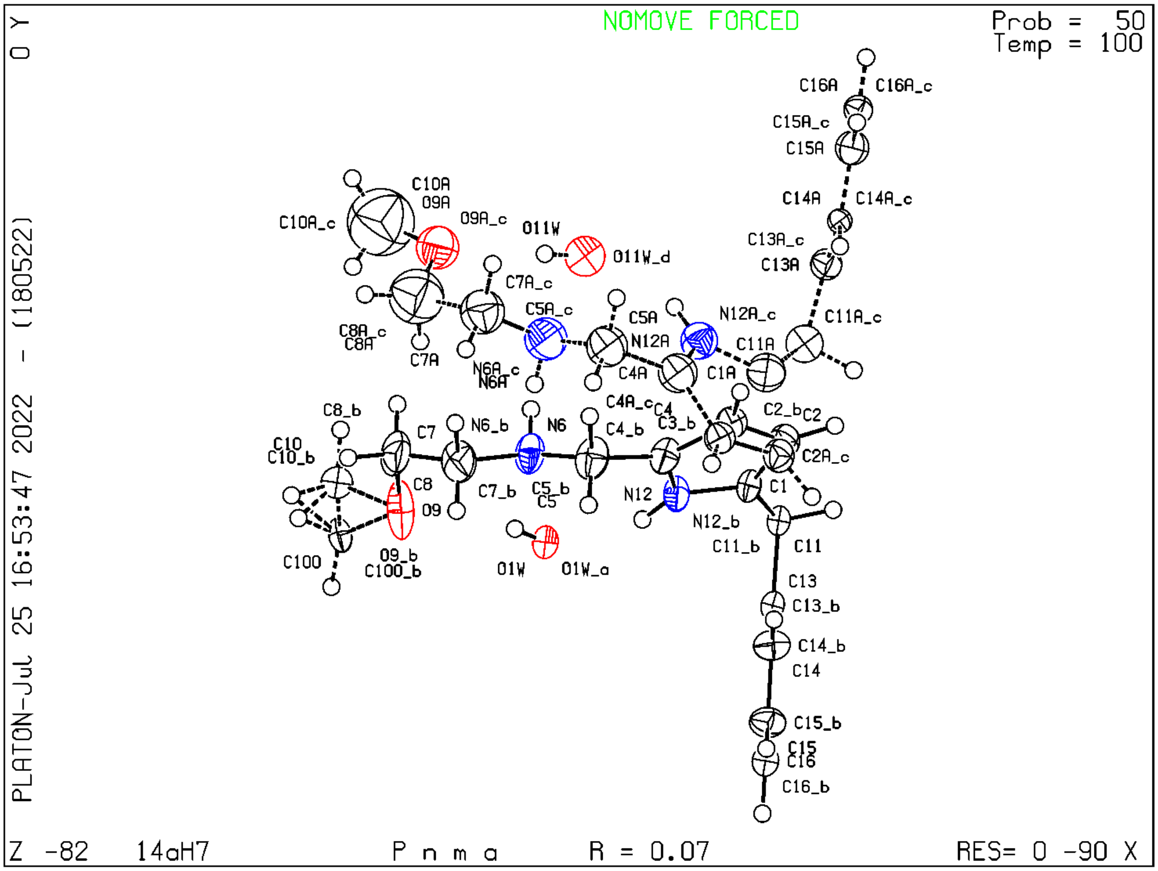

**Datablock 15aH72HCl** - ellipsoid plot

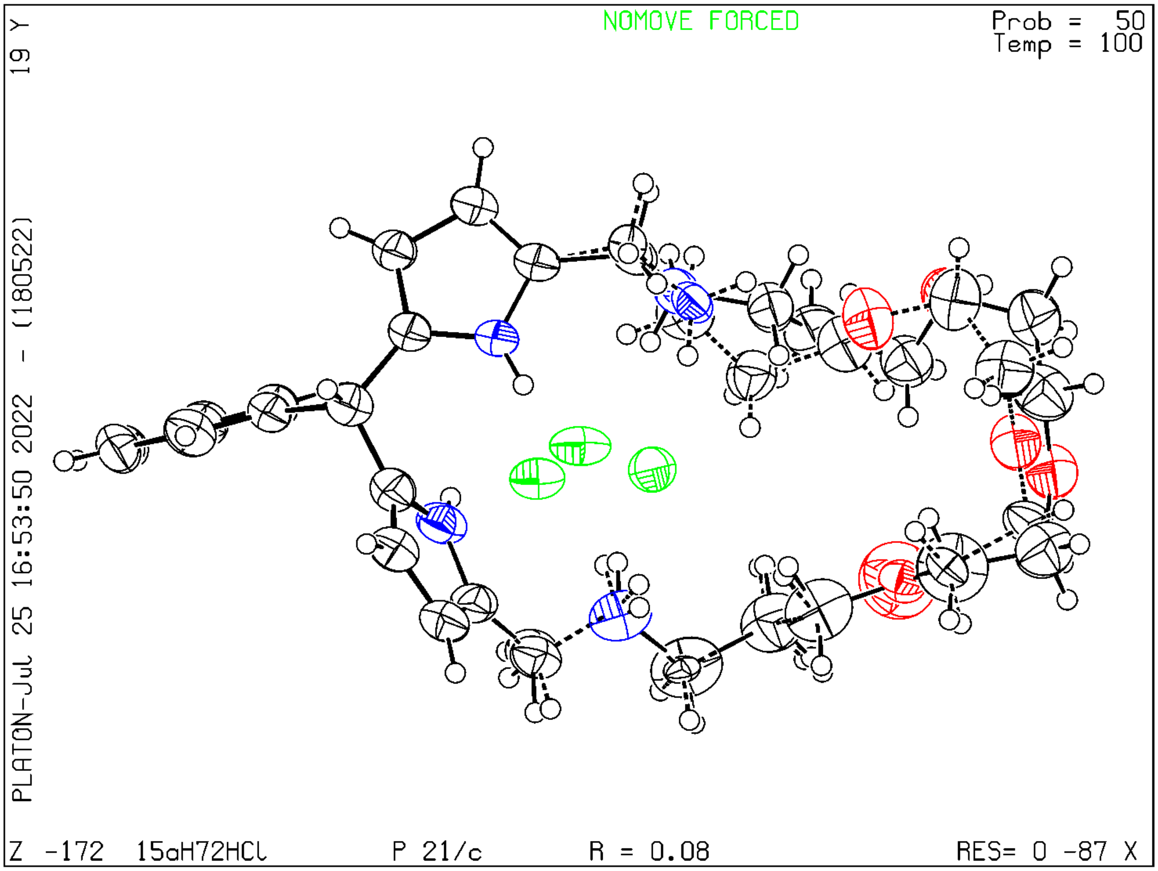

**Datablock 15bH** - ellipsoid plot

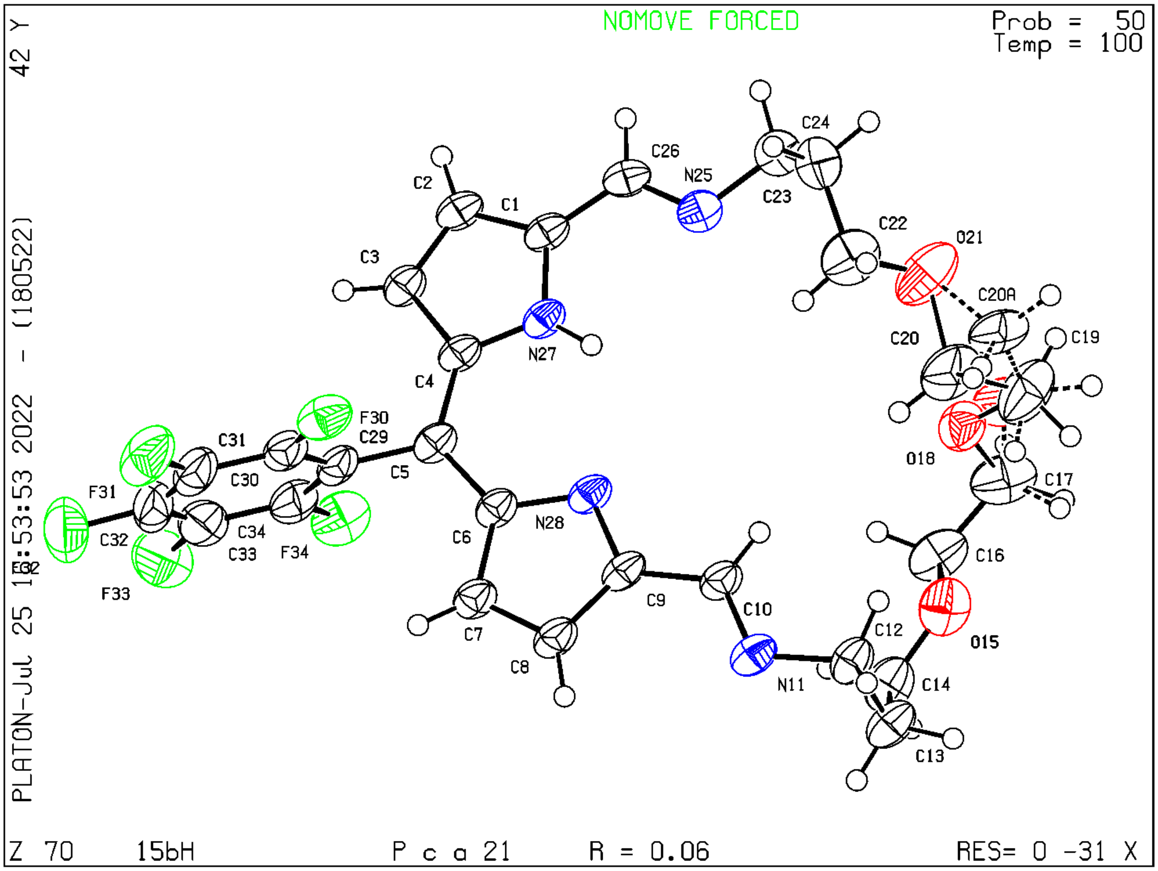

**Datablock 20aPb** - ellipsoid plot

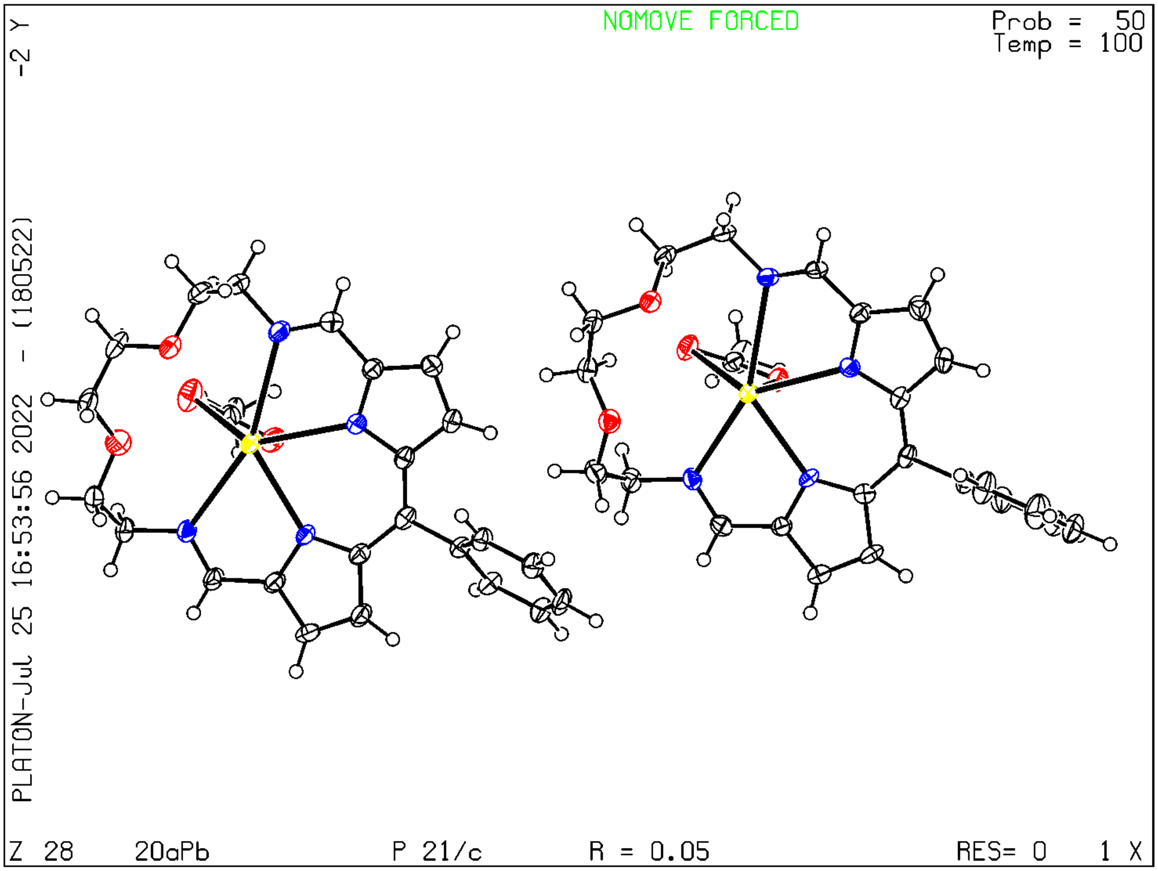

**Datablock 22aZn** - ellipsoid plot

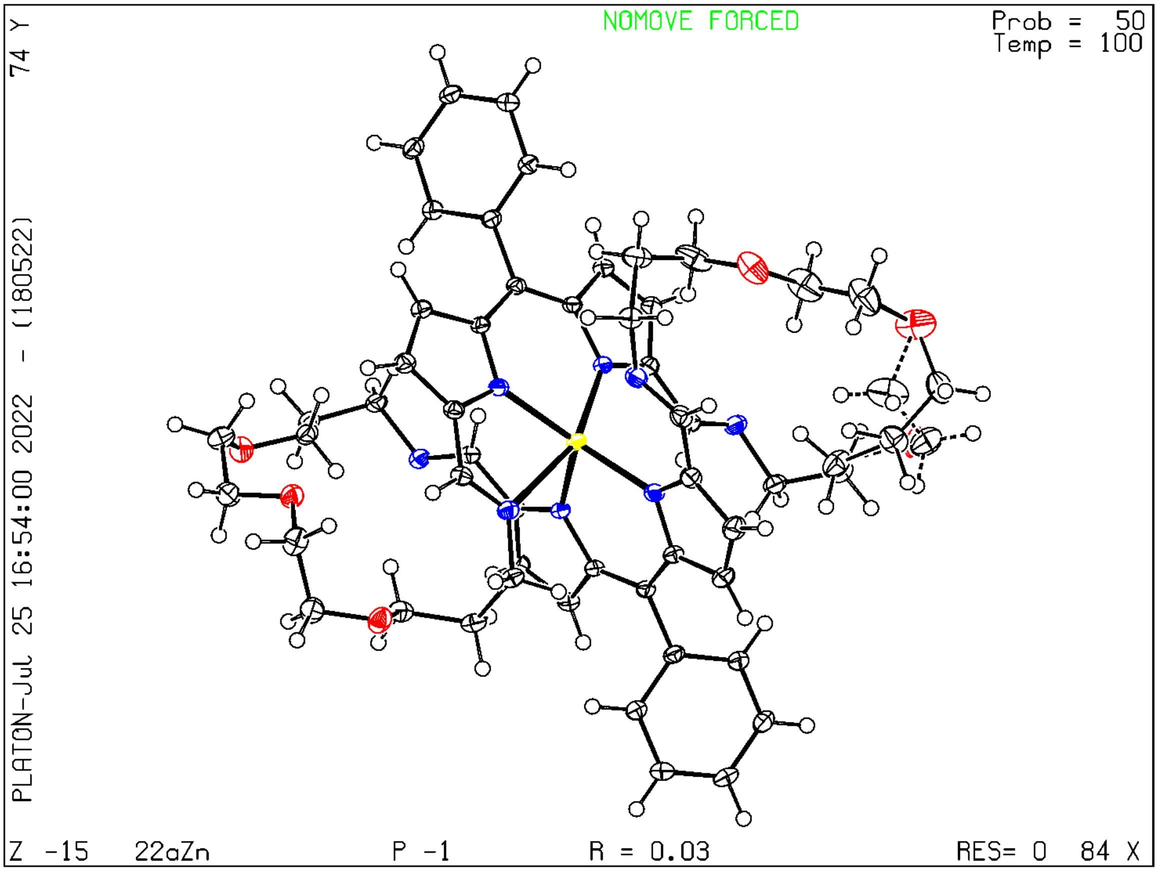

**Datablock 22bZn** - ellipsoid plot

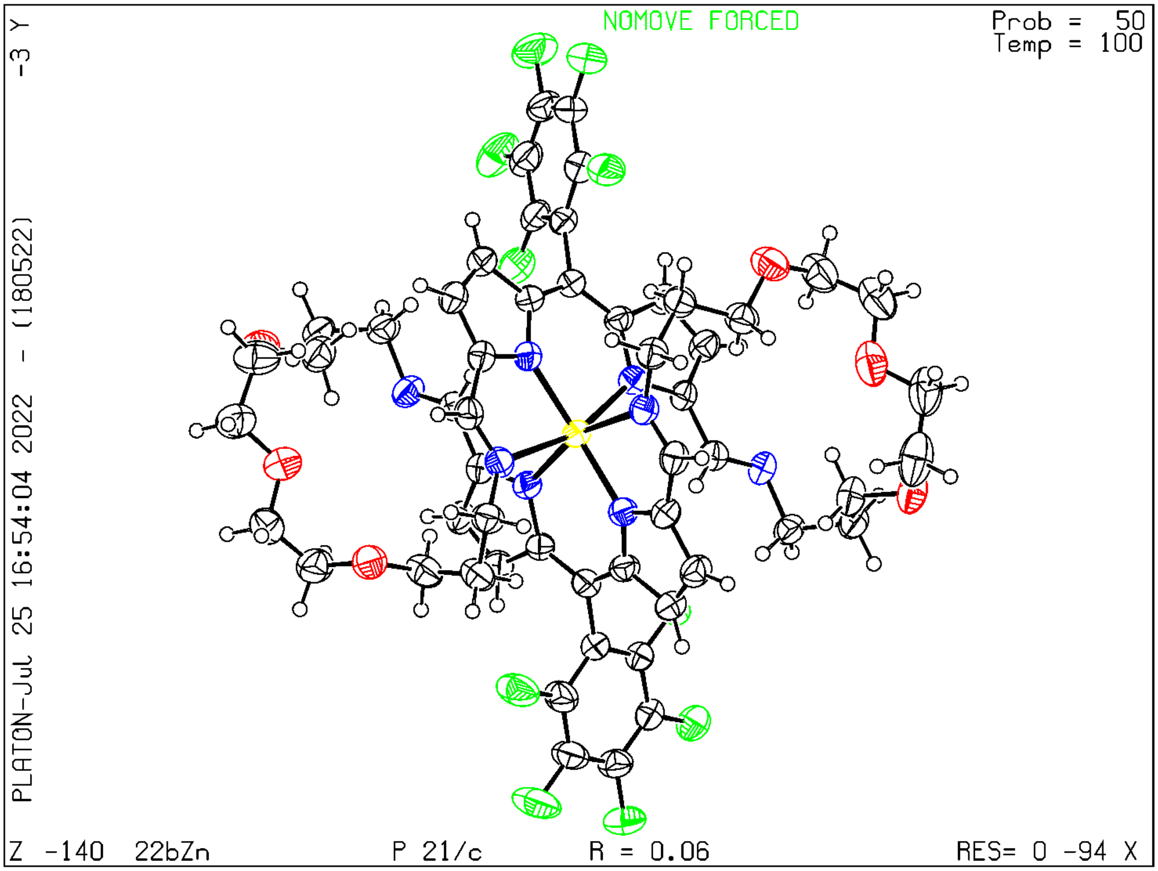

**Datablock 22bHg** - ellipsoid plot

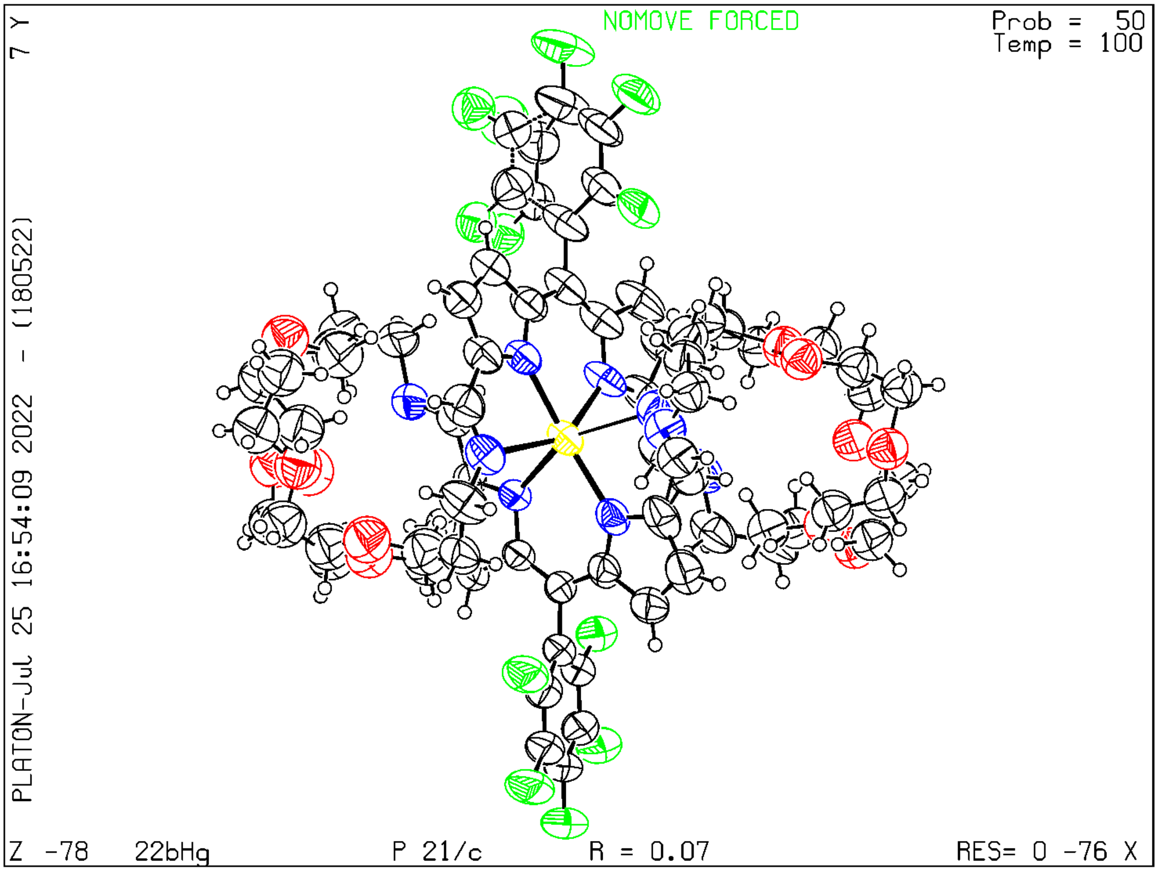

**Datablock 22bCd** - ellipsoid plot

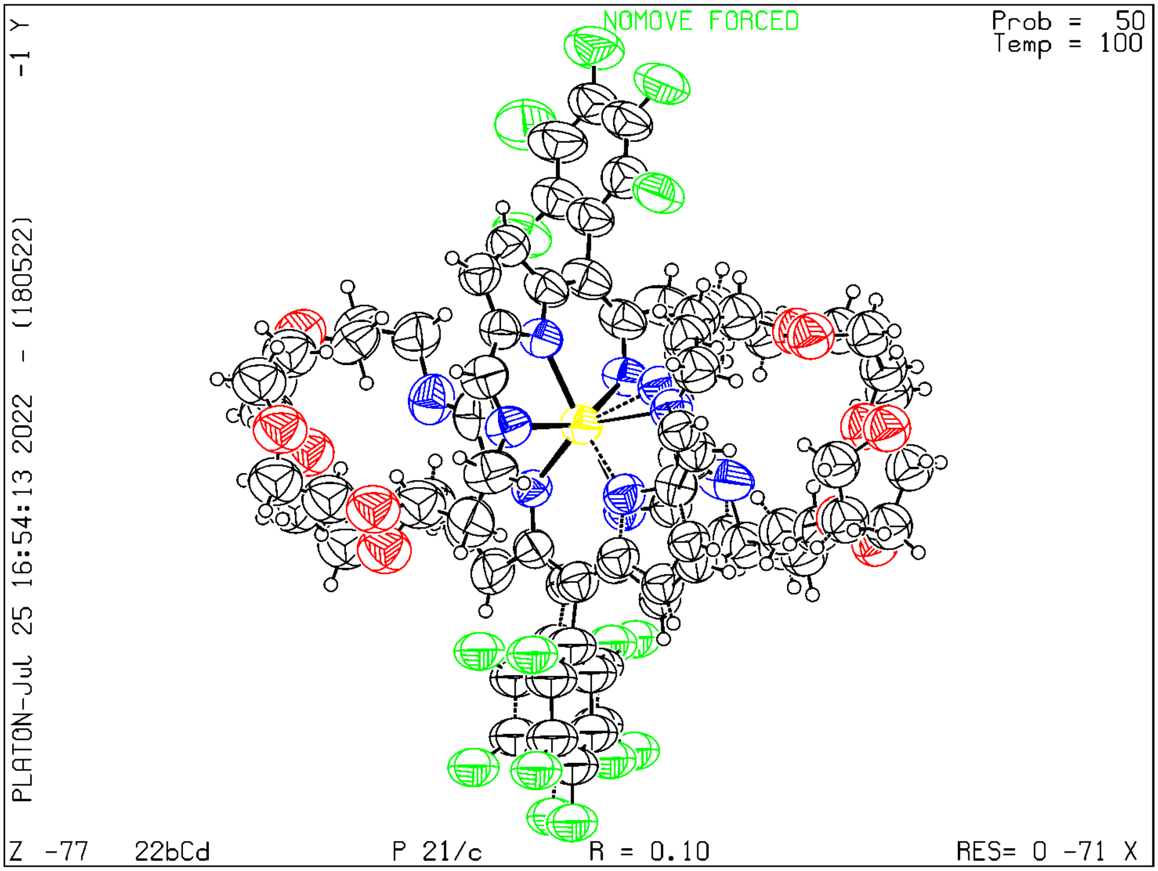

Supplement: Supplementary file 2 — Supporting Information [file ANIE-61-0-s001.pdf]
